# First-line serplulimab or placebo plus chemotherapy in PD-L1-positive esophageal squamous cell carcinoma: a randomized, double-blind phase 3 trial

**DOI:** 10.1038/s41591-022-02179-2

**Published:** 2023-02-02

**Authors:** Yan Song, Bo Zhang, Dao Xin, Xiaoge Kou, Zhenbo Tan, Shu Zhang, Meili Sun, Jin Zhou, Min Fan, Ming Zhang, Yongxiang Song, Suyi Li, Yuan Yuan, Wu Zhuang, Jingdong Zhang, Li Zhang, Hao Jiang, Kangsheng Gu, Huangyang Ye, Ying Ke, Jing Li, Qingyu Wang, Jun Zhu, Jing Huang, The ASTRUM-007 investigators

**Affiliations:** 1 Department of Medical Oncology, National Cancer Center/National Clinical Research Center for Cancer/Cancer Hospital, Chinese Academy of Medical Sciences and Peking Union Medical College, Beijing, China; 2 Department of Medical Oncology, The First Affiliated Hospital of Xinxiang Medical University, Xinxiang, China; 3 Department of Thoracic Surgery, Xingtai People’s Hospital, Xingtai, China; 4 Department of Gastrointestinal Oncology, Shandong First Medical University Cancer Hospital, Shandong Cancer Hospital, Jinan, China; 5 Department of Medical Oncology, Central Hospital Affiliated to Shandong First Medical University, Jinan, China; 6 Department of Medical Oncology, Sichuan Cancer Hospital, Chengdu, China; 7 Department of Radiation Oncology, Fudan University Shanghai Cancer Center, Shanghai, China; 8 Department of Integrated Traditional and Western Medicine, Shanghai Chest Hospital, Shanghai Jiao Tong University, Shanghai, China; 9 Department of Thoracic Surgery, Affiliated Hospital of Zunyi Medical University, Zunyi, China; 10 Department of Medical Oncology, Anhui Provincial Cancer Hospital, Hefei, China; 11 Department of Medical Oncology, Xuzhou Central Hospital, Xuzhou, China; 12 Department of Medical Oncology, Fujian Cancer Hospital, Fuzhou, China; 13 Medical Oncology Department of Gastrointestinal Cancer, Liaoning Cancer Hospital & Institute, Cancer Hospital of China Medical University, Shenyang, China; 14 Department of Oncology, Chongqing University Three Gorges Hospital, Chongqing, China; 15 Department of Radiation Oncology, The First Affiliated Hospital of Bengbu Medical College, Bengbu, China; 16 Department of Medical Oncology, The First Affiliated Hospital of Anhui Medical University, Hefei, China; 17 Department of Medical Oncology, The First Affiliated Hospital of Xiamen University, Xiamen, China; 18 Shanghai Henlius Biotech, Inc., Shanghai, China

**Keywords:** Cancer immunotherapy, Oesophageal cancer

## Abstract

First-line systemic therapeutic options for advanced esophageal squamous cell carcinoma (ESCC) are limited. In this multicenter, double-blind phase 3 trial, a total of 551 patients with previously untreated, locally advanced or metastatic ESCC and PD-L1 combined positive score of ≥1 were randomized (2:1) to receive serplulimab (an anti-PD-1 antibody; 3 mg/kg) or placebo (on day 1), plus cisplatin (50 mg/m^2^) (on day 1) and continuous infusion of 5-fluorouracil (1,200 mg/m^2^) (on days 1 and 2), once every 2 weeks. The study met the primary endpoints. At the prespecified final analysis of progression-free survival (PFS) assessed by the blinded independent radiological review committee, serplulimab plus chemotherapy significantly improved PFS compared with placebo plus chemotherapy (median PFS of 5.8 months and 5.3 months, respectively; hazard ratio, 0.60; 95% confidence interval, 0.48–0.75; *P* < 0.0001). At the prespecified interim analysis of overall survival (OS), serplulimab plus chemotherapy also significantly prolonged OS compared with placebo plus chemotherapy (median OS of 15.3 months and 11.8 months, respectively; hazard ratio, 0.68; 95% confidence interval, 0.53–0.87; *P* = 0.0020). Grade 3 or higher treatment-related adverse events occurred in 201 (53%) and 81 (48%) patients in the serplulimab plus chemotherapy group and the placebo plus chemotherapy group, respectively. Serplulimab plus chemotherapy administered every 2 weeks significantly improved PFS and OS in patients with previously untreated, PD-L1-positive advanced ESCC, with a manageable safety profile. This study is registered with ClinicalTrials.gov (NCT03958890).

## Abstract (web summary)

The combination of anti-PD-1 treatment with cisplatin and 5-fluorouracil in patients with previously untreated, PD-L1-positive esophageal squamous cell carcinoma improved progression-free survival and overall survival.

## Main

Esophageal squamous cell carcinoma (ESCC) is the predominant histological subtype of esophageal cancer and accounts for approximately 84% of all esophageal cancer cases^1^. Despite progress in chemotherapy for patients with unresectable or metastatic ESCC, the overall outcome remains poor, with median overall survival (OS) of 10–12 months (refs. ^2–6^). Therefore, there is an unmet need for new antitumor agents and therapeutic strategies for advanced ESCC.

Systemic chemotherapy remains the backbone of treatment of unresectable or metastatic ESCC. Although 5-fluorouracil (5-FU) or paclitaxel plus cisplatin has been widely used in the first-line setting, programmed cell death protein 1 (PD-1) inhibitors in combination with chemotherapy showed improved efficacy compared with chemotherapy alone in phase 3 trials^2–6^. In these trials, chemotherapy was administered every 3 or 4 weeks. A few randomized trials have demonstrated that survival of patients receiving chemotherapy regimens with shortened intervals between cycles was better than that of patients treated by standard chemotherapy in ovarian cancer, breast cancer and advanced colorectal cancer^7–9^. The results from these trials and the fact that advanced or metastatic ESCC progresses rapidly prompted us to modify the dose frequency to every 2 weeks to increase the possibility of improving efficacy.

Advances in immunotherapy have uncovered programmed cell death protein ligand 1 (PD-L1) as a potential therapeutic target and biomarker for patients with ESCC. PD-L1 overexpression was observed in up to 40% of ESCC^10^. Previously, three phase 3 clinical trials in which PD-1 inhibitors were tested as a second-line treatment in patients with ESCC who were unselected for PD-L1 expression revealed modest activity, as shown by objective response rates (ORRs) ranging from 16.7 to 20.2% (refs. ^11–13^). Although the overall ORRs were unsatisfactory, further analysis from these studies revealed that the treatment outcome was better in ESCC patients with higher PD-L1 expression^11–13^. Several phase 3 randomized studies of first-line therapy in patients with advanced ESCC have also demonstrated a positive association between PD-L1 expression and outcomes with PD-1 inhibitors plus chemotherapy^2–4^. Among them, KEYNOTE-590 revealed that patients with PD-L1 combined positive score (CPS) ≥ 10 experienced greater treatment benefit than those with PD-L1 CPS < 10 (ref. ^2^). Based on the findings of that study, pembrolizumab plus chemotherapy was approved in the United States for the first-line treatment of locally advanced or metastatic esophageal cancer regardless of PD-L1 expression^14^. This combination is also recommended by the National Comprehensive Cancer Network guidelines, but the evidence and consensus categories differ according to PD-L1 subgroup (category 1 for PD-L1 CPS ≥ 10 and category 2B for PD-L1 CPS < 10) (ref. ^15^). However, in Europe, the European Medicines Agency restricted the indication only to patients with PD-L1 CPS ≥ 10 (ref. ^16^). Therefore, to better identify candidates who may benefit from treatment with PD-1 inhibitors plus chemotherapy, further study with more specific stratification according to PD-L1 status is needed.

Serplulimab (HLX10) is a fully humanized, selective immunoglobulin G4 monoclonal antibody against PD-1 receptor^17^. Serplulimab showed promising antitumor activity and a manageable safety profile in various tumor types in phase 2 clinical trials^18,19^. A phase 3 trial of serplulimab plus chemotherapy in patients with untreated extensive-stage small cell lung cancer has reached its primary endpoint of OS^20^; another phase 3 trial in patients with untreated locally advanced or metastatic squamous non-small cell lung cancer has also reached its primary endpoint of progression-free survival (PFS)^21^. Based on these findings, we conducted a phase 3 study (ASTRUM-007) to assess the efficacy and safety of serplulimab in combination with chemotherapy (5-FU plus cisplatin) versus chemotherapy alone as a first-line treatment in patients with advanced or metastatic ESCC with PD-L1 CPS ≥ 1.

## Results

### Patients and treatment

**Fig. 1:**
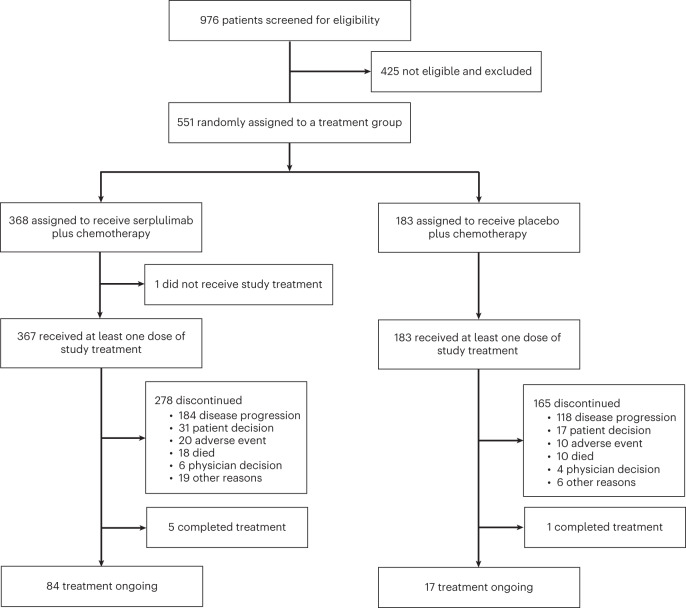
Trial profile. Among patients who were randomized to receive placebo plus chemotherapy, 15 received serplulimab plus chemotherapy because of an error in drug distribution.

A total of 976 patients were screened between 19 June 2019 and 17 December 2021, and 551 of them were randomly assigned to serplulimab plus chemotherapy (*n* = 368) or placebo plus chemotherapy (*n* = 183) (Fig. 1). Baseline patient demographics and disease characteristics were well balanced between groups (Table 1). Of the 551 patients, 470 (85%) were male. In addition, 162 (44%) of the 368 patients in the serplulimab plus chemotherapy group and 79 (43%) of the 183 patients in the placebo plus chemotherapy group had PD-L1 CPS ≥ 10. At the data cutoff date of 15 April 2022, the median follow-up duration from randomization was 14.9 months (interquartile range (IQR), 8.8–19.7 months) for patients in the serplulimab plus chemotherapy group and 15.0 months (IQR, 9.4–19.9 months) in the placebo plus chemotherapy group. A total of six patients completed 2 years of therapy. At the data cutoff date, 84 (23%) of the 368 patients in the serplulimab plus chemotherapy group and 17 (9%) of the 183 patients in the placebo plus chemotherapy group remained on treatment (Fig. 1).

**Table 1:** Baseline characteristics in the ITT population

Characteristic	Serplulimab plus CF group (*n* = 368)	Placebo plus CF group (*n* = 183)
Age (years)		
Median (IQR)	64 (57–68)	64 (57–68)
<65	199 (54%)	98 (54%)
≥65	169 (46%)	85 (46%)
Sex		
Male	317 (86%)	153 (84%)
Female	51 (14%)	30 (16%)
ECOG performance status		
0	93 (25%)	53 (29%)
1	275 (75%)	130 (71%)
Disease status^a^		
Locally advanced	46 (13%)	29 (16%)
Distantly metastatic	322 (88%)	154 (84%)
Number of organs with metastases		
1	184 (50%)	104 (57%)
≥2	184 (50%)	79 (43%)
Sites of metastases		
Lymph node	365 (99%)	182 (99%)
Lung	96 (26%)	42 (23%)
Liver	71 (19%)	32 (17%)
Bone	48 (13%)	15 (8%)
PD-L1 expression		
1 ≤ CPS < 10	206 (56%)	104 (57%)
CPS ≥ 10	162 (44%)	79 (43%)
Smoking status		
Current or former smoker	232 (63%)	115 (63%)
Never smoked	136 (37%)	68 (37%)

Data are presented as *n* (%) unless otherwise stated. Sex was recorded by the investigators according to the identity information provided by the patients. CF, cisplatin and 5-FU.

^a^Percentages may not add up to 100% owing to rounding.

In total, 550 patients received at least one dose of study treatment. The mean duration of treatment exposure was 6.1 months (s.d. 5.12 months) in the serplulimab plus chemotherapy group and 4.6 months (s.d. 3.64 months) in the placebo plus chemotherapy group. In addition, 15 patients in the placebo plus chemotherapy group received serplulimab owing to an error in drug distribution. These patients were included in the intention-to-treat (ITT) analysis set for primary efficacy analysis as randomized. However, for safety analysis, these patients were included in the treatment group of serplulimab plus chemotherapy. A total of 139 (38%) patients in the serplulimab plus chemotherapy group and 95 (52%) in the placebo plus chemotherapy group received subsequent anticancer therapy; 64 (17%) in the serplulimab plus chemotherapy group and 61 (33%) in the placebo plus chemotherapy group received subsequent immunotherapy.

### Efficacy

**Fig. 2:**
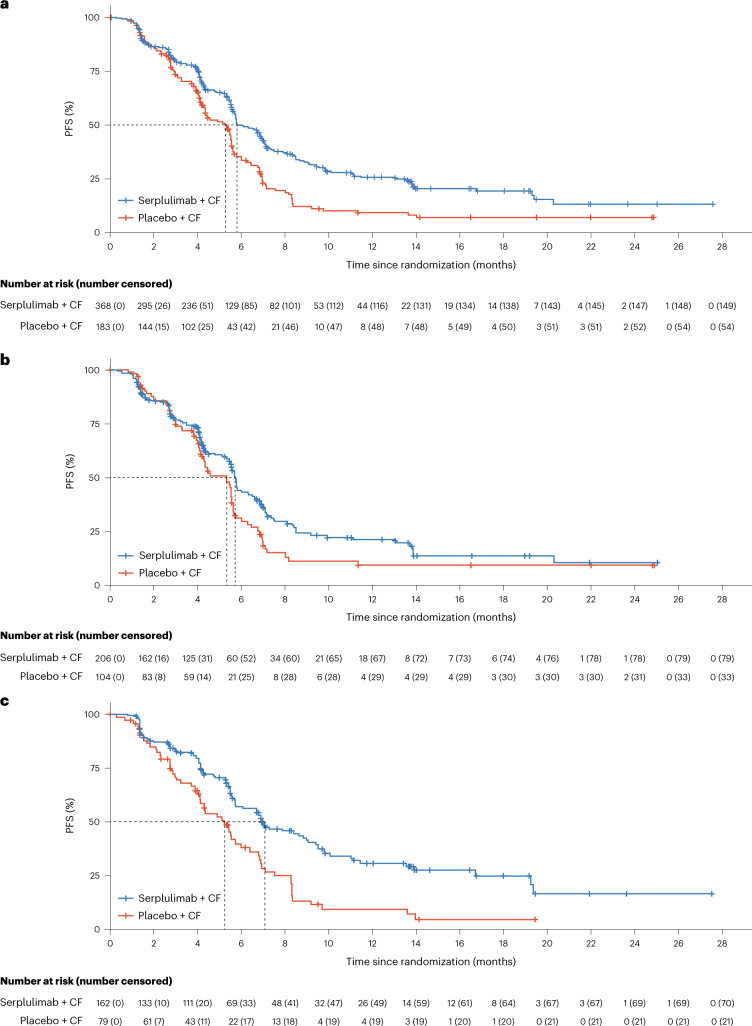
Kaplan–Meier estimates of PFS. **a**, All randomized patients. For serplulimab + CF, *n* = 368, median PFS 5.8 months (95% CI, 5.7–6.9 months). For placebo + CF, *n* = 183, median PFS 5.3 months (95% CI, 4.3–5.6 months). HR, 0.60; 95% CI, 0.48–0.75; *P* < 0.0001. **b**, Patients with PD-L1 expression level of 1 ≤ CPS < 10. For serplulimab + CF, *n* = 206, median PFS 5.7 months (95% CI, 5.5–6.3 months). For placebo + CF, *n* = 104, median PFS 5.3 months (95% CI, 4.2–5.6 months). HR, 0.70; 95% CI, 0.52–0.94; *P* = 0.017. **c**, Patients with PD-L1 CPS ≥ 10. For serplulimab + CF, *n* = 162, median PFS 7.1 months (95% CI, 5.8–9.1 months). For placebo + CF, *n* = 79, median PFS 5.3 months (95% CI, 4.1–6.0 months). HR, 0.48; 95% CI, 0.34–0.68; *P* < 0.0001. Tick marks, data censored at the time of last valid tumor assessment. PFS was assessed in accordance with RECIST v1.1 by the IRRC.

**Extended Data Fig. 1:**
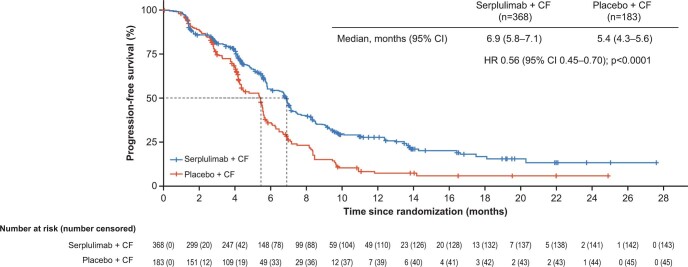
Kaplan–Meier estimates of progression-free survival assessed by investigators in the ITT population. Tick marks represent data censored at the time of last valid tumor assessment. Progression-free survival was assessed per the Response Evaluation Criteria in Solid Tumors version 1.1 by investigators. CF = cisplatin and 5-fluorouracil. CI = confidence interval. CPS = combined positive score. HR = hazard ratio. PD-L1 = programmed death ligand 1.

At the data cutoff date, 219 (60%) patients in the serplulimab plus chemotherapy group and 129 (70%) in the placebo plus chemotherapy group had disease progression or had died. At the time of this final PFS analysis, serplulimab plus chemotherapy met the criteria for superiority in prolonging PFS over placebo plus chemotherapy. The median PFS assessed by the blinded independent radiological review committee (IRRC) was 5.8 months (95% confidence interval (CI), 5.7–6.9 months) in the serplulimab plus chemotherapy group and 5.3 months (95% CI, 4.3–5.6 months) in the placebo plus chemotherapy group (stratified hazard ratio (HR), 0.60; 95% CI, 0.48–0.75; *P* < 0.0001) (Fig. 2a). Findings were similar when assessed by the investigators (stratified HR, 0.56; 95% CI, 0.45–0.70) (Extended Data Fig. 1). The 12-month PFS rate assessed by the IRRC in the serplulimab plus chemotherapy group was 2.8 times that in the placebo plus chemotherapy group (26% versus 9%, respectively).

**Fig. 3:**
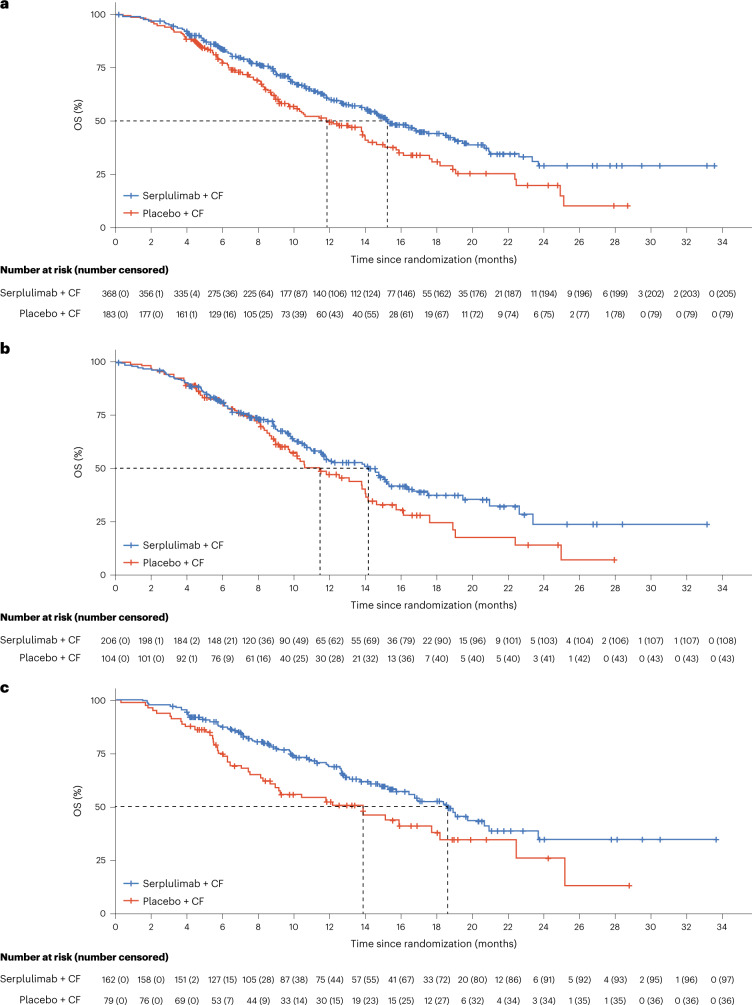
Kaplan–Meier estimates of OS. **a**, All randomized patients. For serplulimab + CF, *n* = 368, median OS 15.3 months (95% CI, 14.0–18.6 months). For placebo + CF, *n* = 183, median OS 11.8 months (95% CI, 9.7–14.0 months). HR, 0.68; 95% CI, 0.53–0.87; *P* = 0.0020. **b**, Patients with PD-L1 expression level of 1 ≤ CPS < 10. For serplulimab + CF, *n* = 206, median OS 14.2 months (95% CI, 11.5–15.3 months). For placebo + CF, *n* = 104, median OS 11.4 months (95% CI, 9.2–14.0 months). HR, 0.74; 95% CI, 0.54–1.03; *P* = 0.066. **c**, Patients with PD-L1 CPS ≥ 10. For serplulimab + CF, *n* = 162, median OS 18.6 months (95% CI, 15.3–20.9 months). For placebo + CF, *n* = 79, median OS 13.9 months (95% CI, 8.3–18.2 months). HR, 0.59; 95% CI, 0.40–0.88; *P* = 0.0082. Tick marks, data censored on the last known survival date.

At the time of this interim analysis of OS, there had been 163 (44%) death events in the serplulimab plus chemotherapy group and 104 (57%) in the placebo plus chemotherapy group. The OS in the serplulimab plus chemotherapy group was significantly longer than that in the placebo plus chemotherapy group (median OS of 15.3 months (95% CI, 14.0–18.6 months) versus 11.8 months (95% CI, 9.7–14.0 months), respectively; stratified HR, 0.68; 95% CI, 0.53–0.87; *P* = 0.0020) (Fig. 3a). Both Schoenfeld residual plots of PFS and OS showed that there were no time-related trends, which indicated that proportional hazard assumptions were met.

According to IRRC assessments, the confirmed ORR was 57.6% (95% CI, 52.4–62.7%; 50 (14%) complete responses) in the serplulimab plus chemotherapy group and 42.1% (95% CI, 34.8–49.6%; 12 (7%) complete responses) in the placebo plus chemotherapy group (odds ratio, 1.85; 95% CI, 1.29–2.65; *P* = 0.0007) (Extended Data Table 1). The median confirmed duration of response (DOR) was 6.9 months (95% CI, 5.6–8.3 months) in the serplulimab plus chemotherapy group and 4.6 months (95% CI, 4.1–5.6 months) in the placebo plus chemotherapy group, with a 6-month DOR rate of 53 and 32%, 12-month DOR rate of 36 and 15% and 18-month DOR rate of 20 and 12% in the respective groups. The unconfirmed ORR assessed by the IRRC in the serplulimab plus chemotherapy group versus placebo plus chemotherapy group was 65.8% versus 50.3% (odds ratio, 1.93; 95% CI, 1.34–2.78; *P* = 0.0004), with an unconfirmed DOR of 5.8 months versus 4.2 months in the respective groups (Extended Data Table 1). Antitumor responses assessed by investigators were similar to those assessed by the IRRC (Extended Data Table 2).

**Fig. 4:**
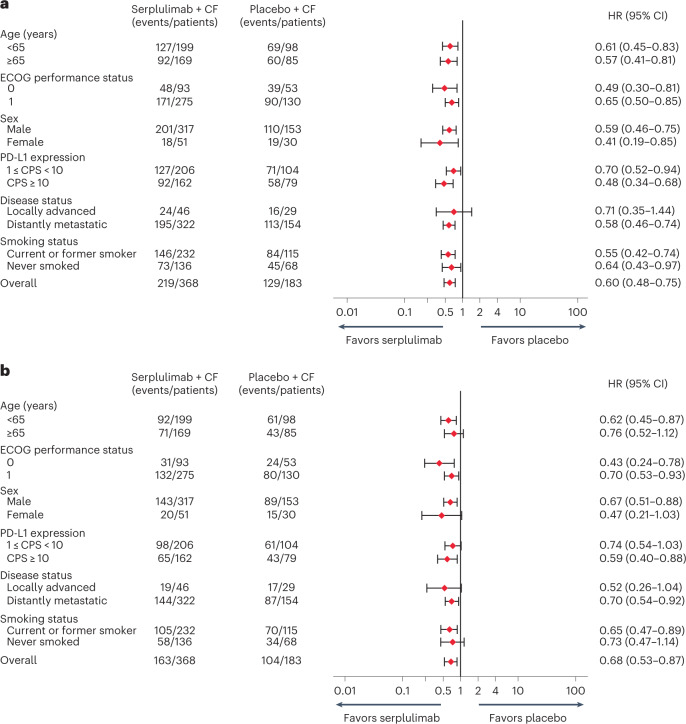
Survival by patient subgroups. **a**,**b**, Forest plot analysis of PFS (**a**) and OS (**b**) in prespecified and post-hoc (smoking status) subgroups in the ITT population for patients receiving serplulimab plus chemotherapy (*n* = 368) versus placebo plus chemotherapy (*n* = 183). PFS was assessed in accordance with RECIST v1.1 by the IRRC. The Cox proportional hazards model with Efron’s method of tie handling was used to assess the magnitude of the treatment difference between groups. Sex was recorded by the investigators according to the identity information provided by the patients.

The HRs for PFS and OS indicated that the survival benefits of adding serplulimab to chemotherapy were generally consistent across prespecified and post-hoc (smoking status) subgroups (Fig. 4). In patients with PD-L1 1 ≤ CPS < 10, the median PFS assessed by the IRRC was 5.7 months in the serplulimab plus chemotherapy group and 5.3 months in the placebo plus chemotherapy group (HR, 0.70; 95% CI, 0.52–0.94; Fig. 2b), and the median OS was 14.2 and 11.4 months (HR, 0.74; 95% CI, 0.54–1.03; Fig. 3b) in the respective groups, both showing a strong trend toward improved survival with the addition of serplulimab. In patients with PD-L1 CPS ≥ 10, adding serplulimab to chemotherapy led to greater survival benefits than that in all randomized patients; the median PFS was 7.1 months in the serplulimab plus chemotherapy group and 5.3 months in the placebo plus chemotherapy group (HR, 0.48; 95% CI, 0.34–0.68; Fig. 2c), and the median OS was 18.6 and 13.9 months (HR, 0.59; 95% CI, 0.40–0.88; Fig. 3c), respectively. Antitumor responses were also improved by serplulimab in both the PD-L1 CPS ≥ 10 and 1 ≤ CPS < 10 subgroups (Extended Data Table 3).

### Safety

Adverse events were coded by Medical Dictionary for Regulatory Activities (v.25.0) and graded per National Cancer Institute Common Terminology Criteria for Adverse Events (v.4.03). Treatment-related adverse events of any grade were observed in 376 (98%) patients in the serplulimab plus chemotherapy group and 165 (98%) in the placebo plus chemotherapy group (Extended Data Table 4). Grade 3 or higher treatment-related adverse events occurred in 201 (53%) patients in the serplulimab plus chemotherapy group and 81 (48%) in the placebo plus chemotherapy group, with deaths due to treatment-related adverse events occurring in 11 (3%) and 3 (2%) patients, respectively. The most commonly reported grade 3 or higher treatment-related adverse events were ‘anemia’ (67 (18%) in the serplulimab plus chemotherapy group versus 34 (20%) in the placebo plus chemotherapy group), ‘neutrophil count decreased’ (71 (19%) in the serplulimab plus chemotherapy group versus 29 (17%) in the placebo plus chemotherapy group) and ‘white blood cell count decreased’ (43 (11%) in the serplulimab plus chemotherapy group versus 11 (7%) in the placebo plus chemotherapy group) (Table 2). Treatment-related adverse events leading to treatment discontinuation occurred in 130 (34%) patients in the serplulimab plus chemotherapy group and 39 (23%) in the placebo plus chemotherapy group (Extended Data Table 4). Treatment-related serious adverse events occurred in 139 (36%) patients in the serplulimab plus chemotherapy group and 54 (32%) in the placebo plus chemotherapy group, with platelet count decreased (26 (7%) versus 2 (1%), respectively) and anemia (18 (5%) versus 4 (2%), respectively) being the most common events.

**Table 2:** Adverse events in the safety set

Adverse event	Serplulimab plus CF group (*n* = 382^a^)		Placebo plus CF group (*n* = 168)	
	Any grade	Grade ≥ 3	Any grade	Grade ≥ 3
Any treatment-emergent adverse events	379 (99%)	243 (64%)	167 (99%)	99 (59%)
Treatment-related adverse events^b^				
Anemia	290 (76%)	67 (18%)	126 (75%)	34 (20%)
Nausea	244 (64%)	11 (3%)	106 (63%)	5 (3%)
White blood cell count decreased	222 (58%)	43 (11%)	102 (61%)	11 (7%)
Neutrophil count decreased	213 (56%)	71 (19%)	90 (54%)	29 (17%)
Platelet count decreased	165 (43%)	15 (4%)	68 (40%)	3 (2%)
Vomiting	165 (43%)	12 (3%)	71 (42%)	5 (3%)
Appetite decreased	161 (42%)	6 (2%)	66 (39%)	0
Asthenia	113 (30%)	3 (1%)	49 (29%)	1 (1%)
Blood creatinine increased	60 (16%)	1 (<1%)	20 (12%)	1 (1%)
Hyponatremia	56 (15%)	18 (5%)	19 (11%)	4 (2%)
Hypoalbuminemia	56 (15%)	1 (<1%)	17 (10%)	0
Constipation	56 (15%)	0	21 (13%)	0
Proteinuria	49 (13%)	1 (<1%)	9 (5%)	0
Weight decreased	47 (12%)	0	18 (11%)	1 (1%)
Aspartate aminotransferase increased	45 (12%)	6 (2%)	7 (4%)	0
Hypokalemia	43 (11%)	14 (4%)	16 (10%)	6 (4%)
Lymphocyte count decreased	42 (11%)	11 (3%)	17 (10%)	4 (2%)
Immune-mediated hypothyroidism	41 (11%)	1 (<1%)	4 (2%)	0
Alanine aminotransferase increased	39 (10%)	6 (2%)	7 (4%)	1 (1%)
Diarrhea	38 (10%)	2 (1%)	13 (8%)	1 (1%)
Hypercholesterolemia	32 (8%)	0	8 (5%)	0
Pyrexia	30 (8%)	2 (1%)	7 (4%)	1 (1%)
Hypothyroidism	30 (8%)	0	9 (5%)	0
Hypomagnesemia	29 (8%)	1 (<1%)	9 (5%)	1 (1%)
Hyperuricemia	28 (7%)	2 (1%)	8 (5%)	1 (1%)
Blood bilirubin increased	27 (7%)	3 (1%)	6 (4%)	2 (1%)
Immune-mediated dermatitis	24 (6%)	6 (2%)	5 (3%)	0
Blood urea increased	24 (6%)	0	6 (4%)	0
Hypoproteinemia	19 (5%)	0	4 (2%)	0
Hypochloremia	13 (3%)	6 (2%)	10 (6%)	3 (2%)

Data are presented as *n* (%). The safety set included all patients who received at least one dose of study treatment. Adverse events were recorded from the first dose of study treatment until 90 days after the last dose or the start of new antitumor treatment, whichever occurred first. Eleven (3%) patients receiving serplulimab plus chemotherapy and three (2%) receiving placebo plus chemotherapy experienced grade 5 treatment-related adverse events. These events included pneumonitis that occurred in four patients, myocarditis in two patients, pneumonia in two patients, dermatitis in one patient, esophageal bleeding in one patient and death of unknown cause in one patient receiving serplulimab plus chemotherapy; and esophageal bleeding in two patients and death of unknown cause in one patient receiving placebo plus chemotherapy.

^a^Includes 15 patients who were randomized to receive placebo plus chemotherapy but received serplulimab plus chemotherapy because of an error in drug distribution.

^b^Treatment-related adverse events with an incidence of 5% or higher in any grade category in either group are shown.

Immune-related adverse events occurred in 132 (35%) patients in the serplulimab plus chemotherapy group and 30 (18%) in the placebo plus chemotherapy group, with ‘immune-mediated hypothyroidism’ (41 (11%) versus 4 (2%), respectively), ‘immune-mediated dermatitis’ (24 (6%) versus 5 (3%), respectively) and ‘immune-mediated hyperthyroidism’ (17 (4%) versus 4 (2%), respectively) being the most common (Extended Data Table 5). Grade 3 or higher immune-related adverse events occurred in 33 (9%) patients in the serplulimab plus chemotherapy group and in 4 (2%) in the placebo plus chemotherapy group. Grade 5 immune-related adverse events were observed in 6 (2%) patients in the serplulimab plus chemotherapy group; there was no grade 5 immune-related adverse event in the placebo plus chemotherapy group.

## Discussion

In this double-blind, placebo-controlled, randomized phase 3 study, we evaluated the efficacy and safety of first-line serplulimab plus chemotherapy versus placebo plus chemotherapy in patients with locally advanced or metastatic, PD-L1-positive (CPS ≥ 1) ESCC. In our prespecified final analysis for PFS and interim analysis for OS, the addition of serplulimab to cisplatin and 5-FU chemotherapy resulted in a significant improvement in both efficacy endpoints. In addition, the safety profile of serplulimab plus chemotherapy was manageable. Other planned secondary endpoints that were not reported in this manuscript but that will be reported in the future are PFS, ORR and DOR assessed by IRRC and by investigators based on immune-response evaluation criteria in solid tumors (iRECIST), pharmacokinetics and immunogenicity of serplulimab, quality-of-life assessment, the relationship between efficacy and microsatellite instability and the relationship between efficacy and tumor mutation burden. In this study, we demonstrate an improvement in survival with the addition of PD-1 blockade to chemotherapy administered every 2 weeks in a solely PD-L1-positive population with advanced ESCC.

Anti-PD-1 antibodies have changed the systemic treatment of advanced esophageal cancer in recent years. In the first-line setting, results from five previous phase 3 studies showed that PD-1 inhibitors plus chemotherapy provided significant improvement in OS over chemotherapy alone in patients with advanced ESCC^2–6^. Consistent with this, in the present study, serplulimab combined with chemotherapy reduced risk of death by 32% compared with chemotherapy alone in patients with PD-L1-positive (CPS ≥ 1), advanced ESCC. The respective estimated survival rates at 12 months and 24 months were 11% and 9% higher with serplulimab plus chemotherapy, suggesting a long-term benefit. Although the difference in median PFS was modest between the two treatment groups (5.8 months for the serplulimab plus chemotherapy group versus 5.3 months for the placebo plus chemotherapy group), the Kaplan–Meier curves for PFS favored serplulimab plus chemotherapy, with a stratified HR of 0.60, and 16% more patients in the serplulimab plus chemotherapy group remained progression free at 12 months. In our trial, 64 of 368 (17%) patients in the serplulimab plus chemotherapy group and 61 of 183 (33%) patients in the placebo plus chemotherapy group received subsequent immunotherapy. The percentages of patients receiving subsequent immunotherapy were comparable with those in several other studies conducted recently in China^4–6^. We also observed a clinically meaningful improvement in ORR with the addition of serplulimab (57.6% versus 42.1%). Collectively, our findings and previous evidence establish first-line PD-1 blockade combined with chemotherapy as one of the standard treatments for patients with advanced ESCC.

Cisplatin plus 5-FU is commonly adopted in the first-line treatment of advanced ESCC, and different schedules for drug delivery exist for this combination. The dose intensities of cisplatin and 5-FU in our 2-week schedule were similar to those in the KEYNOTE-590 and CHECKMATE-648 trials, which used the same chemotherapy backbone^2,3^, but with a lower dosage per cycle delivered within fewer treatment days. Our chemotherapy schedule was well tolerated, as the incidences of hematological toxicities, nausea and vomiting were comparable with those observed in the KEYNOTE-590 and CHECKMATE-648 trials. The ORR assessed by the IRRC in the placebo plus chemotherapy group was 42.1% in our trial, which is higher than those reported for the chemotherapy group in KEYNOTE-590 (27% in all randomized patients and 20% in the Chinese subgroup) and in CHECKMATE-648 (29.3%)^2,3,22^, and comparable with historical data from phase 2 studies (35–35.9%)^23,24^. Although there are limitations regarding cross-trial comparisons, we may conclude that the chemotherapy backbone in our present study did not underperform and could reflect standard of care before the recent progress with PD-1 inhibitors. Our 2-week chemotherapy regimen is therefore an effective option to accompany PD-1 blockade in the first-line treatment of advanced ESCC.

The expression of PD-L1, assessed with the 22C3, 28-8 or SP263 antibodies, has been considered a potential biomarker for efficacy in patients with advanced ESCC treated with anti-PD-1 antibodies^25^. Subgroup analyses from previous phase 3 studies investigating first-line PD-1 inhibition in patients with ESCC have shown that those with higher PD-L1 expression may derive more favorable outcomes from the addition of PD-1 inhibitors than their counterparts with low or no PD-L1 expression^2–4^. However, there remain unanswered questions. First, two main scoring algorithms, tumor proportion score (TPS) and CPS, were used across different trials. TPS is calculated using only PD-L1–expressing tumor cells, whereas CPS captures PD-L1-positive lymphocytes and macrophages. Given the known temporal and spatial intratumoral heterogeneity that is a hallmark of advanced ESCC and its microenvironment^26^, CPS may be more likely than TPS to identify a greater proportion of patients who may benefit from anti-PD-1 therapy and was therefore used in the present trial for patient screening. Nevertheless, this hypothetical superiority has not been confirmed. Second, the optimal cutoff threshold for PD-L1 positivity has not been established yet. Subgroup analyses have been performed according to different levels of CPS in the KEYNOTE-590, JUPITER-06 and ORIENT-15 trials^2,5,6^. In the KEYNOTE-590 trial, patients with ESCC and esophageal adenocarcinoma with PD-L1 CPS ≥ 10 achieved better OS outcomes from the addition of pembrolizumab to cisplatin and 5-FU (HR, 0.62; 95% CI, 0.49–0.78) than those with PD-L1 CPS < 10 (HR, 0.86; 95% CI, 0.68–1.10)^2^. Similarly, in the present trial, CPS was predictive of the degree of OS benefit. The prespecified subgroup analyses showed that patients with PD-L1 CPS ≥ 10 had an HR of 0.59 (95% CI, 0.40–0.88), compared with 0.74 (95% CI, 0.54–1.03) for those with 1 ≤ CPS < 10. However, in the JUPITER-06 and ORIENT-15 trials, toripalimab or sintilimab added to chemotherapy provided a similar degree of OS benefit in patients with PD-L1 CPS ≥ 10 and CPS < 10 (HR of 0.64 for CPS ≥ 10 versus 0.61 for CPS < 10 with the toripalimab combination, and HR of 0.64 for CPS ≥ 10 versus 0.62 for CPS < 10 with the sintilimab combination)^5,6^. These findings suggest an uncertain predictive role of CPS in patients with low scores. Whether it would be useful to select a lower cutoff threshold, or to combine other biomarkers for clinical decision in patients with ESCC with low PD-L1 CPS, requires further investigation.

Serplulimab combined with cisplatin and 5-FU delivered every 2 weeks was well tolerated in our trial. The incidences of grade 3 or higher treatment-related adverse events, serious adverse events and adverse events leading to death were similar between the two treatment groups. A higher incidence of treatment-related adverse events leading to treatment discontinuation was observed in patients treated with serplulimab plus chemotherapy; this was probably related to the immune-related adverse events induced by serplulimab and a longer treatment duration in this group. Similar findings have also been reported in other controlled trials of PD-1 inhibitors in combination with chemotherapy in patients with ESCC^2,3,5,6^. Moreover, the treatment-related adverse events observed in our trial were consistent with those observed previously with serplulimab, cisplatin and 5-FU, with no new safety issues identified.

Our study had some limitations. First, we included only patients from China; however, our results might be extrapolated to patients with ESCC outside China, as no differences were noted in terms of survival between Asian and non-Asian patients in the CHECKMATE-648 study. In addition, the presence of liver or lung metastases at baseline was not considered in stratification. These common visceral metastases are representative of tumor burden and may affect treatment outcomes. Furthermore, biomarkers other than PD-L1 expression were not investigated in our current analysis.

In conclusion, first-line serplulimab in combination with chemotherapy significantly improved PFS and OS in patients with previously untreated, PD-L1-positive, locally advanced or metastatic ESCC, compared with chemotherapy alone, with a manageable safety profile.

## Methods

### Ethics statement

The study was performed in accordance with the Declaration of Helsinki and the International Conference on Harmonisation Good Clinical Practice guidelines. The study protocol was approved by the institutional review boards or ethics committees of all participating centers (the ethics committee of the leading clinical center was the Ethics Committee of National Cancer Center/Cancer Hospital, Chinese Academy of Medical Sciences and Peking Union Medical College). All patients provided written informed consent before participating in the study. Patients received compensation as described in detail in the informed consent form.

### Study design and participants

ASTRUM-007 was a randomized, placebo-controlled, double-blind, phase 3 clinical study conducted at 70 hospitals in China (Supplementary Table 1). Eligible patients were aged 18–75 years with previously untreated, histologically confirmed, inoperable locally advanced or metastatic, PD-L1-positive (CPS ≥ 1) ESCC, with at least one measurable lesion based on central imaging in accordance with RECIST v1.1, adequate organ function, and Eastern Cooperative Oncology Group (ECOG) performance status 0–1. Tumors were centrally tested for PD-L1 immunohistochemistry (22C3 PharmDx kit, Dako North America). CPS is defined as the number of PD-L1–staining cells (tumor cells, lymphocytes and macrophages) as a proportion of the total number of viable tumor cells multiplied by 100. Patients were excluded if they had previously received PD-1 or PD-L1 inhibitors, had central nervous system metastases or presented with active infection or active autoimmune diseases. The full study protocol is provided in the Supplementary Information.

### Randomization and masking

Eligible patients were randomly assigned (2:1) using an integrated web response system to receive serplulimab plus chemotherapy or placebo plus chemotherapy. Randomization was stratified by PD-L1 expression level (CPS ≥ 10 versus CPS < 10), age (≥65 years versus <65 years) and disease status (locally advanced versus distantly metastatic). Patients, investigators and the sponsor’s study team were masked to group assignment.

### Procedures

Patients received serplulimab or placebo (3 mg kg^−1^) on day 1 once every 2 weeks for up to 2 years. All patients received cisplatin (50 mg m^−^^2^) on day 1 for up to 8 cycles and 5-FU (1,200 mg m^−^^2^) continuous administration daily on days 1 and 2 of each cycle for up to 12 cycles, both administered intravenously every 2 weeks. Treatment was continued until disease progression, intolerable toxicities, investigator decision, patient withdrawal of consent, completion of 2 years of therapy or death, whichever occurred first.

Tumor imaging was scheduled once every 6 weeks for 48 weeks from randomization and every 12 weeks thereafter. Response was assessed according to RECIST v1.1 by the blinded IRRC and the investigators locally. During follow-up, patients were contacted every 12 weeks to assess survival. Adverse events and laboratory abnormalities were graded according to the National Cancer Institute Common Terminology Criteria for Adverse Events (v.4.03). Additional details regarding the study treatments, including dose interruptions and modifications, are provided in the study protocol.

### Outcomes

The dual primary endpoints were PFS (time from randomization to first disease progression or death) assessed by the IRRC in accordance with RECIST v1.1 and OS (time from randomization to death due to any cause). Secondary endpoints included IRRC-assessed PFS using iRECIST^27^, investigator-assessed PFS using RECIST v1.1 and iRECIST, ORR, DOR, safety and tolerability, quality of life and investigation of the relationship between biomarkers and clinical outcomes. Details of all study endpoints are available in the study protocol.

### Statistical analysis

The planned sample size was 540 patients, with 339 PFS events and 388 OS events needed to achieve a power of 80% to show an HR of 0.68 for PFS at a one-sided α level of 0.005 and an HR of 0.73 for OS at a one-sided α level of 0.02 for comparison between the serplulimab plus chemotherapy group and the placebo plus chemotherapy group. The primary efficacy analyses were conducted in the ITT population. All randomized patients who received at least one dose of study medication were included in the analysis of safety. The Kaplan–Meier method was used to estimate OS, PFS and DOR. The reverse Kaplan–Meier method was used to estimate the median follow-up duration. Between-group differences in OS and PFS were assessed using the stratified log-rank test and the Cox proportional hazards model. The Schoenfeld residual test was planned to check the proportional hazard assumption. Differences in ORR were assessed using the Cochran–Mantel–Haenszel test and logistic regression.

The statistical analysis plan specified one interim analysis and a final analysis of OS. The interim analysis of OS was planned to be performed during the final analysis of PFS, when approximately 339 IRRC-assessed PFS events had been observed in the ITT population.

The protocol prespecified two primary hypotheses that were tested in parallel: (1) the superiority of serplulimab plus chemotherapy over placebo plus chemotherapy for PFS (assessed by the IRRC in accordance with RECIST v1.1) in all randomized patients; and (2) the superiority of serplulimab plus chemotherapy over placebo plus chemotherapy for OS in all randomized patients. The study was considered successful if serplulimab plus chemotherapy was superior to placebo plus chemotherapy for any primary endpoint. The threshold for statistical significance was 0.01 (two-sided) for the final log-rank analysis of PFS and 0.01 (two-sided) for the interim log-rank analysis of OS (adjusted according to the actual 266 OS events and O’Brien–Fleming-like α-spending function). An O’Brien–Fleming-like α-spending function (Lan–DeMets approximation) was used to control the overall type I error rate. The significance level for each analysis could be modified based on the actual number of PFS and OS events reached at the analytical time point.

The independent data monitoring committee confirmed that the study met the specified efficacy endpoints after reviewing the results of the final analysis of PFS and the interim analysis of OS conducted by an unblinded external statistician. The trial is continuing in order to evaluate outcomes with additional follow-up. All data reported here are based on the interim analysis, with a data cutoff date of 15 April 2022. The statistical analysis plan is available in the Supplementary Information. Sample size calculation and statistical analyses were done using SAS (v9.4). This trial is registered with ClinicalTrials.gov (NCT03958890).

### Reporting summary

Further information on research design is available in the Nature Portfolio Reporting Summary linked to this article.

## Online content

Any methods, additional references, Nature Portfolio reporting summaries, source data, extended data, supplementary information, acknowledgements, peer review information; details of author contributions and competing interests; and statements of data and code availability are available at 10.1038/s41591-022-02179-2.

### Supplementary information

Supplementary Information Supplementary Table 1, study protocol and statistical analysis plan.

Reporting Summary

## Figures and Tables

**Extended Data Table 1:**
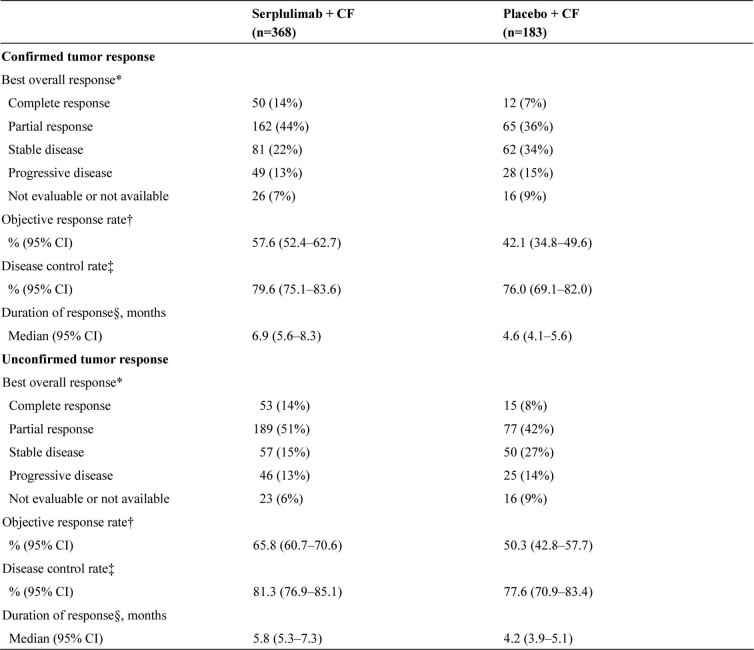
Antitumor response assessed by independent radiological review committee in the ITT population

Data are n (%) unless otherwise stated. Antitumor response was assessed per the Response Evaluation Criteria in Solid Tumors version 1.1. CF = cisplatin and 5-fluorouracil. CI = confidence interval. *Percentages may not add up to 100% due to rounding. †Objective response rate was defined as proportion of patients achieving complete or partial response. ‡Disease control rate was defined as proportion of patients achieving complete or partial response, or stable disease. §Duration of response was assessed in patients who achieved complete or partial response and defined as time from first objective response to disease progression or death from any cause.

**Extended Data Table 2:**
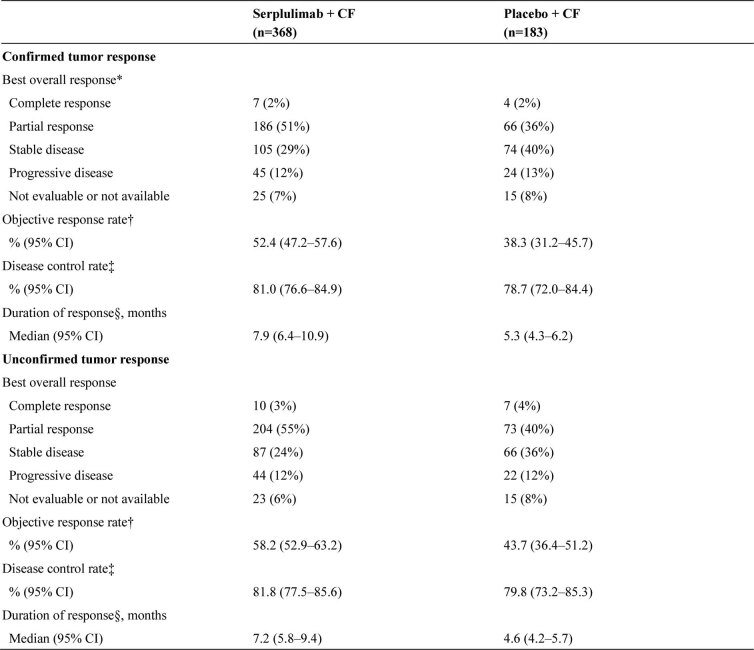
Antitumor response assessed by investigators in the ITT population

Data are n (%) unless otherwise stated. Antitumor response was assessed per the Response Evaluation Criteria in Solid Tumors version 1.1. CF = cisplatin and 5-fluorouracil. CI = confidence interval. *Percentages may not add up to 100% due to rounding. †Objective response rate was defined as proportion of patients achieving complete or partial response. ‡Disease control rate was defined as proportion of patients achieving complete or partial response, or stable disease. §Duration of response was assessed in patients who achieved complete or partial response and defined as time from first objective response to disease progression or death from any cause.

**Extended Data Table 3:**
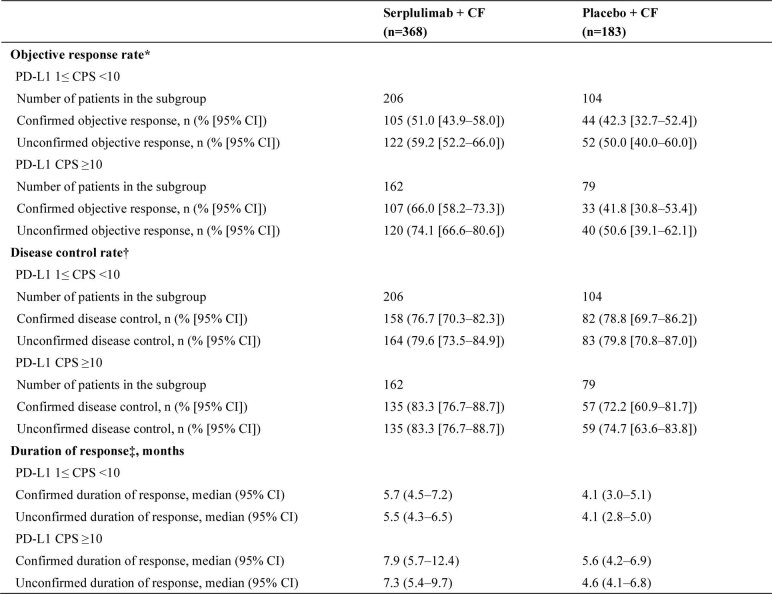
Subgroup analysis of antitumor response assessed by independent radiological review committee in the ITT population

Antitumor response was assessed per the Response Evaluation Criteria in Solid Tumors version 1.1. CF = cisplatin and 5-fluorouracil. CI = confidence interval. CPS = combined positive score. CR = complete response. PD-L1 = programmed death ligand 1. PR = partial response. SD = stable disease. *Objective response rate was defined as proportion of patients achieving complete or partial response. †Disease control rate was defined as proportion of patients achieving complete response, partial response, or stable disease. ‡Duration of response was assessed in patients who achieved complete or partial response and defined as time from first objective response to disease progression or death from any cause.

**Extended Data Table 4:**
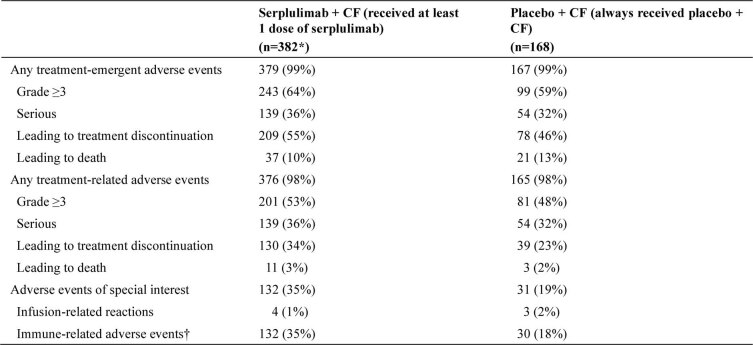
Summary of adverse events in the safety set

Data are n (%). The safety set included all patients who received at least one dose of study treatment. Adverse events were recorded from the first dose of study treatment until 90 days after the last dose or the start of new antitumor treatment, whichever occurred first. CF = cisplatin and 5-fluorouracil. *Inclusive of 15 patients who were randomized to receive placebo plus chemotherapy, but received serplulimab plus chemotherapy due to an error in drug distribution. †Immune-related adverse events were defined as adverse events that were associated with drug exposure and demonstrated immune-mediated mechanisms with no other unequivocal etiology.

**Extended Data Table 5:**
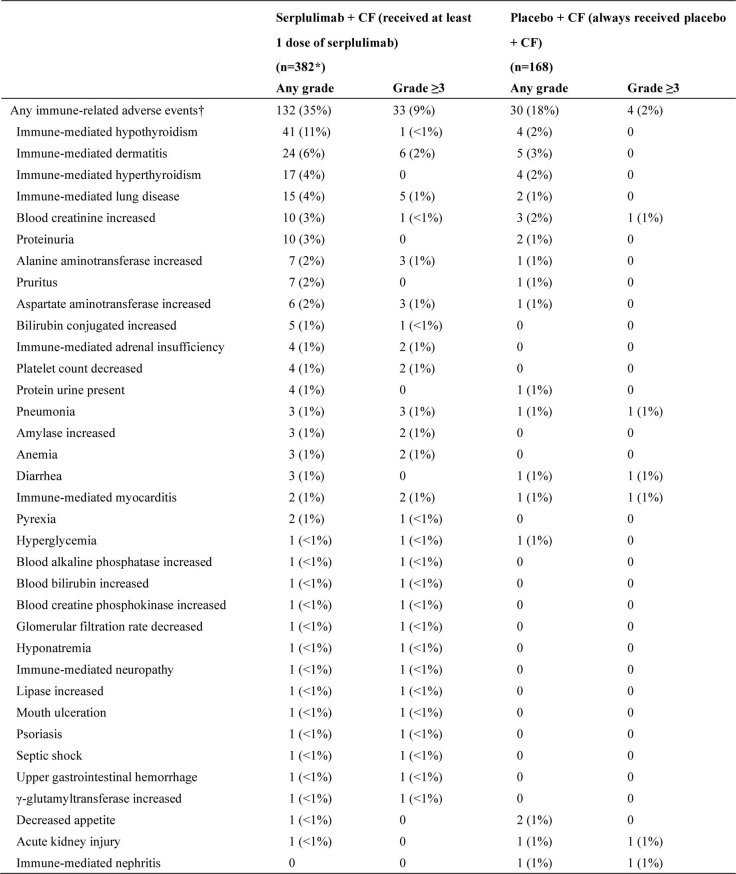
Immune-related adverse events

Data are n (%). Immune-related adverse events of any grade occurring in ≥1% of patients in either group or any immune-related adverse events of grade ≥3 are shown. Six (2%) patients receiving serplulimab plus chemotherapy reported grade 5 immune-related adverse events (two reported immune-mediated lung disease, two reported immune-mediated myocarditis, one reported immune-mediated lung disease and septic shock, and one reported immune-mediated dermatitis). No grade 5 immune-related adverse event occurred in patients receiving placebo plus chemotherapy. Adverse events were recorded from the first dose of study treatment until 90 days after the last dose or the start of new antitumor treatment, whichever occurred first. CF = cisplatin and 5-fluorouracil. *Inclusive of 15 patients who were randomized to receive placebo plus chemotherapy, but received serplulimab plus chemotherapy due to an error in drug distribution. †Immune-related adverse events were defined as adverse events that were associated with drug exposure and demonstrated immune-mediated mechanisms with no other unequivocal etiology.

## Data Availability

All requests for data will be reviewed by the leading clinical site (National Cancer Center/National Clinical Research Center for Cancer/Cancer Hospital, Chinese Academy of Medical Sciences and Peking Union Medical College) and the sponsor (Shanghai Henlius Biotech, Inc) to verify whether the request is subject to any intellectual property or confidentiality obligations. Requests for access to the patient-level data from this study can be submitted via email to J.H. (huangjingwg@163.com) with detailed proposals for use of information, and responses to such requests can be expected within 1 month. A signed data access agreement with the sponsor is required before accessing shared data.

## Code availability

No custom code was used for statistical analysis in this study.

## References

[1] Arnold M, Ferlay J, van Berge Henegouwen MI, Soerjomataram I (2020). Global burden of oesophageal and gastric cancer by histology and subsite in 2018. Gut.

[2] Sun JM (2021). Pembrolizumab plus chemotherapy versus chemotherapy alone for first-line treatment of advanced oesophageal cancer (KEYNOTE-590): a randomised, placebo-controlled, phase 3 study. Lancet.

[3] Doki Y (2022). Nivolumab combination therapy in advanced esophageal squamous-cell carcinoma. N. Engl. J. Med..

[4] Luo H (2021). Effect of camrelizumab vs placebo added to chemotherapy on survival and progression-free survival in patients with advanced or metastatic esophageal squamous cell carcinoma: the ESCORT-1st randomized clinical trial. JAMA.

[5] Wang ZX (2022). Toripalimab plus chemotherapy in treatment-naive, advanced esophageal squamous cell carcinoma (JUPITER-06): a multi-center phase 3 trial. Cancer Cell.

[6] Lu Z (2022). Sintilimab versus placebo in combination with chemotherapy as first line treatment for locally advanced or metastatic oesophageal squamous cell carcinoma (ORIENT-15): multicentre, randomised, double blind, phase 3 trial. Brit. Med. J..

[7] Katsumata N (2009). Dose-dense paclitaxel once a week in combination with carboplatin every 3 weeks for advanced ovarian cancer: a phase 3, open-label, randomised controlled trial. Lancet.

[8] Del Mastro L (2015). Fluorouracil and dose-dense chemotherapy in adjuvant treatment of patients with early-stage breast cancer: an open-label, 2 × 2 factorial, randomised phase 3 trial. Lancet.

[9] de Gramont A (1997). Randomized trial comparing monthly low-dose leucovorin and fluorouracil bolus with bimonthly high-dose leucovorin and fluorouracil bolus plus continuous infusion for advanced colorectal cancer: a French intergroup study. J. Clin. Oncol..

[10] Ohigashi Y (2005). Clinical significance of programmed death-1 ligand-1 and programmed death-1 ligand-2 expression in human esophageal cancer. Clin. Cancer Res..

[11] Kato K (2019). Nivolumab versus chemotherapy in patients with advanced oesophageal squamous cell carcinoma refractory or intolerant to previous chemotherapy (ATTRACTION-3): a multicentre, randomised, open-label, phase 3 trial. Lancet Oncol..

[12] Huang J (2020). Camrelizumab versus investigator’s choice of chemotherapy as second-line therapy for advanced or metastatic oesophageal squamous cell carcinoma (ESCORT): a multicentre, randomised, open-label, phase 3 study. Lancet Oncol..

[13] Kojima T (2020). Randomized phase III KEYNOTE-181 study of pembrolizumab versus chemotherapy in advanced esophageal cancer. J. Clin. Oncol..

[14] KEYTRUDA^®^ (pembrolizumab) [US Food and Drug Administration prescribing information]. https://www.accessdata.fda.gov/drugsatfda_docs/label/2022/125514s123lbl.pdf (Merck Sharp & Dohme Corp., 2022).

[15] *NCCN Clinical Practice Guidelines in Oncology (NCCN Guidelines*^*®*^*). Esophageal and Esophagogastric Junction Cancers.* Version 5.2022. https://www.nccn.org/guidelines/guidelines-detail?category=1&id=1433 (National Comprehensive Cancer Network, 2022).

[16] KEYTRUDA^®^ (pembrolizumab) [European Medicines Agency summary of product characteristics]. https://www.ema.europa.eu/en/documents/product-information/keytruda-epar-product-information_en.pdf (Merck Sharp & Dohme Corp., 2022).

[17] Issafras H (2021). Structural basis of HLX10 PD-1 receptor recognition, a promising anti-PD-1 antibody clinical candidate for cancer immunotherapy. PLoS One.

[18] Qin S (2021). Efficacy and safety of HLX10, a novel anti-PD-1 antibody, in patients with previously treated unresectable or metastatic microsatellite instability-high or mismatch repair-deficient solid tumors: a single-arm, multicenter, phase 2 study. J. Clin. Oncol..

[19] Wu L (2021). Efficacy and safety evaluation of HLX10 (a recombinant humanized anti-PD-1 monoclonal antibody) combined with albumin-bound paclitaxel in patients with advanced cervical cancer who have progressive disease or intolerable toxicity after first-line standard chemotherapy: a single-arm, open-label, phase 2 study. J. Clin. Oncol..

[20] Cheng Y (2022). Effect of first-Line serplulimab vs placebo added to chemotherapy on survival in patients with extensive-stage small cell lung cancer: the ASTRUM-005 randomized clinical trial. JAMA.

[21] *The NDA of Henlius novel anti-PD-1 mAb serplulimab for first-line treatment of sqNSCLC accepted by China’s NMPA, phase 3 MRCT met its primary endpoint*. https://www.henlius.com/en/NewsDetails-3200-26.html (Shanghai Henlius Biotech, Inc., 2021).

[22] Li Z (2021). First-line pembrolizumab plus chemotherapy versus chemotherapy in patients with advanced esophageal cancer: Chinese subgroup analysis of KEYNOTE-590. J. Clin. Oncol..

[23] Iizuka T (1992). Phase II evaluation of cisplatin and 5-fluorouracil in advanced squamous cell carcinoma of the esophagus: a Japanese Esophageal Oncology Group trial. Jpn J. Clin. Oncol..

[24] Bleiberg H (1997). Randomised phase II study of cisplatin and 5-fluorouracil (5-FU) versus cisplatin alone in advanced squamous cell oesophageal cancer. Eur. J. Cancer.

[25] Leone AG (2022). Efficacy and activity of PD-1 blockade in patients with advanced esophageal squamous cell carcinoma: a systematic review and meta-analysis with focus on the value of PD-L1 combined positive score. ESMO Open.

[26] Lin L, Lin D-C (2019). Biological significance of tumor heterogeneity in esophageal squamous cell carcinoma. Cancers.

[27] Seymour L (2017). iRECIST: guidelines for response criteria for use in trials testing immunotherapeutics. Lancet Oncol..

