# Supplementary material for: First-line serplulimab or placebo plus chemotherapy in PD-L1-positive esophageal squamous cell carcinoma: a randomized, double-blind phase 3 trial
Source: Nat Med. 2023 Feb 2;29(2):473–82. doi: 10.1038/s41591-022-02179-2 (PMC9941045; doi:10.1038/s41591-022-02179-2)
Supplement: Supplementary file 1 — Supplementary Table 1, study protocol and statistical analysis plan. [file 41591_2022_2179_MOESM1_ESM.pdf]

nature medicine

Article

https://doi.org/10.1038/s41591-022-02179-2

First-line serplulimab or placebo plus chemotherapy in PD-L1-positive esophageal squamous cell carcinoma: a randomized, double-blind phase 3 trial

In the format provided by the authors and unedited

Supplementary information

Supplementary online content for Yan Song, Bo Zhang, Dao Xin, et al. for

First-line serplulimab versus placebo in combination with chemotherapy in PD-L1–positive esophageal squamous cell carcinoma: a randomized, double-blind phase 3 trial

Table of Contents

Supplementary Table 1: ASTRUM-007 investigators....................2

ASTRUM-007 study protocol and statistical analysis plan....................4

Supplementary Table 1: ASTRUM-007 investigators

Investigator	Site name
Jing Huang	National Cancer Center/National Clinical Research Center for Cancer/Cancer Hospital, Chinese Academy of Medical Sciences and Peking Union Medical College
	National Cancer Center/National Clinical Research Center for Cancer/Cancer Hospital & Shenzhen Hospital
Xiaoge Kou	The First Affiliated Hospital of Xinxiang Medical University
Zhenbo Tan	Xingtai People's Hospital
Shu Zhang	Shandong First Medical University Cancer Hospital, Shandong Cancer Hospital
Meili Sun	Central Hospital Affiliated to Shandong First Medical University
Jin Zhou	Sichuan Cancer Hospital
Min Fan	Fudan University Shanghai Cancer Center
Ming Zhang	Shanghai Chest Hospital, Shanghai Jiao Tong University
Yongxiang Song	Affiliated Hospital of Zunyi Medical University
Suyi Li	Anhui Provincial Cancer Hospital
Yuan Yuan	Xuzhou Central Hospital
Wu Zhuang	Fujian Cancer Hospital
Jingdong Zhang	Liaoning Cancer Hospital & Institute, Cancer Hospital of China Medical University
Li Zhang	Chongqing University Three Gorges Hospital
Hao Jiang	The First Affiliated Hospital of Bengbu Medical College
Kangsheng Gu	The First Affiliated Hospital of Anhui Medical University
Huangyang Ye	The First Affiliated Hospital of Xiamen University
Weiyong Yu	Huai'an First People's Hospital
Weixin Wu	Zhongshan Hospital Affiliated to Xiamen University
Fuming Qiu	The Second Affiliated Hospital Zhejiang University School of Medicine
Mudan Yang	Shanxi Provincial Cancer Hospital
Yanqiao Zhang	The Affiliated Tumour Hospital of Harbin Medical University
Ruixing Zhang	The Fourth Hospital of Hebei Medical University and Hebei Tumor Hospital
Wei Li	The First Hospital of Jilin University
Junxing Huang	Jiangsu Taizhou People's Hospital
Yan Yang	Gansu Provincial Cancer Hospital
Qing Bi	Yunnan Cancer Hospital
Tao Wu	Anyang Cancer Hospital
Chunmei Bai	Chinese Academy of Medical Sciences & Peking Union Medical College
Yunpeng Liu	The First Hospital of China Medical University
Qingxia Fan	The First Affiliated Hospital of Zhengzhou University
Wenying Deng & Ying Liu	Henan Cancer Hospital & The Affiliated Cancer Hospital of Zhengzhou University
Xicheng Wang	The First Affiliated Hospital of Guangdong Pharmaceutical University
Linzhi Lu	Gansu Wuwei Tumour Hospital
Peng Tang	Tianjin Medical University Cancer Institute & Hospital
Yiping Zhu	The First Affiliated Hospital of Wannan Medical College
Jianhua Chen	Hunan Cancer Hospital
Junhe Li	The First Affiliated Hospital of Nanchang University
Yanhong Gu	Jiangsu Province Hospital
Tao Jiang	Tangdu Hospital of Air Force Medical University of PLA

**Investigator**	**Site name**
Qi Wang	The First People's Hospital of Changzhou
Baoping Chang	The First Affiliated Hospital of Henan University of Science and Technology
Weijia Fang	The First Affiliated Hospital Zhejiang University School of Medicine
Zhixiang Zhuang	The Second Affiliated Hospital of Soochow University
Yueyin Pan	Anhui Provincial Hospital
Tianshu Liu	Zhongshan Hospital, Fudan University.
Jifeng Feng	Jiangsu Cancer Hospital
Ying Cheng	Jilin Cancer Hospital
Rong Wu	Shengjing Hospital of China Medical University
Zhongmian Zhang	The Second Affiliated Hospital of Zhengzhou University
Fen Wang	Peking University Shenzhen Hospital
Ke Jiang	Union Hospital, Tongji Medical College, Huazhong University of Science and Technology
Huiting Xu	Hubei Cancer Hospital
Shan Zeng	Xiangya Hospital Central South University
Yong Liu	Chongqing Traditional Chinese Medicine Hospital
Yong Xin	The Affiliated Hospital of Xuzhou Medical University
Guohua Yu	Weifang People's Hospital
Dong Ma	Guangdong Provincial People's Hospital
Shundong Cang	Henan Provincial People's Hospital
Chuan Jin	Affiliated Cancer Hospital and Institute of Guangzhou Medical University
Xinmin Yu	Zhejiang Cancer Hospital
Peiguo Cao	The Third Xiangya Hospital of Central South University
Longqi Chen	West China Hospital of Sichuan University
Enxiao Li	The First Affiliated Hospital of Xi'an Jiaotong University

ASTRUM-007 study protocol and statistical analysis plan

A Randomized, Double-Blind, Multicentre, Phase 3 Clinical Study to Compare HLX10 (Recombinant Humanized Anti-PD-1 Monoclonal Antibody Injection) versus Placebo in Combination with Chemotherapy (Cisplatin + 5-FU) as First-Line Therapy in Patients with Locally Advanced/Metastatic Oesophageal Squamous Cell Carcinoma (ESCC)

Protocol

**Study Drug**	HLX10
**NMPA Approval No.**	2018L02201
**Protocol No.**	HLX10-007-EC301
**Sponsor**	Shanghai Henlius Biotech, Inc.
**Coordinating Investigator**	[redacted]
**Version No.**	4.0
**Version Date**	26 Jun., 2021

Confidentiality Statement

All information in this protocol is the proprietary property of Shanghai Henlius Biotech, Inc, and it is not allowed to be forwarded or copied by any unauthorized individuals without prior written authorization, except the investigators/Ethics Committee/relevant government departments and the sponsor's authorized personnel.

SIGNATURE PAGE

I have read and been familiar with the protocol, confirmed the study plan and such necessary content related to study implementation as included in the protocol, and clarified the responsibilities of the investigators related to the protocol. I agree and will perform relevant responsibilities in strict accordance with Chinese laws and regulations, the Declaration of Helsinki, Good Clinical practices, and this protocol.

Coordinating Investigator: [redacted]

Signature Date

Institution: [redacted]

SIGNATURE PAGE

I have read and been familiar with the protocol, confirmed the study plan and such necessary content related to study implementation as included in the protocol, and clarified the responsibilities of the investigators related to the protocol. I agree and will perform relevant responsibilities in strict accordance with Chinese laws and regulations, the Declaration of Helsinki, Good Clinical practices, and this protocol.

Principal Investigator

Signature Date

Institution

SIGNATURE PAGE

I have read and been familiar with the protocol, confirmed the study plan and such necessary content related to study implementation as included in the protocol, and clarified the responsibilities of the investigators related to the protocol. I agree and will perform relevant responsibilities in strict accordance with Chinese laws and regulations, the Declaration of Helsinki, Good Clinical practices, and this protocol.

[redacted] Director of Medicine
Shanghai Henlius Biotech, Inc.

Date

[redacted], Director of Biostatistics
Shanghai Henlius Biotech, Inc.

Date

TABLE OF CONTENTS

ABBREVIATIONS....10
STUDY SYNOPSIS.... 14
1 INTRODUCTION.... 29
1.1 Study Background....29
1.2 Rationale for Conduct of Study and Dose Selection....31
1.2.1 Nonclinical overview of HLX10.... 31
1.2.2 Clinical studies of HLX10....36
1.2.3 Rationale for selection of chemotherapy as control....36
1.2.4 Rationale for HLX-10 and chemotherapy combination therapy.... 36
1.2.5 Rationale for selection of HLX10 dose.... 37
1.3 Benefit-Risk Assessment....37
1.3.1 Potential benefits....37
1.3.2 Identified and potential risks....38
1.3.3 Overall benefits: risk and ethical assessments....38
2 STUDY OBJECTIVES....39
2.1 Primary Objective....39
2.2 Secondary Objective....39
3 STUDY PLAN....39
3.1 Overall Study Design.... 39
3.1.1 Study type and duration.... 39
3.1.2 Randomisation and blinding.... 39
3.2 Study Endpoints.... 40
3.2.1 Primary endpoint....40
3.2.2 Secondary endpoints....41
3.3 Number of Subjects....41
3.4 Subject Eligibility....41
3.4.1 Inclusion criteria.... 41
3.4.2 Exclusion criteria.... 43
3.4.3 Withdrawal criteria.... 46

3.5 Early Study Termination/Site Closeout....47
4 STUDY TREATMENT....48
4.1 Study Drugs....48
4.2 Administration Method and Dosage....48
4.3 Principles of Administration and Dosage Adjustment....49
4.3.1 General principles....49
4.3.2 Principles for HLX10/placebo medication....50
4.3.3 Dose adjustment principle of chemotherapeutics (the following dose adjustment is provided to the investigator for reference)....50
4.4 HLX10-Related Toxicity and Management....51
4.4.1 Cutaneous adverse events....52
4.4.2 Endocrine disorders....52
4.4.3 Pneumonia....53
4.4.4 Colitis....54
4.4.5 Nephrotoxicity....55
4.4.6 Hepatotoxicity....56
4.4.7 Neuropathy....56
4.4.8 Other immune-related adverse events....57
4.5 Infusion-Related Reactions and Management....57
4.6 Drug Package and Label....59
4.7 Drug Storage, Management and Dispensation....59
4.8 Concomitant and Prohibited Treatments....59
4.9 Treatment Compliance....61
5 STUDY PROCEDURE AND VISITS....61
5.1 Study Procedure....61
5.1.1 Demographics and medical history....61
5.1.2 Prior and concomitant therapy....62
5.1.3 Adverse events....62
5.1.5 Echocardiography....63
5.1.6 12-Lead ECG....63
5.1.7 Complete physical examination....64

5.1.8 Symptom-directed physical examination....64
5.1.9 Height, weight, and vital signs....64
5.1.10 ECOG score....64
5.1.11 Survival status....65
5.1.12 Local laboratory tests....65
5.1.13 Central laboratory assessments....66
5.1.14 Radiological assessment of the tumor....67
5.1.15 Sample collection for biomarkers....68
5.2 Screening (Day –28 to Day –1)....68
5.3 Treatment Period....70
5.4 End-of-Treatment Visit....72
5.5 Follow-Up Period....73
5.5.1 Safety follow-up....73
5.5.2 Survival follow-up....74
6 STUDY ASSESSMENT....75
6.1 Efficacy Assessment....75
6.2 Safety Assessment....75
7 ADVERSE EVENT....75
7.1 Definition of Adverse Event....75
7.2 Adverse Event of Special Interest (AESI)....76
7.3 Definition of Serious Adverse Event....77
7.4 Documentation of Adverse Events....79
7.4.1 Time of gathering adverse event information....79
7.4.2 Follow-up of unresolved adverse events....80
7.4.3 Information collection....80
7.4.4 Severity evaluation....81
7.4.5 Causality assessment....82
7.4.6 Expectedness assessment....83
7.4.7 Progressive disease....83
7.4.8 Newly developed primary tumor....84
7.4.9 Death....84

7.4.10 Drug-induced liver injury (DILI)....84
7.5 Reporting of AEs....85
7.5.1 Reporting requirements for serious adverse events....85
7.6 Overdose....86
7.7 Pregnancy....86
8 STATISTICAL METHODS....87
8.1 Sample Size Estimation....87
8.2 Statistical Analysis Sets....88
8.2.1 Intent-to-treat (ITT) set....88
8.2.2 Per protocol set (PPS)....88
8.2.3 Safety set (SS)....88
8.2.4 Pharmacokinetics set (PKS)....88
8.3 Interim Analysis and Final Analysis....88
8.4 Statistical Analysis Methods....89
8.4.1 Demographics, medical history, and baseline characteristics....89
8.4.2 Medication compliance....89
8.4.3 Efficacy analysis....89
8.4.4 Safety analysis....92
8.4.5 Pharmacokinetics and immunogenicity analysis....92
8.4.6 Biomarker analysis....92
8.4.7 Analysis of patient-reported outcomes....93
9 ETHICS....94
9.1 Ethic Requirements....94
9.2 Informed Consent....94
9.3 Confidentiality of Subjects....95
10 DATA MANAGEMENT....96
10.1 Completion of Raw Data and Electronic Case Report Form (eCRF)....96
10.2 Design and Establishment of the Database....96
10.3 Data Coding....96
10.4 Data Verification....97
10.5 Database Lock....97

10.6 Blind Verification and Unblinding....97
11 STUDY MANAGEMENT....98
11.1 Quality Control and Quality Assurance.... 98
11.1.1 Training....98
11.1.2 Clinical monitoring....98
11.1.3 Audit.... 99
11.1.4 Data management/coding.... 99
11.1.5 Missing and useless data....100
11.2 Documentation and Preservation of Study Data.... 100
11.3 Follow-up and Medical Measures after the End of the Study....100
12 ALL PARTIES' RESPONSIBILITIES.... 100
12.1 Investigator's Responsibilities....100
12.2 Sponsor's Responsibilities....102
13 CONFIDENTIALITY AND PUBLICATION OF STUDY RESULTS....103
14 REFERENCES....104
15 APPENDICES....106
Appendix 1: Common Terminology Criteria for Adverse Events....106
Appendix 2: Response Evaluation Criteria in Solid Tumors (RECIST 1.1).... 107
Appendix 3: Quality of Life Questionnaires EORTC QLQ-C30, EQ-5D-5L and EORTC QLQ-OES18.... 113
Appendix 4: Eastern Cooperative Oncology Group (ECOG) - Performance Status....119
Appendix 5: Fridericia's Correction Formula.... 120
Appendix 6: Cockcroft and Gault Formula.... 120
Appendix 7: New York Heart Association Classification.... 121

ABBREVIATIONS

Abbreviation	Definition
12-ECG	12-lead ECG
ADA	Anti-drug antibody
ADL	Activities of daily living
AE	Adverse event
AESI	Adverse event of special interest
ALP	Alkaline phosphatase
ALT	Alanine aminotransferase
ANC	Absolute neutrophil count
APTT	Activated partial thromboplastin time
ASCO	American Society of Clinical Oncology
AST	Aspartate aminotransferase
ATC	Anatomical therapeutic chemical classification system
BMI	Body mass index
BNP	Brain natriuretic peptide
BUN	Blood urea nitrogen
CHO	Chinese hamster ovary cells
CI	Confidence interval
CL	Clearance
CMH	Cochran-Mantel-Haenszel test
CNS	Central nervous system
CPS	Combined positive score
CR	Complete response
Cr	Creatinine
CRA	Clinical research associate
CSF	Colony-stimulating factor
CT	Computed tomography
CTCAE	Common Terminology Criteria for Adverse Events
CTLA4	Cytotoxic T lymphocyte-associated antigen-4
DCR	Disease control rate
DOR	Duration of response
EAC	Oesophageal adenocarcinoma
ECOG	Eastern Cooperative Oncology Group

Abbreviation	Definition
eCRF	Electronic case report form
EDC	Electronic data capture
EORTC QLQ-C30	European Organization for Research and Treatment of Cancer Quality of Life Questionnaire-C30
EORTC QLQ-OES18	European Organization for Research and Treatment of Cancer Quality of Life Questionnaire - Oesophageal Cancer Module
EQ-5D-5L	European Quality of Life Five-Dimension Five-Level Scale
ESCC	Oesophageal squamous cell carcinoma
FAS	Full analysis set
FDA	Food and Drug Administration
FDG-PET	Fluorodeoxyglucose positron emission tomography
FFPE	Formalin-fixed paraffin-embedded
FT3	Free triiodothyronine
FT4	Free thyroxine
GCP	Good Clinical Practice
Hb	Haemoglobin
HBcAb	Hepatitis B core antibody
HBsAg	Hepatitis B surface antigen
HBV	Hepatitis B virus
HCV	Hepatitis C virus
HIV	Human immunodeficiency virus
HR	Hazard ratio
HRQoL	Health-related quality of life
ICF	Informed consent form
ICH	International Conference on Harmonization
IDMC	Independent Data Monitoring Committee
IEC	Independent Ethics Committee
ILD	Interstitial lung disease
INR	International normalized ratio
irAE	Immune-related adverse event
IRB	Institutional Review Board
IRR	Infusion-related reaction
IRRC	Independent Radiological Review Committee
ITT	Intent-to-treat

Abbreviation	Definition
IVIG	Intravenous immunoglobulin
LFT	Liver function test
LVEF	Left ventricular ejection fraction
MRI	Magnetic resonance imaging
MSI	Microsatellite instability
NA	Not applicable
NCCN	National comprehensive cancer network
NE	Not evaluable
NK cell	Natural killer cell
NMPA	National Medical Products Administration
NT-proBNP	N-terminal pro brain natriuretic peptide
NYHA	New York Heart Association
ORR	Objective response rate
OS	Overall survival
PCP	Pneumocystis pneumonia
PD	Progressive disease
PD-1	Programmed cell death protein 1
PD-L1	Programmed cell death-ligand 1
PET-CT	Positron emission tomography/computed tomography
PFS	Progression-free survival
PI	Principal investigator
PK	Pharmacokinetics
PKS	Pharmacokinetic set
PLT	Platelet
PPS	Per protocol set
PR	Partial response
PS	Performance status
PT	Preferred term
PT	Prothrombin time
PV	Pharmacovigilance
RECIST	Response Evaluation Criteria in Solid Tumors
RO	Receptor occupancy
SAE	Serious adverse event
SAP	Statistical analysis plan

Abbreviation	Definition
SD	Stable disease
SOC	System Organ Class
SOP	Standard Operating Procedure
SS	Safety set
T3	Triiodothyronine
T4	Thyroxine
TB	Total bilirubin
TEAE	Treatment emergent adverse event
TMB	Tumor mutation burden
TNF-α	Tumor necrosis factor α
TSH	Thyroid-stimulating hormone
ULN	Upper limit of normal
US	Ultrasound
WHO-DDE	World Health Organisation Drug Dictionary Enhanced

STUDY SYNOPSIS

**Version no.: V4.0**<br>**Version date: 26 Jun., 2021**	**Protocol no.: HLX10-007-EC301**

Investigational product: HLX10 (recombinant humanised anti-PD-1 monoclonal antibody injection)

Study Title:

A Randomized, Double-Blind, Multicentre, Phase 3 Clinical Study to Compare HLX10 (Recombinant Humanized Anti-PD-1 Monoclonal Antibody Injection) versus Placebo in Combination with Chemotherapy (Cisplatin + 5-FU) as First-Line Therapy in Patients with Locally Advanced/Metastatic Oesophageal Squamous Cell Carcinoma (ESCC)

Number of study sites: multiple

Study Phase: III

Study Objectives:

Primary objective:

- To compare the clinical efficacy of HLX10 versus placebo in combination with chemotherapy as first-line therapy in patients with locally advanced/metastatic ESCC.

Secondary objective:

- To compare the safety and tolerability of HLX10 versus placebo in combination with chemotherapy as first-line therapy in patients with locally advanced/metastatic ESCC.

Study Endpoints:

Primary endpoint:

- PFS (assessed by the IRRC as per RECIST v1.1)

Secondary endpoints:

- Overall survival (OS)
- Progression-free survival (PFS) (assessed by the IRRC as per iRECIST, and assessed by the investigator as per RECIST v1.1)
- Objective response rate (ORR) (assessed by IRRC and the investigator as per RECIST v1.1)
- Relationship between PD-L1 expression in tumor tissue and efficacy
- Duration of response (DOR) (assessed as per RECIST v1.1 and iRECIST)
- Incidence of adverse events (AEs) and serious adverse events (SAEs)
- Pharmacokinetics (PK): Concentration of HLX10 in serum
- Immunogenicity evaluation: Positive rate of anti-drug antibody (ADA)

- Relationship between microsatellite instability (MSI) and tumor mutation burden (TMB) and efficacy
- Quality of life assessment

Study Design and Methods:

This study is a randomized, double-blind, multicentre, phase 3 clinical study to compare the clinical efficacy and safety of HLX10 versus placebo in combination with chemotherapy as first-line therapy in patients with locally advanced/metastatic ESCC.

Eligible subjects in this study will be randomized in a 2:1 ratio to two groups as follows:

- Group A (HLX10): HLX10 + chemotherapy (cisplatin + 5-FU)
- Group B (control): placebo + chemotherapy (cisplatin + 5-FU)

Randomization is stratified by: PD-L1 expression level (CPS < 10 versus CPS ≥ 10), age (≥ 65 years versus < 65 years), and tumor state (locally advanced versus distant metastasis).

After screening, subjects meeting the inclusion criteria and not meeting the exclusion criteria will be enrolled. Included subjects will receive the study drug until loss of clinical benefits, death, unacceptable toxicity, withdrawal of informed consent, or other reasons specified in the protocol, whichever occurs first.

During the study, **if a subject decides to continue with treatment after first progressive disease (as per RECISTv1.1), the subject must sign the informed consent form (ICF) again and must meet the following criteria:**

- Absence of clinical symptoms and signs of significant disease progression (including worsening laboratory results).
- Stable Eastern Cooperative Oncology Group (ECOG) performance status (PS).
- Absence of rapid progression of disease or of tumor progression at critical anatomical sites (e.g., spinal cord compression) that necessitates urgent alternative medical intervention.
- Satisfaction of corresponding laboratory test parameters in inclusion criteria of this study (i.e., meet the inclusion criterion 8).

The subject will enter the next treatment period after progressive disease (first PD) (RECIST v1.1), as follows:

- **Group A**: The investigator will comprehensively judge whether the subject is suitable for continuing with the original treatment regimen according to the actual situation of the subject; if suitable, the subject can choose to continue with the original treatment regimen after signing the informed consent form again, and may receive another radiological examination at an interval of 4–8 weeks. If PD (iRECIST) is confirmed through subsequent radiological examination, the subject will be off to the follow-up period.
- **Group B**: The investigator will comprehensively judge whether the subject is suitable for continuing with the original treatment regimen according to the actual situation of the subject; if suitable, the subject can choose to continue with the original treatment regimen after signing the informed consent form again, and may receive another radiological examination at an interval of 4–8 weeks. If PD (iRECIST) is confirmed through subsequent radiological examination, the subject will be off to the follow-up period.

The primary endpoint of this study is to compare the OS and PFS between two groups, and for the PFS, it will be assessed by the IRRC per RECIST v1.1.

Study Duration:

This study includes three periods: Screening period (28 days), treatment period (until loss of clinical benefits, death, unacceptable toxicity, withdrawal of informed consent, or other reasons specified in the protocol, whichever occurs first), and follow-up period (including safety follow-up period and survival follow-up period).

Number of Subjects: About 540 subjects (360 subjects in HLX10 group, and 180 subjects in placebo group)

Main Inclusion/Exclusion Criteria:

Inclusion Criteria:

1. 18–75 years, male and female;
2. Histologically diagnosed with locally-advanced (determined by local investigator)/recurrent or distantly metastatic ESCC (including gastro-oesophageal junction) that is not resectable or cured by chemoradiotherapy (patient with adenosquamous carcinoma with predominantly squamous cell carcinoma can be enrolled);
3. Have not received any systemic anti-tumor therapy for current recurrence or metastasis. Exceptions: A patient who has received neoadjuvant/adjuvant therapy can be screened if his/her last neoadjuvant/adjuvant treatment is more than 6 months from relapse or PD; a patient who has received curative concurrent chemoradiotherapy or radiotherapy for oesophagus cancer can be screened if his/her last chemotherapy/radiotherapy is more than 6 months from relapse or PD. (Note: For radical concurrent chemoradiotherapy and neoadjuvant/adjuvant therapy (chemotherapy or chemoradiotherapy), any disease progression during treatment or within 6 months after discontinuation should be taken as a failure of first-line treatment, while any disease progression exceeding 6 months after discontinuation should not be taken as a failure of first-line treatment.)
4. At least one measurable lesion as assessed based on central imaging per Response Evaluation Criteria in Solid Tumors (RECIST v1.1) (cavity structures such as oesophagus cannot be considered as measurable lesions). The measurable lesion should not have received local treatment such as radiotherapy (if progression of a lesion located in prior radiotherapy region is confirmed, it can also be selected as a target lesion);
5. Subjects whose tumor samples are PD-L1 positive (CPS ≥ 1). The subjects must provide tumor tissue to measure the PD-L1 expression level;
6. ECOG within 7 days prior to the first dose of study drug: 0–1;
7. Expected survival ≥ 12 weeks;
8. The functions of vital organs meet the following requirements (blood transfusion or use of any cell growth factors and/or thrombopoietic agents is not permitted within 14 days prior to the first dose of the study drug);
    a. Absolute neutrophil count (ANC) ≥ 1.5 × 10^9/L
    b. Platelet count ≥ 100 × 10^9/L;
    c. Haemoglobin ≥ 9 g/dL;
    d. Serum albumin ≥ 3.0 g/dL;
    e. Total bilirubin ≤1.5 × ULN, ALT, AST and/or ALP ≤ 2.5 × ULN; if liver metastasis is present, ALT and/or AST ≤ 5 × ULN; if liver metastasis or bone metastasis is present, ALP ≤ 5 × ULN;

   f. Serum creatinine ≤ 1.5 × ULN or creatinine clearance ≥ 60 mL/min (calculated using Cockcroft-Gault formula);
   g. Activated partial thromboplastin time (APTT), international normalized ratio (INR) and prothrombin time (PT) ≤ 1.5 × ULN;

9. Female subjects of childbearing potential must have a negative blood pregnancy test within 7 days before the first dose. Female subjects of childbearing potential and male subjects having female partners in child-bearing period require at least one medically approved contraceptive method (such as intrauterine device, contraceptive drug, or condom) during the study, for at least 3 months after last dose of HLX10/placebo and for at least 6 months after last chemotherapy;
10. Subjects are willing to participate in this study and sign informed consent form, have good compliance and cooperate with the follow-up.

Exclusion Criteria:

1. BMI < 16.0 kg/m^2;
2. History of gastrointestinal perforation and/or fistulae within 6 months prior to the first dose of study drug;
3. Obvious invasion of tumor into organs (main artery or trachea) adjacent to oesophageal lesion, causing higher risk of bleeding or fistulae; history of stent implantation in the tracheal lumen;
4. Uncontrollable pleural effusion, pericardial effusion or ascites requiring repeated drainage;
5. Prior allergic history to monoclonal antibodies, any ingredient of HLX10, 5-FU, cisplatin or other platinum drugs;
6. Have received any of the following treatment:
   a. Have received prior anti-PD-1 or anti-PD-L1 antibody treatment;
   b. Have received any investigational drug within 4 weeks prior to the first dose of study drug;
   c. Been enrolled in another clinical study at the same time, unless it is an observational (non-interventional) clinical study or in the interventional clinical study follow-up period;
   d. Have received last cycle of anticancer treatment within ≤ 4 weeks prior to the first dose of study drug; palliative radiotherapy for bone metastasis lesion is allowed and should be finished 2 weeks prior to the first dose. Radiotherapy covering more than 30% of bone marrow area within 28 days prior to the first dose is not allowed;
   e. Subjects who require to receive systemic treatment with corticosteroids (> 10 mg/day prednisone equivalent) or other immunosuppressive agents within 14 days prior to the first dose of study drug; inhaled or topical corticosteroids, and replacement with adrenocortical hormone at a dose ≤ 10 mg/day prednisone equivalent are permitted in the absence of active autoimmune disease;
   f. Have received anti-tumor vaccine or have received live vaccine within 4 weeks prior to the first dose of study drug;
   g. Have received major surgery within 28 days prior to the first dose of study drug; major surgery in this study is defined as: any surgery which requires at least 3 weeks of postoperative recovery time before receiving the study treatment. Patients with a history of tumor needle biopsy or lymph node incisional biopsy are included;
7. Toxicity from prior anti-tumor treatment not recovered to ≤ CTCAE Grade 1 (except alopecia) or the level specified in the inclusion criteria;

8. Patients with metastases to the central nervous system;
9. Active autoimmune diseases or history of autoimmune diseases (such as interstitial pneumonia, colitis, hepatitis, hypophysitis, vasculitis, nephritis, hyperthyroidism and hypothyroidism, including but not limited to these diseases or syndromes); except patients with leucoderma or cured childhood asthma/allergy and not requiring any intervention in adulthood; autoimmune mediated hypothyroidism treated with stable-dose thyroid hormone replacement therapy; type I diabetes treated with a stable dose of insulin; subjects who are in a stable state and do not require systemic immunosuppressive therapy (including corticosteroids)；
10. History of immune deficiency, including HIV antibody test positive, or history of other acquired or congenital immune deficiencies, or history of organ transplantation and allogeneic bone marrow transplantation;
11. Subjects have uncontrolled clinical heart and cardiovascular symptoms or diseases, including but not limited to: For example: (1) Heart failure above NYHA grade II; (2) unstable angina pectoris; (3)prior myocardial infarction and cerebral infarction within 6 months; (4) clinically significant supraventricular or ventricular arrhythmia without clinical intervention or inadequately controlled after clinical intervention;
12. Severe infection (CTCAE > grade 2) within 4 weeks prior to the first dose of study drug, such as severe pneumonia, bacteraemia, and infection complications requiring hospitalisation; active pulmonary inflammation accompanied with relevant clinical symptoms or signs based on chest X-ray at baseline; symptoms and signs of infection requiring oral or intravenous antibiotic therapy within 2 weeks prior to the first dose of study drug, except prophylactic use of antibiotics;
13. Subjects with prior and current interstitial pneumonia, pneumoconiosis, drug-related pneumonia, severely impaired lung function, etc. which may disturb the detection and treatment of suspected drug-related pulmonary toxicity; subjects with radiation pneumonia within 6 months;
14. Patients with active tuberculosis infection based on medical history or CT test, or patients with a history of active tuberculosis infection within 1 year before enrolment, or patients with active tuberculosis infection beyond 1 year but without proper treatment;
15. With HBsAg (+) and/or HBcAb (+), and HBV-DNA ≥ 500 IU/mL or 2500 copies/mL at the time of enrolment. Any subject with a measurement above the said criteria can only be enrolled if the measurement decreases to the normal range for at least 2 weeks after antiviral treatment, and he/she continues to receive the antiviral treatment throughout the study. Any subject who has previously needed to receive or were receiving antiviral treatment at the time of screening can only be enrolled if he/she continues to receive antiviral treatment throughout the study, even if HBV-DNA meets the inclusion criteria. Hepatitis C (HCV antibody tested positive and HCV-RNA positive);
16. Patients with any other malignancies diagnosed within 5 years prior to the first dose of study drug, except malignancies having low risk of metastasis and death (5-year survival rate > 90%), such as adequately treated basal cell or squamous cell skin cancer, or carcinoma in situ of cervix uteri, and other carcinoma in situ;
17. Pregnant or lactating women;
18. Subjects having other factors possibly causing study discontinuation, as assessed by the investigator, such as other severe diseases (including metal disease) requiring concomitant treatment, seriously abnormal laboratory test values, family or social factors, and other conditions possibly affecting the safety or study data collection of the subjects.

Study Treatment:

Investigational Products, Dosage, and Mode of Administration:

The study drugs will be administered as follows: Every 2 weeks (14 days) equals to one cycle in the following regimen.

Sequence of the combination treatment: HLX10/placebo, cisplatin, and 5-fluorouraeil (5-FU) will be administered successively on day 1.

Study drug: HLX10 or placebo

3 mg/kg, intravenous infusion (iv), before infusion, it is recommended to filter with 0.2~5 μm in-tube filter, every 2 weeks (14 days) (Q2W), on Day 1 of each cycle, no reduction, and for up to 2 years.

Other study drugs: Combination chemotherapy

- Cisplatin: 50 mg/m^2, intravenous (iv) infusion, Q2W, on Day 1 of each cycle, and dose for less than or equal to 8 cycles.
- 5-fluorouraeil (5-FU): at a total dose of 2400 mg/m^2, continuous iv drip over 44–48 hours in each cycle, Q2W, and for up to 12 cycles.

Treatment with study drugs will continue until loss of clinical benefit, PD, intolerable toxicity, discontinuation decided by the subject or physician, death, withdrawal of informed consent, pregnancy, non-compliance with protocol or procedure requirements, administrative reasons, or other reasons specified in the protocol (whichever occurs first). After 8 cycles of cisplatin and 12 cycles of 5-FU, the subject should not continue to use them even if the subject did not experience the above conditions. HLX10/placebo can be administered for up to 2 years.

Study Assessments:

Efficacy:

After random, assessments will be conducted every 6 weeks (± 7 days) in the first 48 weeks and every 12 weeks (± 7 days) afterwards. The investigator and IRRC will separately assess the radiological conditions of tumor per RECIST v1.1 based on the test results of computed tomography (CT) or magnetic resonance imaging (MRI) (neck, chest, abdomen, pelvis, and any other sites suspected of tumor lesion), and the frequency of tumor assessments can be increased by the investigator as clinically indicated. For subjects who continue with treatment after first PD, radiological assessment should be conducted again after an interval of 4–8 weeks. Subjects who discontinue for reasons other than PD should continue the radiological assessments per schedule until PD, initiation of new anti-tumor therapy, withdrawal of informed consent, death, or study termination, whichever occurs first.

Safety:

Safety will be assessed according to vital signs, physical examinations, laboratory tests, ECG, ECOG performance status, and the number and severity of AEs and serious adverse events (SAEs).

AEs will be defined using preferred term (PT) and system organ class (SOC) in International Conference on Harmonization (ICH) Medical Dictionary for Regulatory Activities (MedDRA).

The safety of the study drug will be assessed per CTCAE v4.03.

Pharmacokinetics and immunogenicity:

Detect the concentration of HLX10 in serum and analyze the presence of anti-drug antibodies (ADA/NAb)

Statistical Analysis Methods:

Analysis Population:

- Intent-to-treat (ITT) set: defined as all subjects randomized into the study. ITT population will be considered as the primary analysis population for efficacy analysis in this study. The analysis of ITT population will be conducted based on randomized treatment groups.
- Per protocol set (PPS): PPS is a subset of ITT set. All randomized subjects without major protocol deviations significantly affecting the primary efficacy evaluation constitute the PPS. The definition of specific PPS will be confirmed before database lock. Analysis based on PPS will, as supportive analysis, complement the analysis based on ITT.
- Safety set (SS): defined as all subjects who have received at least one dose of study drug. The safety population will be the primary analysis population for safety assessment, and will be analysed based on actual treatment groups.
- Pharmacokinetic set (PKS): all subjects who have received at least one dose of HLX10, and have at least one post-dose detectable concentration at a planned PK time point and have no major protocol deviations that may impact the PK evaluation significantly. The PK set will be used for PK analysis.

Sample size estimation:

This study uses parallel dual primary endpoints of PFS and OS, where one interim analysis is planned for OS when a target number of PFS events is observed and one final analysis is planned for OS when a target number of OS events is observed, while only one final analysis is planned for PFS. To control the overall type I errors, the allocation of α is as follows:

PFS: $\alpha = 0.005$ (one-sided)

OS: $\alpha = 0.02$ (one-sided)

In this study, subjects are randomized into the treatment group and the control group at a ratio of 2:1. The sample size is based on the assumption that the median progression-free survival (PFS) is 5 months in the control group, i.e., placebo + chemotherapy (cisplatin + 5-FU), and that the median PFS is 7.35 months in the HLX10 + chemotherapy group, i.e., a hazard ratio (HR) of the HLX10 + chemotherapy group to the control group is approximately 0.68. When type I error $\alpha = 0.005$ (one-sided) and 24-month enrollment are assumed and the final analysis of PFS is planned to be performed at approximately month 4 after the last subject is enrolled, a minimum of 339 PFS events needs to be observed to achieve a power of 80%. Assuming a drop-out rate of 10%, a total of 495 subjects need to be enrolled in the 2 groups (330 in the HLX10 group and 165 in the control group).

Assuming that the median overall survival (OS) of the control group is 10 months and that the median OS of the HLX10 + chemotherapy group is 13.70 months, namely, the HR of the HLX10 + chemotherapy group to the control group is approximately 0.73. One interim efficacy will be analyzed by the Group Sequential Design when a target number of PFS events is observed, and the O'Brien-Fleming-like α-spending function of the Lan-Demets algorithm will be used to control the overall type I error rate $\alpha = 0.002$ (one-sided). Assuming that the duration of enrollment is 24 months and that the final analysis of OS is scheduled to be performed at approximately month 12 after the last subject is enrolled, a minimum of 388 OS events needs to be observed to achieve a power of 80% and a total of 540 patients needs to be enrolled in both groups (360 cases in the HLX10 group and 180 cases in the control group).

Considering the sample size required for PFS and OS evaluation, a total of 540 patients (360 in the HLX10 group

and 180 in the control group) need to be enrolled in this study.

Efficacy analysis:

- Primary efficacy endpoint
  - The progression-free survival (PFS) assessed by Independent Radiological Review Committee (IRRC) as per RECIST v1.1;
  - Overall survival (OS);

Inter-group comparison of PFS and OS will be performed using stratified Log-Rank test with the following stratification factors: PD-L1 expression level (CPS < 10 versus CPS ≥ 10), age (≥ 65 years versus < 65 years), and tumor state (locally advanced versus distant metastasis). Significance levels of PFS are 0.01 (two-sided) at the final analysis; significant levels of OS are 0.014 (two-sided) at the interim analysis and 0.036 (two-sided) at the final analysis; HR and its 95% confidence interval (CI) will be estimated by stratified COX proportional hazards model; the Kaplan Meier method will be used to estimate the median PFS/OS and 95% CI (Brookmeyer-Crowley method) with Kaplan-Meier curves plotted.

With the α redistribution strategy of Group Sequential Holm Procedure, if the original hypothesis is rejected in the interim/final analysis for a primary efficacy endpoint (i.e., the actual P value at the analysis time point is less than the corresponding nominal significant level), all the initial α allocated to the primary efficacy endpoint will be recovered and allocated to another primary efficacy endpoint that does not reject the original hypothesis. And the O'Brien-Fleming-like α-spending function (Lan-DeMets approximation) will be used to recalculate and update significant levels at each analysis time point. For example, in the final analysis of PFS, if the P value of Log-Rank test is less than 0.01, while the P value of OS is greater than or equal to 0.014, all α (one-sided, 0.005) initially allocated to PFS will then be recovered and allocated to OS, i.e., in this case, the total α update of OS is 0.025 (one-sided). The O'Brien Fleming-like α-spending function (Lan-DeMets approximation) will be used to recalculate the significant level of each interim analysis/final analysis of OS, and the actually calculated P value of OS will be compared with the updated significant level.

- Secondary efficacy endpoints
  - Progression-free survival (PFS) assessed by the IRRC as per iRECIST, and PFS assessed by the investigator as per RECIST v1.1 and iRECIST, respectively: The same statistical method will be used as that for the primary efficacy endpoint;
  - Objective response rate (ORR): Includes ORRs assessed by IRRC as per RECIST v1.1 and iRECIST, respectively, as well as ORRs assessed by the investigator as per RECIST v1.1 and iRECIST, respectively. The stratified Miettinen-Nurminen method will be used to test the inter-group difference in the ORRs and their 95% CIs. Stratification factors: Level of PD-L1 expression (CPS < 10 versus CPS ≥ 10), age (≥ 65 years versus < 65 years), and tumor state (locally advanced versus distant metastasis);
  - Duration of response (DOR): Includes DORs assessed by IRRC as per RECIST v1.1 and iRECIST respectively, as well as DORs assessed by the investigator as per RECIST v1.1 and iRECIST respectively.

The Kaplan-Meier method is used to estimate the median DOR, and the Kaplan-Meier curve will be plotted.

Interim analysis and final analysis:

In this study, an Independent Data Monitoring Committee (IDMC) will be established to conduct the interim analysis. The IDMC will be responsible for monitoring of the study safety and efficacy data, assessing the study implementation quality, suggesting study design adjustments, and other emergency analyses and suggestions under blinded conditions and determined by the IDMC.

In this study, one efficacy interim analysis is planned for OS when a target number of PFS events is observed and one final analysis is planned for OS when a target number of OS events is observed, while only one final analysis is planned for PFS. An O'Brien-Fleming-like α-spending function (Lan-DeMets approximation) will be used to control the overall type I error rate.

- The interim analysis of OS is planned to be performed during the final analysis of PFS, with the primary objective to perform safety assessments and superiority tests of PFS and OS endpoints. The significance level of Log-Rank test of PFS in this analysis is 0.01 (two-sided). According to O'Brien Fleming α-spending function, the significance level of Log-Rank test of OS in this analysis (about 75% OS event information) is 0.014 (two-sided);
- The final analysis of OS is planned to be performed when a target number of OS events (approximately 388) is observed, and the significance level α is 0.036 (two-sided) for final OS endpoint analysis;
- Significance level for each analysis will be modified based on the actual number of PFS and OS events reached at the analytical time point. If an original hypothesis is rejected for an endpoint at the analytical time point, the recovery and redistribution of the α endpoint are performed and the significant level of the other endpoint at each analytical time point is updated.

Safety Analysis:

AEs will be described per MedDRA (version 22.0), and graded per CTCAE v4.03 AEs during or after administration of study drug will be summarized per CTCAE grades. Adverse events and concomitant medications during study treatment will be separately summarized by treatment groups. Clinical laboratory parameters, ECOG, vital signs, physical examinations, and ECG will be summarized by treatment group and visit. Analysis will describe and present the observed values and changes from baseline by visit in the study.

Pharmacokinetics:

Descriptive statistics will be used to analyse the serum concentrations at each time point.

Immunogenicity:

Descriptive analysis of ADA/NAb positive rates of HLX10.

The statistical method will be detailed in the statistical analysis plan (SAP).

Quality of life assessment:

The quality of life assessment is analyzed using a summary index suitable for Chinese population (based on MULT8r) for statistical analysis.

The statistical method will be detailed in the statistical analysis plan (SAP).

STUDY SCHEDULE

**Period**	**Screening**		**Treatment (2 weeks/cycle)**					**End of treatment**[1]	**Follow-up**[2]	
**Cycle/visit name**	Screening		1	2	3	4	n	End of treatment	Safety follow-up	Survival follow-up[3]
**Visit time**								Knowing or confirming that the subjects have discontinued the treatment	30 days and 90 days after the last dose (by telephone calls)[2]	Every 12 weeks
**Window (day)**[4]	-28 to -8	-7 to -1		± 3	± 3	± 3	± 3	+ 3	± 7	± 7
**Study management procedure**										
Informed consent form	**X**									
Inclusion/exclusion criteria	**X**									
Demographics and medical history	**X**									
Prior and concomitant therapies[5]	**X**			**X**	**X**	**X**	**X**	**X**	**X**	
**Clinical operations/ evaluations**										
AEs[6]	**X**		**X**	**X**	**X**	**X**	**X**	**X**	**X**	
Quality of life[7]		**X**			**X**		**X**	**X**		
Echocardiography	**X**									
12-lead ECG		**X**		**X**	**X**	**X**	**X**	**X**	**X**	
Complete physical examination	**X**									
Symptom-directed physical examination			**X**	**X**	**X**	**X**	**X**	**X**	**X**	
Height, weight, and vital signs[8]	**X**		**X**	**X**	**X**	**X**	**X**	**X**	**X**	
ECOG score		**X**		**X**	**X**	**X**	**X**	**X**	**X**	
Subsequent anti-tumor therapy									**X**	**X**
Survival status			**X**	**X**	**X**	**X**	**X**	**X**	**X**	**X**

**Period**	**Screening**		**Treatment (2 weeks/cycle)**					**End of treatment[1]**	**Follow-up[2]**	
**Study drug**										
Randomization[9]			**X**							
HLX10/placebo + chemotherapy[9]			**X**	**X**	**X**	**X**	**X**			
**Laboratory operations/ assessments: conducted at the sites**										
Pregnancy test[10]		**X**			**X**		**X**	**X**	**X**	
Haematology, blood biochemistry, coagulation, urinalysis, and cardiac markers[11]		**X**		**X**	**X**	**X**	**X**	**X**	**X**	
Thyroid function[12]		**X**			**X**		**X**	**X**	**X**	
Trypsin[13]	**(X)**		**(X)**	**(X)**	**(X)**	**(X)**	**(X)**	**(X)**	**(X)**	
**Virology examination**										
HBV surface antigen/core antibody and HBV DNA[14]	**X**									
● If at baseline: HBV DNA (< 500 IU/mL or 2500 copies/mL) and 1.HBsAg (+), or 2. HBcAb（+）, the five items of hepatitis B and HBV DNA need to be tested during treatment period.					**X**		**X**	**X**	**X**	
HCV antibody and HCV RNA[14]	**X**									
● If at baseline: In case of HCV antibody (+) and HCV RNA (-), HCV antibody and HCV RNA need to be tested during treatment period.					**X**		**X**	**X**	**X**	

Period	Screening	Treatment (2 weeks/cycle)					End of treatment[1]	Follow-up[2]	
HIV antibody	**X**								
**Laboratory operations/ assessments: conducted by central laboratory**									
HLX10-PK and ADA/NAb[15]	**X**	X	**X**	**-**	**X**	**X**	**X**	**X**	
Expression of PD-L1 in tumor tissue	**X**								
Expression of MSI and TMB in tumor tissue	**X**								
MSI and TMB tests of blood samples	**X**								
**Efficacy evaluation**									
Radiological examinations[16]	**X**	**X**					**X**	**(X)**	**(X)**
**Biomarker sample collection**									
Tumor tissue samples[17] (PD-L1, MSI, and TMB)	**X**								
Blood samples[17] (MSI and TMB)	**X**								

1. For a subject who discontinues the study treatment for any reason, an end-of-treatment (EOT) visit should be performed whenever possible. If the subject has completed the laboratory tests specified in the study flow chart within 7 days (including day 7) prior to the EOT visit, repeated tests are not required; if the EOT visit is completed within 30 (± 7) days after the last dose of the investigational product, no safety follow-up visit is required. This study **allows re-screening** and re-screened subjects will be assigned with new screening numbers.
2. Subjects who discontinue the treatment for reasons other than PD should try to continue the radiological assessments per schedule **until PD (including the PD determined by RECIST v1.1 and that by iRECIST, if any)**, initiation of new anti-tumor therapy, withdrawal of informed consent, death, or study termination, whichever occurs first. Patients should receive a safety follow-up at the study site 30 (± 7) days after the last dose of the investigational product (HLX10). Prior to the initiation of new anti-tumor therapy, patients are required to receive a safety follow-up by telephone 90 ± 7 days after the last dose (HLX10), and only the information of AE and AE-related concomitant medications will be collected.
3. The subjects should be followed up for survival via telephone calls every 12 weeks ± 7 days before initiation of a new anti-tumor treatment or termination of all study drugs; the frequency of survival follow-up can be increased if appropriate.

Period	Screening	Treatment (2 weeks/cycle)	End of treatment[1]	Follow-up[2]

4. The window of the screening period is 28 days, the window of the treatment period and the end-of-treatment visit is 3 days (the tumor assessment window is 7 days), and the window of the follow-up period is 7 days. For ECOG performance status, pregnancy test, haematology, serum chemistry, coagulation, urinalysis, thyroid function, and cardiac marker tests during screening period, data **within 7 days prior to the first dose of the study drug** should be documented, and the subjects should meet the corresponding inclusion criteria and not meet any exclusion criteria for enrolment.
5. Records of prior medications taken from 30 days before screening visit to the date of signing informed consent form will be collected. Records of concomitant medications taken from the date of signing informed consent form to 30 days after the last dose of the investigational product will be collected. AE-related concomitant therapies are to be recorded to **90 days** after the last dose of the investigational product.
6. All AEs, as well as SAEs, are to be collected and recorded from the first dose of investigational product (C1D1) to the subject, until 90 days after the last dose or the start of new anti-tumor treatment (whichever occurs first), after which only SAEs related to the investigational product (HLX10/placebo) are recorded. For SAEs that occurred after ICF signature and before the first dose of investigational product (C1D1), only SAEs (within 24 hours of being informed) resulting from protocol-mandated interventions (e.g., invasive manipulations and biopsies) are to be reported.
7. Quality of life questionnaires include European Quality of Life Five-Dimension Five-Level Scale (EQ-5D-5L) and European Organization for Research and Treatment of Cancer Quality of Life Questionnaire - C30 (EORTC QLQ-C30) and European Organization for Research and Treatment of Cancer Quality of Life Questionnaire - Oesophageal Cancer Module (EORTC QLQ-OES18). Quality of life assessment will be performed in the screening and every 2 cycles after the first dose, i.e. assessment will be completed before administration in Cycles 3, 5, 7... until end of treatment. If no assessment has been performed within past 6 weeks at end-of-treatment visit, one quality of life assessment needs to be performed at this visit.
8. **Height will only be measured at screening**; vital signs include body temperature, pulse, respiratory rate, and blood pressure. Body weight will be measured before each dose. If the change of the subject's body weight from **baseline** (body weight before the first dose) is ≤ 10% during treatment, the dose modification of study drug is not necessary. If the change of body weight is >10%, the dose needs to be recalculated, and at this time, the new weight will be used as the baseline value for subsequent weight measurements.
9. The study drug will be administered on Day 1 of each cycle after all clinical and laboratory operations/assessments are completed, and every two weeks is a cycle. (The time interval from the date of randomization to the start date of treatment (i.e. date of first administration) shall not exceed 3 days).
10. Women of childbearing potential can only be enrolled when they have negative blood pregnancy test within 7 days prior to the first dose of the study drug. During treatment period, the pregnancy test will be performed within 3 days **prior to every two doses** (blood or urine. If urine pregnancy test is positive, blood pregnancy is further required). The administration of the study drugs must be scheduled after the results of the tests are available. Analysis will be performed at the local site.
11. Laboratory tests include haematology, blood biochemistry, coagulation, urinalysis, and cardiac markers. Haematology includes red blood cell count, haemoglobin, platelet and white blood cell count, white blood cell count with differential and percentage (neutrophils, lymphocytes, basophils, eosinophils, and monocytes);

Period	Screening	Treatment (2 weeks/cycle)	End of treatment[1]	Follow-up[2]

blood biochemistry includes serum urea/urea nitrogen, creatinine, sodium, potassium, magnesium, chloride, bicarbonate/carbon dioxide combining power/total carbon dioxide (TCO_2), calcium, phosphorus, blood glucose, total and direct bilirubin, alanine aminotransferase (ALT), aspartate aminotransferase (AST), alkaline phosphatase, lactate dehydrogenase, total cholesterol, total protein, and albumin; urinalysis includes: specific weight, pH, urine glucose, urine protein, urine casts, ketone body, and blood cell; coagulation includes: international normalized ratio (INR), prothrombin time (PT), and activated partial thromboplastin time (aPTT); cardiac markers include: myocardial enzymes (creatine kinase (CK) and its isoenzymes, troponin (TnI or TnT)), and brain natriuretic peptide (BNP and/or NT-proBNP). These tests will be performed within **3 days prior to each cycle**; when the above laboratory tests are arranged on the same day as administration of study drug, administration can only be performed after the test results are obtained.

12. Thyroid function test includes the analysis of triiodothyronine (T3 or FT3), thyroxine (T4 or FT4), and thyroid-stimulating hormone (TSH). During treatment period, the thyroid function test will be performed within 3 days **prior to every two doses**. The administration of the study drugs must be scheduled after the results of the tests are available. Analysis will be performed at the local site.
13. Tests for trypsin (trypsin, serum amylase, and lipase) are **optional** and could be performed selectively based on the routine practice of local study sites. (x): optional tests.
14. All subjects are tested for HBsAg/HBcAb or anti-HCV antibody at screening; HBsAg or HBcAb positive subjects have to be analysed by HBV DNA titre, and subjects tested positive for anti-HCV antibody have to receive further HCV RNA assay. At screening (baseline), if: HBV DNA (< 500 IU/mL or 2500 copies/mL) and 1. HBsAg (+), or 2. HBcAb (+), the subject needs to be tested **every 2 cycles** during the treatment period for the five indicators of hepatitis B: 1). HBsAg (hepatitis B surface antigen); 2). HBsAb (Hepatitis B surface antibody); 3). HBeAg (Hepatitis B e antigen); 4). HBeAb (Hepatitis B e antibody); 5). HbcAb (Hepatitis B core antibody) and HBV DNA. In case of anti-HCV antibody (+) and HCV RNA (-) at baseline, anti-HCV antibody and HCV RNA should be tested **every 2 cycles** during the treatment period.
15. PK and ADA (NAb) sampling: (Note: ADA/Nab samples will only be collected pre-dose and procedures are described in the laboratory manual.)
    - HLX10-PK and ADA (NAb) samples will be collected at the following time points: within 24 h prior to the dosing of HLX10 in cycles 1, 2, 4, 6, 8, and every 4 cycles thereafter, within 0.5 h after the dosing of HLX10 in cycles 1 and 8 of the treatment period (for PK only), and at EOT visit and safety follow-up.
16. CT or MRI test will be performed at screening, every 6 weeks (± 7 days) in the first 48 weeks after randomized medication, and every 12 weeks (± 7 days) after 48 weeks (test sites include neck, chest, abdomen, pelvis, and any other sites suspected of tumor lesion; **MRI or CT of the brain** (MRI is preferred) must be performed at baseline, and will be performed by the investigator as clinically indicated during treatment; **bone scan** will be performed by the investigator as clinically indicated at baseline and during treatment); the test method for the same site should be maintained as consistent as possible throughout the study; contrast should be used if there is no contraindication. The investigator and the IRRC will separately assess the radiological results per RECIST v1.1 (the frequency of tumor assessments can be increased by the investigator as clinically indicated). The investigator should determine subsequent treatment based on his/her efficacy assessment. For subjects who continue with treatment after first PD (RECIST v1.1), radiological assessment needs to be conducted again

Period	Screening	Treatment (2 weeks/cycle)	End of treatment[1]	Follow-up[2]
after an interval of 4–8 weeks. If tumor assessment has been performed within 28 days prior to first dose in the same hospital using the same method and machine, then this tumor assessment can be taken as baseline tumor assessment. At the end-of-treatment visit, if radiological examination of the tumor has been performed within past 4 weeks, there is no need to take a re-test. **Subjects who discontinue the treatment for reasons other than PD should continue the radiological assessments per schedule** until PD, initiation of new anti-tumor therapy, withdrawal of informed consent, death, or study termination, whichever occurs first.<br>17. Subjects **must** provide formalin-fixed paraffin-embedded (FFPE) tumor samples (paraffin blocks or unstained slides) collected at non-radiotherapy sites and relevant pathological reports of these samples. In the absence of recent archival tumor tissue samples, a fresh biopsy of a tumor lesion at screening will be accepted in order to obtain the corresponding tumor samples (the number of samples depends on biopsy). The tumor tissue sections and blood samples will be used for testing the expression level of PD-L1, MSI, and TMB (where PD-L1 is required, while MSI and TMB are optional). Fresh sample collection, resection, core needle biopsy, and excisional, incisional, punch, or forceps biopsies are all acceptable. Needle aspiration samples (i.e. samples lacking of complete tissue structure and only providing cell suspension and/or cell smear), brushing samples, and cell pellet samples from pleural or peritoneal effusion are not acceptable. **See the Laboratory Manual for detailed requirements for tissue samples.**				

1 INTRODUCTION

1.1 Study Background

Oesophageal cancer is a malignancy mainly originated from oesophageal epithelial cell and includes the major pathological types of oesophageal squamous cell carcinoma (ESCC) and oesophageal adenocarcinoma (EAC). The distribution of pathological types varies in different regions. EAC is the main pathological type in Western countries such as Europe and US, accounting for approximately 70%, while more than 90% of oesophageal cancer is ESCC in China[1]. Oesophageal cancer is highly prevalent in Asia, South Africa, East Africa, and Southern France. Oesophageal cancer is the sixth leading cause of cancer deaths worldwide[2], and the fourth leading cause of cancer deaths in developing countries[3].

According to Chinese Journal Of Cancer Research 2018 database[4], in 2014 the number of new oesophageal cancer cases was about 258,000 in China, resulting the sixth most common cancer nationwide; the number of deaths was about 193,000, leading to the fourth common cause of cancer-related mortality nationwide. Main risk factors causing oesophageal cancer include nitrosamines and their precursors as well as some fungi and their toxins. Tobacco, heavy alcohol consumption and age are also closely related to oesophageal cancer. Age > 40 years is one of high risk factors causing oesophageal cancer[1].

Because the symptoms of early oesophageal cancer are always not obvious, the anti-cancer awareness of Chinese people is weak and screening conditions are limited, most patients are truly in clinical middle or advanced stage at the time of diagnosis, losing the opportunity for surgical treatment. At present, systemic therapies (chemotherapy/targeted therapy) are mainly adopted for these patients in clinic. However, the efficacy of both chemotherapy and targeted therapy has been limited with high rate of recurrence and metastasis. Globally, the 5-year survival rate of patients with middle and advanced oesophageal cancer is still lower than 15%, the objective response rate is only 20%-40%, and the median survival is about 8–10 months[5]. In China, the 5-year survival rate of oesophageal cancer is about 30%, and it's only 18% in urban areas, far below 33.2% in rural areas, and also with a trend of declining[6]. Therefore, there is an urgent need to explore therapies which can significantly improve the outcome of patients with oesophageal cancer.

In the past 20 years, with the deep research of tumor immunology, tumor immunotherapy has become one of research focuses in the world. Tumor immunotherapy was evaluated as top of scientific breakthroughs of the year by *Science* in 2013. PD-1 was initially cloned from mouse T cell hybridoma by Ishida et al.[7] as a member of CD28 superfamily, and as a monomer

glycoprotein. Because the activation of PD-1 gene may participate in the classical type of programmed cell death, it was named as programmed cell death receptor-1. PD-1 is mainly expressed on the activated macrophages, T lymphocytes, B lymphocytes, NK cells, and some bone marrow cells, and its ligands PD-L1 (programmed cell death ligand-1) and PD-L2 are mainly expressed in tumor cells and antigen presenting cells[8,9,10]. The binding of PD-1 to PD-L1 weakens the immune response of human bodies, protecting tumor tissues from attack by cytotoxic T cells to induce immune tolerance of tumor[13,14]. Therefore, the anti-tumor response of T cells can be increased by blocking the binding of PD-1 to its ligand PD-L1[15], making PD-1/PD-L1 one of hot topics in recent tumor immunotherapy research.

Several experiments have proven that PD-L1 is highly expressed in oesophageal cancer[11,12], and the expression of PD-L1 is closely related to the depth of tumor invasion and poor prognosis. Currently, pembrolizumab (trade name: Keytruda) has been approved by FDA for the treatment of PD-L1-positive patients with recurrent locally advanced or metastatic gastric/gastroesophageal junction cancer. This breakthrough has great significance in treatment of oesophageal cancer.

On 14 Nov., 2018, Merck & Co Inc. announced on its official website that its phase 3 clinical study KEYNOTE-181 to evaluate PD-1 tumor immunotherapy pembrolizumab for treatment of advanced or metastatic oesophageal or gastroesophageal junction cancer had achieved significant improvement in OS. This study included more than 600 subjects with advanced or metastatic EAC or ESCC, or Siewert type I adenocarcinoma of esophagogastric junction, who had progressive disease after first-line treatment. These subjects were randomized at a ratio of 1:1 to receive pembrolizumab monotherapy or chemotherapy. The study results showed that pembrolizumab had achieved statistically significant improvement in OS in PD-L1-positive (CPS $\geq$ 10) patients compared with the chemotherapy, reaching the primary endpoint. Although the key secondary endpoints PFS and ORR had not been formally evaluated yet, the improvement of OS in the ITT study population and patients with squamous histology demonstrated a favourable trend.

On 09 Jan., 2019, Ono Pharmaceutical announced on its official website that its phase 3 clinical study ATTRACTION-3 had achieved positive results. This study included 390 subjects with unresectable advanced/recurrent oesophageal cancer, who were randomized to receive nivolumab monotherapy or chemotherapy (docetaxel or paclitaxel). The study results showed that the nivolumab had achieved statistically significant improvement in OS compared with the chemotherapy, reaching the primary endpoint (OS); the improvement in OS was unrelated to the status of PD-L1. The complete results of the study will be published later. Domestic pharmaceutical enterprises such as Hengrui Medicine and BeiGene have also successively

conducted phase 3 clinical studies for PD-1 inhibitors in oesophageal cancer. More studies related to immunotherapy for advanced oesophageal cancer will be conducted at home and abroad in future. Immunotherapy has become a new direction in treatment of oesophageal cancer.

1.2 Rationale for Conduct of Study and Dose Selection

1.2.1 Nonclinical overview of HLX10

HLX10 is an innovative monoclonal antibody against PD-1 target, which is developed by Shanghai Henlius Biotech, Inc. independently. HLX10 is a humanized IgG4 monoclonal antibody. Its gene sequence is screened by the hybridoma technique, and humanization is completed through genetic engineering. CHO is used as a host cell to construct a stable cell strain, and the produced protein has typical Y-shaped human immunoglobulin IgG4 structure formed by connecting two identical heavy chains and two identical light chains via disulfide link.

In-vitro pharmacodynamics of HLX10

A series of *in-vitro* PD studies of HLX10 versus positive control nivolumab showed that: HLX10 bound to the surfaces of activated T cells expressing PD-1 and had the effect to block the binding of PD-1 to its ligand PD-L1 or PD-L2. Both binding and blocking effects were dose-dependent. The *in-vitro* mixed leucocyte reaction (MLR) analysis of HLX10 showed that HLX10 blocked the immunosuppressive effect depending on the binding pathway of PD-1 to its ligands to further stimulate activated $CD4^+$ T cells, so that the T cells were improved in cell reproductive capacity and produced more IL-2 cytokines. This phenomenon demonstrated dose dependence in both HLX10 and positive control nivolumab groups.

Moreover, in order to study the PD-1 receptor occupancy of HLX10 on human T cells, different doses of HLX10 [redacted] were preincubated with whole blood form [redacted] healthy subjects to simulate the injection of HLX10 into human blood. The experiment results showed: The PD-1 receptor occupancy on $CD3^+$ T cells increased with the increase of HLX10 dose for preincubation. In the [redacted] healthy subjects, once the serum concentration of HXL10 reached [redacted] the PD-1 receptor occupancy on $CD3^+$ T cells had already reached more than [redacted] in [redacted] of them.

***In-vivo* pharmacodynamics of HLX10**

The anti-tumor efficacy and safety assessments in the HT-29 human colon cancer cell NOD/SCID immunodeficiency mice subcutaneous xenograft tumor model showed that, HLX10 had no adverse effects on the health status and body weight of mice in all dose groups, indicating

high safety of HLX10. As for tumor inhibition, HLX10 at [redacted] effectively inhibited the growth of HT-29 human colon cancer cell NOD/SCID immunodeficiency mouse subcutaneous xenograft tumor (P < 0.0001) in the presence of human peripheral blood mononuclear cells.

The anti-tumor efficacy and safety assessments in the NCI-H292 human NSCLC cell NOD/SCID immunodeficiency mice subcutaneous xenograft tumor model showed that, HLX10 at higher doses had no adverse effects on the health status and body weight of mice in all dose groups, indicating high safety of HLX10. As for tumor inhibition, by comparing high-dose HLX10 and placebo, both tumor volume measurements and data statistics showed that, HLX10 at [redacted] significantly inhibited the growth of NCI-H292 tumor tissues (P < 0.001) in the presence of human peripheral blood mononuclear cells.

In dose-finding study (P16-106-TS), PK study (P16-106-YD), and long-term toxicity study in the cynomolgus monkeys (P16-106-CD), the receptor occupancy (RO) at different time points was concomitantly studied after intravenously injecting different doses of HLX10, to provide a basis for selection of clinically effective dose and first dose.

Final results showed that, the results of human peripheral blood PD-1 RO *in vivo* were consistent with that *in vitro*, i.e. once the serum concentration of HLX10 in subjects reached [redacted], the PD-1 RO on $CD3^+$ T cells had already reached more than [redacted] In cynomolgus monkeys, when the serum concentrations of two tested animals in [redacted] group were lower than the lower limit of detection [redacted] their corresponding RO values were still [redacted] and [redacted] respectively. With all results together, it can be concluded that: when [redacted] of HLX10 is given to cynomolgus monkeys in single dose, RO saturation rate can be maintained for more than [redacted] weeks; when a dose of [redacted] is continuously given to cynomolgus monkeys for [redacted] weeks (once a week [QW]), some animals can still reach [redacted] RO saturation rate after end of [redacted] week recovery period. It can be inferred that a clinically low dose (lower than [redacted]) of HLX10 may reach RO saturation and demonstrate good efficacy.

Tissue cross reactivity of HLX10

The tissue cross reactivity experiments of HLX10 in human tissues showed: HLX10-Biotin ([redacted] specifically bound to normal human lymphocytes, including lymph nodes, lungs, ileum, stomach, spleen, fallopian tube, colon, and thymus tissue.

The tissue cross reactivity experiments of HLX10 in cynomolgus monkey tissues showed: HLX10-Biotin ([redacted] specifically bound to normal cynomolgus monkey lymphocytes, including stomach, jejunum, colon, spleen, thymus, and mesenteric lymph nodes.

General pharmacology of HLX10

A general pharmacological evaluation for central nervous system, cardiovascular system, and respiratory system was conducted in cynomolgus monkeys. This study was a concomitant study of the long-term toxicity study. During the study, no test article-related abnormalities in clinical symptoms were observed in animals in each group, and no obvious abnormalities were observed in their behavioural activities and breathing; in high-dose group, abnormal findings of the female dying animal euthanized on D[redacted] were found to be related to amoebic infection, and abnormal findings of the female animal died on D[redacted] were found to be related to allergic reactions caused by administration. No toxicologically significant regular changes were seen in living animals in body temperature, systolic blood pressure, diastolic blood pressure, mean arterial pressure, oxygen saturation, heart rate, and ECG parameters such as P-R interval, Q-T, QTc interval, and QRS duration. Therefore it was considered that no obvious effects were observed on central nervous system, cardiovascular system, and respiratory system when doses at [redacted] iv infusion, QW were given to cynomolgus monkeys for consecutive [redacted] weeks.

Acute toxicity of HLX10

A dose-finding toxicity study was performed in cynomolgus monkeys receiving repeated iv infusions of HLX10 for [redacted] weeks at [redacted] Administration was on D[redacted] and all animals were euthanized on D[redacted] for gross anatomy. No death or impending death was observed in animals during the study. In the middle-dose group, one male animal had loose and soft stools on D[redacted] and D[redacted] respectively, and another male animal also had loose stools on D[redacted], D[redacted] and D[redacted] which were considered to be test article-related. No test article-related abnormal symptoms were observed in animals in the low-dose group and high-dose group. No abnormal administration-related changes in body weight, food consumption, body temperature, ECG parameters, coagulation parameters, serum chemistry, or gross anatomy were seen in animals in all dose groups. [redacted] [redacted] and [redacted] of animals were found ADA positive in the [redacted], [redacted], and [redacted] dose groups, respectively. The first occurrence of ADA was D[redacted] and the titre range was [redacted] In this study, HLX10 at [redacted] was well tolerated when given to the cynomolgus monkeys for [redacted] weeks. The dose of [redacted] can be selected for longer-term repeated-dose study.

Long-term toxicity of HLX10

A toxicity and toxicokinetics (TK) study was performed in cynomolgus monkeys receiving repeated iv infusions of HLX10 for [redacted] weeks with [redacted]-week recovery. Placebo and HLX10 at [redacted], iv infusion, QW were given for consecutive [redacted] weeks. No test article-related death or impending death was observed in animals in the low-dose and

mid-dose groups during the study. At the dose of [redacted] [redacted] female animal died due to administration-related allergic reactions; [redacted] female animal was euthanized in dying state on D[redacted] because of amoebic infection. Loose stools and/or soft stools could be seen in all dose groups, but were slightly more frequent and longer in the female animals in the high-dose group ([redacted]). These gastrointestinal symptoms might be associated with administration and were consistent with the adverse reactions reported for PD-1 monoclonal antibodies. Except for [redacted] deaths in the high-dose group, gastrointestinal reactions (loose stools/soft stools) in the other animals were completely recovered after [redacted]-week recovery period. No other abnormal test article-related symptoms were seen in living animals in the administration groups during the study. All animals in the administration groups were ADA positive except for the female animal euthanized on D[redacted] in the high-dose group, and the ADA rate was highest in the low-dose group. The antibody titre increased with the increased frequency of administration, and continued until the end of recovery period (D[redacted]). Based on the TK results, the presence of ADA obviously reduced the systemic exposure in all dose groups. After repeated iv infusions of HLX10 at [redacted], QW for consecutive [redacted] weeks, no obvious toxicity was seen in cynomolgus monkeys and no local irritations were seen at the sites of administration. No obvious effect was identified on vital functions, including cardiovascular system, central nervous system, or respiratory system. The maximum no observed adverse effect level (NOAEL) of HLX10 under the conditions of this study was [redacted] At this dose, AUC_{last} and C_{max} values in male and female animals on D[redacted] were [redacted] and [redacted], and [redacted] and [redacted] respectively.

Preclinical pharmacokinetics of HLX10

PK study results of single iv infusion of HLX10 in cynomolgus monkeys showed that: after iv infusion at [redacted] the serum concentration increased with the dose. Systemic exposure (C_{max} and AUC_{last}) also increased with the dose. The mean retention time (MRT) was [redacted]. The elimination half life ($t_{1/2}$) was [redacted] The results showed that the PK profile of the test article was mostly linear over the dose range of [redacted] in cynomolgus monkeys. The clearance [redacted] and volume of distribution (Vz) [redacted] of HLX10 were similar in all dose groups. All animals in the [redacted] HLX10 groups were ADA positive on D[redacted]. The first presence of ADA was D[redacted], and the titre range was [redacted] With the exception that female animals in the [redacted] group had statistically significant lower area under plasma concentration-time curve (AUC_{last} and AUC_{inf}) than male animals ($p < 0.05$), the sex differences in PK parameters were not obvious in the other dose groups. Based on antibody production analysis: [redacted] of female animals in the [redacted] group produced strong antibodies on D[redacted] and the antibody titre was

[redacted], causing faster elimination in female animals than in male animals and lower area under curve (AU_{Clast} and AUC_{inf}).

Toxicokinetics of HLX10

A TK study was concomitant with the dose-finding toxicity study in cynomolgus monkeys receiving repeated iv infusions of HLX10 for [redacted] weeks. The results showed: Systemic exposure (C_{max} and AUC_{last}) of HLX10 increased with the dose on D[redacted] and D[redacted]. Production of ADAs in some animals on D[redacted] caused faster elimination of HLX10 in plasma. In ADA-negative animals, the mean values of C_{max} and AUC_{last} in all dose groups on D[redacted] were higher than those on D[redacted], indicating some extent of drug accumulation. The plasma concentration of HLX10 in pre-dose samples on D[redacted] also showed this pattern. The accumulation factor was [redacted] The ADA-negative group had smaller volume of distribution and slower clearance of HLX10 on D[redacted] than on D[redacted]. The ADA-positive group had obviously increased clearance.

A TK study was also concomitant with the toxicity and TK study in cynomolgus monkeys receiving repeated iv infusions of HLX10 for [redacted] weeks with [redacted]-week recovery. The results showed: Systemic exposure (C_{max} and AUC_{last}) of HLX10 increased with the dose on D[redacted] and D[redacted] demonstrating dose-dependent TK profile. Some animals produced ADAs after repeated dose. In ADA-negative animals, the mean values of C_{max} and AUC_{last} in all dose groups on D[redacted] were higher than those on D[redacted] indicating drug accumulation. The plasma concentration of HLX10 in pre-dose samples on D[redacted] also showed this pattern. The accumulation factor was [redacted]. The ADA-negative group had smaller volume of distribution and slower clearance of HLX10 on D[redacted] than on D[redacted] The ADA-positive group had obviously increased clearance. The male animals in the [redacted] group had slightly higher systemic exposure (AUC_{last} and AUC_{INF}) and slightly lower clearance than female animals on D[redacted] and the sex differences in the PK parameters were not obvious in all the other dose groups on D[redacted] and D[redacted]

Immunogenicity and immunotoxicity of HLX10

An immunogenicity and immunotoxicity evaluation study was conducted in cynomolgus monkeys and it was concomitant with the long-term toxicity study. The study results showed: After repeated iv infusions of HLX10 at [redacted] QW for consecutive [redacted] weeks, no immunotoxicity was seen in cynomolgus monkeys at [redacted] and [redacted] One female animal at [redacted] died due to allergic reactions, with ADA positive and increased post-dose IL-6. $CD3^+$, $CD4^+$ and $CD4^+/CD8^+$ ratios reduced in male animals at [redacted] and the increase in $CD8^+$ was associated with the pharmacological effects of the test article. ADAs were detected in animals administered with HLX10, and the incidence of ADAs was higher in the low-dose group than in the mid-dose and high-dose groups. The antibody titre increased with the infusion time,

causing significantly reduced systemic exposure. HLX10 is a humanized antibody that is exogenous to cynomolgus monkeys, so it is reasonable to see immunogenicity in animals. It is suitable to evaluate the immunogenicity of antibody drugs in human clinical studies.

Preclinical studies of HLX10

Haemolysis test of HLX10 showed that, HLX10 at the concentration of [redacted] had no haemolytic effect on human red blood cells in vitro, causing no aggregation.

A local irritation study was concomitant with the long-term toxicity study, and the study results showed: In cynomolgus monkeys, after repeated iv infusions of HLX10 at [redacted] QW for [redacted] weeks, no obvious irritation damages were observed at the local injection blood vessel and surrounding tissues at the concentration of [redacted]

Genotoxicity studies have not been conducted with HLX10.

1.2.2 Clinical studies of HLX10

At present, HLX10 has been approved by FDA, TFDA, and China NMPA successively to conduct phase 1 clinical dose-escalation study. A total of four dose groups (0.3, 1, 3, and 10 mg/kg, Q2W) will be tested, and about up to 30 subjects will be included. Up to now, all patients in the four dose groups have completed the dose escalation and enrolment, as well as DLT assessment. **More subjects are being included in the extended enrollment of 10 mg/kg in the fourth dose group.**

1.2.3 Rationale for selection of chemotherapy as control

The chemotherapy regimen provided in this study is cisplatin + 5-FU, a recommended first-line chemotherapy regimen for metastatic or locally advanced oesophageal cancer by NCCN guidelines and China's Clinical Guidelines on Oesophageal Cancer (2018), allowing the investigator and subjects have adequate flexibility to perform study treatment according to the standard clinical practice of the drugs.

1.2.4 Rationale for HLX-10 and chemotherapy combination therapy

The above completed and ongoing clinical studies for immunotherapy in treatment of oesophageal cancer confirmed a significant potential of immunotherapy in oesophageal cancer. Meanwhile, several clinical studies of PD-1/PD-L1 inhibitors combined with chemotherapy in treatment of tumors are also ongoing. Both US KEYNOTE-585 study and KEYNOTE-590 study adopted chemotherapy combined with Keytruda in gastric or gastroesophageal junction cancer, which proved that the treatment mode of PD-1/PD-L1 inhibitors combined with chemotherapy would be a new direction in treatment of gastrointestinal tumors.

1.2.5 Rationale for selection of HLX10 dose

Based on the preclinical and clinical phase I results of HLX10, the currently available PK, and ADA data support HLX10 3 mg/kg (average weight) Q2W (once every two weeks (14 days)) as the recommended dose in phase 3 clinical study.

1.3 Benefit-Risk Assessment

1.3.1 Potential benefits

In recent years, several clinical studies overseas have observed the encouraging efficacy of immune checkpoint inhibitors in treatment of metastatic oesophageal cancer. Merck conducted one multicentre phase Ib study i.e. KEYNOTE-028 study[16] to evaluate pembrolizumab in treatment of PD-L1-positive patients with progressive oesophageal or gastroesophageal junction cancer. 23 PD-L1-positive patients were included to receive pembrolizumab (trade name: Keytruda) monotherapy. The results showed: The ORR reached 30%, and 52% of patients showed certain degree of tumor shrinkage. The incidence of Grade 3 treatment-related adverse events (TRAEs) was 17%, and no Grade 4 AEs, death, or treatment interruption due to AEs occurred.

Subsequently in 2016, Merck conducted one phase II clinical study i.e. KEYNOTE-180[17] to evaluate pembrolizumab monotherapy in treatment of previously treated patients with progressive oesophageal or gastroesophageal junction cancer. 121 subjects were included, 52% of them had squamous cancer, and 15% had received more than 4 lines of treatment. All 121 patients received pembrolizumab monotherapy. The results were as follows: the 12-month OS was 28%, the median OS was 5.8 months, the ORR was 10%, the median PFS was 2.0 months, and the incidence of Grade 3–5 AEs was 12%.

In 2014, Ono Pharmaceutical conducted one randomized, open-label, multicentre phase 2 clinical study to evaluate nivolumab in treatment of advanced ESCC patients failed in current standard chemotherapy, i.e. ATTRACTION-01[18] study. A total of 65 subjects with advanced ESCC were included, among which 64 subjects received nivolumab monotherapy, and 68% of patients received more than 4 lines of treatment. In 64 evaluable subjects, the ORR was 17.2%, the median OS was 10.78 months (95% CI: 7.39, 13.93), the median PFS was 1.51 months (95% CI: 1.41, 2.79), and the median duration of response was 11.17 months. 4.7% of subjects achieved CR, 43.5% of patients achieved one survival year, and 17.2% of patients achieved two survival years.

At present, most phase 3 clinical studies of PD-1 inhibitors in treatment of oesophageal cancer are ongoing, lacking of clinical data for support. Based on the preliminary results recently

published from KEYNOTE-181 by Merck and from ATTRACTION-3 study by Ono Pharmaceutical, PD-1 inhibitors have significantly improved the OS of patients with advanced oesophageal cancer when compared with chemotherapy. In conclusion, the study results suggested that treatment with PD-1 inhibitors may bring greater benefit to patients with advanced metastatic or recurrent oesophageal cancer.

1.3.2 Identified and potential risks

PD-1/PD-L1 inhibitors not only can enhance the anti-tumor effect of cellular immunity, but also can enhance the normal immune response of the human body to cause immunotolerance imbalance and immune-related adverse events (irAEs). irAEs may affect any organ of the human body. At present, near 2/3 of the patients treated with immune checkpoint inhibitors have experienced different degrees of irAEs. In February 2018, NCCN and ASCO jointly published guidelines for management of immunotherapy related toxicity. The guidelines state that irAEs are relatively common in skin, intestinal, endocrine, pulmonary and musculoskeletal systems and rare in cardiovascular, blood, renal, nerve, and eye systems. Most irAEs are mild to moderate in severity. The current common known irAEs in patients treated with PD-1/PD-L1 inhibitors include: skin toxicity (mainly including maculopapule and pruritus; 30%-40%), diarrhoea and/or colonitis (8%-19%), fatigue (16%-24%), immune-related hepatitis (5%), hypothyroidism (4%-10%), hyperthyroidism (4%), hypophysitis (<1%), type 1 diabetes, immune-related pneumonitis, sarcoidosis, inflammatory arthritis, etc. Other irAEs are infrequent, such as adverse cardiovascular events, anaemia, thrombopenia, nephritis, encephalopathy, leukoencephalopathy, reversible posterior leukoencephalopathy syndrome, peripheral motor and sensory neuropathy, uveitis, episcleritis, blepharitis, acute pancreatitis, etc.

The safety results of Merck KEYNOTE-028 study showed that the incidence of Grade 3 TRAEs was 17%, and no Grade 4 AEs, death, or treatment interruption due to AEs occurred; when JS001 (recombinant humanized PD-1 monoclonal antibody) produced by Junshi Biosciences in China was used for patients with advanced ESCC, the safety results showed that the incidence of all grades irAEs was 10.2%, and the incidence of Grade 3-5 TRAEs was 37.3%; when PD-1 inhibitor SHR-121 produced by Hengrui Medicine was used for advanced ESCC, the safety results showed that the incidence of Grade 3 TRAEs was 10%, and no Grade 4-5 TRAEs occurred.

1.3.3 Overall benefits: risk and ethical assessments

Currently, chemotherapy for advanced oesophageal cancer has encountered a bottleneck, both recurrence rate and metastasis rate are high, and overall outcome is poor. There is an urgent need to explore a treatment mode which can significantly improve the outcome of patients with oesophageal cancer. PD-1 inhibitors have become a new direction of treatment which can

improve the OS of the patients with advanced oesophageal cancer. Meanwhile, clinical studies at home and abroad showed that PD-1 inhibitors have survival benefit and controllable and tolerable safety. Based on the current first-in-human study data of HLX10, no dose-limiting toxicities were observed. The existing safety data and PK data proved an acceptable safety profile of HLX10 in patients. These data are adequate to support the conduct of a clinical study in this period.

2 STUDY OBJECTIVES

2.1 Primary Objective

To compare the clinical efficacy of HLX10 versus placebo in combination with chemotherapy as first-line therapy in patients with locally advanced/metastatic ESCC.

2.2 Secondary Objective

To compare the safety and tolerability of HLX10 versus placebo in combination with chemotherapy as first-line therapy in patients with locally advanced/metastatic ESCC.

3 STUDY PLAN

3.1 Overall Study Design

This study is a randomized, double-blind, multicentre, phase 3 clinical study to compare the clinical efficacy, safety, and tolerability of HLX10 versus placebo in combination with chemotherapy as first-line therapy in subjects with locally advanced/recurrent or metastatic ESCC, to collect PK parameters and explore the biomarkers related to efficacy.

3.1.1 Study type and duration

This study is a randomized, double-blind, multicentre, phase 3 clinical confirmatory study and includes three periods: screening period (28 days), treatment period (until loss of clinical benefits, death, unacceptable toxicity, withdrawal of informed consent, or other reasons specified in the protocol, whichever occurs first), and follow-up period (including safety follow-up period and survival follow-up period).

3.1.2 Randomisation and blinding

This study is designed as a randomized, double-blind trial. Eligible subjects will be randomized at a ratio of 2:1 into one of the following two groups using Interactive Web Response System/Interactive Voice Response System (IWRS/IVRS):

- Group A (HLX10): HLX10 + chemotherapy (cisplatin + 5-FU)
- Group B (control): placebo + chemotherapy (cisplatin + 5-FU)

Stratification factors at randomization include: expression level of PD-L1 (CPS < 10 versus CPS ≥ 10), age (≥ 65 years versus < 65 years), and tumor status (locally advanced versus distantly metastatic).

After screening, subjects meeting the inclusion criteria and not meeting the exclusion criteria will be enrolled into this study. Included subjects will receive the study drug until loss of clinical benefits, death, unacceptable toxicity, withdrawal of informed consent, or other reasons specified in the protocol, whichever occurs first.

The subjects, investigator, sponsor, and designee will not know the randomized assignments during study treatment, except for emergency unblinding. During the study treatment, if the investigator determines that any situation endangering the life of the subject is related to the study drug, emergency unblinding can be conducted when the investigator believes that awareness of the drug used by the subject is helpful for managing the AEs. The investigator will be responsible for the decision of emergency unblinding, and the sponsor will not delay or reject. However, the investigator may contact the sponsor or its designee before unblinding to discuss the unblinding and the treatment most helpful to the subject. The investigator should ensure that unblinding is conducted only in accordance with the protocol. The investigator should promptly notify the sponsor of emergency unblinding and reasons, and record them clearly on the subject's source document. The unblinding process will be completed on IWRS using personal identification number for emergency unblinding. If unblinding is needed, it can only happen on the concerned subject.

Overall unblinding will be conducted when the last subject completes end-of-treatment visit in this study. Blinding should be maintained unless there is medical emergency (emergency treatment is possible only when the randomized drug used for the subject is known) or unblinding is required by regulatory authority. All random codes can be unblinded when all data have been entered in the database, all data inquires have been solved and the subjects have been included in each analysis set.

3.2 Study Endpoints

3.2.1 Primary endpoint

- PFS (assessed by the IRRC as per RECIST v1.1)
- Overall survival (OS)

3.2.2 Secondary endpoints

- Progression-free survival (PFS) (assessed by the IRRC as per iRECIST, and assessed by the investigator respectively as per RECIST v1.1 and iRECIST)
- Objective response rate (ORR) (assessed by IRRC as per RECIST v1.1 and iRECIST respectively, and by the investigator as per RECIST v1.1 and iRECIST respectively)
- Relationship between expression of PD-L1 in tumor tissues and efficacy
- Duration of response (DOR) (assessed by IRRC as per RECIST v1.1 and iRECIST respectively, and by the investigator as per RECIST v1.1 and iRECIST respectively)
- Incidence of adverse events (AEs) and serious adverse events (SAEs)
- Pharmacokinetics (PK): Concentration of HLX10 in serum
- Immunogenicity evaluation: Positive rate of anti-drug antibody (ADA/NAb)
- Relationship between microsatellite instability (MSI) and tumor mutation burden (TMB) and efficacy
- Quality of life assessment

3.3 Number of Subjects

In this study, a total of about 540 subjects will be enrolled and at least 339 PFS events or 388 OS events have to be observed.

Group A (HLX10): 360 subjects

Group B (control): 180 subjects

3.4 Subject Eligibility

3.4.1 Inclusion criteria

Subjects must meet all of the following inclusion criteria to enter the study:

1. 18–75 years, male and female;
2. Histologically diagnosed with locally-advanced (determined by local investigator)/recurrent or distantly metastatic ESCC (including gastro-oesophageal junction) that is not resectable or cured by chemoradiotherapy (patient with adenosquamous carcinoma with predominantly squamous cell carcinoma can be enrolled);

3. Have not received any systemic anti-tumor therapy for current recurrence or metastasis. Exceptions: A patient who has received neoadjuvant/adjuvant therapy can be screened if his/her last neoadjuvant/adjuvant treatment is more than 6 months from relapse or PD; a patient who has received curative concurrent chemoradiotherapy or radiotherapy for oesophagus cancer can be screened if his/her last chemotherapy/radiotherapy is more than 6 months from relapse or PD. (Note: For radical concurrent chemoradiotherapy and neoadjuvant/adjuvant therapy (chemotherapy or chemoradiotherapy), any disease progression during treatment or within 6 months after discontinuation should be taken as a failure of first-line treatment, while any disease progression exceeding 6 months after discontinuation should not be taken as a failure of first-line treatment.) ;
4. At least one measurable lesion as assessed based on central imaging per Response Evaluation Criteria in Solid Tumors (RECIST v1.1) (cavity structures such as oesophagus cannot be considered as measurable lesions). The measurable lesion should not have received local treatment such as radiotherapy (if progression of a lesion located in prior radiotherapy region is confirmed, it can also be selected as a target lesion);
5. Subjects whose tumor samples are PD-L1 positive (CPS ≥ 1). The subjects must provide tumor tissue to measure the PD-L1 expression level;
6. ECOG within 7 days prior to the first dose of study drug: 0–1;
7. Expected survival ≥ 12 weeks;
8. Functions of major organs meet the following requirements (blood transfusion or use of any cell growth factors and/or thrombopoietic agents is not permitted within 14 days prior to the first dose of the study drug);
   a. Absolute neutrophil count (ANC) ≥ 1.5 × 10^9/L;
   b. Platelet count ≥ 100 × 10^9/L;
   c. Haemoglobin ≥ 9 g/dL;
   d. Serum albumin ≥ 3.0 g/dL;
   e. Total bilirubin ≤ 1.5 × ULN, ALT, AST and/or ALP ≤ 2.5 × ULN; if liver metastasis is present, ALT and/or AST ≤ 5 × ULN; if liver metastasis or bone metastasis is present, ALP ≤ 5 × ULN;
   f. Serum creatinine ≤ 1.5 × ULN or creatinine clearance ≥ 60 mL/min (calculated using Cockcroft-Gault formula);

    g. Activated partial thromboplastin time (APTT), international normalized ratio (INR) and prothrombin time (PT) ≤ 1.5 × ULN;

9. Female subjects of childbearing potential must have a negative blood pregnancy test within 7 days before the first dose. Female subjects of childbearing potential and male subjects having female partners in child-bearing period require at least one medically approved contraceptive method (such as intrauterine device, contraceptive drug, or condom) during the study, for at least 3 months after last dose of HLX10/placebo and for at least 6 months after last chemotherapy;
10. Subjects are willing to participate in this study and sign informed consent form, have good compliance and cooperate with the follow-up.

3.4.2 Exclusion criteria

Subjects who meet any one of the following criteria cannot enter this study:

1. BMI < 16.0 kg/m^2;
2. History of gastrointestinal perforation and/or fistulae within 6 months prior to the first dose of study drug;
3. Obvious invasion of tumor into organs (main artery or trachea) adjacent to oesophageal lesion, causing higher risk of bleeding or fistulae; history of stent implantation in the tracheal lumen;
4. Uncontrollable pleural effusion, pericardial effusion, or ascites requiring repeated drainage;
5. Prior allergic history to monoclonal antibodies, any ingredient of HLX10, 5-FU, cisplatin or other platinum drugs;
6. Have received any of the following treatment:
    a. Have received prior anti-PD-1 or anti-PD-L1 antibody treatment;
    b. Have received any investigational drug within 4 weeks prior to the first dose of study drug;
    c. Been enrolled in another clinical study at the same time, unless it is an observational (non-interventional) clinical study or in the interventional clinical study follow-up period;

d. Have received last cycle of anticancer treatment within ≤ 4 weeks prior to the first dose of study drug; palliative radiotherapy for bone metastasis lesion is allowed and should be finished 2 weeks prior to the first dose. Radiotherapy covering more than 30% of bone marrow area within 28 days prior to the first dose is not allowed;

e. Subjects who require to receive systemic treatment with corticosteroids (> 10 mg/day prednisone equivalent) or other immunosuppressive agents within 14 days prior to the first dose of study drug; inhaled or topical corticosteroids, and replacement with adrenocortical hormone at a dose ≤ 10 mg/day prednisone equivalent are permitted in the absence of active autoimmune disease;

f. Have received anti-tumor vaccine or have received live vaccine within 4 weeks prior to the first dose of study drug;

g. Have received major surgery within 28 days prior to the first dose of study drug; major surgery in this study is defined as: any surgery which requires at least 3 weeks of postoperative recovery time before receiving the study treatment. Patients with a history of tumor needle biopsy or lymph node incisional biopsy are included;

7. Toxicity from prior anti-tumor treatment not recovered to ≤ CTCAE Grade 1 (except alopecia) or the level specified in the inclusion criteria;

8. Patients with metastases to the central nervous system;

9. Active autoimmune diseases or history of autoimmune diseases (such as interstitial pneumonia, colitis, hepatitis, hypophysitis, vasculitis, nephritis, hyperthyroidism, and hypothyroidism, including but not limited to these diseases or syndromes); except patients with leucoderma or cured childhood asthma/allergy and not requiring any intervention in adulthood; autoimmune mediated hypothyroidism treated with stable-dose thyroid hormone replacement therapy; type I diabetes treated with a stable dose of insulin; subjects who are in a stable state and do not require systemic immunosuppressive therapy (including corticosteroids);

10. History of immune deficiency, including HIV antibody test positive, or history of other acquired or congenital immune deficiencies, or history of organ transplantation and allogeneic bone marrow transplantation;

11. Subjects have uncontrolled clinical heart and cardiovascular symptoms or diseases, including but not limited to: For example: (1) heart failure above NYHA Grade II; (2) unstable angina pectoris; (3) prior myocardial infarction and cerebral infarction within 6

months; (4) clinically significant supraventricular or ventricular arrhythmia without clinical intervention or inadequately controlled after clinical intervention;

12. Severe infection (CTCAE > grade 2) within 4 weeks prior to the first dose of study drug, such as severe pneumonia, bacteraemia, and infection complications requiring hospitalisation; active pulmonary inflammation accompanied with relevant clinical symptoms or signs based on chest X-ray at baseline; symptoms and signs of infection requiring oral or intravenous antibiotic therapy within 2 weeks prior to the first dose of study drug, except prophylactic use of antibiotics;

13. Subjects with prior and current interstitial pneumonia, pneumoconiosis, drug-related pneumonia, severely impaired lung function, etc. which may disturb the detection and treatment of suspected drug-related pulmonary toxicity; subjects with radiation pneumonia within 6 months;

14. Patients with active tuberculosis infection based on medical history or CT test, or patients with a history of active tuberculosis infection within 1 year before enrolment, or patients with active tuberculosis infection beyond 1 year but without proper treatment;

15. With HBsAg (+) and/or HBcAb (+), and HBV-DNA ≥ 500 IU/mL or 2500 copies/mL at the time of enrolment. Any subject with a measurement above the said criteria can only be enrolled if the measurement decreases to the normal range for at least 2 weeks after antiviral treatment, and he/she continues to receive the antiviral treatment throughout the study. Any subject who has previously needed to receive or were receiving antiviral treatment at the time of screening can only be enrolled if he/she continues to receive antiviral treatment throughout the study, even if HBV-DNA meets the inclusion criteria. Hepatitis C (HCV antibody tested positive and HCV-RNA positive);

16. Any other malignancies diagnosed within 5 years prior to the first dose of study drug, except malignancies having low risk of metastasis and death (5-year survival rate > 90%), such as adequately treated basal cell or squamous cell skin cancer, or carcinoma in situ of cervix uteri, and other carcinoma in situ;

17. Pregnant or lactating women;

18. Subjects having other factors possibly causing study discontinuation, as assessed by the investigator, such as other serious diseases (including mental disorders) requiring concomitant therapy, serious laboratory abnormalities, familial or social factors, or any circumstance potentially affecting subject safety or test data collection.

3.4.3 Withdrawal criteria

The subject can withdraw from this study at any time without any punishment or any impact on his/her future medical care.

The reasons why the subjects withdrew from the study included：

- Withdrawal of informed consent
- Loss to follow up
- Death

For subjects who have decided to withdraw their informed consent, if consents have been obtained from the subjects and/or their legal representatives, the investigator will collect information relevant to the follow-up, such as survival status, new anti-tumor treatment, etc.

After the discussion between the sponsor and the investigator, the subject can be asked to suspend or withdraw from the study due to the following reasons:

- AEs
- Inappropriate to continue with treatment as determined by the investigator
- Poor compliance, unable to return to visit on time
- Major protocol violations

The primary reason for withdrawing from the study will be recorded in the electronic data collection (EDC) systemin any circumstance. If the subject withdraws from the study early for any reason, the investigator should try to persuade the subject to receive a series of assessments specified for the end-of-treatment visit, and continue to follow the subject having unresolved or unstable AE until this AE is resolved, the subject is lost to follow-up or the investigator considers this AE to be stable or irreversible.

Under the premise of ensuring maximum personal interest of the subject, the sponsor, the regulatory authority, or the Independent Ethics Committee (IEC) may also ask the subject to discontinue the study treatment or withdraw from the study for the sake of safety. Possible reasons include, but are not limited to: No benefit from this study, AEs, major protocol violations, etc.

The withdrawals will not be replaced.

3.5 Early Study Termination/Site Closeout

Required by the investigator

The investigator clearly indicates he/she cannot continue the study. As required by Good Clinical Practice (GCP), the investigator should inform the subjects, the sponsor, the EC, and the NMPA and explain the reasons when he/she decides to early terminate the clinical study.

Required by the sponsor

The sponsor can require an early termination of the trial or early termination of the trial in a certain site for the following reasons:

1) Funding reasons;
2) Administrative reasons;
3) Required number of subjects has been enrolled;
4) The investigator cannot adhere to the study protocol, GCP, etc.;
5) The investigator cannot recruit enough number of subjects;
6) Efficacy and safety considerations.

As required by GCP, prior to terminating a clinical study, the sponsor should inform the investigator, the EC, and NMPA in writing and explain the reasons.

Required by competent authorities

According to the Provisions for Drug Registration, NMPA may order the sponsor to modify the clinical study protocol, suspend or early terminate the clinical study in any of the following cases during clinical study:

1) Failure of the EC to perform its duties;
2) Inability to ensure the safety of the subjects;
3) Failure to report SAEs within the specified time limit;
4) Evidence showing the clinical study drug is ineffective;
5) Quality problems of the clinical study drug;
6) Falsification in the clinical study;
7) Other GCP violations.

All study-related materials (except the documents that must be kept at the site) must be returned to the sponsor when the study is terminated early or the site is closed early. The investigator must preserve other documents until he/she is informed by the sponsor to destroy them.

Upon early discontinuation of study for any reason, the investigator should promptly notify the subject, give the subject appropriate treatment, and follow up.

4 STUDY TREATMENT

4.1 Study Drugs

Investigational product: HLX10

Name	Recombinant humanized anti-PD-1 monoclonal antibody injection Solution (HLX10)
Strength	100 mg (10 mL)/vial
Storage condition	2-8 °C, avoid light
Manufacturer	Shanghai Henlius Biopharmaceuticals Co., Ltd.
Supplier	Shanghai Henlius Biopharmaceuticals Co., Ltd.

Control: Placebo

Externally unrecognized injection containing no active ingredient of HLX10, provided by Shanghai Henlius Biotech, Inc.

Other study drugs

Commercial fluorouracil (5-FU) and cisplatin are centrally provided by Shanghai Henlius Biotech, Inc. For information on formulation, preparation, storage, and administration, refer to the prescription information of currently approved fluorouracil (5-FU) and cisplatin.

4.2 Administration Method and Dosage

The study drugs will be administered as follows: Every 2 weeks (14 days) equals to one cycle in the following regimen.

Sequence of the combination treatment: HLX10/placebo, cisplatin, and 5-fluorouraeil (5-FU) will be administered successively on Day 1.

Study drug: HLX10 or Placebo

3 mg/kg, intravenous infusion (iv), before infusion, it is recommended to filter with 0.2–5 µm in-tube filter; every 2 weeks (14 days) (Q2W), on Day 1 of each cycle, no reduction, and for up to 2 years.

Other study drugs: Combination chemotherapy

- Cisplatin: 50 mg/m^2, iv infusion, Q2W, on Day 1 of each cycle, and for up to 8 cycles.
- 5-fluorouraeil (5-FU): at a total dose of 2400 mg/m^2, continuous iv drip over 44–48 hours in each cycle, Q2W, and dose for less than or equal to 12 cycles.

Treatment with study drugs will continue until the loss of clinical benefit, PD, intolerable toxicity, discontinuation decided by the subject or physician, death, withdrawal of informed consent, pregnancy, non-compliance with protocol or procedure requirements, administrative reasons, or other reasons specified in the protocol (whichever occurs first). After 8 cycles of cisplatin and 12 cycles of 5-FU, the subject should not continue to use them even if the subject did not experience the above conditions. HLX10/placebo can be administered for up to 2 years.

4.3 Principles of Administration and Dosage Adjustment

4.3.1 General principles

- During Cycle 2 and every cycle afterwards, the haematology, hepatic and renal functions of the subjects within 3 days before each dose of the study drug must meet the following requirements:
  - Neutrophil count must be ≥ 1500/mm^3 or 1.5×10^9/L and platelet count must be ≥ 90,000/mm^3 or 90×10^9/L. Treatment should be delayed to provide sufficient recovery time if the above requirements are not met;
  - Hepatic and renal functions must meet the inclusion criteria;
- Chemotherapy is allowed to be suspended for no longer than 42 consecutive days (6 weeks), otherwise the chemotherapy will be discontinued; HLX10/placebo is allowed to be suspended for no longer than 12 consecutive weeks, otherwise the HLX10/placebo treatment will be discontinued. The therapy is required to be discontinued when continuous suspension of drug administration reaches the extent described above, except in the following conditions:
  - Treatments of adverse events result in continuous suspension of medication exceeding the above conditions. If the dose of the steroid hormone used is gradually decreased and

the HLX10/placebo administration interval is prolonged consequently, a continuous suspension of the drug for more than 12 weeks is allowed in this case.

- Interruption of medication longer than the above conditions for non-drug related reasons, such as continuous suspension of medication due to external uncontrollable factors (e.g., epidemic, traffic, and holidays), does not fall under the category of treatment termination. The administration can be resumed.

4.3.2 Principles for HLX10/placebo medication

Starting from the beginning of HLX10/placebo infusion, subjects shall be closely monitored for anaphylaxis that may occur within a few minutes. In case of mild symptoms (such as flushing or local skin reactions), drugs may be administered at a slower speed. For serious hypotension, bronchospasm, or generalised rash/erythema, the administration should be discontinued immediately and appropriate therapies should be given. For a life-threatening reaction, including anaphylaxis, hypersensitivity, renal failure, severe cardiopulmonary events, and severe skin reactions, the medications should be discontinued permanently.

In the event of HLX10/placebo-related toxicity, a delay in HLX10/placebo is allowed **rather than dose modification**. Subjects who miss a scheduled infusion for reasons other than HLX10/placebo-related toxicity should be actively contacted to arrange another visit with the least delay for administration.

4.3.3 Dose adjustment principle of chemotherapeutics (the following dose adjustment is provided to the investigator for reference)

In the event of intolerance to cisplatin and/or 5-FU, the doses of chemotherapy drugs may be modified simultaneously or separately. A 20% reduction in the starting dose may be made for the first occurrence of intolerance to Cisplatin, and the Cisplatin treatment may be discontinued if the investigator judges that the tolerance remains after the dose reduction. A 20% reduction in the starting dose of 5-FU is allowed for each dose, with a maximum of two dose reductions. Subjects requiring a third dose reduction should discontinue 5-FU treatment immediately.

Table 1. Dose adjustment standards for cisplatin and 5-FU

<table>
<tr><th>AE</th><th>Cisplatin</th><th>5-FU</th><th>Restart the treatment</th></tr>
<tr><td>Febrile neutropenia</td><td rowspan="3">One dose reductions are permitted, each by 20% of initial dose</td><td rowspan="3">Two dose reductions are permitted, each by 20% of initial dose</td><td>Reduce the dose after recovery</td></tr>
<tr><td>≥ Grade 3 neutropenia</td><td rowspan="2">Recovery to ≤ Grade 1, reduce the dose</td></tr>
<tr><td>≥ Grade 2 thrombocytopenia</td></tr>
<tr><td>Grade 1 renal toxicity</td><td rowspan="2">Reduction by 20% of initial dose</td><td rowspan="2">Reduction by 20% of initial dose</td><td>Reduce the dose</td></tr>
<tr><td>≥ Grade 3 non-haematological toxicity</td><td>Recovery to ≤ Grade 1, reduce the dose</td></tr>
<tr><td>≥ Grade 2 renal toxicity</td><td>Not applicable</td><td>Not applicable</td><td>Treatment discontinued</td></tr>
</table>

Permanent dose reduction should be performed in the event of first attack of neutropenia-induced fever.

If the dose needs to be adjusted for ANC and/or platelets, the investigator should decide dose adjustment or drug withdrawal according to the minimum value of ANC and/or platelets.

The investigator should notice and be alert to early and obvious signs of myelosuppression, infection, or neutropenia-induced fever so as to quickly and properly manage these complications. The subject must be reminded of the possibility of above signs and encouraged to seek medical advice as soon as possible.

In the event of nausea and/or vomiting, proper antiemetic should be used for control. If Grade 3 nausea/vomiting still occurs when antiemetic is used, the investigator should decide whether to reduce the dose after the event is resolved through symptomatic treatment.

4.4 HLX10-Related Toxicity and Management

If immunotherapy-related toxicity is observed in a subject, HLX10-based measures can be taken according to the following criteria, including permanent discontinuation, suspension, and continuation of HLX10 therapy.

Generally (except for hepatotoxicity), for any subject experiencing a moderate (grade 2) or severe (grade 3 or greater) immune-related adverse event (irAE), the investigational products should be suspended until symptoms or toxicities recover to grade 1 or less before re-administration. For patients reporting grade 2 irAEs, if symptoms do not improve within 1 week, corticosteroids (0.5 mg/kg/day prednisone or equivalent) should be given. In the event of grade 3 or greater irAEs, HLX10 therapy should be stopped immediately and the subject should be treated with a high dose of corticosteroid (1–2 mg/kg/day prednisone or equivalent). Gradually reduce corticosteroid dosage for at least 1 month until symptoms alleviate to grade 1 or below.

4.4.1 Cutaneous adverse events

Skin rashes and pruritus are the most commonly seen and earliest cutaneous AEs related to immunotherapies. The following table provides the medications and therapies in response to a cutaneous irAE:

Table 2. Management of cutaneous toxicity

Grade of rashes	Management	Follow-up
Grade 1–2:<br>Coverage ≤ 30% of body surface area (BSA)	Continue the treatment as per the protocol for grade 1 skin lesions.<br>For grade 2 skin lesions or symptomatic grade 1 skin lesions, delay the treatment as per the protocol.	**If the irAE persists for > 1 week or reoccurs, then**<br>• Consider biopsy.<br>• Start the treatment with methylprednisolone 0.5–1 mg/kg/day by i.v. or its equivalent by oral administration.<br>• Once the condition is improved, gradually reduce the dose of steroids for at least 1 month.<br>• Consider prophylactic antibiotics.<br>• If the steroid can be reduced to a prednisone-equivalent dose less than 10 mg/day, restart the treatment as per the protocol.<br>**If symptoms worsen:** Treat as an event of grade 3 or greater.
Grade 3–4:<br>Coverage ≥ 30% BSA;<br>life-threatening	• Stop the treatment as per the protocol.<br>• Consult the Dermatology Division.<br>• Consider biopsy.<br>• Start the treatment with methylprednisolone 1–2 mg/kg/day by i.v. or equivalent.	**If the condition is improved to grade 1:**<br>• Gradually reduce the dose of steroids for at least 1 month.<br>• Restart the treatment as per the protocol.<br>• Add prophylactic antibiotics.

4.4.2 Endocrine disorders

Immune checkpoint inhibitors tend to cause endocrine disorders. The most common endocrine disorders related to immune checkpoint inhibitors are autoimmune thyroid diseases, hypophysitis, and adrenal insufficiency. The following table provides the medications and therapies in response to an endocrine disorder:

Table 3. Management of endocrine disorders

Seriousness	Management	
Asymptomatic TSH elevations	Continue the treatment as per the protocol.<br>Consult an endocrinologist if TSH is < 0.5 × LLN or ≥ 2 × ULN, or beyond normal range in both of 2 subsequent measurements.	
Symptomatic endocrine disorders	Evaluate the endocrine function<br>Consider hypophysis scan<br>**Symptomatic endocrine disorders accompanied with abnormalities in laboratory data/hypophysis scan:**<br>• Delay the treatment as per the protocol<br>• Prednisone, 1-2 mg/kg/day, i.v. or p.o.<br>• Start appropriate hormone therapy<br>**No abnormalities in laboratory data/hypophysis scan:**<br>Repeat laboratory tests in 1–3 weeks/MRI in 1 month.	**If the condition is improved (with or without hormone replacement):**<br>• Gradually reduce the dose of steroids for more than 1 month and consider prophylactic antibiotics for opportunistic infections.<br>• Resume the treatment as per the protocol.<br>• For patients with adrenal insufficiency, corticosteroid maintenance therapy may be required.
Suspected adrenal crisis	• Stop the treatment as per the protocol<br>• Exclude septicaemia<br>• Intravenous pulse therapy with corticosteroids and mineralocorticoids<br>• Intravenous infusion<br>• Consult an endocrinologist<br>• If adrenal crisis is excluded, treat symptomatic endocrine disorders as above.	

4.4.3 Pneumonia

Pneumonia is an uncommon but potentially serious or fatal complication during treatment with immune checkpoint inhibitors. The following table provides the medications and therapies in response to an immune-mediated pneumonia:

Table 4. Management of pneumonia

CTCAE Grade	Management	Follow-up
Grade 1:<br>With radiographic changes only	• Consider delaying the treatment for 2–4 weeks<br>• Monitor the symptoms every 2–3 days<br>• Consider consulting the Division of Pulmonary Medicine and the Division of Infective Diseases	Repeat imaging at least every 3 weeks<br>**If symptoms or imaging results worsen:**<br>Treat as an event of grade 2 or greater
Grade 2:<br>Mild to moderate symptoms	• Delay the treatment<br>• Consult the Division of Pulmonary Medicine and the Division of Infective Diseases<br>• Monitor the symptoms daily and consider hospitalisation<br>• Start high-dose corticosteroid therapy (1–2 mg/kg/day)	Repeat imaging every 1–3 days<br>**If the symptoms improve:**<br>Gradually reduce the dose of steroids for more than 1 month, and resume the treatment after the toxicity reduces to grade 1 or less.

CTCAE Grade	Management	Follow-up
		**If the symptoms do not improve or even worsen after two weeks:**<br>Treat as an event of grade 3 or greater
Grade 3 or greater:<br>Severe symptoms; severe anoxia; life threatening	• Stop the treatment as per the protocol<br>• Hospitalisation<br>• Consult the Division of Pulmonary Medicine and the Division of Infective Diseases<br>• Start high-dose corticosteroid therapy (1–2 mg/kg/day)<br>• Add prophylactic antibiotics for opportunistic infections<br>• Consider bronchoscopy and lung biopsy	**If the symptoms improve to the baseline:**<br>Gradually reduce the dose of steroids for at least 6<br>weeks<br>**If the symptoms fail to improve or worsen in 48 h:**<br>Use additional immunosuppressants, such as infliximab, intravenously injectable immunoglobulin (IVIG), and mycophenolate mofetil

4.4.4 Colitis

Gastrointestinal AEs often occur in subjects receiving immune checkpoint inhibitor therapy. The most common AE is diarrhoea caused by immune-mediated colitis. The following table provides the medications and therapies in response to a gastrointestinal irAE:

Table 5. Management of gastrointestinal AEs

Grade of diarrhoea	Management	Follow-up
Grade 1:<br>Diarrhoea < 4 times/day, beyond the baseline; none<br>Symptomatic colitis	• Continue the treatment as per the protocol.<br>• Symptomatic therapy<br>• Change diet	Monitor the symptoms closely for worsening<br>Inform the patient and immediately report such worsening<br>**In the event of worsening:**<br>Treat as an event of grade 2 or greater
Grade 2:<br>Diarrhoea 4–6 times/day, beyond the baseline;<br>Colitis: abdominal pain and/or bloody stool	• Delay the treatment<br>• Symptomatic therapy<br>• Intravenous infusion support<br>• Consider hospitalisation for intravenous infusion and monitoring if symptoms worsen	**If the condition is improved to grade 1:**<br>Resume the treatment<br>**If the AE persists for ≥ 3–5 days or reoccurs:**<br>Start corticosteroid therapy (0.5–1 mg/kg/day)<br>**If symptoms improve to grade 1 after corticosteroid therapy:**<br>• Gradually reduce the dose of steroids for more than 1 month.<br>• Add prophylactic antibiotics for opportunistic infections.<br>**If the AE persists for > 3–5 days or worsens after the steroid therapy:**<br>Treat as an event of grade 3 or greater

Grade of diarrhoea	Management	Follow-up
Grade 3 or greater<br>Diarrhoea ≥ 7 times/day, beyond the baseline; incontinence<br>Colitis: serious abdominal pain, peritoneal irritation signs, bowel obstruction, pyrexia, perforation, etc.	• Stop the treatment as per the protocol.<br>• Start high-dose corticosteroid therapy (prednisone 1–2 mg/kg/day or equivalent)<br>• Hospitalisation<br>• Add prophylactic antibiotics<br>• Consider colonoscopy	**If the symptoms improve:**<br>Continue the treatment until grade ≤ 1, and then reduce the dose gradually for more than 1 month.<br>**If the AE persists for > 3–5 days or reoccurs:**<br>• Add infliximab or its equivalent (if it has no contraindication)<br>• Consider IVIG<br>• Avoid the use of infliximab in the case of perforation or septicaemia

4.4.5 Nephrotoxicity

Subjects received immunotherapy should be monitored for signs and symptoms of nephritis, glomerulonephritis, and renal insufficiency. Asymptomatic serum creatinine elevation is most likely to occur. Therefore, serum creatinine levels should be periodically monitored to detect nephrotoxicity. The following table provides the medications and therapies in response to nephrotoxicity:

Table 6. Management of renal adverse events

Grade of serum creatinine elevation	Management	Follow-up
Grade 1:<br>Creatinine ≥ ULN and ≥ baseline but ≤ 1.5 × ULN baseline	• Continue the treatment as per the protocol<br>• Monitor the serum creatinine every week	**If the symptoms recover to the baseline:**<br>Resume routine serum creatinine monitoring as per the protocol<br>**In the event of worsening:**<br>Treat as an event of grade 2 or greater
Grade 2–3:<br>Creatinine > 1.5 × baseline and ≤ 6 × ULN	• Delay the treatment as per the protocol<br>• Monitor the serum creatinine level every 2–3 days<br>• Start the treatment with methylprednisolone 0.5–1 mg/kg/day by i.v., or equivalent by oral administration	**If the symptoms recover to grade 1:**<br>• Gradually reduce the dose of steroids for more than 1 month<br>• Consider adding prophylactic antibiotics for opportunistic infections and resume routine serum creatinine monitoring as per the protocol<br>**If increased creatinine lasts for ≥ 5 days or worsens:**<br>Treat as an event of grade 4
Grade 4:<br>Creatinine ≥ 6 × ULN	• Stop the treatment as per the protocol<br>• Monitor the serum creatinine level daily<br>• Start treatment with methylprednisolone 1–2 mg/kg/day or its equivalent by i.v.<br>• Consult an nephrologist<br>• Consider renal biopsy	**If the symptoms recover to grade 1:**<br>Gradually reduce the steroid dose for at least 1 month and add prophylactic antibiotics

4.4.6 Hepatotoxicity

Hepatotoxicity is a rare irAE in immunotherapy. However, severe hepatotoxicity may lead to fatal hepatic failure. When hepatotoxicity is suspected, viral or other drug-induced hepatitis must be ruled out. The following table provides the medications and therapies in response to hepatotoxicity:

Table 7. Management of hepatotoxicity

**Grade of transaminase elevation**	**Management**	**Follow-up**
Grade 1:<br>AST or ALT ≥ ULN to 2.5 × ULN or total bilirubin ≥ ULN to 1.5 × ULN	Continue the treatment as per the protocol	Continue the weekly monitoring<br>**In the event of worsening:**<br>Treat as an event of grade 2 or greater
Grade 2:<br>AST or ALT ≥ 2.5 × ULN to ≤ 5 × ULN or total bilirubin ≥ 1.5 × ULN to 3 × ULN	Delay the treatment as per the protocol<br>Increase monitoring frequency to once every 3 days.	**If the symptoms recover to the baseline:**<br>Resume the routine monitoring and treatment as per the protocol.<br>**If AST or ALT increases for > 5–7 days or worsens:**<br>• Take prednisone 0.5–1.0 mg/kg/day or equivalent orally, and gradually reduce the dose of steroids for at least 1 month when the liver function recovers to grade 1 or baseline.<br>• Consider prophylactic antibiotics<br>• Resume the treatment as per the protocol
Grade 3–4:<br>AST or ALT ≥ 5 × ULN or total bilirubin ≥ 3 × ULN	• Permanently discontinue the treatment as per the protocol<br>• Monitor liver function tests (LFTs) every 1–2 days<br>• Start the treatment with methylprednisolone 1–2 mg/kg/day by i.v. or equivalent.<br>• Add prophylactic antibiotics<br>• Consult a gastroenterologist	**If the symptoms recover to grade ≤ 2:**<br>Gradually reduce the dose of steroids for at least 1 month.<br>**If the symptoms fail to improve or worsen or rebound in 3–5 days:**<br>• Add mycophenolate mofetil 500 mg–1 g, twice a day<br>• Consider using additional immunosuppressants<br>**Do not use infliximab for immunotherapy-related hepatitis.**

4.4.7 Neuropathy

A wide range of neurological syndromes are associated with immunotherapy, among which the Guillain-Barré syndrome is particularly significant. Other neurological complications include myasthenia gravis, posterior reversible encephalopathy syndrome, enteric neuropathy, aseptic meningitis, and autoimmune encephalitis. The following table provides the medications and therapies in response to neuropathy:

Table 8. Management of neuropathy

**Grade of neuropathy**	**Management**	**Follow-up**
Grade 1:<br>With no or mild symptoms	Continue the treatment as per the protocol	Continue the monitoring.<br>**In the event of worsening:**<br>Treat as an event of grade 2 or greater
Grade 2:<br>Moderate symptoms: limiting activities of daily living (ADL)	• Delay the treatment as per the protocol<br>• Symptomatic therapy<br>• Consider prednisone 0.5–1 mg/kg/day, i.v. or p.o.	**If the symptoms recover to the baseline:**<br>Resume the treatment as per the protocol<br>**If symptoms worsen:**<br>Treat as an event of grade 3 or greater
Grade 3–4:<br>Severe symptoms: limiting self-care ADL;<br>life-threatening	• Stop the treatment as per the protocol<br>• Consult a neurologist<br>• Symptomatic therapy<br>• Start the treatment with methylprednisolone 1–2 mg/kg/day i.v. or equivalent.<br>• Add prophylactic antibiotics	**If the symptoms are improved to grade 2:**<br>Gradually reduce the dose of steroids for at least 1 month.<br>**If the symptoms worsen or atypical symptoms appear:**<br>Consider IVIG or plasmapheresis or other immunosuppressive agents

4.4.8 Other immune-related adverse events

Other immune-related adverse events include cardiac toxicity, haemotoxicity, and oculopathy. All these adverse events are treated as detailed in Paragraph 2 of Section 4.4. Investigators should be highly vigilant for various immune-mediated manifestations. In general, timely treatment with corticosteroids usually has good outcomes and allows the subject to continue with the study.

4.5 Infusion-Related Reactions and Management

Acute infusion reactions (including cytokine release syndrome, angioedema, or anaphylaxis) are different from common anaphylaxis caused by drugs, although some of the characteristics are common to both. Infusion-related reactions often occur during or shortly after drug infusion, and generally relieve within 24 h after completion of the infusion. Symptoms and signs of infusion reactions include anaphylaxis/hypersensitivity, drug-induced fever, arthralgia, bronchospasm, cough, vertigo, and dyspnoea. Serious anaphylaxis may require epinephrine treatment. Infusion-related reactions should be graded according to the NCI CTCAE v4.03. The recommended treatments are listed in Table 9, but the final treatment is determined by the investigators based on their clinical experience.

Table 9. Management of acute infusion-related reactions

<table>
<tr><th>CTCAE Grade</th><th>Management</th><th>Post-infusion prophylactic drugs</th></tr>
<tr><td>Grade 1:
Mild reaction; transient reaction; infusion interruption not indicated; intervention not indicated</td><td>Strengthen monitoring of vital signs until the subject is considered medically stable by investigators.</td><td>None</td></tr>
<tr><td>Grade 2:
Require infusion interruption but responds promptly to symptomatic therapy (e.g., antihistamines, NSAIDs, narcotics, intravenous fluids); and prophylactic medications indicated for ≤ 24 h</td><td>• Stop the infusion and monitor the symptoms.
• Additional treatments, such as intravenous fluids, antihistamines, acetaminophen, or narcotics.
• If the symptoms subside within one hour after drug discontinuation, restart the infusion at 50% of the original infusion rate.
• If symptoms do not subside, continuous monitoring and hospitalisation for further treatment will be required.
• Although adequate prophylactic drugs have been administered, permanent discontinuation of relevant study drugs should be performed for the subjects experiencing grade 2 toxicity.</td><td>Prophylactic use of antihistamines (such as diphenhydramine) and acetaminophen, or determined according to the guidelines of the study site.</td></tr>
<tr><td>Grade 3:
Prolonged (e.g., not rapidly responsive to symptomatic medication and/or brief interruption of infusion); recurrence of symptoms following initial improvement; and hospitalisation indicated for other clinical sequelae
(e.g. kidney damage, pulmonary infiltrates)</td><td rowspan="2">• Stop infusion
• Additional treatment required: intravenous fluids, antihistamines, NSAIDs, acetaminophen, anaesthetics, oxygen therapy, vasopressors, glucocorticoids, and epinephrine.
• Hospitalisation for further treatment is required.
• The subject should immediately stop receiving treatment and permanently stop receiving the related study drug.</td><td rowspan="2">No subsequent infusion</td></tr>
<tr><td>Grade 4:
Life-threatening consequences; urgent intervention indicated</td></tr>
</table>

4.6 Drug Package and Label

The vials of HXL10, 5-FU, cisplatin, and placebo are labelled by a third party designated by the sponsor.

The label includes: drug name, drug number, protocol No., specifications and packaging, dosage and administration, manufacturing lot number, expiry date, storage requirements, and other specific information required by local regulations and Good Manufacturing Practice.

4.7 Drug Storage, Management and Dispensation

The study drugs will be dispensed by the sponsor or a third party designated by the sponsor. The sites shall establish complete drug receipt procedure and assign a person to receive the study drugs and sign the drug receipts. The study drugs can only be used for the trial designated by this protocol, and only authorized staff can have access to these drugs.

The sites shall establish a strict full-time drug management system to take charge of the storage and distribution of the study drugs, and establish a registration system. The sites shall make sure the storage conditions of the study drugs are satisfactory and record and store the study drugs.

Only the study personnel or his/her designee can provide drugs to the subjects and manage these drugs. Dispensation and recovery of the study drugs should be timely recorded in corresponding record sheet. Any loss, scattering, or misuse of the study drugs shall be recorded in detail.

The study drugs will be recovered and destructed by the sponsor or a third party designated by the sponsor, and shall not come into the market.

For detailed operations, refer to the drug management and operation process.

4.8 Concomitant and Prohibited Treatments

The investigator can, at his/her discretion, give all the drugs that he/she deems necessary for the health of the subjects and expected not to interfere with the assessment of the study drugs (i.e. best supportive therapy). Routine therapy against nausea and vomiting and other supportive therapies can be given to the subjects before and after administration of 5-FU and cisplatin according to local medical practice.

All concomitant medications (including start/end date, total daily dose, and indications) must be recorded in the corresponding parts of the subjects' original records and electronic case report forms (eCRF).

Prohibited medications/treatments

The following medications or treatments are prohibited during the study:

- Any other systemic chemotherapy, radiotherapy, immunotherapy, biotherapy, molecular targeted therapy, or immunomodulators (such as thymosin, lentinan, interleukin-12, etc.) having anti-tumor effects; traditional Chinese medicine, etc. that are labelled to have direct anti-tumor effect and/or immunomodulatory effect; local palliative therapy for isolated lesions (except target lesions) is acceptable (such as local surgery or radiotherapy for bone metastases);
- Any other clinical investigational products;
- Immunosuppressants, including but not limited to: systemic corticosteroids at a dose greater than 10 mg/day prednisone or equivalent, methotrexate, azathioprine, and TNF-α blockers. There are some exceptions:
  - ✓ Use of immunosuppressants to manage TRAEs
  - ✓ Short-term prophylactic medication to subjects expected to receive chemotherapy, provided that the prescribing information of the drug requires to use corticosteroids to patients with documented hypersensitivity.
  - ✓ Use in patients allergic to contrast media
  - ✓ In addition to this, inhaled, topical, and intranasal corticosteroids are allowed.
  - ✓ Short-term use of corticosteroids is allowed if clinically indicated and necessary for patient management (for example, used for chronic obstructive pulmonary disease, radiotherapy, nausea, etc.)
- Any attenuated live vaccines shall not be administered to the subjects during study treatment and within 30 days after last dose.
- For details of other medications prohibited in treatment of 5-FU and cisplatin, refer to the Instructions for Use of 5-FU and cisplatin.

Permitted medications/treatments

Medications/treatments permitted during the study include:

- Treatment or symptomatic treatment (including blood products, blood transfusion, infusion, antibiotics, anti-diarrhoeal drugs, etc.), excluding those expected to interfere with the

assessment of study treatment (or have interactions with study treatment), may be conducted for complications or AEs;

- For any suspected immune-related adverse reactions (irAE), HLX10/placebo may be replaced with corticosteroids, depending on the severity of adverse reactions. If there is symptom alleviation, it is recommended to gradually reduce the dose over a period of at least 1 month, until there is no drug to be given. If symptoms do not improve or are even worsen after use of corticosteroids, then non-corticosteroid immunosuppressive therapy should be added. HLX10/placebo will not be resumed when the subject is receiving an immunosuppressive dose of corticosteroids (prednisone over 10 mg/day or equivalents) or other immunosuppressive therapy.
- Antiemetics;
- Nutritional support;
- Drugs or treatments for pre-existing conditions.

4.9 Treatment Compliance

During the study and follow-up, medication information of the study treatment will be recorded in the CRF. Any use of medication not as specified in the protocol will be recorded in the CRF, including medication date and reasons. A clinical research associate (CRA) will review treatment compliance during study site visits and at the end of the study.

5 STUDY PROCEDURE AND VISITS

The study procedure and visits are outlined in the study schedule. All study results need to be recorded in the eCRF.

5.1 Study Procedure

5.1.1 Demographics and medical history

Demographic information includes date of birth, sex, ethnicity, etc.

Collection of oesophageal cancer history of the subjects must be completed at screening, including: clinical stage, pathological diagnosis, diagnostic method, date of diagnosis, lines of prior treatments (history of surgery, history of radiotherapy/chemotherapy, etc.), etc. Collection of personal history of the subjects, including: History of allergy, history of drug dependence, and tobacco use; In addition, it is necessary to collect all the history of anti-tumor surgery as well as

other important diseases and the history of non-tumor major surgery within one year before the subject signs the informed consent.

5.1.2 Prior and concomitant therapy

Prior medications taken from 30 days before screening visit to the time of signing ICF will be recorded and collected. Concomitant medications taken from the time of signing ICF to 30 days after the last study drug treatment will be recorded and collected. Concomitant therapies associated with AE are recorded to 90 days after the last dose. All prior and concomitant therapies (including traditional Chinese medicine products) are required to be documented in eCRF.

5.1.3 Adverse events

All AEs and SAEs are recorded since the subject's first administration of study drug (C1D1). AEs and SAEs are documented until 90 days after the completion of the study treatment or the initiation of a new anti-tumor treatment (whichever occurs first); and thereafter, only SAEs related to the study drug (HLX10/placebo) will be documented.

5.1.4 Quality of life assessment

Quality of life assessments, including EQ-5D-5L, EORTC QLQ-C30, and EORTC QLQ-OES18 assessments, will be performed during the study. The quality of life assessment within 7 days prior to the initial dose must be recorded during the screening period.

5.1.4.1 EQ-5D-5L

EQ-5D is a standardized instrument developed by EuroQol Group, which can be used to make a simple and general assessment of health conditions from the perspective of clinical and economic evaluations (EuroQol Group 1990). This scale is applicable to various health conditions and treatments. It lists simple descriptive features and gives single index values for health status. It can be used for clinical and economic evaluations of health care and population health survey. This questionnaire comprises the following five dimensions: mobility, self-care, usual activities, pain/discomfort, and anxiety/depression. Each dimension has 5 response levels reflecting increase in difficulty (no problems, slight problems, moderate problems, severe problems, and extreme problems) (EuroQol Group 2013).

Since 2009, EuroQol Group has developed one more sensitive EQ5D version (EQ-5D-5L) which enlarges the response range of each dimension, i.e. from 3 severity levels to 5 levels (Herdman et al 2011). Preliminary studies showed that the improvements of 5-level version in determining the properties of parameters when compared with 3-level version are as follows: reducing the ceiling

effect, increasing the reliability and enhancing the ability of distinguishing different health levels (Janssen et al 2008a, Janssen et al 2008b, Pickard et al 2007).

The subjects are asked to indicate his/her health status by choosing the most appropriate statement in each of the five dimensions. This questionnaire also comprises a visual analogue scale. The subjects are asked to score the current health status on a 0-100 scale, and 0 point indicates the poorest health status (see Appendix 3).

5.1.4.2 EORTC QLQ-C30

EORTC QLQ-C30 v3 is an established instrument to assess the health-related quality of life (HRQoL) and is usually used as an endpoint in clinical trials of tumors. This questionnaire assesses HRQoL/health status through 9 multi-item scales: 5 functional scales (physical, role, cognitive, emotional, and social), 3 symptom scales (fatigue, pain, nausea, and vomiting) and 1 global health status/QoL scale. 6 single-item measures include: dyspnoea, insomnia, appetite loss, constipation, diarrhoea, and financial problems (see Appendix 3). The total scores of each of these 15 fields are transformed, so that scores range from 0 to 100; a higher score represents a better level of functioning, a higher level of HRQoL or a higher level of symptoms (Aaronson et al 1993).

5.1.4.3 EORTC QLQ-OES18

EORTC QLQ-OES18 is an oesophageal cancer disease specific and treatment-related supplementary scale to the core questionnaire EORTC QLQ-C30, and contains 18 items in 4 fields for determining relevant symptoms and side effects of treatment: dysphagia, eating difficulties, reflux, and pain (see Appendix 3). The total scores of each field are transformed, so that scores range from 0 to 100; a higher score represents a better level of QoL.

5.1.5 Echocardiography

An echocardiogram examination must be performed for each subject at screening, and left ventricular ejection fraction will be recorded.

As assessed by the investigator, appropriate examinations should be performed in time for subjects who have developed shortness of breath, tachycardia, cough, jugular venous distention, hepatomegaly, and other clinical symptoms during the study.

5.1.6 12-Lead ECG

During screening, test results in 7 days prior to the first dose have to be documented. The 12-lead ECG should be performed for subjects after 5 minutes of rest before each examination. The ECG

test should be completed with results obtained before administration. If any clinically significant ECG abnormality occurs at a visit, a re-examination within 24 hours is recommended.

5.1.7 Complete physical examination

A complete physical examination must be performed at screening, including: head and neck (including thyroid), chest (including heart and lungs), abdomen (liver, bile duct, spleen, and kidney), four limbs, skin, lymph nodes, neurological systems, and general conditions, and the examination results should be recorded; special attention should be paid to the symptoms and signs of the respiratory system.

5.1.8 Symptom-directed physical examination

Symptom-directed physical examinations will be performed by the investigator based on clinical observation and symptoms during study treatment. As assessed by the investigator, new or worsened clinically significant physical examination abnormalities when compared with screening are recorded as AEs.During the first 2-3 days of each cycle, the investigator needs to closely monitor the subjects' general conditions, including dietary status and the presence of adverse reactions, such as nausea, vomiting, and diarrhoea, and give relevant symptomatic support treatments if necessary.

5.1.9 Height, weight, and vital signs

Height is only measured at screening. Vital signs including body temperature (°C), pulse rate (/min), respiratory rate (/min), and blood pressure (mmHg) are measured when the subject have rested for at least 5 minutes, and body weight is recorded.

Body weight and vital signs must be measured before each dose during study treatment. If the change of the subject's body weight from baseline (body weight before the first dose) is ≤ 10%, the dose modification of study drug is not necessary. If the change of body weight is >10%, the dose needs to be recalculated, and at this time, the new weight will be used as the baseline value for subsequent weight measurements.

5.1.10 ECOG score

It is recommended that ECOG performance status be evaluated by the same investigator throughout the study period. First ECOG assessment should be completed within 7 days prior to first dose.

5.1.11 Survival status

The subjects should be followed up for survival via telephone calls every 12 weeks ± 7 days before termination of all study drugs; the frequency of survival follow-up can be increased if appropriate.

5.1.12 Local laboratory tests

Local laboratory tests will be performed at the site's local laboratory. Haematology, serum chemistry, coagulation test, urinalysis, cardiac markers, pregnancy test, thyroid function test, trypsin, and virological test are included. Apart from virological and trypsin tests, other tests in the screening period should be performed within 7 days prior to the first dose. During the treatment, haematology, serum chemistry, coagulation function, cardiac markers, and urinalysis should be tested within 3 days prior to the dose of each cycle and pregnancy and thyroid function tests should be conducted within 3 days before dosing in every 2 cycles. When the above laboratory tests and study medication are scheduled on the same day, the results of such tests must be obtained before the scheduled study medication. Trypsin may be performed selectively based on the routine practice of the local study site.

(1) Haematology

Haematology test includes: red blood cell count, haemoglobin, platelet and white blood cell count, white blood cell count with differential and percentage (neutrophils, lymphocytes, basophils, eosinophils, and monocytes).

(2) Blood biochemistry

Blood biochemistry test includes: serum urea/urea nitrogen, creatinine, sodium, potassium, magnesium, chloride, bicarbonate/carbon dioxide combining power/total carbon dioxide (TCO2), calcium, phosphorus, blood glucose, total and direct bilirubin, alanine aminotransferase (ALT), aspartate aminotransferase (AST), alkaline phosphatase, lactate dehydrogenase, total cholesterol, total protein and albumin.

(3) Coagulation

Coagulation test includes: International Normalized Ratio (INR), Prothrombin Time (PT), and activated partial thromboplastin time (aPTT).

(4) Cardiac markers

Myocardial enzymes (creatine kinase (CK) and its isoenzymes (CK-MB)), troponin (TnI or TnT), and brain natriuretic peptide (BNP and/or NT-proBNP).

(5) Urinalysis

Urinalysis test includes: specific weight, pH, urine glucose, urine protein, urine casts, ketone body, and blood cell.

(6) Thyroid function

Thyroid function test includes: triiodothyronine (T3 or FT3), thyroxine (T4 or FT4), and thyroid-stimulating hormone (TSH).

(7) Trypsin

Trypsin (trypsin, serum amylase, and lipase) may be performed selectively based on routine practice of the local study site.

(8) Pregnancy test

To be enrolled in this study, women of childbearing potential must have a negative result in the blood pregnancy test performed within 7 days prior to the first dose (either blood or urine pregnancy test is acceptable; if urine pregnancy test is positive, then further bloods pregnancy test is needed).

(9) Virology examination

All subjects must be tested for HIV antibody, hepatitis B surface antigen (HBsAg)/core antibody (HBcAb), and hepatitis C virus (HCV) antibody at screening.

HBsAg or HBcAb positive subjects have to be analysed by HBV DNA titre, and subjects tested positive for anti-HCV antibody have to receive further HCV RNA assay. At screening (baseline), if: HBV DNA (< 500 IU/mL or 2500 copies/mL) and 1. HBsAg (+), or 2. HBcAb (+), the subject needs to be tested every 2 cycles during the treatment period for the five indicators of hepatitis B: 1). HBsAg (hepatitis B surface antigen); 2). HBsAb (hepatitis B surface antibody); 3). HBeAg (hepatitis B e antigen); 4). HBeAb (hepatitis B e antibody); 5). HbcAb (hepatitis B core antibody) and HBV DNA. If anti-HCV antibody (+) and HCV RNA (–) are present at baseline, anti-HCV antibody and HCV RNA have to be tested every 2 cycles during the treatment period.

5.1.13 Central laboratory assessments

HLX10-PK, ADA (Nab), and biomarkers (PD-L1, TMB, and MSI) should be tested by the central laboratory.

The tumor tissue sections and blood samples will be used for testing the expression level of PD-L1, MSI, and TMB.

ADA samples will be collected only prior to dosing, as detailed in the laboratory manual.

HLX10-PK and ADA(NAb) sample collection:

HLX10-PK and ADA (NAb) samples will be collected at the following time points: within 24 h before dosing of HLX10 at Cycles 1, 2, 4, 6, 8 and every 4 cycles afterwards, 0.5 hour after dosing of HLX10 at Cycles 1 and 8 (only for PK), at end-of-treatment visit and at safety follow-up.

Detection of PD-L1 expression:

PD-L1 expression is tested using the following two reagents for subject enrollment test described in "Section 5.1.15 Biomarker sample collection" and concomitant diagnosis study in "Section 8.4.6 Biomarker analyses".

Table 10. PD-L1 detection reagent

	Subject enrolment test	concomitant diagnosis study
Kit	Monoclonal Mouse Anti-Human PD-L1 Clone 22C3	PD-L1 antibody reagent<br>(kits of partners designated by the sponsor)
Manufacturer	Dako North America. Inc	-Qualified partners designated by the sponsor

See 5.1.15 for specific sample collection requirements for the PD-L1 detection for patient enrollment and concomitant diagnoses.

5.1.14 Radiological assessment of the tumor

CT or MRI test will be performed at screening, every 6 weeks (± 7 days) in the first 48 weeks after random, and every 12 weeks (± 7 days) after 48 weeks (test sites include neck, chest, abdomen, pelvis, and any other sites suspected of tumor lesion; MRI or CT of the brain (MRI is preferred) must be performed at baseline, and will be performed by the investigator as clinically indicated during treatment; bone scan will be performed by the investigator as clinically indicated at baseline and during treatment); the test method for the same site should be maintained as consistent as possible throughout the study; contrast should be used if there is no contraindication. The investigator and the IRRC will separately assess the radiological results per RECIST v1.1 (the frequency of tumor assessments can be increased by the investigator as clinically indicated). The investigator should determine subsequent treatment based on his/her

efficacy assessment. For subjects who continue with treatment after first PD (RECIST v1.1), radiological assessment needs to be conducted again after an interval of 4–8 weeks. If tumor assessment has been performed within 28 days prior to first dose in the same hospital using the same method and machine, then this tumor assessment can be taken as baseline tumor assessment. At the end-of-treatment visit, if radiological examination of the tumor has been performed within past 4 weeks, there is no need to take a re-test. Subjects who discontinue the treatment for reasons other than PD should continue the radiological assessments per schedule until PD, initiation of new anti-tumor therapy, withdrawal of informed consent, death, or study termination, whichever occurs first.

5.1.15 Sample collection for biomarkers

Subjects must provide formalin-fixed paraffin-embedded (FFPE) tumor samples (paraffin blocks or unstained slides) collected at non-radiotherapy sites and relevant pathological reports of these samples. In the absence of recent archival tumor tissue samples, a fresh biopsy of a tumor lesion at screening will be accepted in order to obtain the corresponding tumor samples (the number of samples depends on biopsy). The tumor tissue sections and blood samples will be used for testing the expression level of PD-L1, MSI, and TMB (where PD-L1 is required, while MSI and TMB are optional). Fresh sample collection, resection, core needle biopsy, and excisional, incisional, punch or forceps biopsies are all acceptable. Needle aspiration samples (i.e. samples lacking of complete tissue structure and only providing cell suspension and/or cell smear), brushing samples, and cell pellet samples from pleural or peritoneal effusion are not acceptable. See the Laboratory Manual for detailed requirements for tissue samples.

5.2 Screening (Day –28 to Day –1)

ICF must be signed and dated by the subject or legal representative in person prior to the commencement of study procedures.

The period of screening is 28 days. The data for ECOG performance status, pregnancy test, haematology, serum biochemistry, coagulation, urinalysis, thyroid function, and cardiac marker within 7 days prior to the first dose of study drug should be recorded, and the subjects can only be enrolled when these data meet the inclusion criteria and not meet any exclusion criteria.

The screening period begins when the subject signs and dates on the ICF, and ends when the subject is randomized or fails at the screening. Laboratory assessments to determine subject eligibility may be repeated during the screening before the subject is confirmed as a screening failure. Any subject who finally fails screening will not be eligible for the study, and has to be

registered in IVR/IWR system as screening failure; however, subjects who fail screening may be screened again as judged by the investigator and be assigned with new screening numbers.

Subjects are required to complete the following study procedures or assessments at screening:

1) Signing of ICF
2) Demographics and medical history
3) Prior and concomitant therapy
4) AEs
5) Quality of life assessment
6) Echocardiography
7) 12-lead ECG
8) Complete physical examination
9) Height, weight, and vital signs
10) ECOG score
11) Local laboratory tests

These include hematology, serum chemistry, coagulation, cardiac function markers, urinalysis, thyroid function (T3 or FT3, T4 or FT4, TSH), pregnancy test (if applicable), virology (hepatitis B surface antigen (HBsAg)/core antibody (HBcAb), hepatitis C virus (HCV) antibody, and HIV antibody test. HBsAg or HBcAb positive subjects have to be analyzed by HBV DNA titre, and subjects tested positive for anti-HCV antibody have to receive the HCV RNA assay). Trypsin may be tested selectively based on the routine practice of the local study site.

12) Central laboratory assessment

Including the acquisition of HLX10-PK/ADA (NAb) and tests of PD-L1, TMB, and MSI.

13) Radiological examination

Subjects are required to receive CT or MRI examination (including the brain, neck, chest, abdomen, pelvic cavity, and any other sites suspected of tumor lesions) at screening. If tumor assessment has been performed within 28 days prior to first dose in the same hospital using the same method and machine, then this tumor assessment can be taken as baseline tumor assessment.

14) Collection of tumor tissue sample (PD-L1, MSI, and TMB)

15) Collection of blood sample (MSI and TMB)

5.3 Treatment Period

Subjects will receive study treatment Q2W until loss of clinical benefit, death, intolerable toxicity, withdrawal of informed consent, or other reasons specified in the protocol, whichever occurs first. The following study procedures must be completed at each visit during the study:

1) Concomitant therapy
2) AEs
3) Quality of life assessment: once every 2 cycles after the first dose (pre-dose in cycles 3, 5, 7, etc.) until EOT.
4) 12-Lead ECG: should be completed with results obtained before each dose.
5) Symptom-oriented physical examination
6) Weight and vital signs
7) ECOG score
8) Survival status
9) Pregnancy test

During the treatment period, women of childbearing potential must receive a pregnancy test at the local site within 3 days prior to every two doses.

10) Laboratory tests
    - Haematology, blood biochemistry, coagulation, cardiac markers, and urinalysis must be performed at the local site within 3 days prior to each dose.
    - Thyroid function must be tested at the local site within 3 days prior to every 2 doses.
    - Trypsin may be performed selectively based on the routine practice of the local study site.
    - Virology tests: At screening (baseline), if: HBV DNA (< 500 IU/mL or 2500 copies/mL) and 1. HBsAg (+), or 2. HBcAb (+), the subject needs to be tested every 2 cycles during the treatment period for the five indicators of hepatitis B: 1). HBsAg; 2).

HBsAb; 3). HBeAg; 4). HBeAb; 5). HbcAb and HBV DNA. If anti-HCV antibody (+) and HCV RNA (–) are present at baseline, anti-HCV antibody and HCV RNA have to be tested every 2 cycles during the treatment period.

11) Central laboratory assessment

HLX10-PK/ADA (NAb) collection should be performed.

12) Radiological examination

- CT or MRI test will be performed at screening, every 6 weeks (± 7 days) in the first 48 weeks after random, and every 12 weeks (± 7 days) after 48 weeks (test sites include neck, chest, abdomen, pelvis, and any other sites suspected of tumor lesion; MRI or CT of the brain (MRI is preferred) must be performed at baseline, and will be performed by the investigator as clinically indicated during treatment; bone scan will be performed by the investigator as clinically indicated at baseline and during treatment); the test method for the same site should be maintained as consistent as possible throughout the study; contrast should be used if there is no contraindication. The investigator and the IRRC will separately assess the radiological results per RECIST v1.1 (the frequency of tumor assessments can be increased by the investigator as clinically indicated). The investigator should determine subsequent treatment based on his/her efficacy assessment. For subjects who continue with treatment after first PD (RECIST v1.1), radiological assessment needs to be conducted again after an interval of 4–8 weeks. If tumor assessment has been performed within 28 days prior to first dose in the same hospital using the same method and machine, then this tumor assessment can be taken as baseline tumor assessment. At the end-of-treatment visit, if radiological examination of the tumor has been performed within past 4 weeks, there is no need to take a re-test. Subjects who discontinue the treatment for reasons other than PD should continue the radiological assessments per schedule until PD (including the PD determined by RECIST v1.1 and that by iRECIST, if any), initiation of new anti-tumor therapy, withdrawal of informed consent, death, or study termination, whichever occurs first.
- During the study, if the subject decides to continue the treatment after the first PD (RECIST v1.1), **the subject must re-sign the ICF and** meet the following criteria:

  a) Absence of clinical symptoms and signs of significant disease progression (including worsening laboratory results).

  b) Stable ECOG performance status.

c) Absence of rapid progression of disease or of tumor progression at critical anatomical sites (e.g., spinal cord compression) that necessitates urgent alternative medical intervention.

d) Satisfaction of corresponding laboratory test parameters in inclusion criteria of this study (i.e., meet the inclusion criterion 8)..

- If a subject has developed PD (first PD; per RECIST v1.1), the next stage of treatment is as follows:

➢ Group A: The investigator will comprehensively judge whether the subject is suitable for continuing with the original treatment regimen according to the actual situation of the subject; if suitable, the subject can choose to continue with the original treatment regimen after signing the ICF again, and may receive another radiological examination at an interval of 4–8 weeks. If PD (iRECIST) is confirmed through subsequent radiological examination, the subject will be off to the follow-up period;

➢ Group B: The investigator will comprehensively judge whether the subject is suitable for continuing with the original treatment regimen according to the actual situation of the subject; if suitable, the subject can choose to continue with the original treatment regimen after signing the ICF again, and may receive another radiological examination at an interval of 4–8 weeks. If PD (iRECIST) is confirmed through subsequent radiological examination, the subject will be off to the follow-up period.

5.4 End-of-Treatment Visit

For a subject who discontinues the study treatment for any reason, an EOT visit should be performed whenever possible. If the subject has completed the laboratory tests specified in the study flow chart within 7 days (including day 7) prior to the EOT visit, repeated tests are not required. No safety follow-up is required if the discontinuation occurs 30 days (± 7 days) following the last dose of study drug. The following information should be collected by the investigator:

1) Concomitant therapy

2) AEs

3) Quality of life assessment

If no assessment has been performed within past 6 weeks at end-of-treatment visit, one quality of life assessment needs to be performed at this visit.

4) 12-lead ECG

5) Symptom-oriented physical examination;

6) Weight and vital signs

7) ECOG score

8) Survival status

9) Laboratory tests

Including haematology, blood biochemistry, coagulation function, cardiac markers, urinalysis, thyroid function, pregnancy test (if applicable), and virological tests (if necessary). Trypsin may be performed selectively based on the routine practice of the local study site.

10) Central laboratory assessment

Including collection of HLX10-PK/ADA (NAb).

11) Radiological assessment of the tumor

At the end-of-treatment visit, if radiological examination of the tumor has been performed within past 4 weeks, there is no need to take a re-test.

5.5 Follow-Up Period

After the EOT visit, subjects will be followed up.

Subjects who discontinue the treatment for reasons other than PD should continue the radiological assessments per schedule until PD, initiation of new anti-tumor therapy, withdrawal of informed consent, death, or study termination, whichever occurs first.

5.5.1 Safety follow-up

Safety follow-up at the study site is required 30 days (± 7 days) following the last dose of study drug. Prior to the initiation of new anti-tumor therapy, patients are required to receive a safety follow-up by telephone 90 ± 7 days after the last dose, and only the information of AE and AE-related concomitant medications will be collected. No safety follow-up is required if the end-of-treatment visit occurs 30 days (± 7 days) following the last dose of study drug.

1) Concomitant therapy

2) AEs

3) 12-lead ECG

4) Symptom-oriented physical examination

5) Weight and vital signs

6) ECOG score

7) Subsequent anti-tumor therapy

8) Survival status

9) Laboratory tests

10) Including haematology, blood biochemistry, coagulation function, cardiac markers, urinalysis, thyroid function, pregnancy test (if applicable), and virological tests (if necessary). Trypsin may be performed selectively based on the routine practice of the local study site.

11) Central laboratory assessment

Including collection of HLX10-PK/ADA (NAb).

12) Radiological assessment of the tumor

Subjects who discontinue the treatment for reasons other than PD should continue the radiological assessments per schedule until PD, initiation of new anti-tumor therapy, withdrawal of informed consent, death, or study termination, whichever occurs first.

5.5.2 Survival follow-up

During survival follow-up, the subjects will be followed for survival only by telephone every 12 weeks ± 7 days; the investigator may increase the frequency of survival follow-up as appropriate. The following assessments will be completed at each follow-up:

1) Documentation of survival status;

2) 2) Documenting subsequent anti-tumor therapies.

3) Radiological assessment of the tumor (except for subjects with disease progression or new anti-tumor therapy)

Subjects who discontinue the treatment for reasons other than PD should continue the radiological assessments per schedule until PD, initiation of new anti-tumor therapy, withdrawal of informed consent, death or study termination, whichever occurs first.

6 STUDY ASSESSMENT

6.1 Efficacy Assessment

In addition to the OS, all the other efficacy endpoints will be based on the tumor response assessment per RECIST v1.1 and iRECIST. Tumor assessment will be performed by qualified personnel at the site and the IRRC. Treatment decisions will be based on the investigator's assessment of tumor response and results will be reported in the eCRF.

Tumor assessment should be performed as scheduled, which is not influenced by treatment interruption or any other events leading to imbalance in disease assessment time between treatment groups.

6.2 Safety Assessment

Safety assessment includes monitoring and recording all AEs (including SAEs), laboratory tests (haematology, blood biochemistry, coagulation, urinalysis, thyroid function, cardiac markers, and tyrisin) 12-lead ECGs, ECGs, ECOG performance status score, vital signs, physical examination, etc.

7 ADVERSE EVENT

7.1 Definition of Adverse Event

AE is defined as any untoward medical occurrence in a patient or clinical study subject administered a pharmaceutical product. An AE does not necessarily have a causal relationship with the treatment. AEs therefore include any unfavourable and unintended signs (including an abnormal test, finding), symptoms or diseases temporally associated with the use of an (investigational) product (IP), regardless of the relatedness to the IP (as defined by ICH).

AEs may include new events, worsening or increased frequency of underlying diseases/conditions/signs, and abnormal results including abnormal laboratory values.

Examples of AEs include, but not limited to:

- Abnormal laboratory results;
- Clinically significant symptoms and signs;
- Changes in physical examination findings;
- Hypersensitivity.

In addition, symptoms or signs resulted from the following events are also included:

- Overdose;
- Drug discontinuation;
- Drug abuse;
- Drug misuse;
- Drug interaction;
- Drug dependence;
- Drug spillover;
- Pregnancy

Abnormal test results should be judged as AEs based on the following criteria:

- The test results are accompanied with symptoms, and (or);
- The test results necessitates drug/surgery intervention, and (or);
- The test results lead to study drug adjustments (beyond the dose adjustments specified in the protocol) or;
- The investigator or sponsor considers that the test results constitute an AE.

If any of the above criteria is not met, repeating the abnormal test alone does not constitute an AE. It is unnecessary to report any incorrectly reported finding, at the investigator's discretion, as an AE.

7.2 Adverse Event of Special Interest (AESI)

AESIs are events of scientific and medical interests in understanding the study drug and may require close monitoring and prompt contact with the sponsor by the investigator. AESIs can be serious or non-serious. Expedited reporting enables continuous monitoring of AESIs in order to describe and understand their association with use of the study drug.

AESI in this study includes infusion-related reaction (IRR) and immune-related adverse event (irAE).

irAEs are defined as AEs that are associated with drug exposure and demonstrate immune-mediated mechanisms with no other unequivocal etiology. Serological, immunological,

and histological (biopsy) data should be used to support the diagnosis of irAEs where appropriate. Appropriate methods should be used to exclude tumors, infections, metabolism, toxins, or other causes of irAEs. More description about the assessment and treatment for irAEs is detailed in "Investigator's Brochure" and Section "4.4 HLX10-Related Toxicity and Management".

For suspected irAEs, the function of the relevant systems should be closely observed and adequate evaluation should be performed to confirm etiology and exclude other causes. Overall, HLX10 should be suspended or permanently discontinued and/or symptomatic treatment, such as glucocorticoids, should be given depending on the severity of events. If AEs are not improved or worsen after glucocorticoid therapy, consideration may be given to increasing the dose of glucocorticoid and/or using other systemic immunosuppressant therapies. When the AE is ≤ Grade 1, the dose of glucocorticoid can be gradually reduced and the treatment should be continued for at least 1 month. HLX10 infusion therapy can be continued when the AE recovers to ≤ Grade 1 and the glucocorticoid dose is reduced to prednisone ≤ 10 mg/day (or equivalent). When a ≥ Grade 3 irAE occurs again (except for endocrine system disorders), the subject should immediately and permanently discontinue the drug and withdraw from the study.

AESI that meets the SAE criteria should be handled in accordance with the relevant procedures of SAE reporting. Despite not meeting the criteria of SAEs (i.e., the submission to the relevant regulatory and ethics department is not required), the following AEs are required to be reported to the sponsor within 24 hours (AESI):

- ≥ grade 2 of colitis, uveitis, interstitial pneumonia, and myocarditis;
- ≥ grade 3 of other immune-related adverse events (excluding immune-related hypothyroidism).

7.3 Definition of Serious Adverse Event

SAEs are defined as any untoward medical occurrence that:

- Leads to death;
- Is life-threatening (an event in which the subject is at risk of death at the time of the event. It does not refer to an event, which hypothetically might cause death, if it is more severe.);
- Requires hospitalisation or prolonged hospitalisation (it is necessary to make clear that the cause of this condition is due to AEs rather than selective surgery and non-medical reasons as described below);

- Leads to persistent or significant disability/incapacity;
- Results in congenital anomaly or birth defect (the offspring of the subject has congenital anomaly or birth defect);
- Leads to other medically important events.

These medically important events may not be immediately life-threatening or result in death, but may be considered as SAEs when they may jeopardize the subject and may require intervention measures to prevent the outcomes listed above through sufficient medical and scientific judgment. Examples of such medical events may include allergic bronchospasm needing intensive treatment in an emergency room or at home, blood dyscrasia or convulsions that do not result in hospitalisation, or development of drug dependence or drug abuse. If the investigator assesses that the clinical signs and symptoms of an AE have a significant clinical impact, the AE is regarded as a SAE. Medically significant events assessed based on medical data and investigator's clinical experience should be handled with reference to expedited reporting of SAEs. In addition, any transmission of suspected infectious substances that may be transmitted through drugs is considered a medically significant event.

Hospitalisation

AEs during the study that lead to hospitalisation or prolonged hospitalisation are regarded as SAEs. Any new hospitalisation to a medical facility (even if less than 24 hours) meets this criteria. Hospitalisation also includes in-hospital transfers to emergency/intensive care units (e.g. from paediatrics to internal medicine, from internal medicine to intensive care unit of coronary heart disease, from neurology to tuberculosis ward).

Hospitalisation in the following facilities is not included:

- Rehabilitation facility;
- Hospice care facility;
- Short-term care facilities (e.g. nursing care);
- Skilled nursing facility;
- Nursing home;
- Observation in routine emergency department;
- Day surgery (outpatient/daytime surgery/daytime operation).

Hospitalisation or prolonged hospitalization due to the following reasons is not regarded a SAE:

- Hospitalisation or prolonged hospitalization not related to any AE or worsening of any original adverse disease (for example, the inspection of persistent laboratory outliers existing prior to treatment);
- Hospitalisation for non-medical reasons (e.g. homeless subject);
- Hospitalisation for administrative reasons (e.g., routine annual checkup);
- Study-specific hospitalisation as defined in the protocol (e.g., operations required to conduct the protocol);
- Selective hospitalisation not related to the deterioration of AE (e.g. for plastic surgery);
- Hospitalisation or surgery scheduled by the subject;

Diagnostic and therapeutic noninvasive and invasive procedures, such as surgery, should not be reported as AEs. However, diseases treated by such procedures should be reported as AEs if they meet the definition of AEs. For example, acute appendicitis that occurs during the reporting period of an AE should be reported as an AE, and appendectomy for the treatment of the disease should be documented as treatment for the AE.

7.4 Documentation of Adverse Events

Data should be collected and documented for all AEs spontaneously reported by subjects, or AEs answered by subjects after the investigator puts forward the open question of "Have you had any health problems since the last visit/questioning to you?", or AEs discovered through observation. When AE data are collected, it is best to document the diagnosis (if possible), rather than a series of signs and symptoms. However, if the diagnosis is known but the patient still has other symptoms or signs that are not fall within the diagnosis, each symptom or sign should be documented separately.

7.4.1 Time of gathering adverse event information

All AEs, as well as SAEs, are to be collected and recorded from the first dose of investigational product (C1D1) to the subject, until 90 days after the last dose or the start of new anti-tumor treatment (whichever occurs first), after which only SAEs related to the investigational product (HLX10/placebo) are recorded.

Note: If, after a subject has completed safety follow-up, the investigator becomes aware of any SAE (including death) in the subject and suspects that this event is related to the investigational

product (HLX10/placebo), the investigator should report it to the sponsor's pharmacovigilance (PV) team or its representative. For SAEs that occurred after ICF signature and before the first dose of investigational product, only SAEs (within 24 hours of being informed) resulting from protocol-mandated interventions (e.g., invasive manipulations and biopsies) are to be reported.

7.4.2 Follow-up of unresolved adverse events

All AEs and SAEs of each subject should be actively followed up throughout the study. Even if the event is ongoing after end of treatment or study, it should be followed by the investigator as possible until all events meet any of the followings:

- The events return to the baseline level;
- The events are stable (the investigator does not expect any further improvement or worsening of the AEs);
- No more information is available (the subject refuses to provide further information or evidence shows that the subject is still lost to follow-up after the greatest possible effort has been made).

The sponsor reserves the right to request additional information (if necessary) about the ongoing AE/SAE from any subject at the end of the study.

7.4.3 Information collection

The following information will be collected for each AE:

- AE (verbatim record)
- AE start and end dates
- Changes in CTCAE grade
- Whether it is an SAE
- The causal relationship between the AE and the study drug evaluated by the investigator
- Action taken with the study drug
- Treatment measures for AE
- Outcome

In addition, the following information will be collected for each SAE:

- Date on which AE meets the SAE criteria
- Date on which the investigator is aware of SAE
- Reasons for being judged as SAE
- Discharge date (if applicable)
- Possible cause of death (if applicable)
- Date of death (if applicable)
- Autopsy results (if applicable)
- Assessment of causality between the SAE and study drug/study operation
- Assessment of causal relationship with other drugs
- AE narrative

7.4.4 Severity evaluation

Note that serious AE and severe AE should be distinguished. An AE that is severe in severity is not necessarily a SAE. For example, vomiting that lasts for several hours can be considered as severe, but it is not a SAE. On the other hand, a stroke that causes only mild dysfunction may be considered as mild, but it is a SAE.

The investigator shall assess the severity of each AE and SAE reported during the study. AEs and SAEs should be assessed and graded based upon NCI CTCAE v4.03. There are Grades 1–5.

Grade	CTCAE Narrative
1	Mild: asymptomatic or mild symptoms; clinical or diagnostic observations only; intervention not indicated.
2	Moderate: minimal, local, or noninvasive intervention indicated; limiting age-appropriate instrumental activities of daily living (ADL)*.
3	Severe or medically significant but not immediately life-threatening: hospitalisation or prolonged hospitalisation indicated; disabling; limiting self-care ADL**.
4	Life-threatening consequences: urgent intervention indicated.
5	Death related to AE

CTCAE = Common Terminology Criteria for Adverse Events
*： Instrumental ADL refers to preparing meals, shopping for groceries or clothes, using the telephone, managing money, etc.
**： Self-care ADL refers to bathing, dressing and undressing, feeding self, using the toilet, taking medications, and not bedridden.
Note that the severity and intensity of AEs should be differentiated. Severe intensity does not necessarily indicate a SAE. For example, a headache may be severe in intensity but not listed as a SAE unless it meets the SAE criteria.

7.4.5 Causality assessment

The investigator must provide a causality assessment of all AEs (both serious and non-serious). The investigator is required to record causal relationships, i.e., "definitely related", "possibly related", "unlikely related", "not related", and "not determinable". "Definitely related", "possibly related", and "not determinable" will be considered a causal relationship between the AE and the drug, i.e., **adverse drug reactions**.

- Definitely related:
  - Reasonable temporal relation +
  - Consistent with the known types of adverse reaction +
  - The AE is resolved or relieved after discontinuation or dosage reduction of the study drug +
  - The AE recurs when the study drug is resumed +
  - There are other reasonable explanations -
- Possibly related:
  - Reasonable temporal relation +
  - Consistent with the known types of adverse reaction ±?
  - The AE is resolved or relieved after discontinuation or dosage reduction of the study drug ±?
  - The AE recurs when the study drug is resumed?
  - Possibility of AEs due to other reasons ±?
- Unlikely related:
  - Reasonable temporal relation -
  - Consistent with the known types of adverse reaction -
  - The AE is resolved or relieved after discontinuation or dosage reduction of the study drug ±?
  - The AE recurs when the study drug is resumed?
  - Possibility of AEs due to other reasons ±?

- Not related:
  - - Reasonable temporal relation -
  - Consistent with the known types of adverse reaction -
  - The AE is resolved or relieved after discontinuation or dosage reduction of the study drug -
  - The AE recurs when the study drug is resumed -
  - Possibility of AEs due to other reasons +
- Not determinable: Essential information for the evaluation is not available

Determination of the Relationship between AEs and IP

	**Definitely related**	**Possibly related**	**Unlikely related**	**Not related**	**Not determinable**
Have a reasonable temporal relationship with the investigational product	+	+	-	-	Essential information for the evaluation is not available
Known types of drug reaction	+	±?	-	-	
Reaction is resolved or relieved after drug discontinuation	+	±?	±?	-	
Reaction recurs when the drug is resumed	+	?	?	-	
Possibility of AEs due to other reasons	-	±?	±?	+	

Note: + Yes – No ± Likely ? Unknown

7.4.6 Expectedness assessment

Unexpected AEs refer to events in which the nature or severity is not consistent with the corresponding drug Reference Safety Information (RSI). For IPs, the expectedness of AEs will be based on the listedness in the Investigator's Brochure. For control drugs that have been approved for marketing, the expectedness of AEs will be based on the listedness in the package inserts.

7.4.7 Progressive disease

PD refers to the deterioration of a subject's condition caused by the disease under study. Deterioration of symptoms and/or signs associated with PD should not be recorded as an AE. Disease progression leading to hospitalization/death does not need to be reported as an SAE.

7.4.8 Newly developed primary tumor

As regards newly developed primary tumor, the investigator should report to the sponsor within 24 h after being informed. Newly developed primary tumors refer to tumors found after the subject has signed the informed consent form and that are not primarily caused by the investigational product.

7.4.9 Death

All deaths that occurred during the study period or within 90 days after the last dose of investigational product under the study protocol or prior to the start of new anti-tumor treatment (whichever occurs first), and all deaths that occurred 90 days after the last dose of investigational product or after the start of new anti-tumor treatment (whichever occurs first) and are suspected to be related to the investigational product (HLX10/placebo) should be reported according to the following requirements:

- In the event of death (not including one due to PD), the AE that causes the death should be reported as an SAE to the CRA, sponsor or its representative within 24 hours, and the leading cause of death should be provided.
- Deaths unexplained should be reported as "Unknown cause of death" as SAE. The cause of death will be further explained during follow-up. Autopsy may help assess the cause of death. If an autopsy is performed, the autopsy report should be provided to the sponsor's pharmacovigilance team or its representative as soon as possible.

7.4.10 Drug-induced liver injury (DILI)

All cases with abnormal liver function test (LFT) results, confirmed to meet the criteria described below and with no other explainable cause, should be considered as potential Hy's Law cases whether an exact etiology for the abnormal LFT has been obtained or not. Events that meet the following criteria (Table 11) should be reported to the sponsor within 24 h.

Table 11. Abnormal liver function test results that should be reported

**Baseline**	**AST or ALT and total bilirubin at baseline within the normal range**	**AST or ALT or total bilirubin above the upper limit of normal values**
Treatment period	ALT or AST ≥ 3 × upper limit of normal (ULN) values accompanied by the following three conditions **in the meantime**:<br>1) Total bilirubin ≥ 2 × ULN<br>2) Alkaline phosphatase ≤ 2 × ULN or unknown<br>3) Free of hemolysis	AST or ALT ≥ 2 × baseline value and ≥ 3 × ULN; or AST or ALT ≥ 8 × ULN (whichever is smaller) **accompanied by** the following three conditions:<br>1) Total bilirubin ≥ 2 × ULN, with an increase of 1 × ULN or > 3 × ULN of the upper limit of normal values (whichever is smaller) compared with that at baseline<br>2) Alkaline phosphatase ≤ 2 × ULN or unknown<br>3) Free of hemolysis

7.5 Reporting of AEs

7.5.1 Reporting requirements for serious adverse events

For all SAEs that occur during the clinical study, the study personnel must report them to the sponsor or designated representative immediately (within 24 hours of awareness). This time requirement also applies to additional information (follow-up information) for previously issued reports of SAEs and initial report and follow-up report for pregnancy cases. The sponsor's representative will work with the investigator to ensure that all necessary information is submitted within the time frame described above.

The investigator is obliged to obtain and provide information of all SAEs to the sponsor within the reporting time frame mentioned above. In addition, the sponsor may request the investigator for more follow-up information rapidly. Such information may be more detailed than that shown on the AE report form. In general, it shall include a description of the AE in sufficient detail to allow for a complete medical assessment of the case and independent assessment of possible causality. In addition, information on other possible causes of AEs, such as concurrent medications and complications, should also be provided by the investigator.

In the event of death of a subject, a summary of autopsy results (if any) must be submitted to the sponsor or designated representative as soon as possible.

A hard copy of completed and signed SAE report should be sent by the investigators to the sponsor and the designated representative by fax/mail. In rare cases, if no fax device is available, a telephone notification is acceptable, followed by a paper copy of the SAE by mail. After giving the notification first by telephone, the investigator is still required to fill out and sign the paper SAE form within 24 hours after awareness of the event.

Contact information to assist the sites in SAE reporting is detailed in the investigator documentation provided to the sites. The original SAE report form and fax confirmation page (if fax is used) must be kept by the site.

The sponsor or its representative should submit the drug safety report as required by regulatory authorities and regulations.

The investigator or any person-in-charge required by a local authority should abide by each regulatory regulation on SAE reporting and submit drug safety reports to the Institutional Review Board (IRB) and/or Independent Ethics Committee (IEC) in accordance with the appropriate requirements.

7.6 Overdose

Overdose refers to that the subject has used (intentionally or accidentally) a drug in excess of the prescribed dose in the protocol. If an overdose occurs, the subject may be given appropriate symptomatic and supportive treatment. Any adverse reactions resulted from overdose should be reported to the CRA and included in the standard AE reporting.

Overdose with a SAE will be reported in accordance with SAE standard reporting methods and time frame.

7.7 Pregnancy

Female subjects or female partners of the male subjects who have child-bearing potential should take appropriate contraceptive measures from the signing of the ICF to at least 3 months after the last dose of the study drug and at least 6 months after the last dose of chemotherapeutics.

During the study, female subjects should stop the study treatment as soon as they become pregnant and inform the investigator. The investigator should report the pregnancy event to the sponsor (or authorized representative) within 24 hours after awareness of the pregnancy. Monitoring of the subjects will be continued until the end of pregnancy. Pregnancy within 6 months after the last dose of the study drug should be reported to the investigator.

If a female subject or the female partner of a male subject becomes pregnant during treatment and within 6 months after the last dose of the study drug, whichever occurs later, the investigator should complete the 'Pregnancy Report Form' within 24 hours after awareness of the pregnancy, report it to the sponsor (or authorized representative), and record it in the eCRF for follow-up of the outcome. Pregnancy events should be followed up till 30 days after the end of pregnancy.

Any AE/SAE occurring in the mother and newborn during pregnancy, such as spontaneous abortion or induced abortion, birth defects or congenital anomaly of the newborn, malformations and abnormalities of stillbirths, complications of the mother and newborn, should be documented and reported with reference to "7.4 Documentation of AEs" and "7.5.1 Reporting Requirements for SAEs".

8 STATISTICAL METHODS

8.1 Sample Size Estimation

This study uses parallel dual primary endpoints of PFS and OS, where one interim analysis is planned for OS when a target number of PFS events is observed and one final analysis is planned for OS when a target number of OS events is observed, while only one final analysis is planned for PFS. To control the overall type I errors, the allocation of α is as follows:

PFS: $\alpha = 0.005$ (one-sided)

OS: $\alpha = 0.02$ (one-sided)

In this study, subjects are randomized into the treatment group and the control group at a ratio of 2:1. The sample size is based on the assumption that the median PFS is 5 months in the of the control group, i.e., placebo + chemotherapy (cisplatin + 5-FU), and the median PFS is 7.35 months in the HLX10 + chemotherapy group, i.e., an HR of the HLX10 + chemotherapy group to the control group is approximately 0.68. When type I error $\alpha = 0.005$ (one-sided) and 24-month enrollment are assumed and the final analysis of PFS is planned to be performed at approximately month 4 after the last subject is enrolled, a minimum of 339 PFS events needs to be observed to achieve a power of 80%. Assuming a drop-out rate of 10%, a total of 495 subjects need to be enrolled in the 2 groups (330 in the HLX10 group and 165 in the control group).

Assuming that the median OS of the control group is 10 months and that the median OS of the HLX10 + chemotherapy group is 13.70 months, namely, the HR of the HLX10 + chemotherapy group to the control group is approximately 0.73. One interim efficacy will be analyzed by the Group Sequential Design when a target number of PFS events is observed, and the O'Brien-Fleming-like α-spending function of the Lan-Demets algorithm will be used to control the overall type I error rate $\alpha = 0.002$ (one-sided). Assuming that the duration of enrollment is 24 months and that the final analysis of OS is scheduled to be performed at approximately month 12 after the last subject is enrolled, a minimum of 388 OS events needs to be observed to achieve a power of 80% and a total of 540 patients needs to be enrolled in both groups (360 cases in the HLX10 group and 180 cases in the control group).

Considering the sample size required for PFS and OS evaluation, a total of 540 patients (360 in the HLX10 group and 180 in the control group) need to be enrolled in this study.

8.2 Statistical Analysis Sets

8.2.1 Intent-to-treat (ITT) set

ITT set is defined as all subjects randomized to the study. The ITT population will be the primary analysis population for the efficacy analysis of this study. The analysis of ITT population will be conducted based on randomized treatment groups.

8.2.2 Per protocol set (PPS)

PPS is a subset of ITT set. All randomized subjects without major protocol deviations significantly affecting the primary efficacy evaluation constitute the PPS. The definition of specific PPS will be confirmed before database lock. Analysis based on PPS will, as supportive analysis, complement the analysis based on ITT.

8.2.3 Safety set (SS)

SS is defined as all subjects who have received at least one dose of study drug. The safety population will be the primary analysis population for safety assessment, and will be analysed based on actual treatment groups.

8.2.4 Pharmacokinetics set (PKS)

All subjects who have received at least one dose of HLX10, and have at least one post-dose detectable concentration at a planned PK time point and have no major protocol deviations that may impact the PK evaluation significantly. The PK set will be used for PK analysis.

8.3 Interim Analysis and Final Analysis

In this study, an Independent Data Monitoring Committee (IDMC) will be established to conduct the interim analysis. The IDMC will be responsible for monitoring the study safety and efficacy data, assessing the study implementation quality, suggesting study design adjustments, and other emergency analyses and suggestions under blinded conditions and determined by the IDMC.

In this study, one efficacy interim analysis is planned for OS when a target number of PFS events is observed and one final analysis is planned for OS when a target number of OS events is observed, while only one final analysis is planned for PFS. An O'Brien-Fleming-like α-spending function (Lan-DeMets approximation) is used to control the overall type I error rate.

- The interim analysis of OS is planned to be performed during the final analysis of PFS, with the primary objective to perform safety assessments and superiority tests of PFS and OS endpoints. The significance level of Log-Rank test of PFS in this analysis is 0.01 (two-sided). According to O'Brien Fleming α-spending function, the significance level of Log-Rank test of OS in this analysis (about 75% OS event information) is 0.014 (two-sided);
- The final analysis of OS is planned to be performed when a target number of OS events (approximately 388) is observed, and the significance level is 0.036 (two-sided) for final OS endpoint analysis;
- Significance level for each analysis will be modified based on the actual number of PFS and OS events reached at the analytical time point. If an original hypothesis is rejected for an endpoint at the analytical time point, the recovery and redistribution of the α endpoint are performed and the significant level of the other endpoint at each analytical time point is updated.

8.4 Statistical Analysis Methods

SAS 9.2 (or above) statistical analysis software will be used for all statistical analyses. Standard descriptive statistics include median, mean, standard deviation, minimum, and maximum for continuous variables; and number and percentage for categorical variables.

The detailed statistical analysis plan and methods are presented in the statistical analysis plan.

8.4.1 Demographics, medical history, and baseline characteristics

Demographic information and baseline characteristic data, medical history, and concomitant medications of all randomized subjects will be summarized using descriptive statistical methods based on randomized grouping.

8.4.2 Medication compliance

Compliance of the study treatment (HLX10 or placebo in combination with cisplatin + 5-FU) will be summarized by study group using descriptive statistical methods.

8.4.3 Efficacy analysis

8.4.3.1 Analysis of primary efficacy endpoint

This study uses parallel dual primary endpoints of PFS and OS which are assessed by the IRRC per RECIST v1.1 criteria.

Progression-free survival (PFS) is assessed by IRRC as per RECIST v1.1. PFS is defined as a period starting from randomization and ending when death due to PD or any other reason (whichever occurs first) is noted for the first time.Data of subjects with neither PD nor death will be censored on the day of the final valid tumor assessment. Data of surviving subjects not undergoing any tumor assessment during the study will be censored on the day of randomization. Data of subjects who have no PD reported and initiate any anti-tumor therapy not specified in the protocol will be censored on the day of the last evaluable tumor assessment prior to the initiation of subsequent anti-tumor treatment. Censoring rules are detailed in the Statistical Analysis Plan (SAP).

Overall survival (OS) is defined as a period from randomisation through death regardless of causality. Data of patients without death record will be censored on the last known survival date. Data of patients not providing any follow-up information will be censored on the day of randomization. Censoring rules are detailed in the Statistical Analysis Plan (SAP).

Inter-group comparison of PFS and OS will be performed using stratified Log-Rank test with the following stratification factors: PD-L1 expression level (CPS < 10 versus CPS ≥ 10), age (≥ 65 years versus < 65 years), and tumor state (locally advanced versus distant metastasis). The significant level of PFS is 0.01 (two-sided) at the final analysis, and the significant levels of OS are 0.014 (two-sided) at the interim analysis and 0.036 (two-sided) at the final analysis; HR and its 95% CI will be estimated by stratified COX proportional risk model; the Kaplan Meier method will be used to estimate the median PFS/OS and 95% CI (Brookmeyer-Crowley method) with Kaplan-Meier curves plotted.

The final analysis of PFS and efficacy interim analysis of OS endpoints are performed when a target number of PFS events is observed, and at this time, when the P-Value (two-sided) is < 0.01 in the final analysis of PFS based on the Log-Rank test or the P-Value (two-sided) is < 0.014 in the interim analysis of OS, the difference in efficacy between HLX10 in combination with chemotherapy (cisplatin + 5-FU) and placebo in combination with chemotherapy (cisplatin + 5-FU) is statistically significant; or the final analysis of OS endpoints is performed when the target number of OS events (approximately 388 cases) is observed, and at this time, when the P-Value (two-sided) is < 0.036 in the final analysis of OS based on the Log-Rank test, the difference in efficacy between HLX10 in combination with chemotherapy (cisplatin + 5-FU) and

placebo in combination with chemotherapy (cisplatin + 5-FU) is statistically significant. When the actual number of observed events does not meet or exceeds the planned number of events at the interim/final analysis, the significant level can be adjusted to control the overall type I error rate using an O'Brien-Fleming-like α-spending function (Lan-DeMets approximation). The judgment boundaries and discontinuation criteria are detailed in the Statistical Analysis Plan (SAP). For patients who continue to use other anti-tumor treatment after the end of study treatment, the subgroup analysis and other methods will be used to assess the real efficacy of the study treatment on the OS.

With the α redistribution strategy of Group Sequential Holm Procedure, if the original hypothesis is rejected in the interim/final analysis for a primary efficacy endpoint (i.e., the actual P value at the analysis time point is less than the corresponding nominal significant level), all the initial α allocated to the primary efficacy endpoint will be recovered and allocated to another primary efficacy endpoint that does not reject the original hypothesis. And the O'Brien-Fleming-like α-spending function (Lan-DeMets approximation) will be used to recalculate and update significant levels at each analysis time point. For example, in the final analysis of PFS endpoints, if the P value of Log-Rank test is less than 0.01, while the P value of OS is greater than or equal to 0.014, all α (one-sided, 0.005) initially allocated to PFS will then be recovered and allocated to OS, i.e., in this case, the total α update of OS is 0.025 (one-sided). The O'Brien Fleming-like α-spending function (Lan-DeMets approximation) will be used to recalculate the significant level of each interim analysis/final analysis of OS, and the actually calculated P value of OS will be compared with the updated significant level.

8.4.3.2 Analysis of secondary efficacy endpoints

Progression-free survival (PFS) is assessed by IRRC as per iRECIST: PFS is defined as a period starting from the randomization and ending with PD determined by iRECIST criteria or death of any reason (whichever occurs first). The statistical method for PFS is the same as that for the primary efficacy endpoint.

PFS assessed by the investigator as per RECIST v1.1 and iRECIST respectively will be statistically analyzed using the same method as that for primary efficacy endpoints.

Objective response rate (ORR) is assessed by the IRRC as per RECIST v1.1 and iRECIST respectively and assessed by the investigator as per RECIST v1.1 and iRECIST respectively. ORR is defined as the percentage of subjects whose best overall responses are evaluated as complete response (CR/iCR) or partial response (PR/iPR). The stratified Miettinen-Nurminen method will be used to test the inter-group difference in the ORRs and their 95% CIs.

Stratification factors: PD-L1 expression level (CPS < 10 versus CPS ≥ 10), age (≥ 65 years versus < 65 years), and tumor state (locally advanced versus distant metastasis).

Duration of response (DOR) is assessed by the IRRC as per RECIST v1.1 and iRECIST respectively and evaluated by the investigator as per RECIST v1.1 and iRECIST respectively. DOR is defined as a period from the first documentation of response (CR/iCR or PR/iPR) to the PD or death (whichever occurs first) determined by RECIST v1.1/iRECIST criteria. The DOR will be analyzed only for subjects whose best overall responses are evaluated as CR/iCR or PR/iPR. Data of patients not experiencing PD or death after achieving response will be censored on the day of the final tumor assessment; if no tumor assessment is performed after response achievement, then data of such patients will be censored on the day of tumor assessment when response is achieved. Censoring rules are detailed in the Statistical Analysis Plan (SAP). The Kaplan-Meier method is used to estimate the median DOR, and the Kaplan-Meier curve will be plotted.

8.4.4 Safety analysis

AEs will be described per MedDRA (version 22.0), and graded per CTCAE v4.03. AEs during or after administration of study drug will be summarized per CTCAE grades. Adverse events and concomitant medications during study treatment will be separately summarized by treatment groups. Clinical laboratory parameters, ECOG, vital signs, physical examinations, and ECG will be summarized by treatment group and visit. Analysis will describe and present the observed values and changes from baseline by visit in the study.

8.4.5 Pharmacokinetics and immunogenicity analysis

Pharmacokinetic parameters will be calculated using WinNonlin 7.0 (or above).

The ADA (NAb) positive rate at each visit time point should be summarized.

The statistical methods will be described in detail in the statistical analysis plan.

Other PK relationships between HLX10 or 5-FU + cisplatin exposure and safety, immunogenicity, and/or efficacy data will be evaluated separately, as appropriate. If this analysis is performed, a separate analysis plan will be prepared and the results will be reported separately (not presented in the clinical study report).

8.4.6 Biomarker analysis

Immunohistochemistry may be used to analyse other markers for tissues obtained to determine the expression state of PD-L1 as part of the screening procedure. The primary objective is to assess the relationship between PD-L1 expression, MSI, TMB, and efficacy.

8.4.7 Analysis of patient-reported outcomes

The patient's functional status and self-perception will be recorded on the EQ-5D-5L Questionnaire, EORTC QLQ-C30 Questionnaire, and EORTC QLQ-OES18 Questionnaire.

The observed values of total score, sub-score, and individual score at each visit and the changes from the baseline will be described statistically based on the randomized group. The specific analysis method will be described in detail in the Statistical Analysis Plan. All PRO analyses will be performed based on the intention to treat (ITT) set unless otherwise indicated.

8.4.7.1 EQ-5D-5L questionnaire analysis

The EQ-5D-5L index comprises five health dimensions (mobility, self-care, usual activities, pain/discomfort, and anxiety/depression). The respondent is asked to tick the statement best describing his/her health on the day in each of the dimensions from five possible options with incrementally increased severity (no problem, slight problems, moderate problems, severe problems, and extreme problem). An EQ-5D health status is a five-digit number, so there are a total of 3125 non-repetitive health status. For example, 11,111 indicates that there is no problem in any of the five dimensions. EQ-5D value set derived from general population sample will be used for scoring (UK-based preference weights are the original one, and value sets derived from other countries will also be used in actual analysis), and these data will be converted to a weighted health state index. A summary index applicable to Chinese population (based on the MULT8r algorithm) is used for statistical analysis. In the absence of value set, EQ-5D-5L to EQ-5D-3L conversion will be used (Oemar and Janssen, 2013). In addition to the descriptive system, the respondents will be asked to answer a visual analogue scale ranging from 0 (the worst health status) to 100 (the best health status) to assess their health on the day of assessment. This score will be reported separately.

8.4.7.2 EORTC QLQ-C30 questionnaire analysis

EORTC QLQ-C30 consists of 30 questions, which can be incorporated to five functional scales (physical, role, cognitive, emotional, and social), three symptom scales (fatigue, pain, and nausea/vomiting), five single items (dyspnoea, insomnia, appetite lost, constipation, and diarrhoea), and an overall health assessment. EORTC QLQ-C30 will be evaluated according to the EORTC QLQ-C30 scoring manual (Fayers et al 2001). The outcome variables for each symptom scale/item, each functional scale/item, and overall health scale are scored from 0 to

100, calculated from scores in EORTC QLQ-C30 according to the EORTC QLQ-C30 scoring manual. Higher scores for overall health status and functional scale indicate better health status/functioning. Higher scores for symptom scale/item indicate higher severity levels of symptoms.

8.4.7.3 EORTC QLQ-OES18 questionnaire analysis

EORTC QLQ-OES18 is an oesophageal cancer disease specific and treatment-related supplementary scale to the core questionnaire EORTC QLQ-C30, and contains 18 items in 4 fields for determining relevant symptoms and side effects of treatment: dysphagia, eating difficulties, reflux, and pain. The total scores of each field are transformed, so that scores range from 0 to 100; a higher score represents a better level of QoL.

9 ETHICS

9.1 Ethic Requirements

This study will be performed in compliance with GCP, the Declaration of Helsinki, applicable laws and regulations and review comments from EC.

The investigator should ensure that the study is reviewed and approved by a qualified EC that follows GCP requirements. Prior to the initiation of the study, the investigator should submit the protocol, ICF, and other necessary materials to the EC for review and approval. The sponsor can provide the study drugs only after receiving approval from the EC. The EC must also be informed of any subsequent protocol supplements and SAEs occurring during the study that may affect the safety of subjects and continued participation in the study. The investigator is responsible for reporting to the EC about the progress of the study. In addition, the investigator must promptly submit copies of all communications with the EC to the sponsor. When reviewing and approving the protocol, the EC must confirm the protocol title, protocol number, and identify the reviewed protocol documents and the review date. Any additions or revisions to the protocol or ICF during the study require written approval from regulatory authorities according to applicable laws and regulations.

9.2 Informed Consent

The investigator must inform subjects of information about the study both in oral and written form. Subjects have the right to get details about the study.

ICF (along with the protocol) must be reviewed and approved by the EC. If necessary, the investigator has the responsibility to explain the contents of the ICF to subjects in a way and

wording that subject could understand. Subjects should have sufficient time to read the ICF before formally signing it.

The final ICF should contain the following: study objectives, procedures and duration of the study, examinations and operations, expected possible benefits and risks for subjects, and notification to subjects that they may be assigned to different groups of the study; treatment and corresponding compensation available to subjects in the event of study-related damages; confidentiality of subject's personal data, etc.

The ICF must be signed and dated by the subject or the subject's legally authorised representative, as well as the investigator who implements the informed consent. Both the investigator and the subject should keep one copy of the ICF. If important new information about the study drug becomes available, the ICF must be revised in writing and submitted to the EC for approval before obtaining the subject's consent again.

9.3 Confidentiality of Subjects

The investigator is responsible for maintaining the anonymity of the subjects. Only initials, numbers and/or codes, rather than subjects' names, can be used to identify subjects in CRF or other documents. The investigator must carefully keep the subject enrolment form that records the subject's code, name, and home address. The investigator must keep strictly confidential documents containing identity information of the subjects.

10 DATA MANAGEMENT

10.1 Completion of Raw Data and Electronic Case Report Form (eCRF)

Data generated in the clinical study will be processed according to the data management of the sponsor or authorized team and relevant SOPs of the biostatistics, in order to ensure the authenticity, integrity, safety, and traceability of clinical study data.

Medidata Rave system is used as the EDC system in this study. Data of the eCRF are all from original medical records and promptly filled in by the investigator or personnel authorized by the investigator. The investigator and all authorized personnel will receive training on system and data entry prior to the initiation of the study and the entry of any data into the system.

10.2 Design and Establishment of the Database

The database should be designed to meet FDA 21 CFR Part 11 requirements and comply with the CDISC criteria. The database should be managed in data traces such as system login, data entry, modification, or deletion.

Access and permissions to the EDC system will be set according to the functions of each relevant person in the study.

10.3 Data Coding

Data department is responsible for the medical coding of this study. Coding includes past medical history, prior medication, concomitant medication, and AEs.

Past medical history and AEs will be coded according to the MedDRA Dictionary, and prior medication and concomitant medication will be coded according to the World Health Organization Drug Dictionary (WHODrug), which is classified using the Anatomical Therapeutic and Chemical Classification (ATC) system. All dictionaries used should be in the version confirmed by the sponsor.

In the process of coding, for any data that cannot be coded due to improper, inaccurate or ambiguous medical terminology, data manager (DM) will ask the investigator to verify and confirm them in the form of data query.

Before the database is locked, DM will send a medical code report to the sponsor, which must be approved by the sponsor.

10.4 Data Verification

Data verification includes computerized program verification (Edit Check), manual verification and verification of the consistency of all external data. A query should be given promptly in the EDC system for inconsistent data or doubtful data found in the verification, and the investigator or personnel authorized by the investigator should address the data query by modifying data or giving explanation in a timely manner. Difficult data situations will be discussed and resolved at the data verification meeting prior to database lock.

10.5 Database Lock

Before the database is locked, all eCRFs need to be electronically signed by the principal investigator to confirm the authenticity and accuracy of the data.

At the end of the study, after the appropriateness of the database established is confirmed in the data verification meeting, the database will be locked jointly by the principal investigator, sponsor, and statisticians. The locked database is not allowed to be changed in principle. However, if any important data problem is found, the database can be unlocked with the consent of the relevant parties and a written document signed, and re-locked after the update of the problematic data.

After that, the database will be statistically analysed by the statisticians according to the requirements of the statistical analysis plan, and the statistical analysis report is provided by the statistical institution.

10.6 Blind Verification and Unblinding

ICH Biostatistics Guidelines E9 recommends blind verification prior to the unblinding process for two purposes:

- Discussing each issue in a blind state to determine the number of subjects in data sets.
- Reviewing the study data in a blind state.

After the data is locked, the grouping of drugs in the study will be unblinded. Prior to unblinding, the data management leader can provide the 'Database Lock/Relock Confirmation Form' signed by the parties to ensure that the database is locked.

11 STUDY MANAGEMENT

11.1 Quality Control and Quality Assurance

In order to ensure the quality of the study, the sponsor and investigator should jointly discuss and establish a clinical study program prior to the formal initiation of the study. GCP training should be provided for clinical study personnel who are participating in the study. All study sites must manage the study drug, including its receiving, storage, dispensing, and return, in accordance with SOP. According to GCP guidelines, necessary measures should be taken during the design and implementation of the study to ensure that data collected are accurate, consistent, complete, and reliable. All observed results and abnormal findings during the clinical study should be carefully verified and documented in a timely manner to ensure the reliability of the data. Strict specifications should be established for various instruments, equipment, reagents, reference standards etc., used for various examinations in clinical study to ensure their normal work. Information required by the protocol will be entered into the CRF by the investigator and verified for completeness and accuracy by the monitor, and be amended or supplemented by staff of the study site. The drug regulatory authorities and the sponsor may entrust the auditor to perform systematic audit for study-related activities and documents to evaluate whether the study is performed in accordance with the protocol, SOPs, and applicable laws and regulations, as well as whether the study data is recorded timely, truthfully, accurately, and completely. The audits should be performed by persons who are not directly involved in the clinical study.

11.1.1 Training

According to GCP guidelines, the CRA should have qualifications recognized by the sponsor. Prior to initiation of the clinical study, the investigator should be trained by the study site director to familiarize and understand the protocol, comprehend GCP guidelines, use consistent recording methods and interpretation criteria, and to strictly carry out the study as specified in the protocol.

11.1.2 Clinical monitoring

CRA is the point of contact between sponsor and investigator. The sponsor's CRA will conduct monitoring in accordance with GCP and the company's SOPs. CRAs will establish and maintain regular contact between the investigator and the sponsor.

CRAs will, in accordance with all relevant regulatory requirements and standards, visit the study site at regular intervals or when necessary, to perform clinical monitoring, supervise the operation and progress of the clinical study, check and confirm the accuracy, integrity and

consistency with original data of all data records, reports, and eCRF entry, and ensure clinical study's conformity with the protocol. The investigator should actively assist CRA.

11.1.3 Audit

During the study period, the sponsor may conduct quality assurance audits to the study sites, databases, and documents. Audits include: drug supply, required study files, records of informed consent process, consistency of CRFs with the source documents, etc. Content and scope of audit may be expanded as appropriate. Upon reasonable notice, the investigator shall allow study-related audits by the auditor commissioned by the sponsor and inspections from the regulatory authorities.

In addition, NMPA may also inspect the study.

11.1.4 Data management/coding

An EDC system will be used for this study, meaning that all eCRF data will be entered in electronic forms at the study site.

The data management and biostatistics department will process the data generated in this clinical study in accordance with the relevant SOPs.

Data collection will be completed by authorized study site personnel designated by the investigator. Appropriate training and security measures should be completed with the investigator and all authorized study site personnel before the study is initiated and before any data is entered into the system for any subjects.

CRAs will compare the eCRF with the source documents to ensure there are no deviations between key data. All entries, corrections, and alterations must be made by the investigator or his/her designee. A query will be given and sent to the investigator by the relevant study staff. For this purpose, the EDC application will be tracked by audits, which means that the name of the study staff, and time and date of the audit will be recorded.

The investigator is responsible for maintaining the source documents. These documents are subject to inspection by CRAs at each monitoring visit. The investigator must submit a complete eCRF for each subject who have received the study drug, regardless of duration of treatment. All supporting documentation, such as laboratory or hospital records, submitted concurrently with eCRF should be clearly identified using the study and subject number. Any personal information, including the subject's name, must be deleted or unidentifiable to maintain the confidentiality of the subject information.

11.1.5 Missing and useless data

The process flow for missing and useless data is detailed in SAP.

11.2 Documentation and Preservation of Study Data

The investigator is responsible for maintaining the documents (protocol and protocol amendments, completed eCRFs, signed ICFs, important communications, and all other supporting documentation) necessary to the study. The study site shall develop a plan for the preservation of these documents for 5 years after the completion of the study. The study site shall maintain these documents until at least 2 years after the final approval for the marketing of the study drug, and at least 5 years after there is no pending or marketing application for the study drug, or after the clinical development of the study drug has been formally discontinued. These documents should be kept for an extended period of time if so requested by the appropriate regulatory authority or by the hospital, institution or private clinic conducting the study. The subject code (subject name and corresponding study number) should also be kept for the same period. Subject to the consent of the sponsor, these documents may be transferred to another responsible party, who shall follow the document preservation policy. The sponsor must be notified in writing when the documents are transferred. The investigator should contact the sponsor before disposing any records of the study.

11.3 Follow-up and Medical Measures after the End of the Study

AEs/SAEs (including laboratory test abnormalities) that are not resolved at the end of the study or at the time of early withdrawal of the subject must be followed up.

After the end of study treatment, the investigator should offer necessary and reasonable medical interventions to the subjects to protect their safety, rights, and interests.

12 ALL PARTIES' RESPONSIBILITIES

12.1 Investigator's Responsibilities

Investigator's responsibilities mainly include but are not limited to:

1) The investigator shall hold a joint discussion with the sponsor to finalize and sign the protocol, which will be implemented after approval by the EC.
2) The investigator must read and understand the contents of the protocol in detail and perform the study strictly in accordance with the protocol.

3) The investigator should understand and be familiar with the property, effect, efficacy, and safety of the study drug (including relevant information in preclinical study of the drug), and at the same time master all new information related to the drug found during the clinical study.

4) The investigator must perform the clinical study in a medical institution equipped with good medical facilities, experimental equipment, and medical staff, which should have all the facilities to deal with emergencies to ensure the safety of subjects. Laboratory results should be accurate and reliable.

5) The investigator should obtain the approval from the medical institution or the regulatory authority, and ensure that there is sufficient time to take charge and complete the clinical study within the period required by the protocol. The investigator should explain study-related information, requirements, and responsibilities to all the staff involved in the clinical study, and ensure that a sufficient number of subjects who meet the protocol enter the clinical study.

6) The investigator should explain to subjects the details related to the study approved by the EC, and obtain the ICF.

7) The investigator will be responsible for making clinical study-related medical decisions to ensure that subjects will receive proper treatment if AEs occur during the study.

8) Take necessary measures to ensure the safety of subjects and record such measures. In the event of SAEs during the clinical trial, the investigator should give immediate and appropriate treatments for the subject, abide by requirements of each regulatory regulation regarding SAE reporting, and submit drug safety reports to the sponsor, the Institutional Review Board (IRB), and/or the Independent Ethics Committee (IEC).

9) The investigator should guarantee that the data on the medical record and CRF is documented truly, accurately, completely, promptly, and legally.

10) The investigator should accept the monitoring and auditing by the CRA or auditor designated by the sponsor, as well as the auditing and inspection by drug regulatory authority to ensure the quality of the clinical study.

11) Agreements will be made between the investigator and sponsor for the cost of the clinical study, and which should be written in the contract. The investigator should not charge subjects for the study drug during the clinical study.

12) After completion of the clinical study, the investigator must write a summary report with signature and date, and send it to the sponsor.

12.2 Sponsor's Responsibilities

Sponsor's responsibilities mainly include but are not limited to:

1) The sponsor should obtain approval from NMPA.
2) The sponsor is responsible for initiating and applying for a clinical study, and providing study funds.
3) The sponsor should provide investigator's brochure, which shall include the chemical, pharmaceutical, toxicological, pharmacological, and clinical (including previous and ongoing studies) information and data of the study drug.
4) The clinical protocol should be designed jointly by the sponsor and investigator. The protocol and contract should be signed on the agreement of both parties.
5) The sponsor should provide study drug and control drug that are identified easily, encoded correctly and labelled with special label for the investigator, and ensure that the quality is qualified. The study drug should be packaged and stored properly as required in the protocol. The sponsor should establish management and documentation systems for the study drug.
6) The sponsor should appoint qualified CRAs who are accepted by the investigator.
7) The sponsor should establish quality control and quality assurance systems for the clinical study, and organize audit of the clinical study to ensure quality.
8) The sponsor should promptly scrutinize the SAEs with the investigator, take necessary actions to ensure the safety, rights, and interests of the subjects, and report in a timely manner to the drug regulatory authority and health administrative department.
9) The sponsor is responsible for submitting the study summary report to the NMPA.
10) The sponsor should purchase the drug clinical study liability insurance for this study. The sponsor should provide comprehensive medical coverage for the subjects participating in the clinical study. For subjects who suffer study-related damage or death, the sponsor is responsible for affording the cost of treatment and providing appropriate financial compensation. The sponsor should provide the investigator with legal and economic guarantees, except for those caused by medical malpractice.

13 CONFIDENTIALITY AND PUBLICATION OF STUDY RESULTS

All information about this study (not limited to the following documents: protocol, Investigator's Brochure) must be kept strictly confidential. The investigator must recognize that the scientific or medical information derived from this study may be of commercial value to the sponsor. The investigator should keep the information and data related to this study confidential. If the investigator intends to publicly publish information related to this study or the conclusions drawn from this study, he/she shall consult with the sponsor in advance and obtain the written consent of the sponsor. The sponsor may require the investigator not to publish information about the study before the investigational product is approved for marketing, in order to protect the sponsor's rights and interests.

The sponsor has the right to issue or publish information or data related to this study, or submit them to the drug regulatory authority. The sponsor should obtain consent from the investigator if investigator's name is to appear in issuance, publication, or advertisement.

14 REFERENCES

[1] China National Health Commission of the People's Republic of China. Guidelines for Diagnosis and Treatment of Oesophagus Cancer, 2018.

[2] Jemal A,F. Bray,M.M.Center,et al.Global cancer statistics.CA Cancer J Clin,2011.61(2):p.69-90.

[3] Pickens A and M.B.Orringer,Geographical distribution and racial disparity in esophageal cancer.Ann Thorac Surg,2003.76(4):p.S13679.

[4] Chen W, Sun K, Zheng R, et al.Cancer incidence and mortality in China, 2014[J]. Chin J Cancer Res, 2018, 30(1):1-12.

[5] Chang S, Kohrt H, Maecker HT. Monitoring the immune competence of cancer patients to predict outcome[J]. Cancer Immunol Immunother, 2014;63:713-719.

[6] Zeng H, Chen W, Zheng R, et al. Changing cancer survival in China during 2003-15: a pooled analysis of 17 population-based cancer registries[J]. The Lancet Global health, 2018, 6(5):e555-e567.

[7] Stadler S, Weina K, Gebhardt C, et al. New therapeutic options for advanced non-resectable malignant melanoma.[J]. Adv Med Sci, 2015, 60(1):83-88.

[8] Basso D,Fogar P,Falconi M,et al.Pancreatic tumors and immature immunosuppressive myeloid cells in blood and spleen：role of inhibitorv co-stimulatorv molecules PDLl and CTLA4.An in vivo and in vitro study[J] .PLOSONE，2013，8(1) :e54824.

[9] Chen S, Liu H, Su N, et al. Myeloid-derived suppressor cells promote age-related increase of lung cancer growth via B7-H1.[J]. Experimental Gerontology, 2015, 61:84-91.

[10] Ohigashi Y, Sho M, Yamada Y, Tsurui Y, Hamada K, Ikeda N, Mizuno T, Yoriki R, Kashizuka H, Yane K. Clinical significance of programmed death-1 ligand-1 and programmed death-1 ligand-2 expression in human esophageal cancer. Clin Cancer Res. 2005;11:2947-2953. 50.

[11] Chen L, Deng H, Lu M, Xu B, Wang Q, Jiang J, Wu C. B7-H1 expression associates with tumor invasion and predicts patient's survival in human esophageal cancer. Int J Clin Exp Pathol. 2014;7:6015-6023.

[12] Nishizumi H . Development of lupus-like autoimmune diseases by disruption of the PD-1 gene encoding an ITIM motif-carrying immunoreceptor[J]. Immunity, 1999, 11(2):141-151.

[13] Okazaki T, Honjo T. PD-1 and PD-1 ligands: from discovery to clinical application[J]. International Immunology, 2007, 19(7):813-824.

[14] Nishizumi H . Development of lupus-like autoimmune diseases by disruption of the PD-1 gene encoding an ITIM motif-carrying immunoreceptor[J]. Immunity, 1999, 11(2):141-151.

[15] Okazaki T, Honjo T. PD-1 and PD-1 ligands: from discovery to clinical application[J]. International Immunology, 2007, 19(7):813-824.

[16] [Doi T, Piha-Paul SA, Jalal Sl,Safety and Antitumor Activity of the Anti–Programmed Death-1 Antibody Pembrolizumab in Patients With Advanced Esophageal Carcinoma.J Clin Oncol. 2018 Jan 1;36(1):61-67].

[17] Shah MA, Kojima T, Hochhauser D, et al. Efficacy and Safety of Pembrolizumab for Heavily Pretreated Patients With Advanced, Metastatic Adenocarcinoma or Squamous Cell Carcinoma of the Esophagus: The Phase 2 KEYNOTE-180 Study. JAMA Oncol. 2018 Dec 20.

[18] Kudo T, Hamamoto Y, Kato K,et al.Nivolumab treatment for oesophageal squamous-cell carcinoma: an open-label, multicentre, phase 2 trial.Lancet Oncol. 2017 May;18(5):631-639.

15 APPENDICES

Appendix 1: Common Terminology Criteria for Adverse Events

This study will report AE using CTCAE v4.03. CTCAE v4.03 can be downloaded from the home page of the Cancer Therapy Evaluation Program (CTEP). CTCAE v4.03 shall be used in all relevant study sites. The URL is as follows:

https://evs.nci.nih.gov/ftp1/CTCAE/CTCAE_4.03/CTCAE_4.03_2010-06-14_QuickReference_5x7.pdfpdfpdf

Appendix 2: Response Evaluation Criteria in Solid Tumors (RECIST 1.1)

The following is the Response Evaluation Criteria in Solid Tumors (RECIST) Version 1.1. For more details, please refer to the English version at https://ctep.cancer.gov/protocolDevelopment/docs/recist_guideline.pdf.

Methodology

- CT and MRI are currently the best reproducible methods to measure target lesions selected for response assessment. Lesions on CT scan are measured based on the assumption that: CT slice thickness is 5 mm or less. When CT scans have slice thickness greater than 5 mm, the minimum size for a measurable lesion should be greater than or equal to 10 mm or 2 times the slice thickness. MRI is acceptable to assess disease throughout the study.
- The same method of assessment and the same technique should be used to characterize each identified and reported lesion throughout the study.
- Ultrasound (US) should not be used to measure objective tumor response or PD. For this study, cross-sectional imaging techniques (CT or MRI) will be used to assess responses such as CR, PR, or SD. Fluorodeoxyglucose positron emission tomography (FDG-PET) is not suitable for evaluation of tumor response. It is sometimes reasonable to incorporate FDG-PET scanning to complement CT scanning in assessment of PD. To determine PD, new lesions are identified using FDG-PET as follows:
    1) PET negative at baseline and positive at follow-up is a sign of PD based on a new lesion.
    2) PET not available at baseline and positive at follow-up: If the positive PET at follow-up corresponds to a new lesion confirmed by CT, this is PD. If the positive PET at follow-up is not confirmed as a new lesion on CT, an additional follow-up CT scan is needed to determine if there is truly progression at this lesion (if so, the day of PD is the date of the initial abnormal PET scan). If the positive PET at follow-up corresponds to a pre-existing lesion on CT that is not progressing based on anatomic images, this is not PD.

As determined by the investigator, in case of incorporation of PET-CT, the CT examination should not be replaced with the specialized CT examination required in this protocol to complete the RECIST measurement, unless the site may confirm that the CT scan performed as part of the PET-CT is of the same diagnostic quality as the diagnostic CT (with intravenous and oral contrast agent).

In rare cases (e.g., after treatment to differentiate between residual benign lesions and residual malignant lesions in tumor types), cytology and histology can be used to differentiate between PR and CR. When effusions are known to be a potential adverse effect of treatment (e.g. with certain taxanes or angiogenesis inhibitors), the cytological confirmation of the neoplastic origin of any effusion that appears or worsens during treatment can be considered if the measurable tumor has met criteria for responses or SD in order to differentiate between responses (or SD) and PD.

Definition of measurable and non-measurable tumors:

All measurements should be taken and recorded in metric notation, using a ruler or callipers. Measurement results should be recorded in a unidimensional method. At baseline, tumor lesions/pathological lymph nodes will be categorized measurable or non-measurable according to the following definitions:

- **Measurable**: At baseline, measurable lesions are defined as: lesions that can be accurately measured in at least one dimension with a minimum size of 10 mm (longest diameter is recorded) by CT scan (CT scan slice thickness no greater than 5 mm). When the CT scan slice thickness is greater than 5 mm, the long diameter of the measurable lesion should be greater than 10 mm or 2 times the slice thickness. Malignant lymph nodes: To be considered pathologically enlarged and measurable, a lymph node must be ≥ 15 mm in short axis when assessed by CT scan.
- **Non-measurable:** All other lesions, including small lesions (longest diameter < 10 mm) or pathological lymph nodes with > 10 mm to < 15 mm short axis) as well as truly non-measurable lesions are considered non-measurable and characterized as non-target lesions. Lesions considered non-measurable include: bone lesions, leptomeningeal disease, ascites, pleural/pericardial effusion, lymphangitic involvement of skin or lung, and abdominal masses identified by physical exam that are not measurable by reproducible imaging techniques. Lymph nodes that have a short axis of < 10 mm are considered non-pathological and will not be recorded or followed up.

Clinical lesions will be considered measurable when they are superficial (e.g., skin nodules and palpable lymph nodes) and the diameter is greater than or equal to 10 mm measured with a calliper. For the skin lesions, documentation by colour photography, including a ruler to estimate the size of the lesion, is recommended. Lesions which cannot be accurately measured with callipers should be recorded as non-measurable.

Special considerations:

Lytic bone lesions or mixed lytic-blastic lesions, with identifiable soft tissue components, that can be evaluated by cross sectional imaging techniques such as CT or MRI, can be considered as measurable lesions if the soft tissue component meets the definition of "measurable" described above. Hyperplastic bone lesions are non-measurable.

Cystic lesions: Lesions that meet the criteria for radiographically defined simple cysts should not be considered as malignant lesions. Cystic lesions thought to represent cystic metastases can be considered as measurable lesions, if they meet the definition of "measurable" described above. However, if noncystic lesions are present in the same patient, they are preferred as target lesions.

Tumor lesions situated in a previously irradiated area, or in an area subjected to other local-regional therapy, are usually not considered measurable unless PD has been demonstrated in the lesion.

Baseline (i.e. pretreatment) confirmation of ‘target' and ‘non-target' lesions

During the treatment, a maximum of 5 target lesions will be selected for measurement (up to 2 lesions per organ). Target lesions should be selected based on their size and their suitability for accurate repeated measurements (either by imaging techniques or clinically).

A sum of the diameters (longest for non-nodal lesions, short axis for nodal lesions) for all target lesions will be calculated and reported as the baseline sum diameters. The baseline sum diameters will be used as reference to further characterize any objective tumor response.

- All other lesions (or sites of disease, including any measurable lesions or pathological lymph nodes that are not selected as target lesions) should be identified as non-target lesions. Non-target lesions should be documented and qualitatively evaluated during treatment. These non-measurable non-target lesions should be followed up as "present", "absent", or in rare cases "confirmed progression".
- Bone lesions: Bone scan, PET scan, or plain films are not adequate to measure bone lesions. Bone scans, MRI, CT, PET, PET/CT, or X-ray should be completed if signs or symptoms indicating bone metastasis are present. Another imaging technique (e.g., X-ray, CT, or MRI) must be used to confirm bone
- metastases in subjects with positive bone scans or PET scan.

Response criteria

The subject's tumor response will be assessed based on the target lesion response, non-target lesion response, and the appearance of new lesions or disappearance of old lesions.

Evaluation of target lesions

*CR:	Disappearance of all target lesions. Any pathological lymph nodes (whether target or non-target) must have reduction in short axis to < 10 mm.
*PR:	At least a 30% decrease in the sum of diameters* of target lesions, taking the baseline sum diameters as reference.
*PD:	At least a 20% increase in the sum of diameters of target lesions, taking the smallest sum of all target lesions recorded since the start of treatment as reference. In addition to the relative increase of 20%, the sum must also demonstrate an absolute increase of at least 5 mm. (Note: The appearance of one or more new lesions is also considered as PD).
*SD:	Neither sufficient shrinkage to qualify for PR nor sufficient increase to qualify for PD, taking the smallest sum diameters on study as reference.
N/A:	No target lesion is identified at baseline.
N/E:	The evaluation is not possible due to incomplete scan, incomplete scan results, or poor scan quality at the time of evaluation of the target lesion.

Diameters used:

For lymph node lesions: Shortest axis

For non-lymph node lesions: Longest sum diameter

The convention below will be applied in this study: If a lesion becomes small and is faintly seen or is too small to measure, a default diameter of 5 mm should be applied. If the size of the lesion subsequently increases in one direction to 5 mm or more, the true diameter of the lesion should be recorded. Lymphadenopathy should always have the actual short axis measurement recorded, even if the nodes regress to below 10 mm on study. When lymph nodes are included as target lesions, the ‘sum' of target lesions may not be zero even if CR criteria are met, since a normal lymph node is defined as having a short axis of < 10 mm. For CR, each lymph node must have a short axis diameter < 10 mm. For PR, SD, and PD, the actual short axis measurement of the lymph nodes is included in the sum of target lesions.

Evaluation of non-target lesions

CR:	Disappearance of all non-target lesions. All lymph nodes must be non-pathological in size (< 10 mm short axis)
Non-CR/Non-PD:	Persistent presence of one or more non-target lesions.
PD:	Appearance of one or more new lesions, or equivocal progression of existing non-target lesions.
N/A:	No non-target lesion is identified at baseline.
N/E:	The evaluation is not possible due to incomplete scan, incomplete scan results, or poor scan quality at the time of evaluation of the non-target lesion.

When the patient also has measurable lesion, to achieve ‘confirmed progression' based on the non-target lesion, there must be an overall level of substantial worsening in the non-target lesion. If the target lesion is evaluated as SD or PR, a significant increase in the overall tumor burden can still lead to discontinuation of therapy. When the patient has only non-measurable lesion, the increase in the non-measurable lesion should be comparable to the increase required for measurable lesion PD (e.g. equivalent to a 20% increase in the sum diameters of all measurable lesions).

Evaluation of best overall response

The overall response of subjects at each time point can be calculated as below:

Target lesions	Non-target lesions	New lesions	Overall response
CR	CR	No	CR
CR	Non-CR/non-PD	No	PR
CR	NE	No	PR
PR	Non-PD or N/E	No	PR
SD	Non-PD or N/E	No	SD
NE	Non-PD	No	NE
PD	Any	Yes or No	PD
Any	PD	Yes or No	PD
Any	Any	Yes	PD

CR = complete response; PR = partial response; SD = stable disease; PD = progressive disease; NE = not evaluable

Time point response of subjects with non-target lesions only

Non-target lesions	New lesions	Overall response
CR	No	CR
Non-CR/non-PD	No	Non-CR/non-PD
NE	No	NE
Confirmed PD	Yes or No	PD
Any	Yes	PD
Non-CR/non-PD is preferred over 'SD' for non-target lesions since SD is increasingly used as endpoint of efficacy in some trials, and this classification is not recommenced when no lesions can be measured.		

Special notes on response assessment

Subjects with a global deterioration of health status requiring discontinuation of treatment without objective evidence of PD should be classified as 'symptomatic progression'. In this case, it is not possible to classify 'PD' as the overall objective response of the tumor. Every effort should be made to document objective evidence of progression even after discontinuation of treatment.

In some circumstances it may be difficult to distinguish residual lesion from normal tissue. When the evaluation of CR depends upon this determination, the investigator may decide to further examine the residual lesion (fine needle aspiration/biopsy) to confirm the CR.

For equivocal findings of progression (e.g. very small and uncertain new lesions; hyperplastic changes, or necrosis in existing lesions), treatment may continue until the next scheduled assessment. If at the next scheduled assessment, progression is confirmed, the date of progression should be the earlier date when progression was suspected.

Duration of overall response

The DOR is measured from the time measurement criteria are first met for CR/PR (whichever is first recorded) until the first date that recurrent or progressive disease is objectively documented, taking the minimum measurement since start of treatment as the reference value to PD.

Appendix 3: Quality of Life Questionnaires EORTC QLQ-C30, EQ-5D-5L and EORTC QLQ-OES18

For information only. Use questionnaires based on the actual situation.

EORTC QLQ-C30 (version 3)

We are interested in some things about you and your health. Please answer all of the questions yourself by circling the number that best applies to you. There are no "right" or "wrong" answers. The information that you provide will remain strictly confidential.

	Not at all	A little	Quite a bit	Very much
1. Do you have any trouble doing strenuous activities, like carrying a heavy shopping bag or a suitcase?	1	2	3	4
2. Do you have any trouble taking a long walk?	1	2	3	4
3. Do you have any trouble taking a short walk outside of the house?	1	2	3	4
4. Do you need to stay in bed or a chair during the day?	1	2	3	4
5. Do you need help with eating, dressing, washing yourself, or using the toilet?	1	2	3	4
**During the past week:**				
6. Were you limited in doing either your work or other daily activities?	1	2	3	4
7. Were you limited in pursuing your hobbies or other leisure time activities?	1	2	3	4
8. Were you short of breath?	1	2	3	4
9. Have you had pain?	1	2	3	4
10. Did you need to rest?	1	2	3	4
11. Have you had trouble sleeping?	1	2	3	4
12. Have you felt weak?	1	2	3	4
13. Have you lacked appetite?	1	2	3	4
14. Have you felt nauseated?	1	2	3	4
15. Have you vomited?	1	2	3	4
16. Have you been constipated?	1	2	3	4

**During the past week:**	Not at all	A little	Quite a bit	Very much
17. Have you had diarrhoea?	1	2	3	4
18. Did you feel tired?				
19. Did the pain interfere with your daily activities?	1	2	3	4
20. Have you had difficulty in concentrating on things, like reading a newspaper or watching television?	1	2	3	4
21. Did you feel tense?	1	2	3	4
22. Did you feel worried?	1	2	3	4
23. Did you feel irritable?	1	2	3	4
24. Did you feel depressed (low mood)?	1	2	3	4
25. Did you have difficulty in remembering things?	1	2	3	4
26. Did your physical condition or medical treatment affect your family life?	1	2	3	4
27. Did your physical condition or medical treatment affect your social activities?	1	2	3	4
28. Did you suffer financial difficulties due to your physical condition or medical treatment?	1	2	3	4

For the following questions please circle the number between 1 and 7 that best applies to you

29. How would you rate your overall health during the past week?

1 2 3 4 5 6 7

Very poor Very good

30. How would you rate your overall quality of life during the past week?

1 2 3 4 5 6 7

Very poor Very good

Health Questionnaire (EQ-5D-5L)

Under each heading, please tick the one box that best describes your health today.

Mobility

I have no problems in walking about ☐

I have slight problems in walking about ☐

I have moderate problems in walking about ☐

I have severe problems in walking about ☐

I am unable to walk about ☐

Self-care

I have no problems washing or dressing myself ☐

I have slight problems washing or dressing myself ☐

I have moderate problems washing or dressing myself ☐

I have severe problems washing or dressing myself ☐

I am unable to wash or dress myself ☐

Usual activities (e.g., work, study, housework, family, or leisure activities)

I have no problems doing my usual activities ☐

I have slight problems doing my usual activities ☐

I have moderate problems doing my usual activities ☐

I have severe problems doing my usual activities ☐

I am unable to do my usual activities ☐

Pain/discomfort

I have no pain or discomfort ☐

I have slight pain or discomfort ☐

I have moderate pain or discomfort ☐

I have severe pain or discomfort ☐

I have extreme pain or discomfort ☐

Anxious/depressed

I am not anxious or depressed ☐

I am slightly anxious or depressed ☐

I am moderately anxious or depressed ☐

I am severely anxious or depressed ☐

I am extremely anxious or depressed ☐

The Best Health Status

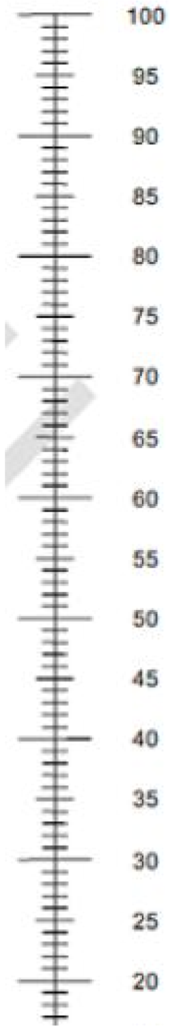

- We would like to know how good or bad your health is today.
- This scale is numbered from 0 to 100.
- 100 means the best health you can imagine
- 0 means the worst health you can imagine.
- Tick an "X" on the scale to indicate how your health is today. Now, please write down the number you marked on the scale in the space below.

Your health today = ☐

The Worst Health Status

EORTC QLQ-OES18

Patients sometimes report the following clinical symptoms. Please indicate the extent of these symptoms or problems you have experienced during the past week and circle the answer that best suits you.

During the past week:	Not at all	A little	Quite a bit	Very much
31. Could you eat solid food?	1	2	3	4
32. Could you eat liquidised or soft food?	1	2	3	4
33. Could you drink liquids (such as water, drinks)?	1	2	3	4
34. Have you had trouble swallowing your saliva?	1	2	3	4
35. Have you choked when swallowing?	1	2	3	4
36. Have you had trouble enjoying your meals?	1	2	3	4
37. Have you felt full up too quickly?	1	2	3	4
38. Have you had trouble with eating?	1	2	3	4
39. Have you had trouble with eating in front of other people?	1	2	3	4
40. Have you had a dry mouth?	1	2	3	4
41. Have you felt the taste of food and drink is different from usual?	1	2	3	4
42. Have you had trouble with coughing?	1	2	3	4
43. Have you had trouble with talking?	1	2	3	4
44. Have you had acid indigestion or heartburn?	1	2	3	4
45. Have you had trouble with acid or bile (taste bitter) coming into your mouth?	1	2	3	4
46. Have you had pain when you eat?	1	2	3	4
47. Have you had pain in your chest?	1	2	3	4
48. Have you had pain in your stomach?	1	2	3	4

Appendix 4: Eastern Cooperative Oncology Group (ECOG) - Performance Status

Score	ECOG status
0	Fully active, able to carry on all pre-disease performance without restriction.
1	Restricted in physically strenuous activity but ambulatory and able to carry out work of a light or sedentary nature, e.g., light housework, office work.
2	Ambulatory and capable of all self-care but unable to carry out any work activities. Up and about more than 50% of waking hours.
3	Capable of only limited self-care, confined to bed or chair more than 50% of waking hours.
4	Completely disabled. Cannot carry on any self-care. Totally confined to bed or chair.
5	Death

Appendix 5: Fridericia's Correction Formula

Fridericia's formula: $QTc = QT/RR^{0.33}$

Appendix 6: Cockcroft and Gault Formula

Creatinine clearance is calculated using serum creatinine measurements (mL/dL):

M: $\frac{(140 - \text{Age}) \times \text{Weight (kg)}}{\text{Serum creatinine (mg/dL)} \times 72} = \text{XX mL/min}$

F: $\frac{(140 - \text{Age}) \times \text{Weight (kg)}}{\text{Serum creatinine (mg/dL)} \times 72} = \text{XX mL/min} \times 0.85$

Adapted from Cockcroft DW et. al. Nephron. 1976;16(1):31-41.

Creatinine clearance is calculated using serum creatinine measurements (µmol/ L):

M: $\frac{(140 - \text{Age}) \times \text{Weight (kg)} \times 1.23}{\text{Creatinine (µmol/L)}}$

F: $\frac{(140 - \text{Age}) \times \text{Weight (kg)} \times 1.23 \times 0.85}{\text{Creatinine (µmol/L)}}$

Adapted from Cockcroft DW et. al. Nephron. 1976;16(1):31-41.

Appendix 7: New York Heart Association Classification

Grade	Behaviour Status
**I**	No limitation of physical activity. Ordinary physical activity does not cause undue fatigue, palpitation, or dyspnoea.
**II**	Slight limitation of physical activity. Comfortable at rest, but ordinary physical activity results in fatigue, palpitation, or dyspnoea.
**III**	Marked limitation of physical activity. Comfortable at rest, but less than ordinary physical activity causes fatigue, palpitation, or dyspnoea.
**IV**	Unable to comfortably engage in any physical activity. Symptoms of cardiac insufficiency at rest. Any physical activity may aggravate the discomfort.

Protocol Amendment Summary of Changes Table

**DOCUMENT HISTORY**		
**Version**	**Date**	**Notes**
Version 4.0	26 Jun 2021	Applicable for China only
Version 3.0	28 Feb 2021	Applicable for China only
Version 2.0	29 Oct 2019	Applicable for China only
Version 1.0	25 Jan 2019	Applicable for China only

Overall Rationale for the Amendment 3

Protocol version 3.0 was updated to Version 4.0, based on comments from the Center for Drug Evaluation, additional clarifications, and correction of minor inconsistencies between sections. In addition, minor corrections, including typographical/grammatical errors, have been made. Changes made during development of Version 4.0 are clarified as follows:

**Section # and Name**	**Description of Change(s)**<br>(*new text is in bold and underlined, deleted text is struck-through)*	**Brief Rationale**
3.4 Subject Eligibility	Section 3.4 Subject Eligibility<br>3.4.1 Inclusion criteria<br>3.Have not received any systemic anti-tumor therapy for current recurrence or metastasis. ~~Except: For patients~~ **Exceptions: A patient** who ~~have~~ **has** received neoadjuvant/adjuvant ~~treatment, the patients~~ **therapy** can be screened if ~~the time~~ **his/her last neoadjuvant/adjuvant treatment is more than 6 months** from last ~~treatment to~~ relapse or ~~progression exceeds 6 months; for patients~~ **PD; a patient** who ~~have~~ **has** received ~~radical~~ **curative** concurrent chemoradiotherapy or ~~chemotherapy~~ **radiotherapy** for ~~oesophageal~~ **esophagus** cancer~~, the patients~~ can be screened if ~~the time~~**his/her last chemotherapy/radiotherapy is more than 6 months** from ~~last treatment to~~ relapse or ~~progression exceeds 12 months;~~**PD**. (Note: For radical concurrent chemoradiotherapy and neoadjuvant/adjuvant therapy (chemotherapy or chemoradiotherapy), any disease progression during treatment or within 6 months after discontinuation should be taken as a failure of first-line treatment, while any disease progression exceeding 6 months after discontinuation should not be taken as a failure of first-line treatment.)<br>3.4.2 Exclusion criteria<br>Subjects who meet any one of the following criteria cannot enter this study:	To clarity the subjects of first-line treatment failure so that more subjects have access to treatment.

Section # and Name	Description of Change(s)<br>(*new text is in bold and underlined, deleted text is struck-through)*	Brief Rationale
	1. BMI < ~~17.5~~**16.0** kg/m^2;	
3.3 Number of Subjects<br>8.1 Sample Size Estimation	Section 3.3 Number of Subjects<br>~~Approximately~~In this study, a total of about 540 subjects will be enrolled and at least ~~355~~**339** PFS events or ~~338~~**388** OS ~~are required in this study events~~ have to be observed. Group A (HLX10): 360 subjects<br>Group B (control): 180 subjects<br>Section 8.1 Sample Size Estimation<br>This study uses parallel dual primary endpoints of ~~progression-free survival (~~PFS~~)~~ and ~~overall survival (~~OS~~)~~, where one interim analysis **is planned for OS when a target number of PFS events is observed** and one final analysis ~~are~~**is** planned for ~~PFS~~**OS when a target number of OS events is observed**, while ~~two interim analyses and~~**only** one final analysis ~~are~~**is** planned for ~~OS~~**PFS**. To control the overall type I errors, the allocation of α is as follows:<br>PFS: $\alpha = 0.005$ (one-sided)<br>OS: $\alpha = 0.02$ (one-sided)<br>In this study, subjects are randomized into the treatment group and the control group at a ratio of 2:1. The sample size is based on the assumption that the median PFS is 5 months in the **of the** control group, i.e., placebo + chemotherapy (cisplatin + 5-FU), and~~that~~ the median PFS is 7.35 months in the HLX10 + chemotherapy group, i.e.,<br>an HR of the HLX10 + chemotherapy group to the control group is approximately 0.68. ~~The Group Sequential Design is used to perform the interim efficacy analysis when 66% event information is available, and the O'Brien-Fleming-like α-spending function of the Lan-Demets algorithm will~~	Revision based on the current project progress and external data. To clarify and define the populations to be analyzed in the study.

Section # and Name	Description of Change(s)<br>(*new text is in bold and underlined, deleted text is struck-through)*	Brief Rationale
	~~be used to control the overall~~**When** type I error ~~rate~~**α = 0.005** (one-sided~~).~~ ~~Assuming that the duration of~~**) and 24-month** enrollment ~~is 24 months~~**are assumed** and ~~that~~ the final analysis of PFS is ~~scheduled~~**planned** to be performed at approximately month 4 after the last subject is enrolled, a minimum of ~~355~~**339** PFS events needs to be observed to achieve a power of 80%. Assuming a drop-out rate of 10%, a total of ~~519~~**495** subjects ~~will be needed~~**need** to ~~enroll~~**be enrolled** in the 2 groups (~~346~~**330** in the HLX10 group and ~~173~~**165** in the control group).<br><br>Assuming that the median OS of the control group is 10 months and that the median OS of the HLX10 + chemotherapy group is 13.70 months, namely, the HR of the HLX10 + chemotherapy group to the control group is approximately 0.73. ~~Interim~~**One interim** efficacy will be analyzed by the Group Sequential Design when ~~approximately 50% and 75% event information~~ **target number of PFS events** is ~~available, respectively~~**observed**, and the O'Brien-Fleming-like α-spending function of the Lan-Demets algorithm will be used to control the overall type I error rate α = 0.02 (one-sided). Assuming that the duration of enrollment is 24 months and that the final analysis of OS is scheduled to be performed at approximately month 12 after the last subject is enrolled, a minimum of 388 OS events needs to be observed to achieve a power of 80% and a total of 540 patients needs to be enrolled in both groups (360 cases in the HLX10 group and 180 cases in the control group).<br><br>Considering the sample size required for PFS and OS evaluation, a total of 540 patients (360 in the HLX10 group and 180 in the control group) need to be enrolled in this study.	
5.1.13 Central laboratory assessments	Section 5.1.13 Central laboratory assessments	Update accompanying diagnostic manufacturers

<table>
<tr><th>Section # and Name</th><th>Description of Change(s)
(new text is in bold and underlined, deleted text is struck-through)</th><th>Brief Rationale</th></tr>
<tr><td></td><td>Table 10. PD-L1 detection reagent
<table>
<tr><th></th><th>Subject enrolment test</th><th>concomitant diagnosis study</th></tr>
<tr><td>Kit</td><td>~~Mnonoglonal~~**Monoclonal** Mouse Anti-Human PD-L1 Clone 22C3
~~Code M3653~~</td><td>PD-L1~~ Antibody Reagent (immunohistochemistry)~~**antibody reagent**
~~Clone WD160~~**(kits of partners designated by the sponsor)**</td></tr>
<tr><td>Manufacturer</td><td>Dako North America. Inc</td><td>~~Wuxi AppTec ZK (Suzhou) Bioscience Ltd.~~**Qualified partners designated by the sponsor**</td></tr>
</table></td><td>and kits according to actual situation.</td></tr>
<tr><td>8.3 Interim Analysis and Final Analysis</td><td>~~One~~ **In this study, one efficacy** interim analysis **is planned for OS when a target number of PFS events is observed** and one final analysis ~~will be performed in this study according to the PFS schedule, and two interim efficacy analyses and~~**is planned for OS when a target number of OS events is observed, while only** one final analysis ~~will be performed based on the OS schedule~~**is planned for PFS**. An O'Brien-Fleming-like α-spending function (Lan-DeMets approximation) is used to control the overall type I error rate.

The~~ first~~ interim analysis of ~~PFS and ~~OS is planned to be performed ~~when approximately 66% of~~**during** the ~~number~~**final analysis** of PFS ~~events is observed~~, with the primary objective to perform safety assessments, **and** superiority tests of PFS and OS endpoints, ~~and sample size reassessment. According to the O'Brien-Fleming α-spending function, the .~~ **The** significance level of ~~the~~ Log-Rank test ~~for~~**of PFS in** this analysis is 0.~~0011~~**01** (two-sided)~~ for PFS and 0.002 (two-sided) for OS.~~</td><td>Based on comments from the Center for drug evaluation, to cancel the interim analysis of PFS.</td></tr>
</table>

Section # and Name	Description of Change(s)<br>(*new text is in bold and underlined, deleted text is struck-through*)	Brief Rationale
	~~The second interim analysis of OS is planned to be performed during the final analysis of PFS, and the primary objective of this interim analysis is to assess safety and to test the superiority of PFS and OS endpoints.).~~ According to ~~the~~ O'Brien- Fleming α-spending function, the significance level of ~~the~~ Log-Rank test ~~for~~**of OS in** this analysis **(about 75% OS event information)** is 0.~~0096~~**014** (two-sided) ~~for PFS and 0.0137 (two-sided) for OS.);~~<br><br>The final analysis of OS is planned to be performed when a target number of OS events (approximately 388) ~~are~~**is** observed, and the significance level is 0.~~0355~~**036** (two-sided) for final OS **endpoint** analysis.;	
8.4.3.1 Analysis of primary efficacy endpoint	Inter-group comparison of PFS and OS will be performed using stratified Log-Rank test with the following stratification factors: PD-L1 expression level (CPS < 10 versus CPS ≥ 10), age (≥ 65 years versus < 65 years), and tumor ~~status~~**state** (locally advanced versus distant metastasis). ~~Significant levels~~**The significant level** of PFS ~~are~~**is** 0.~~0011~~**01 (two-sided)** **at the final analysis, and the significant levels of OS are 0.014** (two-sided) at the interim analysis and 0.~~0096 (two-sided) at the final analysis; significant levels of OS are 0.002 (two-sided) at the first interim analysis, 0.0137 (two-sided) at the second interim analysis, and 0.0355~~**036** (two-sided) at the final analysis; HR and its 95% CI will be estimated by stratified COX proportional risk model; the Kaplan Meier method will be used to estimate the median PFS/OS and 95% CI (Brookmeyer-Crowley method) with Kaplan-Meier curves plotted.<br><br>The ~~first~~ **final analysis of PFS and efficacy** interim analysis of ~~PFS and~~ OS endpoints ~~is~~**are** performed when ~~approximately 66% of the~~**a target** number of PFS events ~~(about 235 cases)~~ is observed, and at this time, when the P-Value (two-sided) is < 0.~~011 in the first interim analysis of PFS based on the Log-Rank test or the P-Value (two-sided) is < 0.002 in the first interim~~	To keep the study design consistent throughout the document.

Section # and Name	Description of Change(s)<br>(*new text is in bold and underlined, deleted text is struck-through)*	Brief Rationale
	~~analysis of OS, the difference in efficacy between HLX10 in combination with chemotherapy (cisplatin + 5-FU) and placebo in combination with chemotherapy (cisplatin + 5-FU) is statistically significant; or the second interim analysis of OS endpoints is performed during the final analysis of PFS, and at this time, when the P-Value (two-sided) is < 0.0096~~**01** in the final analysis of PFS based on the Log-Rank test or the P-Value (two-sided) is < ~~<~~ 0.~~0137~~**014** in the ~~second~~ interim analysis of OS, the difference in efficacy between HLX10 in combination with chemotherapy (cisplatin + 5-FU) and placebo in combination with chemotherapy (cisplatin + 5-FU) is statistically significant; or the final analysis of OS endpoints is performed when the target number of OS events (approximately 388 cases) is observed, and at this time, when the P-Value (two-sided) is < 0.~~0355~~**036** in the final analysis of OS based on the Log-Rank test, the difference in efficacy between HLX10 in combination with chemotherapy (cisplatin + 5-FU) and placebo in combination with chemotherapy (cisplatin + 5-FU) is statistically significant. When the actual number of observed events does not meet or exceeds the planned number of events at the interim/final analysis, the significant level can be adjusted to control the overall type I error rate using an O'Brien-Fleming-like α-spending function (Lan-DeMets approximation). The judgment boundaries and discontinuation criteria are detailed in the Statistical Analysis Plan (SAP). **For patients who continue to use other anti-tumor treatment after the end of study treatment, the subgroup analysis and other methods will be used to assess the real efficacy of the study treatment on the OS.**<br><br>With the α redistribution strategy of Group Sequential Holm Procedure, if the original hypothesis is rejected in the interim/final analysis for a primary efficacy endpoint (i.e., the actual P value at the analysis time point is less than the corresponding nominal significant level), all the initial α allocated to the primary efficacy endpoint will be recovered and allocated to another primary	

**Section # and Name**	**Description of Change(s)**<br>(*new text is in bold and underlined, deleted text is struck-through)*	**Brief Rationale**
	efficacy endpoint that does not reject the original hypothesis. And the O'Brien-Fleming-like α-spending function (Lan-DeMets approximation) will be used to recalculate and update significant levels at each analysis time point. For example, in the~~ first interim~~**final** analysis **of PFS endpoints**, if the P value of ~~PFS based on~~ Log-Rank test is less than 0.0~~0110~~**1**, while the P value of OS is greater than or equal to 0.~~002~~**014**, all α (one-sided, 0.005) initially allocated to PFS will then be recovered and allocated to OS, i.e., in this case, the total α update of OS is 0.025 (one-sided). The O'Brien Fleming-like α-spending function (Lan-DeMets approximation) will be used to recalculate the significant level of each interim analysis/final analysis of OS, and the actually calculated P value of OS will be compared with the updated significant level.	
8.4.3.2 Analysis of secondary efficacy endpoints	Section 8.4.3.2 Analysis of secondary efficacy endpoints<br>Objective response rate (ORR) is assessed by the IRRC as per RECIST v1.1 and iRECIST respectively and assessed by the investigator as per RECIST v1.1 and iRECIST respectively. ORR is defined as the percentage of subjects whose best overall responses are evaluated as complete response (CR/iCR) or partial response (PR/iPR). The stratified Cochran-Mantel-Haenszel (CMH)Miettinen-Nurminen method will be used to test the inter-group variationdifference in the ORRs and their 95% CIs. Stratification factors: Level of PD-L1 expression level (CPS < 10 versus CPS ≥ 10), age (≥ 65 years versus < 65 years), and tumor state (locally advanced versus distant metastasis).	To revised statistical methods.
15 Appendix 3	Quality of Life Scale EORTC QLQ-C30, EQ-5D-5L and EORTC QLQ-LC13 are updated.	To update the Quality of EQ-5D-5L.

Section # and Name	Description of Change(s)<br>(*new text is in bold and underlined, deleted text is struck-through)*	Brief Rationale
Throughout the protocol	Some editorial changes were made.	To keep the consistency throughout protocol.

Overall Rationale for the Amendment 2

Protocol Version 2.0 was updated to Version 3.0 based on the comments from the Center for Drug Evaluation that PFS and OS were recommended as primary endpoints. Additional clarifications, and correction of minor inconsistencies between sections, minor corrections, including typographical/grammatical errors have been made. Changes made during development of Version 3.0 are clarified as follows:

Section # and Name	Description of Change(s)<br>(*new text is in bold and underlined, deleted text is struck-through)*	Brief Rationale
3.2 Study Endpoints<br>3.2.1 Primary endpoint<br>3.2.2 Secondary endpoints<br>8.1 Sample Size Estimation<br>8.4.3.1 Analysis of primary efficacy endpoint	Section 3.2 Study Endpoints<br>3.2.1 Primary endpoints<br>• PFS (assessed by the IRRC as per RECIST v1.1)<br>• Overall survival (OS)<br>3.2.2 Secondary endpoints<br>~~• Overall survival (OS)~~<br>• Progression-free survival (PFS) (assessed by the IRRC as per iRECIST, and assessed by the investigator **<u>respectively</u>** as per RECIST v1.1 **<u>and iRECIST</u>**)	Based on the comments from the Center for Drug Evaluation that PFS and OS were recommended as primary endpoints.

Section # and Name	Description of Change(s)<br>(*new text is in bold and underlined, deleted text is struck-through)*	Brief Rationale
	● Objective response rate (ORR) (assessed by IRRC **as per RECIST v1.1 and iRECIST respectively, and by** ~~and~~ the investigator as per RECIST v1.1 **and iRECIST respectively)**<br>● Relationship between ~~PD-L1~~ expression **of PD-L1** in **tumor tissues**~~tumour tissue~~ and efficacy<br>● Duration of response (DOR) (assessed by IRRC as per RECIST v1.1 and iRECIST **respectively, and by the investigator as per RECIST v1.1 and**~~-~~**iRECIST respectively**)<br>● Incidence of adverse events (AEs) and serious adverse events (SAEs)<br>● Pharmacokinetics (PK): Concentration of HLX10 in serum<br>● Immunogenicity evaluation: Positive rate of anti-drug antibody (ADA**/NAb**)<br>● Relationship between microsatellite instability (MSI) and **tumor**~~tumour~~ mutation burden (TMB) and efficacy<br>● Quality of life assessment<br>3.3 Number of Subjects<br>Approximately **540**~~489~~ subjects and at least **355**~~336~~ PFS events **or 338 OS** are required in this study.<br>● Group A (HLX10): **360**~~326~~ subjects<br>● Group B (control): **180**~~163~~ subjects<br>8.1 Sample Size Estimation<br>**This study uses parallel dual primary endpoints of progression-free survival (PFS) and overall survival (OS), where one interim analysis and**	

Section # and Name	Description of Change(s)<br>(*new text is in bold and underlined, deleted text is struck-through)*	Brief Rationale
	**one final analysis are planned for PFS, while two interim analyses and one final analysis are planned for OS. To control the overall type I errors, the allocation of α is as follows:**<br><br>**PFS: α=0.005 (one-sided)**<br><br>**OS: α=0.02 (one-sided)**<br><br>**In this study, subjects are randomized into the** ~~The randomization ratio of~~ treatment group **and the**~~to~~ control group **at a ratio of** ~~in this study is~~ 2:1. **The sample**~~Sample~~ size is based on the **assumption**~~assumptions~~ that the median PFS **is 5 months in the control group, i.e.,**~~,in~~ placebo + chemotherapy (cisplatin + 5-FU), **and that the median PFS)** ~~group (control)~~ is **7.35**~~5~~ months **in**, ~~and~~ the ~~hazard ratio (HR) of~~ HLX10 + chemotherapy **group, i.e., an HR of the HLX10 + chemotherapy group to the control group is approximately 0.68. The Group Sequential Design is used to perform the interim efficacy analysis when 66% event information is available, and the O'Brien-Fleming-like α-spending function of the Lan-Demets algorithm will be used to control the overall type I error rate α=0.005 (one-sided). Assuming that the** ~~control is 0.7. And assuming the~~ duration of **enrollment**~~enrolment~~ is 24 months, and ~~that~~ the **final analysis**~~overall duration~~ of **PFS**~~study~~ is **scheduled to be performed** ~~29 months, it requires~~ at **approximately month 4 after the last subject is enrolled, a minimum of 355**~~least 336~~ PFS events **needs to be observed** to achieve ~~a85%~~ power **of 80%**~~.at an overall significance level α = 0.05 (two-sided).~~ Assuming ~~athat the~~ drop-out rate **of 10**~~is 20%~~, a total of **519 subjects will be needed to enroll in the 2 groups (346 in the HLX10 group and 173 in the control group).**<br><br>**Assuming that the median OS of the control group is 10 months and that the median OS of the HLX10 + chemotherapy group is 13.70 months,**	

Section # and Name	Description of Change(s) (*new text is in bold and underlined, deleted text is struck-through)*	Brief Rationale
	**namely, the HR of the HLX10 + chemotherapy group to the control group is approximately 0.73. Interim**~~-~~**efficacy will be analyzed by the Group Sequential Design when approximately 50% and 75% event information is available, respectively, and the O'Brien-Fleming-like α-spending function of the Lan-Demets algorithm will be used to control the overall type I error rate α=0.02 (one-sided). Assuming that the duration of enrollment is 24 months and that the final analysis of OS is scheduled to be performed at approximately month 12 after the last subject is enrolled, a minimum of 388 OS events needs to be observed to achieve a power of 80% and a total of 540**~~489~~ patient~~s needs to be enrolled in both groups (360 cases in the HLX10 group and 180 cases in the control group).Considering the sample size required for PFS and OS evaluation, a total of 540~~(~~326~~ patients **(360** in **the** HLX10 group and **180**~~163 patients~~ in **the control**~~placebo~~ group) need to be enrolled in **this study**~~the two groups.~~<br><br>8.3 Interim Analysis and Final Analysis<br><br>**In this study, an**~~An~~ Independent Data Monitoring Committee (IDMC) will be established **to conduct the** ~~in this study for~~ interim analysis. **The IDMC will be responsible for monitoring the study safety and efficacy data, assessing the study implementation quality, suggesting study design adjustments, and other emergency analyses and suggestions under blinded conditions and determined by the IDMC.**<br><br>~~One interim analysis and one final analysis will be performed in this study according to the PFS schedule, and~~ two ~~interim are scheduled, of which~~ **interim efficacy analyses and one final analysis will be performed based on the OS schedule. An O'Brien-Fleming-like α-spending function (Lan-DeMets approximation) is used to control** the overall type I error rate ~~is controlled by O'Brien-Fleming α-spending function (approximated by~~	

Section # and Name	Description of Change(s)<br>(*new text is in bold and underlined, deleted text is struck-through)*	Brief Rationale
	~~Lan-DeMets method).~~<br><br>The first interim analysis **of PFS and OS is planned to**~~will~~ be performed when approximately 66% **of the number of PFS events is observed, with the**~~subjects are enrolled. Its~~ primary objective ~~is~~ to perform **safety assessments, superiority tests of PFS and OS endpoints, and** ~~a revaluation of the~~ sample size **reassessment. According to the O'Brien-Fleming α-spending function, the**~~in a blind state with a~~ significance level **of the Log-Rank test for this analysis is α of 0.0011 (two-sided) for PFS and 0.002 (two-sided) for OS.**~~000001 (two-tailed).~~<br><br>The second interim analysis **of OS is planned to** ~~will~~ be performed **during**~~when 66% PFS cases are observed, so as to assess~~ the **final analysis of PFS,**~~safety~~ and **the**~~efficacy. Its~~ primary objective **of this interim analysis is to assess safety and to test the superiority of PFS and OS endpoints. According to the O'Brien-Fleming α-spending function, the**~~is the superiority assessment, with a~~ significance level ~~α of 0.012 (two-tailed) according to the O'Brien-Fleming boundary α-spending function~~ **of the Log-Rank test for this analysis is 0.0096 (two-sided) for PFS and 0.0137 (two-sided) for OS.**<br><br>**The final**~~Final~~ analysis of **OS is planned to**~~PFS will~~ be **performed**~~conducted~~ when **a**~~the~~ target number **of OS**~~(about 336 PFS~~ events **(approximately 388) are**)~~is~~ observed, **and the significance level is**~~using α~~ 0.**0355**~~046~~ (two-sided) **for final OS analysis.**<br><br>**Significance level for each analysis will be modified** based on the~~O'Brien-Fleming boundary α spending function~~ **actual number of PFS and OS events reached at the analytical time point. If an original hypothesis is rejected for an endpoint at the analytical time point, the recovery and redistribution of the α endpoint are performed and the significant level**	

Section # and Name	Description of Change(s) (*new text is in bold and underlined, deleted text is struck-through)*	Brief Rationale
	**of the other endpoint at each analytical time point is updated.**<br>8.4.3.1 Analysis of primary efficacy endpoint<br>**This study uses parallel dual primary endpoints of** PFS~~-~~**and OS which are**~~-~~assessed by~~-~~**the**~~-~~IRRC per RECIST v1.1 **criteria.**<br>**Progression-free survival (PFS) is assessed by IRRC as per RECIST v1.1.:** PFS is defined as **a period starting**~~the time~~ from randomization **and ending when death due to** ~~the first documented~~ PD or ~~death for~~ any ~~other reason~~ **(cause**~~,~~ whichever occurs first) ~~is noted for the first time.Data of subjects with .~~ **Subjects who** ~~neither PD~~**progress** nor ~~death~~**die** will be censored ~~on the day of the final valid tumor assessment. Data of surviving subjects not undergoing any tumor assessment during the study will be censored on the day of randomization. Data of subjects who have no PD reported and initiate any anti-tumor therapy not specified in the protocol will be censored on the dayat~~ **the date** of the last evaluable ~~tumor~~**tumour assessment. Subjects who neither have any on-study tumour assessment nor die will be censored at the date of randomization. Subjects who do not report any PD and initiate any anti-tumour therapy outside of the protocol will be censored at the date of their last evaluable tumour** assessment prior to the initiation of subsequent anti-~~tumor treatment. Censoring rules are detailed in the Statistical Analysis Plan (SAP).~~<br>~~Overall survival (OS) is defined as a period from randomisation through death regardless of causality. Data of patients without death record~~**tumour therapy. PFS between two groups** will be ~~censored on the last known survival date. Data of patients not providing any follow-up information will be censored on the day of randomization. Censoring rules are detailed in the Statistical Analysis Plan (SAP).~~<br>~~Inter-group comparison of PFS and OS will be performedcompared~~ using	

Section # and Name	Description of Change(s)<br>(*new text is in bold and underlined, deleted text is struck-through)*	Brief Rationale
	stratified ~~Log-Rank~~**log-rank** test, with~~ the following ~~stratification factors~~:~~ ~~PD-L1 a~~**s follows:** expression level **of PD-L1 (**CPS < 10~~%~~ versus CPS ≥ 10)~~,%),~~ age (≥ 65 years versus < 65 years), and ~~tumor~~**tumour** status (locally advanced versus ~~distant metastasis). Significant levels of PFS are 0.0011 (two-sided) at the interim analysis and 0.0096 (two-sided) at the final analysis; significant levels of OS are 0.002 (two-sided) at the first interim analysis, 0.0137 (two-sided)distantly metastatic), at the second interim analysis, and 0.0355 (two-sided) at the final analysis~~**significance level of two-sided 0.05;**~~;~~HR and~~ its~~ 95% **confidence interval** (CI) will be estimated ~~by~~**using the** stratified COX proportional **risk**~~hazard~~ model; the **Kaplan Meier method will be used to estimate the** median **PFS/OS and 95% CI (Brookmeyer-Crowley method) with Kaplan-Meier curves plotted.**<br><br>**The first interim analysis of PFS and OS endpoints is performed when approximately 66% of the number of PFS events (about 235 cases) is observed, and at this time, when the P-Value (two-sided) is < 0.011 in the first interim analysis of PFS based on the Log-Rank test or the P-Value (two-sided) is < 0.002 in the first interim analysis of OS, the difference in efficacy between HLX10 in combination with chemotherapy (cisplatin + 5-FU) and placebo in combination with chemotherapy (cisplatin + 5-FU) is statistically significant; or the second interim analysis of OS endpoints is performed during the final analysis of PFS, and at this time, when the P-Value (two-sided) is < 0.0096 in the final analysis of PFS based on the Log-Rank test or the P-Value (two-sided) is < 0.0137 in the second interim analysis of OS, the difference in efficacy between HLX10 in combination with chemotherapy (cisplatin + 5-FU) and placebo in combination with chemotherapy (cisplatin + 5-FU) is statistically significant; or the final analysis of OS endpoints is performed when the target number of OS events (approximately 388 cases) is observed, and at this time, when the P-Value (two-sided) is <**	

Section # and Name	Description of Change(s) (*new text is in bold and underlined, deleted text is struck-through)*	Brief Rationale
	**<u>0.0355 in the final analysis of OS based on the Log-Rank test, the difference in efficacy between HLX10 in combination with chemotherapy (cisplatin + 5-FU) and placebo in combination with chemotherapy (cisplatin + 5-FU) is statistically significant. When the actual number of observed events does not meet or exceeds the planned number of events at the interim/final analysis, the significant level can be adjusted to control the overall type I error rate using an O'Brien-Fleming-like α-spending function (Lan-DeMets approximation). The judgment boundaries and discontinuation criteria are detailed in the Statistical Analysis Plan (SAP).</u>**<br><br>**<u>With the α redistribution strategy of Group Sequential Holm Procedure, if the original hypothesis is rejected in the interim/final analysis for a primary efficacy endpoint (i.e., the actual P value at the analysis time point is less than the corresponding nominal significant level), all the initial α allocated to the primary efficacy endpoint will be recovered and allocated to another primary efficacy endpoint that does not reject the original hypothesis. And the O'Brien-Fleming-like α-spending function (Lan-DeMets approximation) will be used to recalculate and update significant levels at each analysis time point. For example, in the first interim analysis, if the P value of PFS based on Log-Rank test is less than 0.0011, while the P value of OS is greater than or equal to 0.002, all α (one-sided, 0.005) initially allocated to PFS will then be recovered and allocated to OS, i.e., in this case, the total α update of OS is 0.025 (one-sided). The O'Brien Fleming-like α-spending function (Lan-DeMets approximation) will be used to recalculate the significant level of each interim analysis/final analysis of OS, and the actually calculated P value of OS will be compared with the updated significant level.</u>**<br><br>8.4.3.2 Analysis of secondary efficacy endpoints	

Section # and Name	Description of Change(s)<br>(*new text is in bold and underlined, deleted text is struck-through)*	Brief Rationale
	~~OS~~**Progression-free survival (PFS) is assessed by IRRC as per iRECIST: PFS** is defined as ~~the time~~**a period starting** from **the** randomization ~~to~~**and ending with PD determined by iRECIST criteria or** death ~~for~~**of** any ~~cause. Patients without death records will be censored at the last date known alive. Patients who do not provide any follow-up information will be censored at the date of randomization. The OS is analysed in the same way as the primary efficacy endpoint.~~<br><br>~~PFS assessed by the investigator as per RECIST v1.1 and iRECIST~~ **reason (whichever occurs first).** The statistical method f**or PFS** is the same as **that for** the primary efficacy endpoint.<br><br>PFS assessed by the investigator **as** per RECIST v1.1: ~~The statistical method is~~ **and iRECIST respectively will be statistically analyzed using** the same **method** as ~~the~~**that for** primary efficacy ~~endpoint~~**endpoints.**<br><br>**Objective response rate (**ORR**) is** assessed by **the** IRRC **as per RECIST v1.1** and **iRECIST respectively and assessed by** the investigator as per RECIST v1.1: ~~Defined~~ **and iRECIST respectively. ORR is defined** as the ~~proportion~~**percentage** of subjects ~~who achieve either~~ **whose best overall responses are evaluated as complete response (**CR**/iCR) or partial response (**PR ~~as best overall response.~~**/iPR).** The stratified Cochran-Mantel-Haenszel (CMH) method will be used to test ~~the difference of~~ **inter-group variation in the** ORRs ~~between the two groups~~ **and** ~~estimate the odds ratio and~~**their** 95% ~~confidence interval~~**CIs.** Stratification factors ~~are as follows: Expression level:~~ **Level** of PD-L1 **expression (**CPS < 10~~%~~ versus CPS ≥ 10~~%),~~**),** age (≥ 65 years versus < 65 years), and ~~tumour status~~**tumor state** (locally advanced versus ~~distantly metastatic~~**distant metastasis).**<br><br>**Duration of response (**DOR**) is** assessed by **the** IRRC **as per RECIST v1.1** and **iRECIST respectively and evaluated by** the investigator **as** per	

Section # and Name	Description of Change(s)<br>(*new text is in bold and underlined, deleted text is struck-through)*	Brief Rationale
	RECIST v1.1: ~~Defined as the time~~ **and iRECIST respectively. DOR is defined as a period** from the first documentation of response (CR**/iCR** or PR**/iPR)** to the ~~first documentation of~~ PD or death, **(**whichever occurs first.**) determined by RECIST v1.1/iRECIST criteria. The** DOR ~~is analysed~~**will be analyzed** only for ~~patients achieving the~~**subjects whose** best overall ~~response of~~ **responses are evaluated as** CR**/iCR** or PR~~. Patients who do/~~**iPR. Data of patients** not ~~progress~~**experiencing PD or** ~~die~~**death** after **achieving** response will be censored ~~at~~**on the** date**day** of the ~~last tumour~~**final tumor** assessment; if no ~~tumour~~**tumor** assessment is performed after ~~achieving~~ response~~, censoring~~ **achievement, then data of such patients** will be ~~performed~~ ~~at~~**censored on** the ~~date~~**day** of t~~he tumour~~**tumor** assessment. ~~Median will be estimated using the~~ **when response is achieved. Censoring rules are detailed in the Statistical Analysis Plan (SAP). The** Kaplan-Meier method **is used to estimate the median DOR,** and **the** Kaplan-Meier ~~curves~~**curve** will be plotted.<br><br>~~The DOR assessed by IRRC per iRECIST: The statistical method is the same as the DOR assessed per RECISTv1.1.~~	
8.4.5 Pharmacokinetics and immunogenicity analysis	**Pharmacokinetic**~~The pharmacokinetic~~ parameters will be **calculated**~~analysed by descriptive statistics~~ using WinNonlin **7.0 (or above).**~~for serum drug concentrations at each visit point.~~<br><br>The ADA **(NAb)** positive rate at each visit time point should be summarized.	To clarify the immunogenicity analysis.
3.4.2 Exclusion criteria	3.4.2 Exclusion criteria<br><br>9. Active autoimmune diseases or history of autoimmune diseases (such as interstitial pneumonia, colitis, hepatitis, hypophysitis, vasculitis, nephritis, hyperthyroidism, and hypothyroidism, including but not limited to these diseases or syndromes); except patients with leucoderma or cured childhood	To clarify self-immune diseases that did not require systemic immunosuppressive therapy, and exclusion criteria 14 was

Section # and Name	Description of Change(s)<br>(*new text is in bold and underlined, deleted text is struck-through)*	Brief Rationale
	asthma/allergy and not requiring any intervention in adulthood; autoimmune mediated hypothyroidism treated with stable-dose thyroid hormone replacement therapy; type I diabetes treated with a stable dose of insulin; **<u>subjects who are in a stable state and do not require systemic immunosuppressive therapy (including corticosteroids);</u>**<br>11. Subjects have uncontrolled clinical **<u>heart and</u>** cardiovascular symptoms or diseases, including but not limited to: For example: (1) heart failure above NYHA Grade II; (2) unstable angina pectoris; (3) prior myocardial infarction **<u>and cerebral infarction</u>** within 6 months; (4) clinically significant supraventricular or ventricular arrhythmia without clinical intervention or inadequately controlled after clinical intervention;<br>~~14. Subjects with any known active or suspected autoimmune disease. Subjects in stable state and requiring no systemic treatment with immunosuppressive agents are included;~~	consolidated into exclusion criteria 9.
Throughout the protocol	Some editorial changes were made.	To keep the consistency throughout protocol.

Overall Rationale for the Amendment 1

Protocol Version 1.0 was updated to Version 2.0 based on the IB update of 10 and to supplement HLX10 toxicity management information and treatment information. Additional clarifications, and correction of minor inconsistencies between sections, minor corrections, including typographical/grammatical errors have been made. Changes made during development of Version 2.0 are clarified as follows:

Section # and Name	Description of Change(s)<br>(*new text is in bold and underlined, deleted text is struck-through)*	Brief Rationale
Title page	A Randomized, Double-Blind, ~~Multicenter~~**Multicentre**, Phase 3 Clinical Study to Compare HLX10 (Recombinant Humanized Anti-PD-1 Monoclonal Antibody Injection) versus Placebo in Combination with Chemotherapy (Cisplatin + 5-FU) as First-Line Therapy in Patients with Locally Advanced/Metastatic ~~Esophageal~~**Oesophageal** Squamous Cell Carcinoma (ESCC)	Rewording to avoid possible misunderstanding.
1.2 Rationale for Conduct of Study and Dose Selection<br>1.2.1 Nonclinical overview of HLX10	In-vitro ~~Pharmacodynamics~~**pharmacodynamics** of HLX10<br>A series of in-vitro PD studies of HLX10 versus positive control nivolumab showed that: HLX10 bound to the surfaces of activated T cells expressing PD-1 and had the effect to block the binding of PD-1 to its ligand PD-L1 or PD-L2. Both binding and blocking effects were dose-dependent. The in-vitro mixed leucocyte reaction (MLR) analysis of HLX10 showed that HLX10 blocked the immunosuppressive effect depending on the binding pathway of PD-1 to its ligands to further stimulate activated CD4+ T cells, so that the T cells were improved in cell reproductive capacity and produced more IL-2 cytokines. This phenomenon demonstrated dose dependence in both HLX10 and positive control nivolumab groups.<br>Moreover, in order to study the PD-1 receptor occupancy of HLX10 on human T cells, different doses of HLX10 ([redacted] were preincubated with whole blood form [redacted] healthy subjects to simulate the injection of HLX10 into human blood. The experiment results showed: The PD-1 receptor occupancy on CD3+ T cells increased with the ~~preincubated~~**increase of** HLX10 dose **for preincubation.** In [redacted] ~~out of~~**the** [redacted] healthy subjects, **once the serum concentration of HXL10 reached**[redacted]**,** the PD-1 receptor occupancy on CD3+ T cells ~~**had already**~~ reached more than [redacted] ~~once the serum concentration of HXL10 reached~~ [redacted] **in** [redacted] **of them.**	To add the latest reported clinical study results.

**Section # and Name**	**Description of Change(s)**<br>(*new text is in bold and underlined, deleted text is struck-through)*	**Brief Rationale**
1.2.2 Clinical studies of HLX10	At present, HLX10 has been approved by FDA, TFDA, and China FDA successively to conduct phase 1 clinical dose-escalation study. A total of four dose groups (0.3, 1, 3, and 10 mg/kg, Q2W) will be ~~studied~~**tested**, and **about** up to 30 subjects will be ~~enrolled. The enrollment of~~**included. Up to now, all patients in** the ~~third~~**four** dose ~~group has been~~**groups have** completed~~, and~~ the ~~fourth~~ dose ~~group is under initiation.~~**escalation and enrolment, as well as DLT assessment. Now, expansion and enrolment are planning to be performed for dose group 4 (10 mg/kg).**	To updated according to the latest phase I research progress.
3.4 Subject Eligibility<br>3.4.1 Inclusion Criteriacriteria	3.4.1 Inclusion Criteriacriteria<br>2. Histologically ~~confirmed radically unresectable~~ diagnosed with locally -advanced~~/relapsed (have not received radical treatment such as radical chemoradiotherapy or radical radiotherapy)~~ (determined by local investigator)/recurrent or distantly metastatic ESCC ~~(patients with histologically confirmed~~including gastro-oesophageal junction) that is not resectable or cured by chemoradiotherapy (patient with adenosquamous carcinoma with predominantly squamous cell carcinoma ~~of the gastroesophageal junction are eligible~~can be enrolled);<br>3. Have not received ~~prior~~**any** systemic anti~~-tumor treatment. Excluding: subjects~~ **tumour therapy for current recurrence or metastasis. Except: For patients** who **have** received neoadjuvant/adjuvant treatment**, the patients** can be screened if the time from last treatment to relapse or progression exceeds 6 months; ~~subjects~~**for patients** who **have** received radical concurrent chemoradiotherapy or radiotherapy for ~~esophageal~~**oesophageal** cancer, **the patients** can be screened if the time from last treatment to relapse or progression exceeds 12 months; **(Note: For radical concurrent chemoradiotherapy and neoadjuvant/adjuvant therapy (chemotherapy or chemoradiotherapy), any disease progression**	To clarify "the concept of first-line treatment failure" and optimize the description based on the actual situation.

Section # and Name	Description of Change(s)<br>(*new text is in bold and underlined, deleted text is struck-through)*	Brief Rationale
	**during treatment or within 6 months after discontinuation should be taken as a failure of first-line treatment, while any disease progression exceeding 6 months after discontinuation should not be taken as a failure of first-line treatment.) ;**<br>3.4.2 Exclusion ~~Criteria~~**criteria**<br>~~16. Hepatitis B (HBsAg or HBcAb test positive, and HBV-DNA ≥ 200 IU/mL or 103 copies/mL), hepatitis C (HCV antibody test positive, and HCV-RNA higher than the lower limit of detection of the analysis method);~~<br>~~16.~~ **17. With HBsAg (+) and/or HBcAb (+), and HBV-DNA ≥ 500 IU/mL or 2500 copies/mL at the time of enrolment. Any subject with a measurement above the said criteria can only be enrolled if the measurement decreases to the normal range for at least 2 weeks after antiviral treatment, and he/she continues to receive the antiviral treatment throughout the study. Any subject who has previously needed to receive or were receiving antiviral treatment at the time of screening can only be enrolled if he/she continues to receive antiviral treatment throughout the study, even if HBV-DNA meets the inclusion criteria. Hepatitis C (HCV antibody tested positive and HCV-RNA positive);**	
1.3 Schedule of Activities (SoA) footnote #11<br>8.1 Tests and Evaluations during the Study<br>Laboratory Tests<br>3.4.3 Withdrawal criteria	3.4.3 Withdrawal ~~Criteria~~**criteria**<br>The subject can withdraw from this study at any time without any punishment or any impact on his/her future medical care.<br>The subject must withdraw from the study in the following conditions:<br>● Withdrawal of informed consent<br>● If the subject withdraws informed consent but is still willing to receive end-of-treatment visit or survival follow-up, this will be recorded on the ICF	To amended pregnancy to mandatory withdrawal criteria.

Section # and Name	Description of Change(s) (*new text is in bold and underlined, deleted text is struck-through)*	Brief Rationale
	kept by the investigator and signed and dated by the subject; relevant data collected at follow-up will be recorded in the electronic data capture (EDC) system. If the subject withdraws informed ~~consent and is unwilling to provide subsequent information, no subsequent assessments will be performed and no additional information will be collected.~~<br>**<u>Pregnancy</u>**<br>The sponsor ~~may save~~ and ~~continue to use~~ the~~ information collected before withdrawal of informed consent.~~<br>~~The sponsor and/or the investigator can~~ **<u>investigators may</u>** ask the subject **<u>upon discussion to discontinue the treatment due</u>** to ~~withdraw from this study for~~ the following reasons ~~after discussing with~~ , **<u>or withdraw from</u>** the ~~subject~~**<u>study depending on protocol violation:</u>**<br>● AEs<br>● Inappropriate to continue with treatment as determined by the investigator<br>● Poor compliance, unable to return to visit on time<br>● Major protocol violations<br>~~● Pregnancy~~	
4.4 HLX10-Related Toxicity and Management	**<u>4.4 HLX10-Related Toxicity and Management</u>**<br>**<u>If immunotherapy-related toxicity is observed in a subject, HLX10-based measures can be taken according to the following criteria, including permanent discontinuation, suspension, and continuation of HLX10 therapy.</u>**	To supplement HLX10 toxicity management information and treatment information.

**Section # and Name**	**Description of Change(s)** (*new text is in bold and underlined, deleted text is struck-through)*	**Brief Rationale**
	**Generally (except for hepatotoxicity), for any subject experiencing a moderate (grade 2) or severe (grade 3 or greater) immune-related adverse event (irAE), the investigational products should be suspended until symptoms or toxicities recover to grade 1 or less before re-administration. For patients reporting grade 2 irAEs, if symptoms do not improve within 1 week, corticosteroids (0.5 mg/kg/day prednisone or equivalent) should be given. In the event of grade 3 or greater irAEs, HLX10 therapy should be stopped immediately and the subject should be treated with a high dose of corticosteroid (1–2 mg/kg/day prednisone or equivalent). Gradually reduce corticosteroid dosage for at least 1 month until symptoms alleviate to grade 1 or below.**<br><br>**4.4.1 Cutaneous adverse events**<br><br>**Skin rashes and pruritus are the most commonly seen and earliest cutaneous AEs related to immunotherapies. The following table provides the medications and therapies in response to a cutaneous irAE:**<br><br>**Table 2. Management of cutaneous toxicity**	

**Grade of rashes**	**Management**	**Follow-up**
Grade 1–2:<br>Coverage ≤ 30% of body surface area (BSA)	Continue the treatment as per the protocol<br>for grade 1 skin lesions.<br>For grade 2 skin lesions or	**If the irAE persists for > 1 week or reoccurs, then**<br>• Consider biopsy.<br>• Start the treatment with methylprednisolone 0.5–1 mg/kg/day by i.v. or its equivalent by oral administration.

Section # and Name	Description of Change(s) (*new text is in bold and underlined, deleted text is struck-through)*	Brief Rationale
	<table><tr><td></td><td>symptomatic grade 1 skin lesions,<br>delay the treatment as per the protocol.</td><td>• Once the condition is improved, gradually reduce the dose of steroids for at least 1 month.<br>• Consider prophylactic antibiotics.<br>• If the steroid can be reduced to a prednisone-equivalent dose less than 10 mg/day, restart the treatment as per the protocol.<br>**If symptoms worsen:** Treat as an event of grade 3 or greater.</td></tr><tr><td>Grade 3–4:<br>Coverage > 30% BSA; life-threatening</td><td>• Stop the treatment as per the protocol.<br>• Consult the Dermatology Division.<br>• Consider biopsy.<br>• Start the treatment with methylprednisolone 1–2 mg/kg/day by i.v. or equivalent.</td><td>**If the condition is improved to grade 1:**<br>• Gradually reduce the dose of steroids for at least 1 month.<br>• Restart the treatment as per the protocol.<br>• Add prophylactic antibiotics.</td></tr></table><br>**<u>4.4.2 Endocrine disorders</u>**<br>**<u>Immune checkpoint inhibitors tend to cause endocrine disorders. The most common endocrine disorders related to immune checkpoint</u>**	

Section # and Name	Description of Change(s) (*new text is in bold and underlined, deleted text is struck-through)*	Brief Rationale

inhibitors are autoimmune thyroid diseases, hypophysitis, and adrenal insufficiency. The following table provides the medications and therapies in response to an endocrine disorder:

Table 3. Management of endocrine disorders

<table>
<tr><th>Seriousness</th><th colspan="2">Management</th></tr>
<tr><td>Asymptomatic TSH elevations</td><td colspan="2">Continue the treatment as per the protocol.
Consult an endocrinologist if TSH is < 0.5 × LLN or > 2 × ULN, or beyond normal range in both of 2 subsequent measurements.</td></tr>
<tr><td>Symptomatic endocrine disorders</td><td>Evaluate the endocrine function
Consider hypophysis scan
Symptomatic endocrine disorders accompanied with abnormalities in laboratory data/hypophysis scan:
• Delay the treatment as per the protocol
• Prednisone, 1-2 mg/kg/day, i.v. or p.o.
• Start appropriate hormone therapy
No abnormalities in laboratory data/hypophysis scan:
Repeat laboratory tests in 1–3 weeks/MRI in 1 month.</td><td>If the condition is improved (with or without hormone replacement):
• Gradually reduce the dose of steroids for more than 1 month and consider prophylactic antibiotics for opportunistic infections.
• Resume the treatment as per the protocol.
• For patients with adrenal insufficiency, corticosteroid maintenance therapy may be required.</td></tr>
</table>

Section # and Name	Description of Change(s) (*new text is in bold and underlined, deleted text is struck-through)*	Brief Rationale

Suspected adrenal crisis	• Stop the treatment as per the protocol<br>• Exclude septicaemia<br>• Intravenous pulse therapy with corticosteroids and mineralocorticoids<br>• Intravenous infusion<br>• Consult an endocrinologist<br>• If adrenal crisis is excluded, treat symptomatic endocrine disorders as above.

4.4.3 Pneumonia

Pneumonia is an uncommon but potentially serious or fatal complication during treatment with immune checkpoint inhibitors. The following table provides the medications and therapies in response to an immune-mediated pneumonia:

Table 4. Management of pneumonia

**CTCAE Grade**	**Management**	**Follow-up**
Grade 1:<br>With radiographic changes only	• Consider delaying the treatment for 2–4 weeks<br>• Monitor the symptoms every 2–3 days<br>• Consider consulting the Division of Pulmonary Medicine and the Division of Infective Diseases	Repeat imaging at least every 3 weeks<br>**If symptoms or imaging results worsen:**<br>Treat as an event of grade 2 or greater

Section # and Name	Description of Change(s) (*new text is in bold and underlined, deleted text is struck-through)*	Brief Rationale

Grade 2:<br><br>Mild to moderate symptoms	• Delay the treatment<br>• Consult the Division of Pulmonary Medicine and the Division of Infective Diseases<br>• Monitor the symptoms daily and consider hospitalisation<br>• Start high-dose corticosteroid therapy (1–2 mg/kg/day)	Repeat imaging every 1–3 days<br><br>**If the symptoms improve:**<br><br>Gradually reduce the dose of steroids for more than 1 month, and resume the treatment after the toxicity reduces to grade 1 or less.<br><br>**If the symptoms do not improve or even worsen after two weeks:**<br><br>Treat as an event of grade 3 or greater
Grade 3 or greater:<br><br>Severe symptoms; severe anoxia; life threatening	• Stop the treatment as per the protocol<br>• Hospitalisation<br>• Consult the Division of Pulmonary Medicine and the Division of Infective Diseases<br>• Start high-dose corticosteroid therapy (1–2 mg/kg/day)<br>• Add prophylactic antibiotics for opportunistic infections<br>• Consider bronchoscopy and lung biopsy	**If the symptoms improve to the baseline:**<br><br>Gradually reduce the dose of steroids for at least 6<br><br>weeks<br><br>**If the symptoms fail to improve or**<br><br>**worsen in 48 h:**<br><br>Use additional immunosuppressants, such as infliximab, intravenously injectable immunoglobulin (IVIG), and mycophenolate mofetil

Section # and Name	Description of Change(s) (*new text is in bold and underlined, deleted text is struck-through)*	Brief Rationale

4.4.4 Colitis

Gastrointestinal AEs often occur in subjects receiving immune checkpoint inhibitor therapy. The most common AE is diarrhoea caused by immune-mediated colitis. The following table provides the medications and therapies in response to a gastrointestinal irAE:

Table 5. Management of gastrointestinal AEs

Grade of diarrhoea	Management	Follow-up
Grade 1:<br>Diarrhoea < 4 times/day, beyond the baseline; none<br>Symptomatic colitis	• Continue the treatment as per the protocol.<br>• Symptomatic therapy<br>• Change diet	Monitor the symptoms closely for worsening<br>Inform the patient and immediately report such worsening<br>**In the event of worsening:**<br>Treat as an event of grade 2 or greater
Grade 2:<br>Diarrhoea 4–6 times/day, beyond the baseline;<br>Colitis: abdominal pain and/or bloody stool	• Delay the treatment<br>• Symptomatic therapy<br>• Intravenous infusion support<br>• Consider hospitalisation for intravenous infusion and	**If the condition is improved to grade 1:**<br>Resume the treatment<br>**If the AE persists for ≥ 3–5 days or reoccurs:**<br>Start corticosteroid therapy (0.5–1 mg/kg/day)<br>**If symptoms improve to grade 1 after corticosteroid therapy:**

Section # and Name	Description of Change(s) (*new text is in bold and underlined, deleted text is struck-through)*	Brief Rationale
	<table><tr><td></td><td>monitoring if symptoms worsen</td><td>• Gradually reduce the dose of steroids for more than 1 month.<br>• Add prophylactic antibiotics for opportunistic infections.<br>**If the AE persists for > 3–5 days or worsens after the steroid therapy:**<br>Treat as an event of grade 3 or greater</td></tr><tr><td>Grade 3 or greater<br>Diarrhoea ≥ 7 times/day, beyond the baseline; incontinence<br>Colitis: serious abdominal pain, peritoneal irritation signs, bowel obstruction, pyrexia, perforation, etc.</td><td>• Stop the treatment as per the protocol.<br>• Start high-dose corticosteroid therapy (prednisone 1–2 mg/kg/day or equivalent)<br>• Hospitalisation<br>• Add prophylactic antibiotics<br>• Consider colonoscopy</td><td>**If the symptoms improve:**<br>Continue the treatment until grade ≤ 1, and then reduce the dose gradually for more than 1 month.<br>**If the AE persists for > 3–5 days or reoccurs:**<br>• Add infliximab or its equivalent (if it has no contraindication)<br>• Consider IVIG<br>• Avoid the use of infliximab in the case of perforation or septicaemia</td></tr></table><br>**4.4.5 Nephrotoxicity**	

Section # and Name	Description of Change(s) (*new text is in bold and underlined, deleted text is struck-through)*	Brief Rationale
	**<u>Subjects received immunotherapy should be monitored for signs and symptoms of nephritis, glomerulonephritis, and renal insufficiency. Asymptomatic serum creatinine elevation is most likely to occur. Therefore, serum creatinine levels should be periodically monitored to detect nephrotoxicity. The following table provides the medications and therapies in response to nephrotoxicity:</u>**	

<u>Table 6. Management of renal adverse events</u>

Grade of serum creatinine elevation	Management	Follow-up
Grade 1:<br>Creatinine > ULN and > baseline but ≤ 1.5 × baseline	• Continue the treatment as per the protocol<br>• Monitor the serum creatinine every week	**If the symptoms recover to the baseline:**<br>Resume routine serum creatinine monitoring as per the protocol<br>**In the event of worsening:**<br>Treat as an event of grade 2 or greater
Grade 2–3:<br>Creatinine > 1.5 × baseline and ≤ 6 × ULN	• Delay the treatment as per the protocol<br>• Monitor the serum creatinine level every 2–3 days<br>• Start the treatment with methylprednisolone 0.5–1 mg/kg/day by i.v.,	**If the symptoms recover to grade 1:**<br>• Gradually reduce the dose of steroids for more than 1 month<br>• Consider adding prophylactic antibiotics for opportunistic infections

**Section # and Name**	**Description of Change(s)** (*new text is in bold and underlined, deleted text is struck-through)*	**Brief Rationale**

	or equivalent by oral administration	and resume routine serum creatinine monitoring as per the protocol<br>**If increased creatinine lasts for > 5 days or worsens:**<br>Treat as an event of grade 4
Grade 4:<br>Creatinine > 6 × ULN	• Stop the treatment as per the protocol<br>• Monitor the serum creatinine level daily<br>• Start treatment with methylprednisolone 1–2 mg/kg/day or its equivalent by i.v.<br>• Consult an nephrologist<br>• Consider renal biopsy	**If the symptoms recover to grade 1:**<br>Gradually reduce the steroid dose for at least 1 month and add prophylactic antibiotics

4.4.6 Hepatotoxicity

Hepatotoxicity is a rare irAE in immunotherapy. However, severe hepatotoxicity may lead to fatal hepatic failure. When hepatotoxicity is suspected, viral or other drug-induced hepatitis must be ruled out. The following table provides the medications and therapies in response to hepatotoxicity:

Table 7. Management of hepatotoxicity

Section # and Name	Description of Change(s) (*new text is in bold and underlined, deleted text is struck-through*)	Brief Rationale

Grade of transaminase elevation	Management	Follow-up
Grade 1:<br>AST or ALT > ULN to 2.5 × ULN or total bilirubin > ULN to 1.5 × ULN	Continue the treatment as per the protocol	Continue the weekly monitoring<br>**In the event of worsening:**<br>Treat as an event of grade 2 or greater
Grade 2:<br>AST or ALT > 2.5 × ULN to ≤ 5 × ULN or total bilirubin > 1.5 × ULN to 3 × ULN	Delay the treatment as per the protocol<br>Increase monitoring frequency to once every 3 days.	**If the symptoms recover to the baseline:**<br>Resume the routine monitoring and treatment as per the protocol.<br>**If AST or ALT increases for > 5–7 days or worsens:**<br>• Take prednisone 0.5–1.0 mg/kg/day or equivalent orally, and gradually reduce the dose of steroids for at least 1 month when the liver function recovers to grade 1 or baseline.<br>• Consider prophylactic antibiotics<br>• Resume the treatment as per the protocol

Section # and Name	Description of Change(s) (*new text is in bold and underlined, deleted text is struck-through)*	Brief Rationale
	(see nested content below)	

Grade 3–4:<br>AST or ALT > 5 × ULN or total bilirubin > 3 × ULN	• Permanently discontinue the treatment as per the protocol<br>• Monitor liver function tests (LFTs) every 1–2 days<br>• Start the treatment with methylprednisolone 1–2 mg/kg/day by i.v. or equivalent.<br>• Add prophylactic antibiotics<br>• Consult a gastroenterologist	**If the symptoms recover to grade ≤ 2:**<br>Gradually reduce the dose of steroids for at least 1 month.<br>**If the symptoms fail to improve or worsen or rebound in 3–5 days:**<br>• Add mycophenolate mofetil 500 mg–1 g, twice a day<br>• Consider using additional immunosuppressants<br>**<u>Do not use infliximab for immunotherapy-related hepatitis.</u>**

<u>4.4.7 Neuropathy</u>

<u>A wide range of neurological syndromes are associated with immunotherapy, among which the Guillain-Barré syndrome is particularly significant. Other neurological complications include myasthenia gravis, posterior reversible encephalopathy syndrome, enteric neuropathy, aseptic meningitis, and autoimmune encephalitis. The following table provides the medications and therapies in response to neuropathy:</u>

<u>Table 8. Management of neuropathy</u>

Section # and Name	Description of Change(s) (*new text is in bold and underlined, deleted text is struck-through)*	Brief Rationale

**Grade of neuropathy**	**Management**	**Follow-up**
Grade 1:<br>With no or mild symptoms	Continue the treatment as per the protocol	Continue the monitoring.<br>**In the event of worsening:**<br>Treat as an event of grade 2 or greater
Grade 2:<br>Moderate symptoms: limiting activities of daily living (ADL)	• Delay the treatment as per the protocol<br>• Symptomatic therapy<br>• Consider prednisone 0.5–1 mg/kg/day, i.v. or p.o.	**If the symptoms recover to the baseline:**<br>Resume the treatment as per the protocol<br>**If symptoms worsen:**<br>Treat as an event of grade 3 or greater
Grade 3–4:<br>Severe symptoms: limiting self-care ADL; life-threatening	• Stop the treatment as per the protocol<br>• Consult a neurologist<br>• Symptomatic therapy<br>• Start the treatment with methylprednisolone 1–2 mg/kg/day i.v. or equivalent.<br>• Add prophylactic antibiotics	**If the symptoms are improved to grade 2:**<br>Gradually reduce the dose of steroids for at least 1 month.<br>**If the symptoms worsen or atypical symptoms appear:**<br>Consider IVIG or plasmapheresis or other immunosuppressive agents

Section # and Name	Description of Change(s) (*new text is in bold and underlined, deleted text is struck-through)*	Brief Rationale
	**4.4.8 Other immune-related adverse events**<br><br>**Other immune-related adverse events include cardiac toxicity, haemotoxicity, and oculopathy. All these adverse events are treated as detailed in Paragraph 3 of Section 4.4. Investigators should be highly vigilant for various immune-mediated manifestations. In general, timely treatment with corticosteroids usually has good outcomes and allows the subject to continue with the study.**<br><br>**4.5 Infusion-Related Reactions and Management**<br><br>**Acute infusion reactions (including cytokine release syndrome, angioedema, or anaphylaxis) are different from common anaphylaxis caused by drugs, although some of the characteristics are common to both. Infusion-related reactions often occur during or shortly after drug infusion, and generally relieve within 24 h after completion of the infusion. Symptoms and signs of infusion reactions include anaphylaxis/hypersensitivity, drug-induced fever, arthralgia, bronchospasm, cough, vertigo, and dyspnoea. Serious anaphylaxis may require epinephrine treatment. Infusion-related reactions should be graded according to the NCI CTCAE v5.0. The recommended treatments are listed in Table 10, but the final treatment is determined by the investigators based on their clinical experience.**<br><br>**Table 9. Management of acute infusion-related reactions**	

Section # and Name	Description of Change(s) (*new text is in bold and underlined, deleted text is struck-through)*	Brief Rationale

CTCAE Grade	Management	Post-infusion prophylactic drugs
Grade 1: Mild reaction; transient reaction; infusion interruption not indicated; intervention not indicated	Strengthen monitoring of vital signs until the subject is considered medically stable by investigators.	None
Grade 2: Require infusion interruption but responds promptly to symptomatic therapy (e.g., antihistamines, NSAIDs, narcotics, intravenous fluids); and prophylactic medications indicated for ≤ 24 h	• Stop the infusion and monitor the symptoms.<br>• Additional treatments, such as intravenous fluids, antihistamines, acetaminophen, or narcotics.<br>• If the symptoms subside within one hour after drug discontinuation, restart the infusion at 50% of the original infusion rate.<br>• If symptoms do not subside, continuous monitoring and hospitalisation for further treatment will be required.<br>• Although adequate prophylactic drugs have been administered, permanent discontinuation of relevant study drugs should be	Prophylactic use of antihistamines (such as diphenhydramine) and acetaminophen, or determined according to the guidelines of the study site.

Section # and Name	Description of Change(s) (*new text is in bold and underlined, deleted text is struck-through)*	Brief Rationale

	performed for the subjects experiencing grade 2 toxicity.	
Grade 3:<br>Prolonged (e.g., not rapidly responsive to symptomatic medication and/or brief interruption of infusion); recurrence of symptoms following initial improvement; and hospitalisation indicated for other clinical sequelae<br>(e.g. kidney damage, pulmonary infiltrates)	• Stop infusion<br>• Additional treatment required: intravenous fluids, antihistamines, NSAIDs, acetaminophen, anaesthetics, oxygen therapy, vasopressors, glucocorticoids, and epinephrine.<br>• Hospitalisation for further treatment is required.<br>• The subject should immediately stop receiving treatment and permanently stop receiving the related study drug.	No subsequent infusion
Grade 4:<br>Life-threatening consequences; urgent intervention indicated		

Section # and Name	Description of Change(s)<br>(*new text is in bold and underlined, deleted text is struck-through)*	Brief Rationale
5.1.2 Prior and concomitant therapy<br>5.1.3 Adverse events	5.1.2 Prior and concomitant therapy<br>Prior ~~treatment history should be collected~~**medications taken** from 30 days ~~prior to~~**before** screening visit**, to the time of signing ICF will be recorded** and ~~concomitant treatment history should be~~ collected. **Concomitant medications taken** from **the time of** signing~~ the~~ ICF **to the end of** safety follow-up~~.~~ **will be recorded and collected.** Concomitant ~~medications related to SAEs need to be~~**therapies associated with AE are** recorded ~~until~~**to** 90 days after **the** last dose~~ of study drug.~~. All prior and concomitant therapies (including traditional Chinese ~~preparations) should~~**medicine products) are required to** be ~~recorded~~**documented** in~~ the~~ eCRF.<br>5.1.3 ~~AEs~~ **Adverse events**<br>All AEs ~~should be~~**and SAEs are** recorded ~~from~~**since** the **time of** signing ~~of~~ ICF,~~ and AEs after treatment should be recorded.~~ **AEs and SAEs are documented** until 90 days after ~~last dose~~t**he completion** of **the study treatment or the initiation of a new anti-tumour treatment (whichever occurs first); and thereafter, only SAEs related to the** study drug **(HLX10/placebo) will be documented.**	
5.1.12 Local laboratory tests	5.1.12 Local ~~Laboratory Tests~~**laboratory tests**<br>Local laboratory tests will be performed at the site's local laboratory. ~~Hematology, blood biochemistry~~**Haematology, serum chemistry,** coagulation ~~and~~**test**, urinalysis~~ tests will,~~ **cardiac markers, pregnancy test, thyroid function test, trypsin, and virological test are included. Apart from virological and trypsin tests, other tests in the screening period should be performed within 7 days** ~~before~~**prior to the** first dose ~~at screening, and .~~ **During the treatment, haematology, serum chemistry, coagulation function, cardiac markers, and urinalysis should be tested**	

Section # and Name	Description of Change(s) (*new text is in bold and underlined, deleted text is struck-through)*	Brief Rationale
	within 3 days prior to **<u>the dose of </u>**each cycle ~~during treatment period; when~~**<u>and pregnancy and thyroid function tests should be conducted within 3 days before dosing in every 2 cycles. When</u>** the above laboratory tests ~~are arrangedand~~ study medication are scheduled on the same day ~~as administration of study drug, administration can only,~~ **<u>the results of such tests must be obtained before the scheduled study medication. Trypsin may</u>** be performed ~~after the test results are obtained~~**<u>selectively based on the routine practice of the local study site.</u>**<br>…<br>**<u>(4) Cardiac markers</u>**<br>**<u>Myocardial enzymes (creatine kinase (CK) and its isoenzymes (CK-MB)), troponin (TnI or TnT), and brain natriuretic peptide (BNP and/or NT-proBNP).</u>**<br>…<br>(7) **<u>Trypsin</u>**<br>**<u>Trypsin (trypsin, serum amylase, and lipase) may be performed selectively based on routine practice of the local study site.</u>**<br>**<u>(8) Pregnancy test</u>**<br>**<u>To be enrolled in this study, women of childbearing potential must have a negative result in the blood pregnancy test performed within 7 days prior to the first dose.(either blood or urine pregnancy test is acceptable; if urine pregnancy test is positive, then further bloods pregnancy test is needed).</u>**<br>5.1.13 Central laboratory assessments	

Section # and Name	Description of Change(s)<br>(*new text is in bold and underlined, deleted text is struck-through)*	Brief Rationale
	**HLX-PK,** ADA**, and biomarkers (PD-L1, TMB, and MSI) should be tested by the central laboratory.**<br><br>**The tumour tissue sections and blood** samples will ~~only~~**be used for testing the expression level of PD-L1, MSI, and TMB.**<br><br>**ADA samples will** be collected **only** prior to dosing, as detailed in the laboratory manual.<br><br>HLX10-PK and ADA samples will be collected at the following time points: 1 day before dosing of HLX10 at Cycles 1, 2, 4, 6, 8 and every 4 cycles afterwards, 0.5 hour after dosing of HLX10 at Cycles 1 and 8 (only for PK), **at** end-of-treatment visit and at safety follow-up.	
7.2 Adverse Event of Special Interest (AESI)	7.2 Adverse Event of Special Interest (AESI)<br><br>AESIs are events of scientific and medical interests in understanding the study drug and may require close monitoring and prompt contact with the sponsor by the investigator. AESIs can be serious or non-serious. Expedited reporting enables continuous monitoring of AESIs in order to describe and understand their association with t~~he use of study drug. AESIs for HLX10 include, but not limited to, events with potential inflammatory or immune-mediated mechanisms and that may require more frequent monitoring and/or intervention, such as steroids, immunosuppressants, and/or hormone replacement therapies~~**use of the study drug.**<br><br>**AESI in this study includes infusion-related reaction (IRR) and other immune-related adverse event (irAE).**<br><br>irAEs are defined as AEs that are associated with drug exposure and demonstrate immune-mediated mechanisms with no other unequivocal etiology. Serological, immunological, and histological (biopsy) data should	To define the AESI.

Section # and Name	Description of Change(s)<br>(*new text is in bold and underlined, deleted text is struck-through)*	Brief Rationale
	be used to support the diagnosis of irAEs ~~when~~**where** appropriate. Appropriate methods should be used to exclude tumorstumours, infections, metabolism, toxins, or other causes of irAEs. More specific guidelines for theirdescription about the assessment and treatment arefor irAEs is detailed in ~~Appendix 8.~~**"Investigator's Brochure" and Section "4.4 HLX10-Related Toxicity and Management".**<br><br>For suspected irAEs, the function of the relevant systems should be closely observed and adequate evaluation should be performed to confirm etiology and exclude other causes. Overall, HLX10 should be suspended or permanently discontinued and/or symptomatic treatment, such as glucocorticoids, should be given depending on the severity of events. If AEs are not ~~relieved~~**improved** or worsen after glucocorticoid therapy, **consideration may be given to** increasing **the** dose of glucocorticoid and/or using other systemic immunosuppressant therapies should be considered.. When the AE is ~~<~~**≤** Grade 1, the dose of glucocorticoid can be gradually reduced and the treatment should be continued for at least 1 month. HLX10 infusion therapy can be continued when the AE recovers to ~~<~~**≤** Grade 1 and the glucocorticoid dose is reduced to prednisone ~~<~~**≤** 10 mg/day (or ~~other~~ equivalent ~~drugs~~). When a ≥ Grade 3 irAE occurs again (except for endocrine system disorders), the subject should immediately and permanently discontinue the drug and withdraw from the study.<br><br>**AESI that meets the SAE criteria should be handled in accordance with the relevant procedures of SAE reporting. Despite not meeting the criteria of SAEs (i.e., the submission to the relevant regulatory and ethics department is not required), the following AEs are required to be reported to the sponsor within 24 hours (AESI):**<br><br>**● ≥ grade 3 of infusion reaction (infusion-related adverse reaction);**	

Section # and Name	Description of Change(s)<br>(*new text is in bold and underlined, deleted text is struck-through)*	Brief Rationale
	**● ≥ grade 2 of diarrhoea/colitis, uveitis, immune-related pneumonia, and immune-related myocarditis;**<br>**● ≥ grade 3 of other immune-related adverse events.**	
7.4.3 Events Occurring after the Subject's Withdrawal from the Study	7.4.3~~ Events Occurring after the Subject's Withdrawal from the Study~~<br>~~After the subject withdraws from the study, the investigator is no longer obligated to actively collect and report new AEs or SAEs after a 90-day safety follow-up period or after initiation of new anti-tumor therapy (whichever occurs first). However, if the investigator is aware of any SAE, including death, after a subject has withdrawn from the study, and the event is reasonably considered to be related to the study drug, the investigator should notify the sponsor's pharmacovigilance team or representative.~~	To delete the events occurring after the Subject's Withdrawal from the Study.
8.3 Interim Analysis and Final Analysis	8.3 Interim Analysis and Final Analysis<br>An Independent Data Monitoring Committee (IDMC) will be established in this study for interim analysis. In this study, ~~one~~**two** interim ~~analysis is~~**analyses are** scheduled, of which the overall type I error rate is controlled by O'Brien-Fleming α-spending function (approximated by Lan-DeMets method).<br>● The first ~~PFS~~ interim analysis will be~~ conducted~~**performed** when **approximately** 66% **subjects are enrolled. Its primary objective is to perform a revaluation** of the ~~PFS events (about 222 events)~~ ~~is~~ **sample size in a blind state with a significance level α of 0.000001 (two-tailed).**<br>● **The second interim analysis will be performed when 66% PFS cases are** observed, **so as** to assess the safety and efficacy ~~in~~ . **Its primary objective is** the ~~treatment group. α~~ **superiority assessment, with a significance level α**	To clarify the interim analysis and final analysis.

Section # and Name	Description of Change(s)<br>(*new text is in bold and underlined, deleted text is struck-through)*	Brief Rationale
	**of** 0.012 (two~~-sided) is used int~~**ailed) according to** the ~~first interim analysis based on the O'Brien~~**O'Brien**-Fleming **boundary** α -spending function.<br>● Final analysis of PFS will be conducted when the target number (about 336 PFS events) is observed, using α 0.046 (two-sided) based on the O'Brien-Fleming **boundary** α spending function.	
8.4.4 Safety analysis	8.4.4 Safety ~~Analysis~~**analysis**<br>AEs will be described ~~in accordance with the terms of the~~ **per** MedDRA **(version 22.0),** and graded ~~according to~~**per** CTCAE ~~v4.03~~**v5.0**. AEs during or after ~~the first~~ administration of study drug will be summarized per CTCAE grades. ~~Treatment-emergent adverse~~**Adverse** events ~~(TEAE)~~ and concomitant medications ~~will be respectively summarized by~~**during study** treatment. **will be separately summarized by treatment groups.** Clinical laboratory parameters, ECOG, vital signs, physical examinations, and ECG will be summarized by treatment **group** and ~~follow up~~ visit. Analysis will describe and present the observed values and changes from baseline by visit in the study.	To clarify the safety analysis.
10.2_Appendix 1	The version of Common Terminology Criteria for Adverse Events (CTCAE) is updated from v4.03 to v5.	To update the version of CTCAE.
Throughout the protocol	Some editorial changes were made.	To keep the consistency throughout protocol.

A Randomized, Double-Blind, Multicentre, Phase 3 Clinical Study to Compare HLX10 (Recombinant Humanized Anti-PD-1 Monoclonal Antibody Injection) versus Placebo in Combination with Chemotherapy (Cisplatin + 5-FU) as First-Line Therapy in Patients with Locally Advanced/Metastatic Oesophageal Squamous Cell Carcinoma (ESCC)

Protocol No.: HLX10-007-EC301

Statistical Analysis Plan

Version No.: 1.0

Date: 05/11/2022

Signature Page of Statistical Analysis Plan

A Randomized, Double-Blind, Multicentre, Phase 3 Clinical Study to Compare HLX10 (Recombinant Humanized Anti-PD-1 Monoclonal Antibody Injection) versus Placebo in Combination with Chemotherapy (Cisplatin + 5-FU) as First-Line Therapy in Patients with Locally Advanced/Metastatic Oesophageal Squamous Cell Carcinoma (ESCC)

Protocol No.: HLX10-007-EC301

Statistical Analysis Plan

Version No.: 1.0

[redacted]	Date
Statistician	Shanghai Henlius Biotech, Inc.
[redacted]	Date
Quality Control Statistician	Shanghai Henlius Biotech, Inc.
[redacted]	Date
Medical Manager	Shanghai Henlius Biotech, Inc.
[redacted]	Date
Clinical Pharmacology and Toxicology Manager	Shanghai Henlius Biotech, Inc.

Version Revision

Version No.	Revision Date	Revised by	Revised Content
1.0	05/11/2022	[redacted]	First version

Table of Contents

Table of Comparison for Abbreviations......7
1. Brief Introduction......11
2. Study Objectives......11
2.1 Primary Objective......11
2.2 Secondary Objective......12
3. Study Design......12
3.1 Overall Design......12
3.2 Study Endpoints......18
3.2.1 Efficacy Endpoints and Estimands......18
3.2.2 Safety Endpoints......23
3.2.3 Pharmacokinetic Endpoints......26
3.2.4 Immunogenicity Endpoints......27
3.2.5 Biomarker Analysis......27
3.2.6 Quality of Life Evaluation......27
3.3 Sample Size......28
3.4 Randomization and Blinding......29
3.4.1 Randomization......29
3.4.2 Blinding......29
3.5 Safety/Data Monitoring......30
4. Analysis Sets......30
5. Statistical Analysis Methods......34
5.1 General Principles......34
5.1.1 Definition of Time Point......36
5.1.2 Processing of Measurements at Unscheduled Visits......37
5.1.3 Processing of Missing Data......38
5.1.4 Covariates and Subgroups......44
5.1.5 Processing of Multiple Comparison/Multiplicity......45

5.1.6 Interim Analysis 47
5.2 Study Subjects 48
5.2.1 Subject Distribution 48
5.2.2 Major Protocol Violation....49
5.3 Demographic Information and Baseline Characteristics....49
5.3.1 Demographic Information 49
5.3.2 Medical History Related to Esophageal Squamous Cell Carcinoma....50
5.3.3 Prior Anti-tumor Therapy for Esophageal Squamous Cell Carcinoma 51
5.3.4 Medical History 54
5.3.5 Prior Medications....55
5.3.6 Prior Procedures....55
5.3.7 Smoking History and History of Alcohol Use, Allergy History and History of Drug Dependence 56
5.3.8 Biomarker Test during Screening Period 56
5.3.9 Other Tests during Screening Period 57
5.4 Compliance and Concomitant Medications 57
5.4.1 Study Medication and Compliance 58
5.4.2 New Anti-tumor Therapy for Esophageal Squamous Cell Carcinoma during Study Period 59
5.4.3 Concomitant Medications....63
5.4.4 Concomitant Procedures....64
5.5 Efficacy Analysis....64
5.5.1 Primary Efficacy Analysis....64
5.5.2 Secondary Efficacy Analysis....75
5.5.3 Biomarker Analysis....83
5.5.4 Subgroup Analysis 83
5.5.5 Other Analyses....84
5.6 Safety Analysis 85
5.6.1 Adverse Events 86

5.6.2 Laboratory Tests 97
5.6.3 12-lead ECG 98
5.6.4 Vital Signs 99
5.6.5 Physical Examination 99
5.6.6 ECOG Score 99
5.7 Pharmacokinetic Analysis 99
5.8 Immunogenicity Analysis 101
5.9 Biomarker Analysis 101
5.10 Quality of Life Score 101
5.10.1 EQ-5D-5L 102
5.10.2 EORTC QLQ-C30 103
5.10.3 EORTC QLQ-OES18 104
6. Changes to the Protocol/Statistical Analysis Plan 105
6.1 Changes Inconsistent with the Protocol 105
6.1.1 Definition of "Modified Intention-to-treat Set (mITT)" 105
6.1.2 Definition of Estimand 106
6.2 Changes to Statistical Analysis Plan 107
7. References 107

TABLE OF COMPARISON FOR ABBREVIATIONS

Acronyms/Abbreviations	Terminology
ADA	Anti-drug antibody
AE	Adverse event
AESI	Adverse event of special interest
ALT	Alanine aminotransferase
aPTT	Activated partial thromboplastin time
AST	Aspartate aminotransferase
ATC	Anatomical Therapeutic Chemical classification system
BMI	Body mass index
CMH	Cochran-Mantel-Haenszel test
COD	Data analysis cut-off date
CPS	Combined positive score
CR	Complete response
CT	Computed tomography
CTCAE	Common Terminology Criteria for Adverse Events
DCR	Disease control rate
DOR	Duration of response
ECOG	Eastern Cooperative Oncology Group
eCRF	Electronic Case Report Form
EORTC QLQ-C30	European Organization for Research and Treatment of Cancer Core Quality of Life Questionnaire
EORTC QLQ-OES18	European Organization for Research and Treatment

	of Cancer Oesophageal Cancer Module
EQ-5D-5L	EuroQoL Five Dimension Five Level Scale
ESCC	Esophageal squamous cell carcinoma
FFPE	Formalin-fixed paraffin-embedded
FT3	Free triiodothyronine
FT4	Free thyroxine
Hb	Hemoglobin
HBcAb	Hepatitis B core antibody
HBsAg	Hepatitis B surface antigen
HBV	Hepatitis B virus
HCV	Hepatitis C virus
HIV	Human immunodeficiency virus
HR	Hazard ratio
ICF	Informed Consent Form
ICH	The International Conference on Harmonization of Technical Requirements for Registration of Pharmaceuticals for Human Use
IDMC	Independent Data Monitoring Committee
INR	International normalized ratio
irAE	Immune-related adverse events
iRECIST	Immune Response Evaluation Criteria in Solid Tumors
IRRC	Independent Radiology Review Committee
ITT	Intention-to-treat set

IWRS/IVRS	Interactive Web/Voice Response System
kg	Kilogram
LS	Least squares
mean	Mean
MedDRA	Medical Dictionary for Regulatory Activities
mg	Milligram
min	Minimum
MMRM	Mixed-effects Model Repeated Measure
MRI	Magnetic Resonance Imaging
MSI	Microsatellite instability
NE	Non-evaluable
ORR	Objective response rate
OS	Overall survival
PD	Progressive disease
PD-1	Programmed death receptor 1
PD-L1	Programmed death ligand 1
PFS	Progression-free survival
PI	Principal investigator
PK	Pharmacokinetics
PKS	Pharmacokinetics set
PPS	Per-protocol set
PR	Partial response
PT	Preferred term

PT	Prothrombin time
RECIST	Response Evaluation Criteria in Solid Tumors
SAE	Serious adverse event
SAS	Statistical analysis software
SD	Stable disease
SOC	System Organ Class
SS	Safety set
T3	Triiodothyronine
T4	Thyroxine
TCO2	Total carbon dioxide
TEAE	Treatment-emergent adverse events
TMB	Tumor mutational burden
TSH	Thyroid stimulating hormone

1. BRIEF INTRODUCTION

Esophageal cancer is a malignant tumor that originates from esophageal epithelial cells and the main pathological types include esophageal squamous cell carcinoma (ESCC) and esophageal adenocarcinoma (EAC). The distribution of pathological types differ in different regions. Esophageal cancer in Western countries in Europe and North America is mainly EAC, which accounts for approximately 70%, while over 90% of esophageal cancer in China is ESCC. Esophageal cancer is the 6th leading cause of death from cancer in the world. However, in developing countries, it is the 4th leading cause of death from cancer. Recently, many overseas clinical studies have observed that immune checkpoint inhibitor therapy has achieved encouraging efficacy in the treatment of metastatic esophageal cancer. Completed and ongoing clinical trials on immunotherapy for esophageal cancer have affirmed the huge potential of immunotherapy in esophageal cancer. At the same time, there are many ongoing clinical trials using PD-1/PD-L1 inhibitor combined with chemotherapy for cancer. HLX10 is a novel monoclonal antibody independently developed by Shanghai Henlius Biotech, Inc. for the PD-1 target and it has sequentially obtained the approval of U.S. FDA, TFDA and China's NMPA to conduct dose-escalation phase 1 clinical trials. At present, the enrollment of patients for 4 dose arms have been completed for the dose-escalation study and the DLT assessment has been completed. More subjects are being included in the 4th dose arm, 10 mg/kg, as part of expanded enrollment.

This Statistical Analysis Plan (SAP) is written based on the study protocol (V4.0, version date: June 26, 2021), corresponding Case Report Forms (CRF) (V5.0, version date: December 08, 2021), relevant study documents and registration regulations to describe the data processing and statistical analysis methods, so as to evaluate efficacy and safety in the HLX10-007-EC301 project, and provide the table and figure templates for displaying data in the Clinical Study Report (CSR). Updates to the protocol or relevant study documents may require updates to this plan. This Statistical Analysis Plan shall be finalized before database lock.

2. STUDY OBJECTIVES

2.1 PRIMARY OBJECTIVE

To compare the clinical efficacy of HLX10 or placebo in combination with chemotherapy as

first-line therapy in patients with locally advanced/metastatic esophageal squamous cell carcinoma (ESCC).

2.2 SECONDARY OBJECTIVE

To compare the safety and tolerability of HLX10 versus placebo in combination with chemotherapy as first-line therapy in patients with locally advanced/metastatic esophageal squamous cell carcinoma (ESCC).

3. STUDY DESIGN

3.1 OVERALL DESIGN

This study is a randomized, double-blind, multicenter, phase 3 clinical study to compare the clinical efficacy, safety and tolerability of HLX10 versus placebo in combination with chemotherapy as first-line treatment regimen in subjects with locally advanced/recurrent or metastatic esophageal squamous cell carcinoma and to collect PK parameters and explore biomarkers related to efficacy.

The eligible subjects of this study shall be randomized to the following 2 arms at a ratio of 2:1 using the Interactive Web/Voice Response System (IWRS/IVRS):

- Arm A (HLX10 arm): HLX10 + chemotherapy (cisplatin + 5-FU)
- Arm B (control arm): placebo + chemotherapy (cisplatin + 5-FU)

The stratification factors for randomization include: PD-L1 expression level (CPS < 10 versus CPS ≥ 10), age (≥65 years old versus < 65 years old), and tumor status (locally advanced versus distant metastasis).

Subjects can only enter this study if they meet the inclusion criteria and do not meet any exclusion criteria at screening. Enrolled subjects shall be treated with the study drug until loss of clinical benefit, death, toxicity intolerance, withdrawal of informed consent, or other reasons specified by the protocol (whichever occurs first). Even if subjects do not experience the aforementioned situations after receiving 8 cycles of cisplatin and 12 cycles of 5-FU, they shall not continue using the said drugs. The maximum duration of dosing of HLX10/placebo is 2 years.

This trial consists of 3 periods: screening period (28 days), treatment period (until loss of clinical benefit, death, intolerable toxicity, withdrawal of informed consent or other reasons specified by the protocol, whichever occurs first), and follow-up period (including safety follow-up period and

survival follow-up period).

Refer to Table 1 for the study event flow chart.

Table 1 Study Event Flow Chart

**Study Period**	**Screening Period**		**Treatment Period (Each Cycle is 2 Weeks)**					**Discontinuation of Study Treatment**[1]	**Follow-up Period**[2]	
**Treatment Cycle/Visit Name**	Screening period		1	2	3	4	n	Discontinuation of study treatment	Safety follow-up	Survival follow-up[3]
**Visit Time**								After learning or confirming that the subject has discontinued the study treatment	30 days, 90 days (by telephone only) after the final study drug treatment[2]	Every 12 weeks
**Window Period (Day)**[4]	-28 to -8	-7 to -1		± 3	± 3	± 3	± 3	+ 3	± 7	± 7
**Study management procedures**										
Informed Consent Form	X									
Inclusion and exclusion criteria	X									
Demographics and medical history	X									
Prior treatments and concomitant treatments[5]	X			X	X	X	X	X	X	
**Clinical procedures/assessment**										
Adverse events[6]	X		X	X	X	X	X	X	X	
Quality of life[7]		X			X		X	X		
Echocardiography	X									
12-lead electrocardiogram (ECG)		X		X	X	X	X	X	X	
Complete physical examination	X									
Symptom-directed physical examination			X	X	X	X	X	X	X	
Body height, weight and vital signs[8]	X		X	X	X	X	X	X	X	
ECOG score		X		X	X	X	X	X	X	
Subsequent anti-tumor therapy									X	X
Survival status			X	X	X	X	X	X	X	X
**Study drug**										
Randomization[9]			X							
HLX10/placebo + chemotherapy[9]			X	X	X	X	X			
**Laboratory procedures/assessment: conducted at study site**										
Pregnancy test[10]		X			X		X	X	X	
Routine blood test, blood biochemistry test, coagulation test, routine urine test and cardiac function markers[11]		X		X	X	X	X	X	X	
Thyroid function test[12]		X			X		X	X	X	
Pancreatic enzymes[13]	(X)		(X)	(X)	(X)	(X)	(X)	(X)	(X)	
**Virology test**										
HBV surface antigen/core antibody, HBV DNA[14]	X									
If at baseline: HBV DNA (< 500 IU/mL or 2,500 copies/mL), and					X		X	X	X	

1. HBsAg (+), or 2. HBcAb (+), hepatitis B 5-item and HBV DNA tests shall be performed during the treatment period.									
HCV antibody, HCV RNA[14]	X								
If at baseline: HCV antibody (+) and HCV RNA (-), HCV antibody and HCV RNA shall be tested during the treatment period				X		X	X	X	
HIV antibody	X								
**Laboratory procedures/assessment: to be conducted at central laboratory**									
HLX10 - PK, ADA/NAb[15]		X	X	-	X	X	X	X	
PD-L1 expression of tumor tissues	X								
MSI and TMB expression of tumor tissues	X								
MSI and TMB tests of blood samples	X								
**Efficacy evaluation**									
Imaging test[16]	X	X					X	(X)	(X)
**Biomarker sample collection**									
Tumor tissue samples [17] (PD-L1, MSI, TMB)	X								
Blood samples [17] (MSI, TMB)	X								

1. If subjects discontinue the study treatment for any reason, the end-of-study-treatment visit shall be carried out as far as possible. If a subject completes the laboratory tests specified in the trial flow chart within 7 days (including 7 days) before the end-of-study-treatment visit, the tests do not have to be repeated. If the end-of-study-treatment visit occurs 30 days (± 7 days) after the final study drug treatment, safety follow-up does not have to be carried out. **Repeated screening** is allowed in this study and a new screening number shall be given during repeated screening.
2. Subjects who discontinue medication for reasons other than progressive disease continued to undergo imaging assessments at the scheduled time as far as possible **until progressive disease (including PD determined according to RECIST v1.1 and iRECIST (if any))**, initiation of a new anti-tumor therapy, withdrawal of informed consent, death or the end of the study (whichever occurs first). Subjects shall undergo safety follow-up at the site 30 days (± 7 days) after the final study drug treatment. If a new anti-tumor therapy has yet to be initiated, a safety follow-up shall be carried out again by telephone 90 ± 7 days after the last drug administration to only collect information on the subject's AE and concomitant medications related to the AE.
3. After subjects discontinue all study drug treatments, a telephone survival follow-up shall be carried out every 12 weeks ± 7 days; the frequency of survival follow-ups shall be increased as appropriate.
4. The window period of the screening period is 28 days, the window period of the treatment period and end-of-study-treatment visit is 3 days (the tumor assessment window period is 7 days), and the window period for the follow-up period was 7 days. Data of ECOG performance status score, pregnancy test, routine blood test, blood biochemistry test, coagulation test, routine urine test, thyroid function test and cardiac biomarker test performed during the screening period **within 7 days before the first drug administration** shall be recorded and shall meet the corresponding inclusion criteria and none of the exclusion criteria before the patient is enrolled.
5. Records of prior treatment shall be collected from 30 days before the screening visit to the signing of the informed consent form, and records on concomitant treatments shall be collected from the signing of the informed consent form to 30 days after the final study drug treatment. Concomitant treatments related to AEs shall be recorded until **90 days** after the final study drug treatment.
6. All AEs and SAEs shall be collected and recorded from the first administration of the study drug (C1D1) to the subject to 90 days after

the final study drug treatment or initiation of a new anti-tumor therapy (whichever occurs first); thereafter, only SAEs related to the study drug (HLX10/placebo) shall be recorded. Only serious adverse events caused by the protocol's mandatory interventions (for example, invasive procedures, biopsy, etc.) have to be reported after the signing of the ICF and before the first administration of the study drug (C1D1) (within 24 hours after learning about the event).

7. The quality of life scales include the EuroQoL Five Dimension Five Level Scale (EQ-5D-5L), the European Organization for Research and Treatment of Cancer Core Quality of Life Questionnaire (EORTC QLQ-C30) and European Organization for Research and Treatment of Cancer Oesophageal Cancer Module (EORTC QLQ-OES18). They shall be carried out during the screening period and once every 2 dosing cycles after the first drug administration, i.e., they shall be completed before drug administration in Cycles 3, 5, 7… until the discontinuation of the study treatment. If assessment is not performed within the 6 weeks before the end-of-treatment visit, the quality of life assessment shall be performed once at this visit.

8. **Body height is only measured during the screening period**; vital signs include body temperature, pulse, respiratory rate, and blood pressure. Weight shall be measured prior to each drug administration. If the change in the subject's weight from the **baseline** reference weight (weight before first drug administration) during the study is ≤ 10%, dose adjustment of study drug is not required. If the weight change was > 10%, the dose shall be re-calculated and the new weight shall be used as the baseline value for subsequent weight measurements.

9. The study drug shall be administered after all the clinical and laboratory procedures/assessments are completed, and every 2 weeks constitutes a dosing cycle. (The interval between the date of randomization and the day of first drug administration cannot exceed 3 days).

10. Women of childbearing age have to undergo a blood pregnancy test within 7 days prior to the first drug administration and will only be enrolled when the results are negative. The test (both blood and urine tests are accepted and if the urine pregnancy test was positive, blood pregnancy test is required for further examination) is performed within 3 days before **every 2 administrations** in the treatment period. Study drug administration shall be scheduled after the test results are obtained. Analyses will be conducted at the local study site.

11. Routine laboratory tests include routine blood test, blood biochemistry test, coagulation test, routine urine test and cardiac function marker test. Routine blood test items include red blood cell count, hemoglobin, platelet and white blood cell count, differential white blood cell count and percentages (neutrophil, lymphocyte, basophil, eosinophil, and monocyte). Blood biochemistry test items include blood urea nitrogen/urea, creatinine, sodium, potassium, magnesium, chlorine, bicarbonate/carbon dioxide combining power/total carbon dioxide (TCO_2), calcium, phosphorus, blood glucose, total bilirubin, direct bilirubin, alanine aminotransferase (ALT), aspartate aminotransferase (AST), alkaline phosphatase, lactate dehydrogenase, total cholesterol, total protein, and albumin. Routine urine test items include: specific gravity, pH, urine glucose, urine protein, urinary cast, urine ketone body and blood cells. Coagulation test include: international normalized ratio (INR), prothrombin time (PT), and activated partial thromboplastin time (aPTT). Cardiac function marker test include: myocardial zymogram [creatine kinase (CK) and its isozymes and troponin (TnI or TnT)] and brain natriuretic peptide (BNP and/or NT-proBNP). The tests shall be carried out **<u>within 3 days before drug administration of each cycle</u>**. When the aforementioned laboratory tests and study drug administration are scheduled on the same day, study drug administration shall be scheduled after the test results are obtained.

12. Thyroid function tests include triiodothyronine (T3 or FT3), thyroxine (T4 or FT4), and thyroid-stimulating hormone (TSH) analyses. Of which, the tests shall be carried out within 3 days before **every 2 drug administrations** in the treatment period, and study drug administration shall be scheduled after the test results are obtained. Analyses will be conducted at the local study site.

13. Pancreatic enzymes (trypsin, serum amylase, and lipase) tests are **optional** test items and shall be selectively tested according to the routine procedures of the local study site. (x): indicates that the test is optional.

14. All subjects have to undergo hepatitis B surface antigen (HBsAg)/core antibody (HBcAb), and hepatitis C virus (HCV) antibody tests during the screening period. HBsAg or HBcAb positive subjects shall be further tested for hepatitis B virus (HBV) DNA titer, and HCV antibody positive subjects shall be further tested for HCV RNA. If at screening (baseline): HBV DNA (< 500 IU/mL or 2,500 copies/mL),

and 1. HBsAg (+) or 2 HBcAb (+), hepatitis B 5-item (1). HBsAg (hepatitis B surface antigen) 2). HBsAb (hepatitis B surface antibody) 3). HBeAg (hepatitis B e antigen) 4). HBeAb (hepatitis B e antigen) 5). HbcAb (hepatitis B core antibody)) and HBV DNA tests shall be performed **every 2 cycles** during the treatment period. If at baseline: HCV antibody (+), and HCV RNA (-), HCV antibody and HCV RNA shall be tested **every 2 cycles** during the treatment period;

15. PK and ADA (NAb) sample collection: (Note: ADA/NAb samples shall only be collected before drug administration. Refer to the Laboratory Manual for details.)

- HLX10-PK and ADA (NAb) sample collection shall be carried out at the following time points: within 24 hours before HLX10 administration in Cycles 1, 2, 4, 6, and 8 and every 4 cycles thereafter, within 0.5 hours after the end of HLX10 administration in Cycles 1 and 8 of the treatment period, and at the end-of-treatment visit and safety follow-up.

16. CT or MRI [examination sites include the neck, chest, abdomen, pelvis and any other sites suspected to have tumor lesions; of which, **brain MRI or CT (MRI is preferred)** shall be carried out during the baseline period and by the investigator as clinically needed during the treatment period; **bone scans** shall be carried out during the baseline period and by the investigator as clinically needed during the treatment period] during the screening period, every 6 weeks (± 7 days) in the first 48 weeks before randomization and every 12 weeks (± 7 days) after 48 weeks. The examination method of the same site should be kept consistent as far as possible throughout the entire study. Contrast agent should be used unless there were contraindications. The investigator and the IRRC shall respectively assessed the tumor imaging results according to RECIST 1.1 (the frequency of tumor assessment may be increased by the investigator as clinically needed), and the investigator should determine subsequent treatment based on the efficacy evaluation results. Subjects who continue drug administration after the first PD (RECIST v1.1) shall undergo imaging assessment again every 4 – 8 weeks. If tumor evaluation is conducted within 28 days prior to the first drug administration and the same method and machine are used at the same hospital, it can serve as baseline tumor evaluation. During the end-of-treatment visit, if tumor imaging is performed within the past 4 weeks, tumor imaging does not have to be performed again for the end-of-treatment visit. **Subjects who discontinue medication for reasons other than progressive disease shall continue to undergo imaging assessments at the scheduled time until progressive disease**, initiation of a new anti-tumor therapy, withdrawal of informed consent, death, or the end of the study (whichever occurs first).

17. Subjects **shall** provide tumor samples (paraffin block or unstained section) that are formalin-fixed, paraffin-embedded (FFPE) and collected from sites that did not receive radiotherapy, as well as the relevant pathological reports of the above specimens. If a subject does not have archived tumor tissue samples, a fresh biopsy of tumor lesion performed during screening period will be acceptable to obtain the corresponding tumor samples (the quantity of the specimens obtained depends on the biopsy). Tumor tissue section and blood samples will be used for the testing of PD-L1 expression level, MSI and TMB (of which, the PD-L1 test is mandatory and the MSI and TMB tests are optional). Freshly collected specimens, resection, core needle biopsy, excision, incision, punch or forceps biopsies are all accepted. Samples collected by needle aspiration (i.e., samples without complete tissue structure that are only provided for cell suspension and/or cell smear), brushing samples, and precipitated cell samples that come from pleural or peritoneal effusion are not acceptable. **Refer to the Laboratory Manual for details on the tissue sample requirements.**

3.2 STUDY ENDPOINTS

3.2.1 Efficacy Endpoints and Estimands

3.2.1.1 Primary Efficacy Endpoints

This study uses PFS and OS as parallel dual primary endpoints.

3.2.1.1.1 Progression-Free Survival (PFS)

Progression-free survival is defined as the time from randomization to the time of the first recorded progressive disease or death due to any reason (whichever occurred first) as assessed by the IRRC according to RECIST v1.1.

The calculation formula of PFS was: PFS (month) = (min (date of first recorded progressive disease, death or censoring) - randomization date + 1) / 30.4375. If PFS needs to be censored as a new anti-tumor therapy is initiated for esophageal squamous cell carcinoma during the study drug treatment period and no progressive disease, death, etc. are observed before that, refer to "5.5.1.1 Progression-free Survival (PFS)" for the detailed censoring rules.

Population: Patients with locally advanced/metastatic esophageal squamous cell carcinoma (ESCC) who meet the inclusion criteria and do not meet the exclusion criteria

Treatments: HLX10/placebo in combination with chemotherapy (cisplatin + 5-FU), with 2 weeks (14 days) as 1 cycle

Variable: Progression-free survival (PFS) assessed by the IRRC according to RECIST v1.1

Intercurrent events and handling strategy:

#	Intercurrent Event	Handling Strategy and Description
1	• No progressive disease/death is observed before the initiation of a new anti-tumor therapy for esophageal squamous cell carcinoma<br>• Emergency unblinding or accidental unblinding due to other reasons	**Hypothetical strategy**: Assuming that the risk of progressive disease or death of a subject who experiences an intercurrent event is the same as that of a subject who does not experience an intercurrent event, PFS is censored on the date of the last tumor imaging examination before the intercurrent event<br>As the Independent Radiology Review Committee (IRRC) carries out evaluation independently, is not affected by

		other information and is not connected to the investigator's information, IRRC-assessed PFS is not affected by the investigator's emergency unblinding or accidental unblinding due to other reasons. Hence, investigator-assessed PFS shall only be censored on the date of the last tumor imaging examination before emergency unblinding or accidental unblinding due to other reasons
2	• Death before progressive disease	**Composite strategy**: Death is a direct reflection of the patient's loss of clinical benefits. Hence, death and progressive disease are both used as the target endpoints of PFS
3	• Temporary interruption of treatment<br>• Discontinuation of treatment due to adverse events or other non-progressive disease reasons<br>• Use of rescue medication	**Treatment policy strategy**: Temporary interruption of treatment, discontinuation of treatment due to adverse events or other reasons and use of rescue medication reflect clinical practice. Hence, a treatment policy strategy is used to continue collecting patients' imaging assessment data until progressive disease/death

Population-level summary: Hazard Ratio

3.2.1.1.2 Overall Survival (OS)

Overall survival is defined as the time from randomization to death from any cause.

The calculation formula of OS was: OS (month) = (min (date of death, censoring date) - randomization date + 1) / 30.4375. If OS is censored due to reasons such as loss to follow-up, refer to "5.5.1.2 Overall Survival (OS)" for the detailed censoring rules.

Population: Same as the population defined in "3.2.1.1.1 Progression-free Survival (PFS)"

Treatments: Same as the treatments defined in "3.2.1.1.1 Progression-free Survival (PFS)"

Variable: Overall survival (OS)

Intercurrent events and handling strategy:

#	Intercurrent Event	Handling Strategy and Description
1	• Initiation of a new anti-tumor therapy for esophageal squamous cell carcinoma<br>• Temporary interruption of treatment<br>• Discontinuation of treatment due to adverse events or other non-progressive disease reasons<br>• Use of rescue medication	**Treatment policy strategy**: Initiation of a new anti-tumor therapy for esophageal squamous cell carcinoma, temporary interruption of treatment, discontinuation of treatment due to adverse events or other reasons and use of rescue medication reflect clinical practice and do not affect the objectivity of overall survival (OS). Hence, a treatment policy strategy is used to continue collecting the patient's survival follow-up data until progressive disease/death

Population-level summary: Hazard Ratio

3.2.1.2 Secondary Efficacy Endpoints

The secondary efficacy endpoints of this study include the objective response rate (ORR) and duration of response (DOR).

3.2.1.2.1 Objective Response Rate (ORR)

Objective response rate (ORR) is defined as the proportion of subjects with IRRC-assessed best overall response (BOR) of complete response (CR) or partial response (PR) according to RECIST v1.1 and subjects who do not undergo post-baseline tumor assessment are deemed to have no response.

Population: Same as the population defined in "3.2.1.1.1 Progression-free Survival (PFS)"

Treatments: Same as the treatments defined in "3.2.1.1.1 Progression-free Survival (PFS)"

Variable: Proportion of IRRC-assessed BOR of CR or PR according to RECIST v1.1

Intercurrent events and handling strategy:

#	Intercurrent Event	Handling Strategy and Description
1	• CR or PR is not achieved before initiation of new anti-tumor therapy for esophageal squamous cell carcinoma	**While-on-treatment strategy**: A systemic new anti-tumor therapy will affect the biological effects or clinical efficacy of the study drug. Hence, the while-on-treatment strategy is used if CR or PR is not observed before the

	• CR or PR is not achieved before emergency unblinding or accidental unblinding due to other reasons	initiation of a new anti-tumor therapy for esophageal squamous cell carcinoma and only imaging data before the initiation of a new anti-tumor therapy for esophageal squamous cell carcinoma is included in the assessment<br>Emergency unblinding or accidentally unblinding due to other reasons will affect the clinical judgment of the investigator. As the Independent Radiology Review Committee (IRRC) carries out evaluation independently, is not affected by other information and is not connected to the investigator's information, IRRC-assessed ORR is not affected by the investigator's emergency unblinding or accidental unblinding due to other reasons. Hence, the while-on-treatment strategy is only used for investigator-assessed ORR and imaging data before emergency unblinding/accidental unblinding due to other reasons are involved in the investigator's assessment of ORR
2	• Temporary interruption of treatment<br>• Discontinuation of treatment due to adverse events or other non-progressive disease reasons<br>• Use of rescue medication	**Treatment policy strategy**: Temporary interruption of treatment, discontinuation of treatment due to adverse events or other reasons and use of rescue medication reflect clinical practice. Hence, a treatment policy strategy is used to continue collecting patients' imaging assessment data

Population-level summary: Odds Ratio

3.2.1.2.2 Duration of Response (DOR)

Duration of response (DOR) is defined as the IRRC-assessed time from the first recorded response (CR or PR) to the first recorded progressive disease or death according to RECIST v1.1.

The calculation formula of DOR was: DOR (month) = (min (date of first recorded progressive disease, death or censoring) - date of first recorded response (CR/PR) + 1) / 30.4375. If DOR has to

be censored as a new anti-tumor therapy is initiated for esophageal squamous cell carcinoma during the study drug treatment period and no progressive disease, death, etc. are observed before that, refer to "5.5.2.2 Duration of Response Event (DOR)" for the detailed censoring rules.

Population: Same as the population defined in "3.2.1.1.1 Progression-free Survival (PFS)" and subjects who achieved CR or PR

Treatments: Same as the treatments defined in "3.2.1.1.1 Progression-free Survival (PFS)"

Variable: IRRC-assessed duration of response (DOR) according to RECIST v1.1

Intercurrent events and handling strategy:

#	Intercurrent Event	Handling Strategy and Description
1	• CR or PR is not achieved before initiation of new anti-tumor therapy for esophageal squamous cell carcinoma<br>• CR or PR is not achieved before emergency unblinding or accidental unblinding due to other reasons	**While-on-treatment strategy**: A systemic new anti-tumor therapy will affect the biological effects or clinical efficacy of the study drug. Hence, the while-on-treatment strategy is used if CR or PR is not observed before the initiation of a new anti-tumor therapy for esophageal squamous cell carcinoma and the subject is deemed to have not achieved CR or PR during the study medication period.<br>Emergency unblinding or accidentally unblinding due to other reasons will affect the clinical judgment of the investigator. As the Independent Radiology Review Committee (IRRC) carries out evaluation independently, is not affected by other information and is not connected to the investigator's information, IRRC-assessed CR or PR is not affected by the emergency unblinding or accidental unblinding of the investigator due to other reasons. Hence, the subject is deemed to not have achieved CR or PR during the study medication period solely based on the investigator-assessed results
1	• No progressive disease/death is	**Hypothetical strategy**: Assuming that the risk of

	observed before the initiation of a new anti-tumor therapy for esophageal squamous cell carcinoma after achieving CR or PR<br>• Emergency unblinding or accidental unblinding due to other reasons after achieving CR or PR	progressive disease or death of a subject who experiences an intercurrent event is the same as that of a subject who does not experience an intercurrent event, DOR is censored on the date of the last tumor imaging examination before the intercurrent event<br>As the Independent Radiology Review Committee (IRRC) carries out evaluation independently, is not affected by other information and is not connected to the investigator's information, IRRC-assessed DOR is not affected by the investigator's emergency unblinding or accidental unblinding due to other reasons. Hence, investigator-assessed DOR is only censored on the date of the last tumor imaging examination before emergency unblinding or accidental unblinding due to other reasons
2	• Death before progressive disease	**Composite strategy**: Death is a direct reflection of the patient's loss of clinical benefits. Hence, death and progressive disease are both used as the target endpoints
3	• Temporary interruption of treatment<br>• Discontinuation of treatment due to adverse events or other non-progressive disease reasons<br>• Use of rescue medication	**Treatment policy strategy**: Temporary interruption of treatment, discontinuation of treatment due to adverse events or other reasons, sporadic treatments prohibited by the protocol, transfer of treatment arm or crossover treatment and use of rescue medication reflect clinical practice. Hence, a treatment policy strategy is used to continue collecting the patient's imaging assessment data until progressive disease/death

Population-level summary: Hazard Ratio

3.2.2 Safety Endpoints

The safety evaluation indicators of this study include: adverse events, laboratory tests, vital signs, physical examination, ECOG score, echocardiography and 12-lead ECG.

3.2.2.1 Adverse Events

Adverse event (AE) refers to any untoward medical occurrence of a patient or clinical study subject administered a pharmaceutical product . There is not necessarily a causal relationship between the adverse event and the treatment. Adverse events includes adverse and abnormal signs (includes abnormal tests and test findings), symptoms or diseases temporally related to the use of the (investigational) drug, regardless of whether they are related to the (investigational) drug (according to the ICH definition). All AEs and SAEs from the first administration of the study drug (C1D1) of the subject shall be collected and recorded until 90 days after the last drug administration or initiation of a new anti-tumor therapy (whichever occurs first) and only SAEs related to the study drug (HLX10/placebo) shall be recorded thereafter.

Adverse events of special interests (AESI) of this study include infusion reactions (infusion-related adverse reactions, IRR) and immune-related adverse events (irAE).

3.2.2.2 Laboratory Tests

Includes routine blood test, blood biochemistry test, coagulation test, routine urine test, cardiac function marker test, pregnancy test, thyroid function test, pancreatic enzymes and virology tests. Apart from the virology and pancreatic enzyme tests, other tests shall be performed within 7 days before the first drug administration. During the treatment period, the routine blood test, blood biochemistry test, coagulation test, cardiac function marker test and routine urine test shall be performed within 3 days before drug administration in each cycle and thyroid function test and pregnancy test shall be performed within 3 days before drug administration every 2 dosing cycles.

➢ Routine blood test (red blood cell count, hemoglobin, platelet count, white blood cell count, differential white blood cell count and percentages (neutrophil, lymphocyte, basophil, eosinophil, and monocyte).

➢ Blood biochemistry test (blood urea nitrogen/urea, creatinine, sodium, potassium, magnesium, chlorine, bicarbonate/carbon dioxide binding power/total carbon dioxide (TCO2), calcium, phosphorus, blood glucose, total bilirubin, direct bilirubin, alanine aminotransferase (ALT), aspartate aminotransferase (AST), alkaline phosphatase, lactate dehydrogenase, total cholesterol, total protein, and albumin)

➢ Coagulation test (international normalized ratio (INR), prothrombin (PT), and activated

partial thromboplastin time (aPTT))

- Cardiac function marker test (myocardial zymogram [creatine kinase (CK) and its isozymes (CK-MB) and troponin (TnI or TnT) and brain natriuretic peptide (BNP and/or NT-proBNP))
- Routine urine test (specific gravity, pH, urine glucose, urine protein, urinary cast, urine ketone body and blood cells)
- Thyroid function tests (triiodothyronine (T3 or FT3), thyroxine (T4 or FT4), and thyroid-stimulating hormone (TSH))
- Pancreatic enzymes (trypsin, serum amylase, and lipase, tests were selectively tested according to the routine procedures of the local study site)
- Pregnancy Test
- Virology test: If HBV DNA (<500 IU/mL or 2,500 copies/mL) during the screening period (baseline), and 1. HBsAg (+), or 2. HBcAb (+), hepatitis B 5-item (1). HBsAg 2). HBsAb 3). HBeAg 4). HBeAb 5). HbcAb) and HBV DNA tests shall be performed every 2 cycles during the treatment period. If HCV antibody (+), and HCV RNA (-) at baseline, HCV antibody and HCV RNA tests shall be performed every 2 cycles during the treatment period;

3.2.2.3 Vital Signs

Weight and vital signs shall be measured before every drug administration during the study treatment period. Vital signs examination indicators include:

- Body temperature (°C)
- Pulse (beats/minute)
- Respiratory rate (breaths/minute)
- Systolic blood pressure/diastolic blood pressure (mmHg)
- Weight (kg)

3.2.2.4 Physical Examination

Complete physical examination shall be carried out during the screening period, including: head and neck (including thyroid), chest (including heart, lungs), abdomen (liver, gallbladder, spleen, kidneys), extremities, skin, lymph nodes, nervous system and general conditions of subjects.

During the study treatment period, the investigator shall carry out symptom-directed physical examination based on clinical observations and symptoms. Clinically significant abnormal physical

examination results that are determined by the investigator to have significantly worsened from the screening period or that are new shall be recorded as adverse events.

3.2.2.5 ECOG Score

ECOG score assessment shall be performed within 7 days before the first study drug administration during the screening period, once every 2 weeks in the study drug treatment period, at the end-of-study-treatment visit and during the safety follow-up period.

3.2.2.6 Echocardiography

Echocardiography shall be performed during the screening period and the left ventricular ejection fraction shall be recorded (LVEF, %).

3.2.2.7 12-lead ECG

12-lead ECG shall be performed within 7 days before the first study drug administration during the screening period, once every 2 weeks in the study drug treatment period, at the end-of-study-treatment visit and during the safety follow-up. The assessment indicators include:

- Heart rate (HR, beats/min)
- PR interval (msec)
- QRS duration (msec)
- QT interval (msec)
- QTcF interval (msec)

3.2.3 Pharmacokinetic Endpoints

3.2.3.1 Serum Drug Concentration

HLX10 drug concentration in serum at all scheduled sampling time points shall be summarized. Samples shall be collected within 24 hours before HLX10 administration in Cycles 1, 2, 4, 6, and 8 and every 4 cycles thereafter, within 0.5 hours after the end of HLX10 administration in Cycles 1 and 8 of the treatment period, and at the end-of-treatment visit and during safety follow-up.

3.2.3.2 Pharmacokinetic Parameters

Refer to Table 2 below for the pharmacokinetic (PK) parameters of this study.

Table 2 Pharmacokinetic Parameters and Calculation Method

PK Parameter	Name	Unit	Calculation Method
C1-C_{max}	Maximum concentration after 1st drug administration	µg/mL	Take the actual measured value
C2-C_{trough}	Trough concentration before the 2nd drug administration	µg/mL	Take the actual measured value
C8-C_{max}	Maximum concentration after 8th drug administration	µg/mL	Take the actual measured value
C8-C_{trough}	Trough concentration before the 8th drug administration	µg/mL	Take the actual measured value
R_{ac}_C_{max}	Accumulation ratio of maximum concentration	None	C8-C_{max}/ C1-C_{max}
R_{ac}_C_{trough}	Accumulation ratio of trough concentration	None	C8-C_{trough}/ C2-C_{trough}

3.2.4 Immunogenicity Endpoints

Blood samples shall be collected for immunogenicity test within 24 hours before HLX10 administration in Cycles 1, 2, 4, 6, and 8 and every 4 cycles thereafter, at the end-of-treatment visit and during safety follow-up.

Immunogenicity assessment indicators include:

- Anti-drug antibody (ADA) positive rate
- Neutralizing antibody (NAb, to be tested only when ADA is positive) positive rate

3.2.5 Biomarker Analysis

- The relationship between PD-L1 expression of tumor tissues and efficacy shall be explored
- The relationship between microsatellite instability (MSI), tumor mutational burden (TMB) and efficacy shall be explored

3.2.6 Quality of Life Evaluation

The quality of life evaluation includes:

- EuroQol Five Dimension Five Level Scale (EQ-5D-5L)
- European Organization for Research and Treatment of Cancer Core Quality of Life Questionnaire (EORTC QLQ-C30)
- European Organization for Research and Treatment of Cancer Oesophageal Cancer Module (EORTC QLQ-OES18)

The quality of life assessment shall be performed during the screening period, once every 2 weeks during the study drug treatment period and at the end-of-study-treatment visit. If assessment is performed within 6 weeks before the end-of-treatment visit, quality of life assessment shall be performed once at this visit.

3.3 SAMPLE SIZE

This study uses PFS and OS as parallel dual primary endpoints, of which an interim analysis of OS shall be performed when the target number of PFS events have occurred and the final analysis shall be performed when the target number of OS events have occurred. PFS will only be analyzed once during final analysis. In order to control the overall type I error, α assignment is as follows:

PFS: α = 0.005 (one-sided)

OS: α = 0.02 (one-sided)

The randomization ratio of the treatment arm and control arm in the study is 2:1. The sample size is based on the assumption that the median progression-free survival (PFS) of the control arm, i.e. placebo + chemotherapy (cisplatin + 5-FU) is 5 months, the median progression-free survival (PFS) of the HLX10 + chemotherapy arm is 7.35 months, i.e. the hazard ratio (HR) of the HLX10 + chemotherapy arm to the control arm is approximately 0.68. Hence, type I error is set as α = 0.005 (one-sided) and the enrollment time is 24 months. PFS final analysis will be performed approximately 4 months after the last subject is enrolled and 339 PFS events need to be observed to obtain 80% power. Assuming that the drop-out rate is 10%, a total of 495 patients (330 patients in the HLX10 arm and 165 patients in the control arm) need to be enrolled into the 2 arms.

Assuming that the median overall survival (OS) of the control arm is 10 months, the median overall survival (OS) of the HLX10 + chemotherapy arm is 13.70 months, i.e. the hazard ratio (HR) is approximately HLX10 + chemotherapy to the control arm is 0.73, using Group Sequential Design,

interim efficacy analysis shall be performed once when the target number of PFS event has been reached. The O' Brien-Fleming α spending function of Lan-Demets algorithm shall be used to control overall type I error α = 0.02 (one-sided). Assuming that the enrollment time is 24 months, OS final analysis shall be performed approximately 12 months after the last subject is enrolled and 388 OS events need to be observed to obtain 80% power. Moreover, a total of 540 patients need to be enrolled into the 2 arms (360 patients in the HLX10 arm and 180 patients in the control arm).

Comprehensively considering the sample size required for PFS and OS evaluation, a total of 540 patients need to be enrolled for this study (360 patients in HLX10 arm and 180 patients in control arm).

3.4 RANDOMIZATION AND BLINDING

3.4.1 Randomization

This study adopts a randomized, double-blind study design. The eligible subjects will be randomized at a ratio of 2:1 to the following 2 arms using the Interactive Web/Voice Response System (IWRS/IVRS):

- Arm A (HLX10 arm): HLX10 + chemotherapy (cisplatin + 5-FU)
- Arm B (control arm): placebo + chemotherapy (cisplatin + 5-FU)

The stratification factors for randomization include: PD-L1 expression level (CPS < 10 versus CPS ≥ 10), age (≥65 years old versus < 65 years old), and tumor status (locally advanced versus distant metastasis).

The randomization code table shall be generated and stored by a third-party non-blinded statistician who is responsible for randomization, and kept strictly confidential from the blinded study team before database lock and study unblinding.

3.4.2 Blinding

During the study treatment, the subject, investigator, sponsor and designated personnel shall not know about the random treatment assignment, except after emergency unblinding. The non-blinded statistician who is responsible for randomization shall securely archive the randomization plan and random code table to ensure that the entire blinded study team have no way of finding out the treatment arm assignment throughout the entire study. During the study drug treatment, if the

investigator determines that the subject experiences a life-threatening situation related to the use of the study drug and the investigator believes that knowing the drug used by the subject is helpful for the treatment of the adverse event, emergency unblinding may be performed. The investigator should ensure that unblinding is performed according to the provisions of the protocol.

This study will perform overall unblinding after the last subject is randomized and completes the end-of-study visit. Unblinding will be performed only in case of emergency (emergency rescue can only be conducted if the type of randomized drug received by the subject is known) or as required by regulatory authorities. Otherwise, blinding should be maintained. All randomization codes will be unblinded only after all data are entered into the database, all data queries are resolved and subjects are included in analysis sets.

3.5 SAFETY/DATA MONITORING

An independent data monitoring committee (IDMC) shall be set up in this study to carry out the interim analysis and shall be responsible for the safety and efficacy data monitoring of this study, study implementation quality assessment, provision of trial design adjustment recommendations and performing other emergency analyses and recommendations, etc. in a blinded state that the IDMC decides to add. Unless there are special circumstances, IDMC will convene a meeting approximately once every 6 months to monitor the safety of subjects, assess the subject implementation quality and the integrity and scientificity of the entire study, etc.

Refer to the “Independent Data Monitoring Committee Charter” for details of the IDMC members, responsibilities and meeting format, etc. of this project.

4. ANALYSIS SETS

This study shall include the following analysis sets:

Intention-to-treat set (ITT)

Defined as all randomized subjects who entered the trial. The ITT population will serve as the primary analysis population for the efficacy analysis of this study. Analyses targeted at the ITT population will be conducted based on the randomized treatment arms.

Modified Intention-to-treat Set (mITT)

Defined as all randomized subjects who entered the trial and did not experience systemic drug

dispensing errors. The mITT population shall be used as the sensitivity analysis population for efficacy analysis in this study and the mITT population analysis shall be analyzed based on the randomized treatment arms. Subjects who experienced systemic drug dispensing errors are defined as the 51 subjects who were affected by erroneous IWRS settings and assigned to the placebo arm but received HLX10 therapy.

In April 2020, a subject in this study experienced an SAE and the pharmacovigilance department needed to perform unblinding and decide whether to report the SAE to the regulatory department. On April 26, 2020, the IWRS administrator discovered after receiving the unblinding reminder email that subjects who were randomized to the placebo arm were receiving HLX10 combination chemotherapy. Subsequently, it was discovered in an internal review that subjects who were 33.3 kg $<$ weight $\leq$ 66.6 kg in the control arm were erroneously set to be in the HLX10 arm in the IWRS system drug settings. As of April 26, 2020, this weight arm enrolled a total of 51 subjects, resulting in some of the subjects in the control arm being erroneously treated with HLX10 in combination with chemotherapy.

On the day the issue was discovered, the system setting error was corrected. On April 30, 2020, ethics submission of 21 affected study sites and informed notification to the affected subjects were initiated. On October 27, 2020, the event was reported to the National Medical Products Administration (NMPA). On November 09, 2020, an IDMC meeting was convened to review the safety data of this study and this event. IDMC recommends to continue the study according to the current plan. The sponsor was requested to actively take measures and further communicate with the ethics committee and CDE, so as to seek guiding opinions. Hence, on November 12, 2020 and December 10, 2020, the leading site's ethics committee convened a 3rd and 4th meeting for this event, respectively, and granted approval to continue the study. It also requested for informed notification and unblinding to be performed on 7 of the affected subjects. As of April 23, 2021, 6 out of the 7 subjects that the leading site's ethics committee requested to unblind were unblinded. One subject died on November 07, 2020 and the sub-site's ethics committee filed the approval to not perform unblinding. In addition, 2 subjects should be unblinded as per the request of the sub-site's ethics committee.

Upon discussion, clinical medical experts believe that the systemic drug dispensing error cannot represent the actual clinical practice and cannot reflect the true therapeutic effects of the HLX10 arm

or the control arm in actual clinical practice. Therefore, it is recommended to exclude the efficacy data of subjects who experienced systemic drug dispensing errors from the ITT set and use it as the modified intention-to-treat set (mITT), using ITT as the primary efficacy analysis population and mITT as the sensitivity analysis population.

Per-Protocol Set (PPS)

The per-protocol set is a sub-set of the ITT and all randomized and enrolled subjects who did not experience major protocol deviations that significantly affect the primary efficacy assessment constitute the per-protocol set. The efficacy analysis based on PPS shall be used as a supportive analysis of the ITT analysis. The specific definition of the per-protocol set population shall be confirmed before database lock.

Safety Set (SS)

Defined as all subjects who received the investigational drug at least once. The SS is the primary analysis population for safety indicators, and will be analyzed based on the treatment that subjects actually received.

Pharmacokinetics Set (PKS)

Defined as all subjects who received at least one administration of HLX10, have at least one detected concentration after drug administration at the scheduled PK time point, and have no major protocol violations that may significantly impact PK assessment. PKS shall be used for pharmacokinetic analysis.

All statistical analysis sets will be discussed during the data review meeting and confirmed before database lock. Refer to Table 3 for the relationship between analysis variables and the analysis sets.

Table 3 Corresponding Relationship between Analysis Variables and Analysis Population

Endpoint Variables and Estimands	Analysis Sets
**Subject distribution and baseline characteristics**	
Subject distribution	All subjects
Analysis set distribution	Randomized and enrolled subjects
Major protocol violation	ITT, mITT

Demographic information	ITT, mITT
Medical history related to esophageal squamous cell carcinoma	ITT, mITT
Prior surgical therapy/radiotherapy/systemic therapy/other therapy for esophageal squamous cell carcinoma	ITT, mITT
Past medical history	ITT, mITT
Prior/concomitant medications	ITT, mITT
Prior/concomitant procedures	ITT, mITT
Smoking history and history of alcohol use, allergy history and history of drug dependence	ITT, mITT
Biomarker test during screening period	ITT, mITT
Virology test during screening period	ITT, mITT
Echocardiography during screening period	ITT, mITT
New surgical therapy/radiotherapy/systemic therapy/other therapy for esophageal squamous cell carcinoma	ITT, mITT
**Efficacy Analysis**	
Progression-free survival (PFS)	ITT, mITT, PPS
Overall survival (OS)	ITT, mITT, PPS
Objective response rate (ORR）	ITT, mITT, PPS
Duration of response (DOR)	ITT, mITT, PPS
**Safety Analysis**	
Study medication and compliance — HLX10/placebo, cisplatin, fluorouracil	SS
Adverse event	SS
Laboratory tests	SS
12-lead ECG	SS
Vital signs	SS
ECOG score	SS
Physical examination	SS
**Pharmacokinetic Analysis**	

Serum drug concentration of HLX10	PKS
Pharmacokinetic parameters of HLX10	PKS
**Immunogenicity data**	
Anti-drug antibody (ADA/NAb)	SS
**Quality of life evaluation**	
EQ-5D-5L, EORTC QLQ-C30, EORTC QLQ-OES18	ITT
**Biomarker Analysis**	
Relationship between PD-L1 expression of tumor tissues and efficacy	ITT, mITT, PPS
Relationship between microsatellite instability (MSI), tumor mutational burden (TMB) and efficacy	ITT, mITT, PPS

5. STATISTICAL ANALYSIS METHODS

5.1 GENERAL PRINCIPLES

All statistical analyses and reports in this study shall be completed in accordance with the guiding principles of ICH E3 and E9. Unless otherwise specified, all statistical analyses and reports shall be completed using statistical analysis software package (SAS Institute Inc., USA) 9.2 or later versions. Statistical analysis reports shall be created using Microsoft Word 2016 or higher document/RTF/PDF formats and based on SAS® (9.2 or higher versions) and the analysis process shall be fully programmed.

Unless otherwise specified, descriptive statistics for quantitative indicators include number of subjects, arithmetic mean, standard deviation, median, maximum and minimum. Descriptive statistics of serum drug concentration and PK parameters also include coefficient of variation (CV%), geometric mean and geometric coefficient of variation. Descriptive statistics of qualitative indicators (including classification indicators or grading indicators) include number of subjects, percentage and/or number of events. If the number of subjects is 0, the percentage will not be presented. Unless otherwise specified, the denominator for percentage calculation is the total number of subjects in the corresponding arm of the corresponding analysis population.

Unless otherwise specified, the maximum and minimum will be kept to the same number of

decimal places as raw data, mean, geometric mean and median will be kept to 1 more decimal place than raw data, standard deviation will be kept to 2 more decimal places than raw data, coefficient of variation and geometric coefficient of variation will be kept to 2 more decimal places than percentage, but all statistics shall not have more than 3 decimal places after rounding. The percentage of qualitative indicators will be kept to 1 decimal place after rounding.

Unless otherwise specified, a two-sided test will be used for all statistical tests in the study. Generally, if p-value is greater than or equal to 0.0001 in the statistical test, it is kept to 4 decimal places. If p-value is smaller than 0.0001, it is expressed as "P <0.0001". If p-value is greater than 0.9999, it is expressed as "P > 0.9999". The confidence interval will be kept at 1 more decimal place than the point estimate.

If there are any tests or assessments that have yet to be performed, the data lists will only list the visit results of completed relevant tests and assessments. Unscheduled assessments (laboratory data or vital signs that are additionally collected at unscheduled clinical visits or those collected during assessment or treatment of AEs) will only be listed, but not statistically summarized. If the same laboratory indicator has multiple test results at a certain visit, other than the baseline result during the screening period, only the first valid test result shall be included in the statistical summary and other test results shall only be included in the data lists. Invalid and unscheduled laboratory test results shall not be included in the statistical summary and only be included in the data lists.

Changes in categorical data from baseline will be statistically summarized using a crosstab. If there are any tests or assessments that have yet to be performed, the data lists will only list the visit results of completed relevant tests and assessments.

As the expected number of randomized subjects of each study site is relatively small and there are many participating study sites, the study data of all study sites will be combined for summary analysis, so as to estimate the general therapeutic effects of the study drug. The "site" factor will not be considered in the statistical model or subgroup analysis.

Unless otherwise specified, the data lists will be arranged in order by treatment arm, subject number, visit, assessment date/time (if applicable) and parameters.

All data as of the data analysis cut-off date (COD) or end of study will be summarized. The follow-up time is defined as min (the latest date of performing various assessments on the subject or

the subject-related event in the database, the date of death/loss to follow-up/withdrawal from the study or COD) - date of randomization + 1.

The format of tables, figures and listings will be specified in a separate TFL Mockup Shell document.

5.1.1 Definition of Time Point

Unless otherwise specified, the baseline of this study is defined as the last measurement that can be accurately obtained before randomization or the first study drug administration (i.e., before drug administration on the day of the first drug administration) and change from baseline is defined as the difference between the measurement obtained at a scheduled examination time point and the baseline value. If a subject does not use the drug after randomization, the date of randomization shall be used as the reference date defined by the baseline.

The reference date is defined as the date of randomization or the first study drug administration and the day of study of an assessment or event relative to the reference date is defined as:

- if the date of assessment or event is on or after the reference date,
  day of study = date of assessment/event - reference date + 1
- if the date of assessment or event is before the reference date,
  day of study = reference date - date of assessment/event

Time variables that use "day" as the unit can be converted to time variables with "month" as the unit according to (12 * number of days / 365.25 or number of days / 30.4375) or converted to time variables with "week" as the unit according to (number of days / 7).

"End-of-treatment (EoT)" date is defined as the date when the subject completes all specified study drug treatments according to the protocol requirements, or date of progressive disease (iCPD if continued administration of the study drug treatment is approved after the first progression), death, permanent discontinuation of subject's study medication as decided by the investigator or date that the subject is no longer willing to receive study drug treatment, whichever occurs first.

"End-of-treatment visit" is defined as the visit carried out within 30 days after the subject is confirmed to have ended treatment (and should be completed before the subject initiates a new anti-tumor therapy).

"End-of-study (EoS)" date is defined as the date of subject's death, early withdrawal from the

study or the date that the sponsor decides to terminate the entire study.

5.1.2 Processing of Measurements at Unscheduled Visits

Measurements obtained at unscheduled visits only need to be considered during statistical analysis of the following relevant indicators:

- baseline measurement

Baseline is defined as the last measurement that can be accurately obtained before randomization or the first study drug administration (i.e., before drug administration on the day of the first drug administration) and test data of test results at scheduled or unscheduled visits recorded during the screening period that are the closest to randomization or the first study drug administration shall be used as the baseline measurement.

- measurements during treatment period

Table 4 Processing of Measurements during Treatment Period

Type of Data	Scheduled Visit			Unscheduled Visit			Remarks
	Tables	Figures	Listings	Tables	Figures	Listings	
Imaging examination	√	√	√	√	√	√	Test results that require IRRC/investigator approval and from scheduled and unscheduled tests will be included in efficacy analysis
Safety examination	√	√	√	×	×	√	
Serum drug concentration test	√	√	√	×	×	√	
Immunogenicity test	√	√	√	×	×	√	
Quality of life evaluation	√	√	√	×	×	√	

5.1.3 Processing of Missing Data

Unless otherwise specified, missing baseline, efficacy, safety, pharmacokinetics, immunogenicity, and quality of life assessment results or test results in this study will not be imputed. For time-event efficacy data, refer to “5.5 Efficacy Analysis” for target event censoring rules. The main purpose of data processing is to process missing data when summarizing data into tables instead of imputing raw data. Source data should still be presented in listings.

5.1.3.1 Missing Date of Esophageal Squamous Cell Carcinoma Diagnosis

For missing dates of first esophageal squamous cell carcinoma diagnosis, first metastasis diagnosis, and latest recurrence,

（1） If only the day is missing, impute the date based on the first day of the known month (i.e., “xx 01, xxxx”).

（2） The date shall not be imputed otherwise.

The missing date cannot be imputed to be later than the date of ICF signing and only subjects with non-missing date after imputation will be included in the statistical summary.

5.1.3.2 Missing Date of Prior/Concomitant Medication/Procedure

If the start date or end date of medication/procedure is partially missing, the missing date shall be imputed to determine whether it is prior or concomitant medication/procedure.

- **Missing Start Date of Medication/Procedure**

（1） If the year and month are known, impute the first day of the known month (i.e., “xx 01, xxxx”).

（2） If only the year is known, impute the first day of January of the known year (i.e., “January 01, xxxx”).

（3） If the year, month and day are all missing, the date shall not be imputed and only the end date shall be used to determine whether the medication/procedure is used before or after the first drug administration.

- **Missing End Date of Medication/Procedure**

If the end date of medication/procedure is completely or partially missing, the end date shall be

estimated in a way that maximizes the duration of the medicine/procedure.

（1） If the year and month are known, impute the last day of the known month (i.e., “xx 28/29/30/31, xxxx”).

（2） If only the year is known, impute the last day of the same year (i.e., “December 31, xxxx”).

（3） If the year, month and day are all missing and the answer to whether ongoing is not “Yes”, use the start date as the end date. If the answer to whether ongoing is “Yes”, impute the analysis data cut-off date (COD) as the end date.

5.1.3.3 Missing Date of Anti-Tumor Therapy for Esophageal Squamous Cell Carcinoma

If the start date or end date of anti-tumor therapy for esophageal squamous cell carcinoma is partially missing, the missing date shall be imputed to determine the duration of prior anti-tumor therapy and the time of the end of treatment relative to the subject’s informed consent. If the date of surgical therapy for esophageal squamous cell carcinoma is missing, it shall be imputed according to the rules of “Missing End Date of Prior Anti-tumor Therapy”.

- **Missing Start Date of Prior Anti-tumor Therapy**

（1） If the year and month are known, impute the first day of the known month (i.e., “xx 01, xxxx”).

（2） If only the year is known, impute the first day of January of the known year (i.e., “January 01, xxxx”).

（3） If the year, month and day are all missing, the date shall not be imputed.

- **Missing End Date of Prior Anti-tumor Therapy**

（1） If the year and month are known, impute the last day of the known month (i.e., “xx 28/29/30/31, xxxx”).

（2） The date shall not be imputed otherwise.

（3） The imputed end date of prior anti-tumor therapy shall not be later than the date of informed consent signing.

5.1.3.4 Missing Date of New Anti-tumor therapy for Esophageal Squamous Cell Carcinoma

- **Missing Start Date**

If the start date/ date of a new anti-tumor therapy for esophageal squamous cell carcinoma is

partially missing, the missing date shall be imputed to determine whether the therapy affects the evaluation of primary efficacy indicators. The missing start date/end date of "new radiotherapy for esophageal squamous cell carcinoma", "new surgical therapy for esophageal squamous cell carcinoma" and "new systemic therapy for esophageal squamous cell carcinoma" shall only be imputed for the assessment of efficacy indicators. The imputed start date/end date shall not be earlier than the date of the subject's first study drug administration (C1D1). If the imputed start date/end date is earlier than the date of the first study drug administration, the missing date shall be imputed based on the date of the first study drug administration (C1D1).

（1） If the year and month are known, impute the first day of the known month (i.e., "xx 01, xxxx").

（2） If only the year is known, impute the first day of January of the known year (i.e., "January 01, xxxx").

（3） If the year, month and day are all missing, the date shall not be imputed and the record of this new anti-tumor therapy for esophageal squamous cell carcinoma shall not be included in efficacy indicator assessment.

- **Missing End Date**

（1） If only the year and month are known, impute the last day of the known month (i.e., "xx 28/29/30/31, xxxx) or the analysis data cut-off-date (COD), whichever is earlier.

（2） If only the year is known or the year, month and day are all missing and the answer to whether ongoing is not "Yes" (if applicable), use the start date as the end date. If the answer to whether ongoing is "Yes" (if applicable), impute the analysis data cut-off date (COD) as the end date.

5.1.3.5 iRECIST Assessment Results Systematically not Filled In

If the tumor imaging assessment result of a subject according to RECIST v1.1 is available but the assessment result according to iRECIST is not filled in systematically , the efficacy assessment result according to iRECIST of the corresponding visit cycle shall be imputed as the efficacy assessment result according to RECIST v1.1. The letter "i" representing assessment according to iRECIST shall also be added to the front of the corresponding result. If the assessment according to RECIST v1.1 of a certain cycle is "SD", the iRECIST assessment result of the corresponding visit cycle shall be imputed as "iSD". The first PD result assessed according to iRECIST shall be imputed

as ”iUPD”. These imputation rules are only applicable for assessment results according to iRECIST that correspond to imaging visits before the first progressive disease (PD), as assessed according to RECIST v1.1. The assessment results according to iRECIST that correspond to imaging visits after the first PD shall not be imputed.

5.1.3.6 Missing Information on Adverse Events (AE)

5.1.3.5.1 Missing Date of Adverse Event

If the date of the adverse event is missing, and comparison with the reference date (such as the date of first study drug administration or data analysis cut-off date (COD)) is required (such as to determine whether it is a treatment-emergent adverse event (TEAE)), the missing date shall be imputed according to the following method before determination, unless otherwise specified. For the imputation of missing dates of adverse event, the imputed start date shall not be earlier than the date of the subject’s first study drug administration (C1D1). If the imputed start date is earlier than the date of the first study drug administration, the missing date shall be imputed based on the date of the first study drug administration(C1D1). The end date shall not be later than the date of death. If the imputed end date is later than the date of death, the missing date shall be imputed based on the date of death.

- **Missing Start Date of Adverse Event**

If the start date of the AE is completely or partially missing, the start date is assumed to occur at the earliest time of treatment possible, unless other data can prove that the AE did not occur during the treatment period (such as the AE end date is earlier than the date of the first drug administration).

（1） If the year and month are known, and the year and month are not the same year and month as the first study drug administration, impute the first day of the known month (i.e., “xx 01, xxxx”).

（2） If the year and month are known, and the year and month are the same year and month as the first study drug administration, the start date of the AE shall be the same as the date of the first study drug administration (date refers to “xx xx, xxxx”).

（3） If only the year is known and the year is not the same as the year of the first study drug administration, impute “January 01, xxxx”.

（4） If only the year is known, and the year is the same as the year of the first study drug

administration, the start date of the AE shall be the same as the date of first study drug administration (date refers to “xx xx, xxxx”.

（5） If the year, month and day are all missing, the date of first study drug administration shall be used as the corresponding start date of the adverse event.

（6） The date is considered to be missing otherwise.

（7） If the imputed start date falls after the end date, the end date shall be used as the corresponding start date.

- **Missing End Date of Adverse Event**

When calculating the duration of an AE, if the AE is not resolved by the analysis cut-off date or the end of the study, the end status of AE will be censored and imputed with the date of the last follow-up visit or the date of death (whichever is earlier). Unresolved includes AEs with outcome status of “Not Recovered/Not Resolved”, “Recovering/Resolving”, “Aggravating/Worsening”, “Unknown” or unclear end time. If the end date of the AE is completely or partially missing, the end date shall be imputed in a way that maximizes the duration of the AE.

（1） If the year and month are known, impute the last day of the known month (i.e., “xx 28/29/30/31, xxxx”).

（2） If only the year is known, impute the last day of the same year (i.e., “December 31, xxxx”).

（3） If the end date imputed according to the above 2 rules is later than the analysis data cut-off date (COD), impute the analysis data cut-off date as the end date of the AE.

5.1.3.5.2 Missing Correlation of Adverse Event

When the correlation between an AE and the study drug is missing, the AE will be summarized as a treatment-related adverse event.

5.1.3.7 Processing of Laboratory Test Values

Missing laboratory test results will not be imputed. If the test value is recorded as below (and equal to) or above (and equal to) the test range value (e.g.,$<$ x, $\leq$ x, $>$ x, $\geq$ x), it will be processed based on the test range value (i.e., = x) at the time of statistical description, but will still be listed based on the result recorded in the CRF at the time of data listing, i.e., “$<$ x”, “$\leq$ x”, “$>$ x”, or “$\geq$ x”.

5.1.3.8 Missing Information on Death

（1） Data of subjects with specific death information but missing date of death shall be processed as that of death events.

（2） If the year, month and date are completely missing, impute the last accurately known date of survival + 1 as the date of death.

（3） If the month and day are missing, impute the (min (date of last accurately known date of survival + 1, analysis data cut-off date) or January 01 in the year of death), whichever is later, as the date of death.

（4） If the day is missing, impute the (min (date of last accurately known date of survival + 1, analysis data cut-off date), or the first day of the same month the date the death was learned), whichever is later, as the date of death.

The last accurately known date of survival is defined as the latest complete date (excluding the survival follow-up date with survival status of “unknown/lost to follow-up” or “death”, the follow-up date 90 days after the last drug administration, the end date of treatment with HLX10/placebo/cisplatin/fluorouracil due to “loss to follow-up”, and the date of early withdrawal from the study due to the main reason of “subject lost to follow-up”) that can be obtained from all events/finding tests or assessments related to the subject in all collected data.

5.1.3.9 Missing End Date of Treatment

（1） If the subject has specified the end of treatment and the “end date of treatment” is missing, the date of the event selected in the “main reason for end of treatment” shall be imputed. If the date of the event cannot be confirmed, the date of the last study drug administration + 1 shall be imputed.

（2） If the subject is clearly “still receiving treatment” or has not specified the end of treatment as of the analysis data cut-off date (COD), the analysis data cut-off date (COD) shall be imputed when calculating results related to the duration of study treatment.

5.1.3.10 Missing End Date of Study

（3） If the subject has clearly “completed the trial” or “withdraws early” and the “study completion/withdrawal date” is missing, the latest date that can be obtained from all relevant events/finding tests or assessments of the subject shall be imputed.

（4） If the subject is clearly “still receiving treatment” or “still undergoing follow-up” as of the analysis data cut-off date (COD), the analysis data cut-off date (COD) shall be imputed when calculating results related to the duration of the study.

5.1.3.11 Serum Drug Concentration Data Processing

Refer to Table 5 below for the principles for processing of serum drug concentrations and pharmacokinetic parameters.

Table 5 Principles for Processing of Serum Drug Concentrations and Pharmacokinetic Parameters

Type	Processing Rules		
	Tables	Figures	Listings
Serum blood concentration is below the limit of quantification (BLQ)	Process values below BLQ at the time of serum drug concentration summary as 0 (present as “NC” when geometric mean and geometric coefficient of variation cannot be calculated)	Process values below BLQ at the time of serum drug concentration plotting as 0	Present the original results, e.g. “BLQ”
Missing serum drug concentration	Not included in statistical summary	Not included in plotting	Present original results
Pharmacokinetic (PK) parameters	Not included in statistical summary when calculation is not possible	not applicable	Present the calculation results and mark as “NC” when calculation is not possible

5.1.3.12 Processing of Abnormal Values and Outliers

Any abnormal outliers will be checked if discovered. If necessary, they will be corrected or confirmed as abnormal outliers after confirmation with the investigator.

5.1.4 Covariates and Subgroups

Important baseline characteristics in this study include:

- ECOG score
- sex

- MSI/MMR (MSS/MSI-L or MSI-H)
- TMB (< 10 muts/Mb or ≥ 10 muts/Mb)

If the important baseline characteristics are not balanced between the arms, the factors of heterogeneity will be included as covariates in the stratified analysis, and the actual measured values will be used when the baseline characteristics are included in the analysis.

Subgroup analyses, including subgroups defined by stratification factors and subgroups defined by biomarkers, will be performed in this study:

- age < 65 years old vs. ≥65 years old
- ECOG score: 0 vs. 1
- sex: male vs. female
- PD-L1 expression level: CPS < 10 vs. CPS ≥ 10
- tumor status: locally advanced vs. distant metastasis
- MSI/MMR: microsatellite stable or microsatellite instability-low (MSS/MSI-L) or microsatellite instability-high (MSI-H)
- TMB: < 10muts/Mb vs. ≥ 10muts/Mb

The true stratification/subgroup factor values collected shall be used for analysis.

5.1.5 Processing of Multiple Comparison/Multiplicity

This study uses PFS and OS as parallel dual primary endpoints, of which an interim analysis of OS shall be performed when the target number of PFS events have occurred and the final analysis shall be performed when the target number of OS events have occurred. PFS will only be analyzed once during final analysis. In order to control the overall type I error, α assignment is as follows:

PFS: α = 0.005 (one-sided)

OS: α = 0.02 (one-sided)

As of 04/15/2022 (interim analysis data cut-off date), a total of 267 OS events were observed, and the maturity of OS event information content was approximately 68.81%. The significance level of the interim analysis test for the OS indicator shall be adjusted using the SAS PROC SEQDESIGN module such that the significance level of the log-rank test for the OS indicator in this interim analysis is 0.010 (two-sided), and the significance level of the log-rank test for the OS indicator in the final analysis is 0.037 (two-sided).

The α reassignment strategy of the Group Sequential Holm Procedure shall be used. If the null hypothesis is rejected in the interim analysis for a primary efficacy endpoint (i.e., the *p*-value actually obtained at the time point of the analysis is less than the corresponding nominal test level), the initial α assigned to the primary efficacy endpoint will be fully recovered and assigned to another primary efficacy indicator that did not reject the original hypothesis. The O'Brien-Fleming α spending function (using Lan-DeMets method for approximation) method shall be used to recalculate and update the test level of the indicator at each analysis time point. For example, if the *p*-value of the log-rank test is less than 0.01 at the time of the final analysis of PFS, and the *p*-value of the log-rank test of the OS indicator is greater than or equal to 0.010, the initial α (one-sided 0.005) assigned to the PFS indicator will be fully recovered and assigned to the OS indicator. In this case, the total α of the OS indicator will be updated to 0.025 (one-sided). The test level of the interim analysis/final analysis of the OS indicator will be recalculated using the O'Brien-Fleming α spending function (using Lan-DeMets method for approximation) to obtain a test level of 0.014 (two-sided) for the OS indicator in the interim analysis. The test level of the final analysis is 0.046 (two-sided) and the *p*-value obtained from actual calculation of the OS indicator shall be compared with the updated test level.

Refer to Table 6 below for the test level of each indicator at each analysis time point.

Table 6 Test Level at Each Analysis Time Point

Scenario	Progression-free survival (PFS)				Overall survival (OS)			
Analysis Time Point	Before Update		After Update		Before Update		After Update	
	Z value	*p*-value[1]	Z value	*p*-value[1]	Z value	*p*-value[1]	Z value	*p*-value[1]
Neither PFS in final analysis nor OS in interim analysis is superior								
Interim analysis[2]	2.580	0.010	/	/	2.580	0.010	/	/
Final analysis	—	—	—	—	2.086	0.037	/	/
PFS is superior in final analysis, but OS in interim analysis is not superior (α assigned to PFS is transferred to OS and the test margin is updated)								
Interim analysis[2]	2.580	0.010	/	/	2.580	0.010	2.470	0.014
Final analysis	—	—	—	—	2.086	0.037	1.997	0.046
PFS in the final analysis is not superior, but OS in the interim analysis is superior (α assigned to OS is transferred to PFS and the test margin is updated)								
Interim analysis[2]	2.580	0.010	2.326	0.020	2.580	0.010	/	/
Final analysis	—	—	—	—	/	/	/	/
Both PFS in final analysis and OS in interim analysis are superior								

Interim analysis[2]	2.580	0.010	/	/	2.580	0.010	/	/
Final analysis	—	—	—	—	/	/	/	/

Note:

[1] The p-value in the table indicates the nominal test level (two-sided).

[2] The final analysis of PFS shall be performed when the number of target events (339 PFS events) have occurred, the interim analysis of OS shall be performed at the same time as the final analysis of PFS, and the final analysis of OS shall be performed when the number of target events (388 OS events) have occurred. Therefore, the interim analysis in the table represents the final analysis of PFS and the interim analysis of OS, and the final analysis represents the final analysis of OS.

5.1.6 Interim Analysis

In this study, an interim analysis of OS shall be performed when the target number of PFS events have occurred and the final analysis shall be performed when the target number of OS events have occurred. PFS will only be analyzed once during final analysis. O'Brien-Fleming α-spending function (using the Lan-DeMets algorithm) will be used to control overall type I error rate (one-sided 0.025):

$$\alpha(t_k) = 2\left(1 - \Phi\left(\frac{\Phi^{-1}(1-\alpha/2)}{\sqrt{t_k}}\right)\right)$$

of which, t_k is the information content accumulated during the kth interim analysis, Φ is a standard normal distribution function, and Φ^{-1} is a quantile inverse function of the standard normal distribution function.

- The interim analysis of OS shall be performed at the time of the final analysis of PFS. The primary objectives are safety assessment and superiority test of PFS and OS indicators. The significance level of the log-rank test for the PFS indicator in this analysis is 0.01 (two-sided). As of 04/15/2022 (interim analysis data cut-off date), a total of 267 OS events were observed, and the maturity of OS event information content was approximately 68.81%. According to the O'Brien-Fleming α spending function, the significance level of the log-rank test for the OS indicator in this interim analysis is 0.010 (two-sided) (since the SAS module reports two-sided p-value, the significance level here is presented as two-sided).
- The final analysis of OS shall be performed when the target number of OS events (about 388 events) is observed, and the significance level α of the final analysis log-rank test for the OS

indicator is 0.037 (two-sided);

- The significance level will be adjusted for each analysis based on the actual number of OS events at the analysis time point. If an indicator rejects the null hypothesis at the analysis time point, α of the indicator will be recovered and reassigned, and the test level of each analysis time point of another indicator will be updated.

The interim analysis of this study will be performed by independent statisticians and the results will be provided to the IDMC separately. The IDMC will assess the results of the interim analysis and provide recommendations accordingly. Reports and recommendations for interim analysis work will be presented in a separate document. If the IDMC provides recommendations for superiority in the final analysis of PFS and/or interim analysis of OS upon review, the sponsor will decide whether to lock the database and submit the data results to the regulatory authorities.

5.2 STUDY SUBJECTS

5.2.1 Subject Distribution

Descriptive statistics will be used to summarize subject distribution in screening and screen failure, randomization and enrollment, study drug treatment, study completion and early withdrawal, and subjects included in each analysis set (and reasons for exclusion from each analysis set), and subject distribution charts will be produced (generated using SAS/Word tool).

- The number and percentage of subjects who have undergone screening, are screen failures and the main reasons for screen failure will be summarized.
- The number and percentage of subjects randomized and enrolled and treated/not treated with the study drug will be summarized by treatment arm and total.
- The number and percentage of subjects who are still receiving treatment and ended treatment at the time of the analysis data cut-off date (COD) and the main reasons for ending treatment will be summarized by treatment arm and total.
- The number and percentage of subjects who are still receiving treatment, still undergoing follow-up, completed the study and withdrew early, and the main reasons for early withdrawal at the time of analysis data cut-off (COD) will be summarized by treatment arm and total. Subjects who are still undergoing follow-up is defined as subjects who have ended treatment but have not

completed/withdrawn from the study, including safety follow-up and survival follow-up. Completion of the study includes subjects who died or who are still receiving treatment/undergoing follow-up at the time of the sponsor’s decision to terminate the entire study;

- The number and percentage of subjects who are and are not included each analysis set, as well as the main reasons for not being included in each analysis set will be summarized by treatment arm.
- The main reasons for subjects’ screen and enrollment failure will be listed.
- The study drug treatment status of subjects and the main reasons for discontinuation of treatment will be listed.
- The main reasons for the end of the study of subjects will be listed.
- The subjects’ analysis sets will be listed.

5.2.2 Major Protocol Violation

Major protocol violations in different treatment arms will be summarized using descriptive statistics, including:

- number and percentage of subjects who experienced at least one major protocol violation;
- number and percentage of subjects in each major protocol violation category.

All subjects with protocol deviations and protocol violations and details of protocol deviations/violations will be listed. All major protocol violations and their impact on statistical analysis shall be discussed and confirmed by the investigator, sponsor or designated clinical study experts, medical experts, statisticians, etc. at the data review meeting.

5.3 DEMOGRAPHIC INFORMATION AND BASELINE CHARACTERISTICS

Demographic information and baseline characteristics analyses will be based on ITT and mITT, and subject demographics and baseline characteristics will be statistically summarized by treatment arm and total.

5.3.1 Demographic Information

Continuous indicators will be statistically described using the non-missing number of subjects, arithmetic mean, standard deviation, median, minimum and maximum, mainly including:

- age (years old)

- body height (cm)
- Weight (kg)
- body mass index (BMI, kg/m^2, calculated as: weight / (body height / 100) ^2)

Categorical indicators will be statistically described using the number and percentage of subjects according to the categories on the CRF, mainly including:

- stratification by age (≥ 65 years old or < 65 years old)
- sex (male or female)
- ethnicity (Han Chinese or others)
- baseline ECOG score (0 point or 1 point)
- PD-L1 expression level (1 ≤ CPS < 10, CPS ≥ 10)
- tumor status (locally advanced or distant metastasis)

Details of subject demographics and baseline characteristics will be listed.

5.3.2 Medical History Related to Esophageal Squamous Cell Carcinoma

Continuous indicators will be statistically described using the non-missing number of subjects, arithmetic mean, standard deviation, median, minimum and maximum, mainly including:

- time from first diagnosis to informed consent (months, calculated as: (date of informed consent signing - date of first esophageal squamous cell carcinoma diagnosis) / 30.4375, if the date of first esophageal squamous cell carcinoma diagnosis is missing, process according to the rules of "5.1.3.1 Missing Date of Esophageal Squamous Cell Carcinoma Diagnosis");
- time from first metastasis diagnosis to informed consent (months, calculated as: (date of informed consent signing - date of first metastasis diagnosis) / 30.4375, if the date of first metastasis diagnosis is missing, process according to the rules of "5.1.3.1 Missing Date of Esophageal Squamous Cell Carcinoma Diagnosis");
- time from most recent recurrence to informed consent (months, calculated as: (date of informed consent signing - date of most recent recurrence) / 30.4375, if the date of the most recent recurrence is missing, process according to the rules of "5.1.3.1 Missing Date of Esophageal Squamous Cell Carcinoma Diagnosis");

Categorical indicators will be statistically described using the number and percentage of subjects according to the categories on the eCRF, mainly including:

- histopathological type at first diagnosis
- degree of differentiation
- site of primary tumor at first diagnosis (multiple sites may be selected, including single primary site and multiple primary sites)
- first TNM stage
  - primary tumor (T)
  - regional lymph node (N)
  - distant metastasis (M)
- first clinical stage of tumor
- TNM stage of esophageal squamous cell carcinoma at time of informed consent signing
  - primary tumor (T)
  - regional lymph node (N)
  - distant metastasis (M)
- clinical stage at time of informed consent signing
- tumor status at the time of informed consent signing
- site if first metastasis diagnosis is distant metastasis (multiple sites may be selected, including single primary site and multiple primary sites)
- whether it recurred

The first diagnosis date, first diagnosis result, histology type at first diagnosis, degree of differentiation, site of primary tumor at first diagnosis, first TNM stage (primary tumor (T), regional lymph node (N), distant metastasis (M)), first clinical stage of tumor, TNM stage at the time of informed consent signing (primary tumor (T), regional lymph node (N), distant metastasis (M)), clinical stage at the time of informed consent signing, tumor status at the time of informed consent signing, date of first metastasis diagnosis, site if metastasis is distant metastasis, whether it recurred and the date of last recurrence of the subjects in each arm will be listed.

5.3.3 Prior Anti-tumor Therapy for Esophageal Squamous Cell Carcinoma

5.3.3.1 Prior Surgical Therapy for Esophageal Squamous Cell Carcinoma

Continuous indicators will be statistically described using the non-missing number of subjects,

number of cases (if necessary), arithmetic mean, standard deviation, median, minimum and maximum, mainly including:

- time from last prior surgery to informed consent (months, calculated as: (date of informed consent signing - date of last prior surgery) / 30.4375))

Categorical indicators will be statistically described using the number and percentage of subjects according to the categories on the CRF, mainly including:

- whether there is any prior surgical therapy for esophageal squamous cell carcinoma
- purpose of surgery

History of prior surgical therapies for esophageal squamous cell carcinoma will be coded using the ICH Medical Dictionary for Regulatory Activities (MedDRA) 23.0 or later versions, the number and percentage of subjects will be summarized by treatment arm and total according to System Organ Class (SOC) and Preferred Term (PT), and sorted by SOC > PT in descending order based on the total number of subjects.

Whether a subject has prior surgical therapy for esophageal squamous cell carcinoma, name of surgery, purpose of surgery, date of surgery, System Organ Class (SOC) and Preferred Term (PT) will be listed.

5.3.3.2 Prior Radiotherapy for Esophageal Squamous Cell Carcinoma

Continuous indicators will be statistically described using the non-missing number of subjects, number of cases (if necessary), arithmetic mean, standard deviation, median, minimum and maximum, mainly including:

- duration of radiotherapy (days, calculated as: end date of last radiotherapy - start date of first radiotherapy + 1) if the subject has received multiple prior radiotherapies, the duration of radiotherapy is the sum of the duration of prior radiotherapies
- radiotherapy dose (Gray), if the subject has received multiple prior radiotherapies, the radiotherapy dose is the sum of the doses of the prior radiotherapies
- time from the end of the last radiotherapy to the date of informed consent (months, calculated as: (date of informed consent - date of end of last radiotherapy) / 30.4375)

Categorical indicators will be statistically described using the number and percentage of subjects according to the categories on the CRF, mainly including:

- whether there is prior radiotherapy for esophageal squamous cell carcinoma
- type of radiotherapy
- radiotherapy site
- purpose of treatment
- whether there was progressive disease at the site of prior radiotherapy

Whether there is prior radiotherapy for esophageal squamous cell carcinoma, type of radiotherapy, radiotherapy site, purpose of radiotherapy, radiotherapy dose, start date, end date, and whether there was progressive disease at the site of prior radiotherapy will be listed by treatment arm.

5.3.3.3 Prior Systemic Therapy for Esophageal Squamous Cell Carcinoma

Continuous indicators will be statistically described using the non-missing number of subjects, number of cases (if necessary), arithmetic mean, standard deviation, median, minimum and maximum, mainly including:

- duration of treatment (days, calculated as: end date of last systemic therapy - start date of first systemic treatment + 1), if the subject has received multiple prior systemic therapies, the duration of treatment is the sum of the duration of prior systemic therapies
- end of last treatment to date of informed consent (months, calculated as: (date of informed consent - end date of last systemic therapy) / 30.4375)

Categorical indicators will be statistically described using the number and percentage of subjects according to the categories on the CRF, mainly including:

- whether there is any prior systemic therapy related to esophageal squamous cell carcinoma
- purpose of treatment
- type of treatment

The names of prior systemic therapies for esophageal squamous cell carcinoma will be coded using the Anatomical Therapeutic Chemical classification system (ATC) in the World Health Organization Drug Dictionary (WHO) B3 2020 Mar or later versions. The number and percentage of subjects with prior anti-tumor systemic therapy will be summarized according to the highest codable ATC classification level and Preferred Name (PN) by treatment arm and total. For example, ATC Level 4 (chemical classification) is preferred. If Level 4 is not available, ATC Level 3 (pharmacological classification) will be used for coding and summary, so on and so forth. They will

be sorted by ATC > PN in descending order based on the total number of subjects.

Whether there is prior systemic therapy for esophageal squamous cell carcinoma, including purpose of treatment, type of treatment, therapeutic drug, and start and end dates, will be listed by treatment arm.

5.3.3.4 Other Prior Therapy for Esophageal Squamous Cell Carcinoma

Continuous indicators will be statistically described using the non-missing number of subjects, number of cases (if necessary), arithmetic mean, standard deviation, median, minimum and maximum, mainly including:

- time from the last treatment to the date of informed consent (months, calculated as: (date of informed consent - date of last other therapy) / 30.4375)

Categorical indicators will be statistically described using the number and percentage of subjects according to the categories on the CRF, mainly including:

- whether there are any other prior therapies related to esophageal squamous cell carcinoma
- purpose of treatment

Whether there is a history of other prior therapy for esophageal squamous cell carcinoma, including the name of treatment, purpose of treatment and date of treatment, will be listed by treatment arm.

5.3.4 Medical History

Categorical indicators will be statistically described using the number and percentage of subjects according to the categories on the CRF, mainly including:

- past medical history other than esophageal squamous cell carcinoma
- CTCAE grade
- whether ongoing
- whether there are other relevant surgeries

Medical history other than esophageal squamous cell carcinoma (including history of surgical therapy) will be coded using the ICH Medical Dictionary for Regulatory Activities (MedDRA) 23.0 or later versions, and the number and percentage of subjects will be summarized according to System Organ Class (SOC) and Preferred Term (PT).

Past medical history of subjects other than esophageal squamous cell carcinoma (including history of surgical therapy), including name of disease, CTCAE grade, start date, whether ongoing, end date, System Organ Class (SOC), Preferred Term (PT), whether there are relevant surgeries and name of surgery, will be listed by treatment arm.

5.3.5 Prior Medications

Prior medications are defined as drugs (including traditional Chinese medicine preparations) which end date of medication is between 30 days before the start of the screening period and the day before the signing of the informed consent form. If a certain medication cannot be classified as “prior medication” or “concomitant medication” after the missing medication date is imputed according to the rules of “5.1.3.3 Missing Date of Prior/Concomitant Medication/Procedure”, it will be deemed as “concomitant medication”.

Prior medications will be coded using World Health Organization Drug Dictionary (WHODD) B3 2020 Mar or later versions. The number and percentage of subjects with at least 1 prior medication during the study will be summarized by treatment arm and total, the number and percentage of subjects with prior medication will be summarized by therapeutic classification (ATC2) and Preferred Name (PN), and sorted by ATC 2 > PN in descending order based on the total number of subjects.

Details of the subject’s prior medication, including the name of the drug, indications, single dose and unit, frequency of administration, route of administration, start date, whether ongoing and end date, etc., will be listed by treatment arm and marked as “prior medication”.

5.3.6 Prior Procedures

Prior procedure is defined as procedure which end date is between 30 days before the start of the screening period and the day before the signing of the informed consent form. If a certain procedure cannot be classified as “prior procedure” or “concomitant procedure” after the missing treatment date is imputed according to the rules of “5.1.3.3 Missing Date of Prior/Concomitant Medications/procedures”, it will be deemed as “concomitant procedure”.

Prior procedures will be coded using the ICH Medical Dictionary for Regulatory Activities (MedDRA) 23.0 or later versions. The number and percentage of subjects with at least 1 prior procedure during the study will be described by treatment arm and total, and the number and

percentage of subjects that used procedure will be summarized by System Organ Class (SOC) and Preferred Term (PT), and sorted by SOC > PT in descending order based on the total number of subjects.

Details of the subject's prior procedures, including the name of the treatment, indications, start date, whether ongoing and end date, etc. will be listed by treatment arm, and marked as "prior procedure".

5.3.7 Smoking History and History of Alcohol Use, Allergy History and History of Drug Dependence

Continuous indicators will be statistically described using the non-missing number of subjects, arithmetic mean, standard deviation, median, minimum and maximum, mainly including:

- smoking history and history of alcohol use
  - ➢ smoking duration (years)
  - ➢ number of cigarettes smoked (cigarettes/day)
  - ➢ duration of alcohol use (years)

Categorical indicators will be statistically described using the number and percentage of subjects according to the categories on the eCRF, mainly including:

- smoking history and history of alcohol use
  - ➢ whether there is a smoking history
  - ➢ whether there is a history of alcohol use
- allergy history
  - ➢ whether there is a history of drug allergies
  - ➢ whether there is a history of other allergies
- history of drug dependence
  - ➢ whether there is a history of addictive drug dependence

The subjects' smoking history and history of alcohol use, allergy history and history of drug dependence will be listed by treatment arm.

5.3.8 Biomarker Test during Screening Period

The test results of tumor tissue biomarkers (PD-L1, TMB and MSI tests) and blood sample

biomarkers (TMB and MSI tests) of subjects during the screening period will be summarized.

Continuous indicators will be statistically described using the non-missing number of subjects, arithmetic mean, standard deviation, median, minimum and maximum, mainly including:

- PD-L1 test
  - combined positive score (CPS)
  - tumor proportion score (TPS, %)
  - tumor-associated immune cells positive rate (IC, %)
  - mononuclear immune cell density score (MIDS)
  - MIDS Bin
- TMB test (mutations/Mb)

Categorical indicators will be statistically described using the number and percentage of subjects according to the categories on the eCRF, mainly including:

- PD-L1 test (1 ≤ CPS < 10, CPS ≥ 10)
- MSI test results (microsatellite instability-high (MSI-H), microsatellite instability-low (MSI-L), and microsatellite stable (MSS))
- TMB test (< 10 mutations/Mb, ≥ 10 mutations/Mb)

The sample type, sampling date and test results of tumor tissues and blood sample biomarkers of subjects during the screening period will be listed by treatment arm.

5.3.9 Other Tests during Screening Period

Virology test results during the screening period will be statistically described using the number and percentage of subjects, including HBsAg, HBcAb, HIV and HCV antibodies, according to the categories on the CRF.

Baseline echocardiography results will be summarized and described, including the continuous indicator left ventricular ejection fraction (LVEF, %) and categorical indicator test results.

The virology test results and baseline echocardiography results of the subjects during the screening period will be listed by treatment arm.

5.4 COMPLIANCE AND CONCOMITANT MEDICATIONS

Study medication and compliance analyses will be performed based on SS. During the study,

new anti-tumor therapies for esophageal squamous cell carcinoma, concomitant medications and concomitant procedures may affect the efficacy evaluation, so their analyses will be based on ITT and mITT. The study medication and compliance, new anti-tumor therapies for esophageal squamous cell carcinoma, concomitant medications and concomitant procedures will be statistically summarized by treatment arm and total.

5.4.1 Study Medication and Compliance

Compliance is defined as: the total actual dose / total planned dose * 100%, the total actual dose is the sum of the actual study dose in all treatment cycles, and the total planned dose is the sum of the theoretical study dose in all treatment cycles. The medication status and compliance during the study of HLX10/placebo, cisplatin and fluorouracil will be summarized respectively.

Continuous indicators will be statistically described using the non-missing number of subjects, number of cases (if necessary), arithmetic mean, standard deviation, median, minimum and maximum, mainly including:

- total planned/actual dose of the study (mg)
- overall trial compliance (%), calculated as: total actual dose / total planned dose * 100%
- number of days of treatment (days, calculated as: date of last drug administration - date of first drug administration + 1)
- total number of treatment cycles (continuation of medication after temporary drug interruption is counted as the same cycle)
- mean number of days between injections (days, calculated as: (date of last drug administration - date of first drug administration + 14) / total number of treatment cycles, only summarized among subjects with number of treatment cycles greater than 1)
- mean infusion time (minutes, calculated as: sum (end time of injection - start time of injection + 1) of dosing cycle / total number of treatment cycles)
- planned (or actual) dose intensity (mg/week, calculated as: planned (or actual) dose intensity = cumulative total planned (or actual) dose / total planned (or actual) number of dosing cycles, total number of weeks = (date of last drug administration - date of first drug administration + 7) / 7)
- relative dose intensity (%, calculated as: planned dose intensity / actual dose intensity *

100%)

Categorical indicators will be statistically described using the number and percentage of subjects according to the categories on the CRF, mainly including:

- overall trial compliance category (< 80%, 80% – 120%, > 120%)
- total number of treatment days category (exposure ≥ 6 months, exposure ≥ 12 months)
- measures taken for the investigational drug, including
  - ➢ whether there is a dose adjustment
    - ◆ dose unchanged
    - ◆ interruption of drug administration
    - ◆ discontinuation of drug administration
    - ◆ dose reduction
  - ➢ change in infusion rate
    - ◆ infusion rate unchanged
    - ◆ infusion rate increased
    - ◆ infusion rate decreased
  - ➢ reason for taking measures
    - ◆ poor drug compliance
    - ◆ adverse event
    - ◆ not applicable
    - ◆ others

Details on the drug administration in each cycle of each subject, including treatment arm, subject number, cycle, whether medication is taken, planned dose, actual dose, start date and time, end date and time, dose adjustment, changes in infusion rate and reasons for taking measures, start date and name of adverse event (if adverse events is the reason for taking measures), will be listed.

Details on the exposure of each subject to drug injections, including subject number, date of first injection, date of last injection, number of days of treatment, total number of injections, compliance (%) and category (< 80%, 80% – 120%, > 120) during treatment period, mean interval (days) and mean infusion time (minutes), will be listed.

5.4.2 New Anti-tumor Therapy for Esophageal Squamous Cell Carcinoma during Study

Period

5.4.2.1 NEW SURGICAL THERAPY FOR ESOPHAGEAL SQUAMOUS CELL CARCINOMA DURING STUDY PERIOD

Continuous indicators will be statistically described using the non-missing number of subjects, arithmetic mean, standard deviation, median, minimum and maximum, mainly including:

- time from first drug administration to subsequent surgical therapy for esophageal squamous cell carcinoma (months, calculated as: (first recorded start date of new surgical therapy - date of first drug administration + 1) / 30.4375), if the subject has multiple new surgical therapy records, the record closest to the first study drug administration will be included in the summary

Categorical indicators will be statistically described using the number and percentage of subjects according to the categories on the CRF, mainly including:

- whether any subsequent surgical therapies related to esophageal squamous cell carcinoma are received
- purpose of surgery

History of subsequent surgical therapies for esophageal squamous cell carcinoma will be coded using the ICH Medical Dictionary for Regulatory Activities (MedDRA) 23.0 or later versions, the number and percentage of subjects will be summarized according to System Organ Class (SOC) and Preferred Term (PT), and sorted by SOC > PT in descending order based on the total number of subjects.

Whether a subject receives subsequent surgical therapy for esophageal squamous cell carcinoma, name of surgery, purpose of surgery, date of surgery, System Organ Class (SOC) and Preferred Term (PT) will be listed by treatment arm.

5.4.2.2 New Radiotherapy for Esophageal Squamous Cell Carcinoma during Study Period

Continuous indicators will be statistically described using the non-missing number of subjects, number of cases (if necessary), arithmetic mean, standard deviation, median, minimum and maximum, mainly including:

- time from first drug administration to subsequent radiotherapy for esophageal squamous cell carcinoma (months, calculated as: (first recorded start date of subsequent radiotherapy - date of

first drug administration + 1) / 30.4375), if the subject has multiple subsequent radiotherapy records for esophageal squamous cell carcinoma, the record closest to the first study drug administration will be included in the summary

- duration of radiotherapy (days, calculated as: end date of last radiotherapy - start date of first radiotherapy + 1), only summarized among subjects without missing start date after imputation, and if radiotherapy is still ongoing as of the analysis cut-off date, the analysis cut-off date will be imputed if the subject has multiple subsequent radiotherapy records for esophageal squamous cell carcinoma, the duration is the total duration of new radiotherapies
- radiotherapy dose (Gray), if the subject has multiple subsequent radiotherapy records for esophageal squamous cell carcinoma, the radiotherapy dose is the sum of the doses of the subsequent radiotherapies

Categorical indicators will be statistically described using the number and percentage of subjects according to the categories on the CRF, mainly including:

- whether there is subsequent radiotherapy for esophageal squamous cell carcinoma
- type of radiotherapy
- radiotherapy site

Whether there is subsequent radiotherapy for esophageal squamous cell carcinoma, type of radiotherapy, radiotherapy site, cumulative total radiotherapy dose, start date, and end date will be listed by treatment arm.

5.4.2.3 Systemic Therapy for Esophageal Squamous Cell Carcinoma during Study Period

Continuous indicators will be statistically described using the non-missing number of subjects, arithmetic mean, standard deviation, median, minimum and maximum, mainly including:

- time from first drug administration to subsequent systemic therapy for esophageal squamous cell carcinoma (months, calculated as: (first recorded start date of subsequent systemic therapy - date of first drug administration + 1) / 30.4375), if the subject has multiple subsequent systemic therapy records for esophageal squamous cell carcinoma, the record closest to the first study drug administration will be included in the summary
- duration of treatment (days, calculated as: end date of subsequent systemic therapy - start

date + 1), if the subject has multiple subsequent systemic therapy records for esophageal squamous cell carcinoma, the duration of treatment is the sum of the duration of the subject's systemic therapies; the duration will only be summarized among subjects who have non-missing start dates after imputation and if the systemic therapy is still ongoing as of the analysis cut-off date, the analysis cut-off date shall be imputed

Categorical indicators will be statistically described using the number and percentage of subjects according to the categories on the CRF, mainly including:

- whether there is any subsequent systemic therapy related to esophageal squamous cell carcinoma
- type of treatment
- reason for change in treatment

The names of subsequent systemic therapies for esophageal squamous cell carcinoma will be coded using the Anatomical Therapeutic Chemical classification system (ATC) in the World Health Organization Drug Dictionary (WHODD) B3 2020 Mar or later versions. The number and percentage of subjects with subsequent anti-tumor systemic therapy will be summarized according to the highest codable ATC classification level and Preferred Name (PN) by treatment arm and total. For example, ATC Level 4 (chemical classification) is preferred. If Level 4 is not available, ATC Level 3 (pharmacological classification) will be used for coding and summary, so on and so forth. They will be sorted by ATC > PN in descending order based on the total number of subjects.

Whether there is subsequent systemic therapy for esophageal squamous cell carcinoma, including type of treatment, therapeutic drug, reason for change in treatment, start date, whether ongoing and end date will be listed by treatment arm.

5.4.2.4 Other Therapy for Esophageal Squamous Cell Carcinoma during Study Period

Continuous indicators will be statistically described using the non-missing number of subjects, number of cases (if necessary), arithmetic mean, standard deviation, median, minimum and maximum, mainly including:

- time from first drug administration to other subsequent therapy for esophageal squamous cell carcinoma (months, calculated as: (first recorded start date of other subsequent therapy - date of first drug administration + 1) / 30.4375), if the subject has multiple other subsequent therapy records

for esophageal squamous cell carcinoma, the record closest to the first study drug administration will be included in the summary

Categorical indicators will be statistically described using the number and percentage of subjects according to the categories on the CRF, mainly including:

- whether there is any other subsequent therapies related to esophageal squamous cell carcinoma
- purpose of treatment

Whether there is a history of other subsequent therapy for esophageal squamous cell carcinoma, including the name of treatment, purpose of treatment and date of treatment, will be listed by treatment arm.

5.4.3 Concomitant Medications

Concomitant medications are defined as all medications with a start date on or after informed consent signing, or a start date before informed consent signing and an end date after informed consent signing, or a start date before informed consent signing and without a recorded end date but which use is recorded as ongoing, up to 30 ± 7 days after the last study drug treatment (the concomitant treatment related to AE is recorded up to 90 ± 7 days after the last study treatment), or the initiation of new anti-tumor therapy (whichever occurs first). If a certain medication cannot be classified as "prior medication" or "concomitant medication" after the missing medication date is imputed according to the rules of "5.1.3.3 Missing Date of Prior/Concomitant Medication/ Procedure ", it will be deemed as "concomitant medication.

Concomitant medications will be coded using World Health Organization Drug Dictionary (WHODD) B3 2020 Mar or later versions. The number and percentage of subjects with at least 1 concomitant medication during the study will be summarized by treatment arm and total, the number and percentage of subjects with concomitant medication will be summarized by therapeutic classification (ATC 2) and Preferred Name (PN), and sorted by ATC 2 > PN in descending order based on the total number of subjects.

Details of the subject's concomitant medication, including the name of the drug, indications, single dose and unit, frequency of administration, route of administration, start date, whether ongoing and end date, etc. will be listed by treatment arm, and marked as "concomitant medication".

5.4.4 Concomitant Procedures

Concomitant procedure is defined as procedure with a start date on or after informed consent signing, or a start date before informed consent signing and end date after informed consent signing, or a start date before informed consent signing and without a recorded end date of procedure but which use is recorded as ongoing, until 30 ± 7 days after the last study drug treatment (the concomitant procedure related to AE is recorded until 90 ± 7 days after the last study treatment), or the initiation of a new anti-tumor therapy (whichever occurs first). If a certain procedure cannot be classified as "prior procedure" or "concomitant procedure" after the missing treatment date is imputed according to the rules of "5.1.3.3 Missing Date of Prior/Concomitant Medications/Procedure", it will be deemed as "concomitant procedure".

Concomitant procedures will be coded using the ICH Medical Dictionary for Regulatory Activities (MedDRA) 23.0 or later versions. The number and percentage of subjects with at least 1 concomitant procedure during the study will be described by treatment arm and total, the number and percentage of subjects that used procedure will be summarized by System Organ Class (SOC) and Preferred Term (PT), and sorted by SOC > PT in descending order based on the total number of subjects.

Details of the subject's concomitant procedures, including the name of the treatment, indications, start date, whether ongoing and end date, etc. will be listed by treatment arm, and marked as "concomitant procedure".

5.5 EFFICACY ANALYSIS

5.5.1 Primary Efficacy Analysis

This study uses PFS and OS as parallel dual primary endpoints, of which an interim analysis of OS shall be performed when the target number of PFS events have occurred and the final analysis shall be performed when the target number of OS events have occurred. PFS will only be analyzed once during final analysis.

5.5.1.1 Progression-Free Survival (PFS)

Analysis population: The primary analysis population of this study is the intention-to-treat set (ITT),

defined as all subjects randomized and enrolled in the trial. Analysis will be based on the randomized treatment arms.

5.5.1.1.1 Main Estimation

The hypothesis test for PFS in this study is:

H_0: St(t) ≤ Sp(t)

H_1: St(t) > Sp(t)

St(t) represents the PFS survival probability function of the HLX10 arm, and Sp(t) represents the PFS survival probability function of the control arm.

The efficacy data assessed by the Independent Radiology Review Committee (IRRC) according to RECIST v1.1 (except for death)and the stratified log-rank test will be used for the primary analysis. The stratification factors are: PD-L1 expression level (CPS < 10 versus CPS ≥ 10), age (≥65 years old versus < 65 years old), and tumor status (locally advanced versus distant metastasis). The significance level of PFS is two-sided 0.01 (final analysis). HR and its 95% confidence interval will be estimated using the stratified COX proportional hazards model; median PFS and 95% confidence interval will be estimated using Kaplan Meier method (based on Brookmeyer-Crowley method with log-log transformation (default option in SAS is CONFTYPE = LOGLOG)), standard error will be estimated using Greenwood's formula, and Kaplan-Meier curves will be plotted. Kaplan-Meier method will be used to calculate the 6, 9, and 12-month progression-free survival rates and the 95% confidence interval.

Reference program (adjusted according to actual situation):

```
proc lifetest data=anadata method=km outsurv=surv timelist=(6,9,12) reduceout conftype=loglog;
    time Time*Status(1); /*1 indicates censoring*/
    strata Trt/test=logrank;
run;


proc phreg data=adadata;
    class Trt(ref='0');
    model Time*Status(1)=Trt/risklimits=pl alpha=0.05 ties=efron; /*1 indicates censoring*/
    strata pdl1cat agecat tumstat;
```

```
    hazardratio Trt/diff = ref;
run;
```

Refer to the table below for PFS censoring principles.

#	Indicator	Situation	Event Date or Censoring Date	Censored
1	PFS	Progressive disease (PD) or death observed during the study	Event date: date of first PD or date of death, whichever occurs first	No
2.1	PFS	No progressive disease is observed before initiation of new anti-tumor therapy<br>Note: New anti-tumor therapy includes surgical therapy, radiotherapy, and systemic therapy	Reference situation:<br>（1） No tumor imaging assessment is performed after randomization before initiation of new anti-tumor therapy<br>Treatment: Censor on date of randomization<br>（2） No progressive disease is observed before initiation of new anti-tumor therapy during imaging follow-up period (i.e., at least 1 tumor imaging examination result is available)<br>Treatment: Censor on date of last imaging examination before initiation of new anti-tumor therapy	Yes
2.2	PFS	Emergency unblinding or accidental unblinding due to SAE/special circumstances or emergency/other reason	Reference situation:<br>（1） No tumor imaging assessment or death after randomization before emergency unblinding or accidental unblinding due to SAE/special circumstances or emergency/other reasons<br>Treatment: Censor on date of randomization<br>（2） Emergency unblinding or accidental unblinding due to SAE/special	Yes

			circumstances or emergency/other reason during imaging follow-up period (i.e., at least 1 tumor imaging result is available)<br>Treatment: Censor on the date of the last tumor imaging examination before emergency unblinding or accidental unblinding<br>Note: Censoring rules for "emergency unblinding or accidental unblinding due to SAE/special circumstances or emergency/other reason" apply only to the circumstances specified in methodsSensitivity Analysis 3, 4 and 8	
3	PFS	Progressive disease or death after missing ≥2 consecutive scheduled imaging visits	Reference situation:<br>（1）As of the analysis cut-off date, progressive disease or death is observed after 2 or more imaging visit cycles (including imaging examinations once every 6 weeks for the first 48 weeks and imaging examinations once every 12 weeks after 48 weeks that are not performed) from the subject's randomization date, and there is no tumor imaging assessment during the period<br>Treatment: Censor on date of randomization<br>（2）Progressive disease or death is observed after missing tumor imaging examinations of 2 or more consecutive imaging follow-up cycles (including imaging examinations once every 6 weeks for the first 48 weeks and imaging examinations once every 12 weeks after 48 weeks that are not performed) during	Yes

			imaging follow-up period (i.e., at least 1 tumor imaging examination result is available)<br>Treatment: Censor on the date of the last tumor imaging examination before the missing examination	
4	PFS	No progressive disease or death is observed before the end of study/analysis cut-off date/dropout date	Reference situation:<br>Study has ended or study has not ended but treatment/follow-up is still ongoing as of the analysis cut-off date, and no progressive disease or death is observed<br>Treatment: Censor on the date of last tumor imaging examination before the end of study/analysis cut-off date/dropout date of the subject	Yes
5	PFS	No post-baseline imaging examination	Reference situation:<br>No tumor imaging assessment from randomization to the end of the study of the subject<br>Treatment: Censor on date of randomization	Yes
6	PFS	Major protocol violations that affect efficacy assessment	All major protocol violations that may affect the efficacy assessment will be discussed and confirmed at the data review meeting, and a decision will be made to (1) censor on the date of the last tumor imaging examination before the major protocol violation event; (2) censor on the date of the major protocol violation event; (3) exclude the data of this subject from the analysis set; (4) use other jointly approved treatment methods.	Yes

Note: 1. Categorize according to the priority order of censoring rules 2.1 – 5, categorize 2.1 and 2.2 according to

the "initiation of new anti-tumor therapy" and "emergency unblinding or accidental unblinding", whichever occurs first, and categorize 6 according to the processing method confirmed in the data review meeting.

2. If the intercurrent event occurs on the same day as the imaging examination/death, the imaging examination/death on the same day will still be included in the calculation and assessment of PFS.

5.5.1.1.2 Sensitivity Analysis

#	Description of Assumptions and Estimation Method
Sensitivity Analysis 1	In order to evaluate the robustness of efficacy evaluation in the ITT population, analysis based on the mITT population will be used for sensitivity analysis, and the analysis method is the same as the .Main Estimation
Sensitivity Analysis 2	In order to evaluate the robustness of progressive disease assessed using RECIST v1.1 after the use of immunotherapy drugs, IRRC-assessed PFS according to iRECIST will be used for sensitivity analysis, and the analysis method is the same as the Main Estimation.<br>Progressive disease will be determined according to the guidelines for use of iRECIST and the following rules:<br>（1）the time of first iUPD is the time of progressive disease if iUPD occurs for the first time, followed by iCPD;<br>（2）the time of first iUPD is the time of progressive disease if iUPD occurs for the first time, followed by death;<br>（3）the time of first iUPD is the time of progressive disease if iUPD occurs for the first time, the subject discontinues the treatment specified by the protocol or does not undergo further assessment thereafter, or is assessed to be iUPD thereafter;<br>（4）this iUPD will not be considered progressive disease if iUPD occurs for the first time and iCR/iPR/iSD occurs thereafter, and imaging visits will continue until the next assessment of iUPD; whether the time of the next iUPD is the time of progressive disease shall be based on rules (1) to (3).
Sensitivity Analysis 3	IRRC-assessed PFS will be censored on the date of the last tumor imaging examination

	before emergency unblinding or accidental unblinding due to other reasons for the sensitive analysis of the main estimation, so as to assess the impact of emergency unblinding or accidental unblinding of investigators due to other reasons on IRRC imaging assessment. The analysis method is the same as the .main estimation
Sensitivity Analysis 4	Due to incorrect drug setting in IWRS, some subjects in the control arm were incorrectly administered with HLX10 in combination with chemotherapy, so the subjects with drug dispensing error will be included into the HLX10 arm for sensitivity analysis based on ITT and mITT analyses. In addition, the 2 situations where IRRC imaging assessment is not affected and affected by emergency unblinding or accidental unblinding of investigators due to other reasons shall be considered. The analysis method is the same as the .main estimation
Sensitivity Analysis 5	When the assumption of “Baseline risk varies in each strata, but has a common proportional hazard ratio” is changed to “Baseline risk is the same in each strata, and has a common proportional hazard ratio” in the main estimation, the unstratified Cox regression analysis method will be used to carry out sensitivity estimation of the therapeutic effects accordingly. This analysis corresponds to the same estimands as the primary analysis method and aims to examine the heterogeneity assumptions in the primary analysis and to explore whether the overall results are robust.
Sensitivity Analysis 6	As the study drug is an immunosuppressant (PD-1), there may be a delayed effect or tailing effect. Comparison in primary estimation is carried out using the Max-Combo test for sensitivity analysis. The Max-Combo test is based on 4 Fleming-Harrington (FH) weights, namely FH (0, 0), FH (0, 1), FH (1, 0) and FH (1, 1). Refer to Reference 7.<br>Reference program:<br>proc lifetest data=anadata;<br>time Time*Status(1); /*1 indicates censoring*/<br>strata Trt / test=FH(0,0 0,1 1,0 1,1);<br>run;

Sensitivity Analysis 7	If important baseline characteristics (ECOG score, sex, MSI/MMR (MSS/MSI-L or MSI-H), TMB (< 10 muts/Mb or ≥10 muts/Mb)) are not balanced between the arms, the factors of heterogeneity will be included as covariates in the stratified Cox regression analysis.
Sensitivity Analysis 8	In order to evaluate the robustness of IRRC-assessed progressive disease, the PFS efficacy assessment results of the investigator under the above circumstances will be used for sensitivity analysis. The analysis method is the same as the .main estimation The investigator may learn about subjects' grouping after emergency unblinding or accidental unblinding due to other reasons. Hence, the investigator may be biased in the assessment of imaging results. Therefore, investigator-assessed PFS only considers the situation where PFS is censored on the last tumor imaging date before emergency unblinding or accidental unblinding due to other reasons (i.e., the situation in methodsSensitivity Analysis 3 and 4, where PFS is censored on the last tumor imaging date before emergency unblinding or accidental unblinding due to other reasons).

5.5.1.1.3 Supplementary Analysis

#	Description of Assumptions and Estimation Method
Supplementary analysis 1	The PPS population will be used for analysis, and the statistical analysis method is the same as that of the primary statistical analysis. The PPS is defined as a subset of ITT, and all randomized and enrolled subjects who did not experience major protocol deviations that significantly affect the primary efficacy assessment constitute the per-protocol set. The specific definition of the per-protocol set population shall be confirmed before database lock.
Supplementary analysis 2	If the proportional hazards assumption (PH assumption) in the primary statistical analysis is not established, the 6-month restricted mean survival time (RMST) and 12-month restricted mean survival time (RMST) will be used for supplementary analysis.<br>Reference program:

	proc lifetest data=anadata plots=(rmst) rmst(CL tau=6) ;<br>time Time*Status(1); /*1 indicates censoring*/<br>strata Trt;<br>run;

5.5.1.2 Overall Survival (OS)

Analysis population: Same as the analysis population in "5.5.1.1 Progression-free Survival (PFS)".

5.5.1.2.1 Main Estimation

The hypothesis test for OS in this study is:

H_0: St(t) ≤ Sp(t)

H_1: St(t) ＞ Sp(t)

St(t) represents the OS survival probability function of the HLX10 arm, and Sp(t) represents the OS survival probability function of the control arm.

The stratified log-rank test will be used for the primary analysis, and the stratification factors are: PD-L1 expression level (CPS ＜ 10 versus CPS ≥ 10), age (≥65 years old versus ＜ 65 years old), and tumor status (locally advanced versus distant metastasis). The significance levels of OS are two-sided 0.010 (interim analysis) and two-sided 0.037 (final analysis), respectively. HR and its 95% confidence interval will be estimated using stratified COX proportional hazards model; median OS and 95% confidence interval will be estimated using Kaplan Meier method (based on Brookmeyer-Crowley method with log-log transformation (default option in SAS is CONFTYPE = LOGLOG)), standard error will be estimated using Greenwood's formula, and Kaplan-Meier curves will be plotted. Kaplan-Meier method will be used to calculate the 6, 12, 18, and 24-month survival rates and the 95% confidence interval.

The reference program is the same as that in "5.5.1.1 Progression-free Survival (PFS)".

Refer to the table below for OS censoring principles.

#	Indicator	Situation	Event Date or Censoring Date	Censored
1	OS	Death observed during the study	Event date: date of death	No
2	OS	Still surviving as of the end of study/analysis cut-off date	Treatment: Censor on the earlier of the last confirmed date of survival of the subject and the end of study/analysis cut-off date	Yes
3	OS	Loss to follow-up	Treatment: Censor on the last confirmed date of survival of the subject	Yes
4	OS	Early withdrawal from the study due to reasons other than loss to follow-up	Treatment: Censor on the date of withdrawal from the study	Yes

Note: The last confirmed date of survival of the subject is defined as the latest complete date (including the date of imaging examination uploaded to the IRRC and excluding the survival follow-up date with survival status of "unknown/lost to follow-up" or "death" in the CRF, the follow-up date 90 days after the last drug administration, the end date of treatment with HLX10/placebo/cisplatin/fluorouracil due to "loss to follow-up", and the date of early withdrawal from the study due to the main reason of "subject lost to follow-up") that can be obtained from all events/finding tests or assessments related to the subject in all collected data.

5.5.1.2.2 Sensitivity Analysis

#	**Description of Assumptions and Estimation Method**
Sensitivity Analysis 1	In order to evaluate the robustness of efficacy evaluation in the ITT population, analysis based on the mITT population will be used for sensitivity analysis, and the analysis method is the same as the .main estimation
Sensitivity Analysis 2	Due to incorrect drug setting in the IWRS, some subjects in the control arm were incorrectly administered with HLX10 in combination with chemotherapy, so the subjects with drug dispensing error will be included into the HLX10 arm for sensitivity analysis based on ITT and mITT analyses. The analysis method is the same as the .main estimation
Sensitivity Analysis 3	When the assumption of "Baseline risk varies in each strata, but has a common proportional hazard ratio" is changed to "Baseline risk is the same in each strata, and has

	a common proportional hazard ratio" in the main estimation, the unstratified Cox regression analysis method will be used to carry out sensitivity estimation of the therapeutic effects accordingly. This analysis corresponds to the same estimands as the primary analysis method and aims to examine the heterogeneity assumptions in the primary analysis and to explore whether the overall results are robust.
Sensitivity Analysis 4	As the study drug is an immunosuppressant (PD-1), there may be a delayed effect or tailing effect. Comparison in primary estimation is carried out using the Max-Combo test for sensitivity analysis. The Max-Combo test is based on 4 Fleming-Harrington (FH) weights, namely FH (0 ,0), FH (0, 1), FH (1, 0) and FH (1, 1). Refer to Reference 7.<br>Reference program:<br>proc lifetest data=anadata;<br>time Time*Status(1); /*1 indicates censoring*/<br>strata Trt / test=FH(0,0 0,1 1,0 1,1);<br>run;
Sensitivity Analysis 5	If important baseline characteristics (ECOG score, sex, MSI/MMR (MSS/MSI-L or MSI-H), TMB (< 10 muts/Mb or ≥10 muts/Mb)) are not balanced between the arms, the factors of heterogeneity will be included as covariates in the stratified Cox regression analysis.

5.5.1.2.3 Supplementary Analysis

#	Description of Assumptions and Estimation Method
Supplementary analysis 1	The PPS population will be used for analysis, and the statistical analysis method is the same as that of the primary statistical analysis. The PPS is defined as a subset of ITT, and all randomized and enrolled subjects who did not experience major protocol deviations that significantly affect the primary efficacy assessment constitute the per-protocol set. The specific definition of the per-protocol set population shall be confirmed before database lock.

Supplementary analysis 2	If the proportional hazards assumption (PH assumption) in the primary statistical analysis is not established, the 12-month restricted mean survival time (RMST) and 24-month restricted mean survival time (RMST) will be used for supplementary analysis.<br>Reference program:<br>proc lifetest data=anadata plots=(rmst) rmst(CL tau=6) ;<br>time Time*Status(1); /*1 indicates censoring*/<br>strata Trt;<br>run;

The final analysis of the PFS indicator and interim analysis of the OS indicator shall be performed when the number of target PFS events have been observed. At this time, when the *p*-value (two-sided) in the final analysis of PFS based on the log-rank test is < 0.01 or the *p*-value (two-sided) of the interim analysis of OS is < 0.010, the difference in the efficacy between the HLX10 in combination with chemotherapy (cisplatin + 5-FU) arm and placebo in combination with chemotherapy (cisplatin + 5-FU) arm is statistically significant. Alternatively, the final analysis of OS shall be performed when the target number of OS events have been observed (approximately 388 cases). At this time, when the *p*-value (two-sided) in the final analysis of OS based on the log-rank test is < 0.037, the difference in efficacy between the HLX10 in combination with chemotherapy (cisplatin + 5-FU) arm and placebo in combination with chemotherapy (cisplatin + 5-FU) arm is statistically significant. The O'Brien-Fleming type α-spending function (using the Lan-DeMets method to approximate) shall be used to adjust the test level and control overall type I error rate if the actual number of observed events is smaller than or exceeds the number of planned events at the time of interim/final analysis.

5.5.2 Secondary Efficacy Analysis

The secondary efficacy endpoints of this study include the objective response rate (ORR) and duration of response (DOR).

5.5.2.1 Objective Response Rate (ORR)

Analysis population: Same as the analysis population in "5.5.1.1 Progression-free Survival (PFS)".

5.5.2.1.1 Main Estimation

The IRRC will summarize the number and percentage of subjects with best overall response (BOR) according to RECIST v1.1 criteria. The best overall response is the overall tumor response assessment (according to RECIST v1.1) during the medication period, and is based on the following priority levels: 1. complete response (CR)＞ 2.partial response (PR) ＞3. stable disease (SD) ＞4.progressive disease (PD) ＞5.non-evaluable (NE). If SD is ≥ 42 days from the date of randomization, i.e., if the time from randomization to the best overall response assessment of SD is ＜ 42 days, subjects with stable disease will not be included.

IRRC-assessed efficacy data according to RECIST v1.1 will be used for the primary analysis. The ORR difference between the two arms and its 95% confidence interval will be tested using the stratified Cochran-Mantel-Haenszel (CMH) method. The stratification factors are: PD-L1 expression level (CPS ＜ 10 versus CPS ≥ 10), age (≥65 years old versus ＜ 65 years old), and tumor status (locally advanced versus distant metastasis). The true stratification factor values collected will be used for the analysis. Clopper Pearson method will be used to calculate 95% CI of each treatment arm.

At the same time, disease control rate (DCR) will be analyzed. DCR is defined as the proportion of subjects with IRRC-assessed best overall response (BOR) of CR, PR or SD according to RECIST v1.1. The statistical analysis method is the same as that for ORR.

5.5.2.1.2 Sensitivity Analysis

#	Description of Assumptions and Estimation Method
Sensitivity Analysis 1	In order to evaluate the robustness of efficacy evaluation in the ITT population, analysis based on the mITT population will be used for sensitivity analysis, and the analysis method is the same as the .main estimation
Sensitivity Analysis 2	In order to evaluate the robustness of objective response rate (ORR) and disease control rate (DCR) assessed using RECIST v1.1 after the use of immunotherapy drugs, IRRC-assessed ORR and DCR according to iRECIST will be used for sensitivity analysis, and the analysis method is the same as the .main estimation

Sensitivity Analysis 3	In order to assess the impact of emergency unblinding or accidental unblinding by the investigator due to other reasons on IRRC imaging assessment, the IRRC imaging assessment results after emergency unblinding or accidental unblinding due to other reasons will not be considered for the sensitivity analysis of the primary analysis method. The analysis method is the same as the .main estimation
Sensitivity Analysis 4	Due to incorrect drug setting in the IWRS, some subjects in the control arm were incorrectly administered with HLX10 in combination with chemotherapy, so the subjects with drug dispensing error will be included into the HLX10 arm for sensitivity analysis based on ITT and mITT analyses. In addition, the 2 situations where IRRC imaging assessment is not affected and affected by emergency unblinding or accidental unblinding of investigators due to other reasons shall be considered. The analysis method is the same as the .main estimation
Sensitivity Analysis 5	If important baseline characteristics (ECOG score, sex, MSI/MMR (MSS/MSI-L or MSI-H), TMB ($<$ 10 muts/Mb or $\geq$10 muts/Mb)) are not balanced between the arms, the factors of heterogeneity will be included as covariates in the stratified CMH analysis.
Sensitivity Analysis 6	In order to evaluate the robustness of IRRC-assessed progressive disease, the ORR and DCR efficacy assessment results of the investigator under the above circumstances will be used for sensitivity analysis. The analysis method is the same as the .main estimation The investigator may learn about subjects' grouping after emergency unblinding or accidental unblinding due to other reasons. Hence, the investigator may be biased in the assessment of imaging results. Therefore, investigator-assessed ORR and DCR only considers the tumor imaging assessment results before emergency unblinding or accidental unblinding due to other reasons will be used (i.e., the situation in methodsSensitivity Analysis 3 and 4 where ORR and DCR are affected by emergency unblinding or accidental unblinding due to other reasons).

5.5.2.1.3 Supplementary Analysis

#	Description of Assumptions and Estimation Method
Supplementary analysis 1	The PPS population will be used for analysis, and the statistical analysis method is the same as that of the primary statistical analysis. The PPS is defined as a subset of ITT, and all randomized and enrolled subjects who did not experience major protocol deviations that significantly affect the primary efficacy assessment constitute the per-protocol set. The specific definition of the per-protocol set population shall be confirmed before database lock.

5.5.2.2 Duration of Response (DOR)

Analysis population: Same as the analysis population in “5.5.1.1 Progression-free Survival (PFS)”.

5.5.2.2.1 Main Estimation

DOR will be only analyzed for subjects who achieved CR or PR. The efficacy data (except for death) assessed by the Independent Radiology Review Committee (IRRC) according to RECIST v1.1 and the stratified log-rank test will be used for the primary analysis. The stratification factors are: PD-L1 expression level (CPS $<$ 10 versus CPS $\geq$ 10), age (≥65 years old versus $<$ 65 years old), and tumor status (locally advanced versus distant metastasis). HR and its 95% confidence interval will be estimated using stratified COX proportional hazards model; median DOR and 95% confidence interval will be estimated using Kaplan Meier method (based on Brookmeyer-Crowley method with log-log transformation (default option in SAS is CONFTYPE = LOGLOG)) standard error will be estimated using Greenwood’s formula, and Kaplan-Meier curves will be plotted. Kaplan-Meier method will be used to calculate the 6, 9, 12, 18, and 24-month sustained response rates and the 95% confidence interval.

The reference program is the same as that in “5.5.1.1 Progression-free Survival (PFS)”.

Refer to the table below for DOR censoring principles.

#	Indicator	Situation	Event Date or Censoring Date	Censored
1	DOR	Progressive disease (PD) or death observed during the	Event date: date of first PD or date of death, whichever occurs first	No

		study		
2.1	DOR	No progressive disease is observed before initiation of new anti-tumor therapy<br>Note: New anti-tumor therapy includes surgical therapy, radiotherapy, and systemic therapy	Reference situation:<br>No progressive disease is observed before the initiation of a new anti-tumor therapy after achieving CR or PR for the first time<br>Treatment: Censor on date of last imaging examination before initiation of new anti-tumor therapy	Yes
2.2	DOR	Emergency unblinding or accidental unblinding due to SAE/special circumstances or emergency/other reason	Reference situation:<br>No tumor imaging assessment or death from achieving CR or PR for the first time to emergency unblinding or accidental unblinding due to SAE/special circumstances or emergency/other reason<br>Treatment: Censor on the date of the last tumor imaging examination before emergency unblinding or accidental unblinding<br>Note: Censoring rules for “emergency unblinding or accidental unblinding due to SAE/special circumstances or emergency/other reason” apply only to the circumstances specified in methodsSensitivity Analysis 3, 4 and 7	Yes
3	DOR	Progressive disease or death after missing ≥2 consecutive scheduled imaging visits	Reference situation:<br>Progressive disease or death is observed after missing tumor imaging examinations of 2 or more consecutive imaging follow-up cycles (including imaging examinations once every	Yes

			6 weeks for the first 48 weeks and imaging examinations once every 12 weeks after 48 weeks that are not performed) during imaging follow-up period after achieving CR or PR for the first time<br>Treatment: Censor on the date of the last tumor imaging examination before the missing examination	
4	DOR	No progressive disease or death is observed before the end of study/analysis cut-off date/dropout date	Reference situation:<br>Study has ended or study has not ended but treatment/follow-up is still ongoing as of the analysis cut-off date, and no progressive disease or death is observed<br>Treatment: Censor on the date of last tumor imaging examination before the end of study/analysis cut-off date/dropout date of the subject	Yes
5	DOR	Major protocol violations that affect efficacy assessment	All major protocol violations that may affect the efficacy assessment will be discussed and confirmed at the data review meeting, and a decision will be made to (1) censor on the date of the last tumor imaging examination before the major protocol violation event; (2) censor on the date of the major protocol violation event; (3) exclude the data of this subject from the analysis set; (4) use other jointly approved treatment methods.	Yes

Note: 1. Categorize according to the priority order of censoring rules 2.1 – 4, categorize 2.1 and 2.2 according to the “initiation of new anti-tumor therapy” and “emergency unblinding or accidental unblinding”, whichever

occurs first, and categorize 5 according to the processing method confirmed in the data review meeting.

2. If the intercurrent event occurs on the same day as the imaging examination/death, the imaging examination/death on the same day will still be included in the calculation and assessment of DOR.

5.5.2.2.2 Sensitivity Analysis

#	Description of Assumptions and Estimation Method
Sensitivity Analysis 1	In order to evaluate the robustness of efficacy evaluation in the ITT population, analysis based on the mITT population will be used for sensitivity analysis, and the analysis method is the same as the .main estimation
Sensitivity Analysis 2	In order to evaluate the robustness of progressive disease after objective response assessed using RECIST v1.1 after the use of immunotherapy drugs, IRRC-assessed DOR according to iRECIST will be used for sensitivity analysis, and the analysis method is the same as the main estimation.<br>Progressive disease will be determined according to the guidelines for use of iRECIST and the following rules:<br>（1） the time of first iUPD is the time of progressive disease if iUPD occurs for the first time, followed by iCPD;<br>（2） the time of first iUPD is the time of progressive disease if iUPD occurs for the first time, followed by death;<br>（3） the time of first iUPD is the time of progressive disease if iUPD occurs for the first time, the subject discontinues the treatment specified by the protocol or does not undergo further assessment thereafter, or is assessed to be iUPD thereafter;<br>（4） this iUPD will not be considered progressive disease if iUPD occurs for the first time and iCR/iPR/iSD occurs thereafter, and imaging visits will continue until the next assessment of iUPD; whether the time of the next iUPD is the time of progressive disease shall be based on rules (1) to (3).
Sensitivity Analysis 3	IRRC-assessed DOR will be censored on the date of the last tumor imaging examination before emergency unblinding or accidental unblinding due to other

	reasons for the sensitive analysis of the main estimation, so as to assess the impact of emergency unblinding or accidental unblinding of investigators due to other reasons on IRRC imaging assessment. The analysis method is the same as the .main estimation
Sensitivity Analysis 4	Due to incorrect drug setting in the IWRS, some subjects in the control arm were incorrectly administered with HLX10 in combination with chemotherapy, so the subjects with drug dispensing error will be included into the HLX10 arm for sensitivity analysis based on ITT and mITT analyses. In addition, the 2 situations where IRRC imaging assessment is not affected and affected by emergency unblinding or accidental unblinding of investigators due to other reasons shall be considered. The analysis method is the same as the .main estimation
Sensitivity Analysis 5	When the assumption of “Baseline risk varies in each strata, but has a common proportional hazard ratio” is changed to “Baseline risk is the same in each strata, and has a common proportional hazard ratio” in the main estimation, the unstratified Cox regression analysis method will be used to carry out sensitivity estimation of the therapeutic effects accordingly. This analysis corresponds to the same estimands as the primary analysis method and aims to examine the heterogeneity assumptions in the primary analysis and to explore whether the overall results are robust.
Sensitivity Analysis 6	If important baseline characteristics (ECOG score, sex, MSI/MMR (MSS/MSI-L or MSI-H), TMB (< 10 muts/Mb or ≥10 muts/Mb)) are not balanced between the arms, the factors of heterogeneity will be included as covariates in the stratified Cox regression analysis.
Sensitivity Analysis 7	In order to evaluate the robustness of IRRC-assessed progressive disease, the DOR efficacy assessment results of the investigator under the above circumstances will be used for sensitivity analysis. The analysis method is the same as the .main estimation The investigator may learn about subjects’ grouping after emergency unblinding or accidental unblinding due to other reasons. Hence, the investigator may be biased in the assessment of imaging results. Therefore, investigator-assessed DOR only considers the situation where DOR is censored on the last tumor imaging date before emergency unblinding or accidental unblinding due to other reasons (i.e., the situation

	in methodsSensitivity Analysis 3 and 4, where DOR is censored on the last tumor imaging date before emergency unblinding or accidental unblinding due to other reasons).

5.5.2.2.3 Supplementary Analysis

#	Description of Assumptions and Estimation Method
Supplementary analysis 1	The PPS population will be used for analysis, and the statistical analysis method is the same as that of the primary statistical analysis. The PPS is defined as a subset of ITT, and all randomized and enrolled subjects who did not experience major protocol deviations that significantly affect the primary efficacy assessment constitute the per-protocol set. The specific definition of the per-protocol set population shall be confirmed before database lock.

5.5.3 Biomarker Analysis

To assess the relationship between PD-L1 expression in tumor tissues and efficacy, PD-L1 will be used as a subgroup to explore the differences in efficacy endpoint indicators between different strata.

Exploratory analysis: To explore the relationship between microsatellite instability (MSI), tumor mutational burden (TMB) and efficacy, MSI and TMB will be used as subgroups to explore the differences in efficacy endpoint indicators between different strata.

5.5.4 Subgroup Analysis

Subgroup analyses of efficacy endpoints stratified by important characteristic factors will be provided. The intergroup hazard ratio (HR) and its 95% confidence interval (Efron`s method) of primary efficacy endpoints PFS and OS and the secondary efficacy endpoint DOR, that are estimated by the stratified COX proportional hazard model will be provided, and the intergroup difference will be statistically tested using the stratified log-rank method. The odds ratio (OR) and 95% confidence interval of each strata of the secondary efficacy endpoint ORR will be provided. Subgroup analysis results will be used to create a forest plot. If the subgroup factor itself is one of the randomization

stratification factors, this model will be equivalent to the Cox model, log-rank test or CMH test stratified by the remaining randomization stratification factors. Subgroup analyses includes subgroups defined by stratification factors and subgroups defined by biomarkers (if the population of a certain subgroup is less than 5% of the ITT/mITT/PPS population, separate subgroup analyses will not be performed):

- age ＜ 65 years old vs. ≥ 65 years old
- ECOG score: 0 vs. 1
- sex: male vs female
- PD-L1 expression level: CPS ＜ 10 vs. CPS ≥ 10
- tumor status: locally advanced vs. distant metastasis
- MSI/MMR: microsatellite stable or microsatellite instability-low (MSS/MSI-L) or microsatellite instability-high (MSI-H)
- TMB: ＜ 10muts/Mb vs. ≥ 10muts/Mb

The data collected using CRF or external data will be grouped based on the true values in subgroup analysis.

5.5.5 Other Analyses

Kaplan-Meier method will be used to calculate the median follow-up time of all the subjects and its 95% confidence interval. The reference program is the same as that in “5.5.1.1 Progression-free Survival (PFS)” but grouping is not considered.

Refer to the table below for median follow-up time censoring principles.

#	Indicator	Situation	Event Date or Censoring Date	Censored
1	Median follow-up time	No death is observed before the end of study/analysis cut-off date/dropout date	Event date: end of study/analysis cut-off date/dropout date	No
2	Median follow-up time	Death	Treatment: Censor on date of death	Yes

The number and percentage of subjects with target events and of each censoring type will be

summarized by treatment arm and total.

The waterfall plot of the best response change from baseline in the sum of target lesion diameter and length will be plotted based on best overall response (BOR).

Tumor imaging results assessed by the investigator after the subjects receive treatment, including subject number, visit, whether the subject has new lesions, date and results of overall target lesion assessment, date and results of overall non-target lesion assessment, overall tumor response assessment (RECIST 1.1), and overall tumor response assessment (iRECIST 1.1), will be listed.

The tumor imaging results assessed by the IRRC after the subjects receive treatment, including subject number, visit, whether the subject has new lesions, date and results of overall target lesion assessment, date and results of overall non-target lesion assessment, overall tumor response assessment (RECIST 1.1), and overall tumor response assessment (iRECIST 1.1), will be listed. The target lesions, non-target lesions, and new tumor lesion detection and assessment results will be listed. The assessment results of non-nodal target lesions include the date of non-nodal lesion detection, lesion number, lesion site, site description, sum of longest diameter of non-nodal lesions, and assessment method and results. The assessment results of nodal target lesions include the date of nodal lesion examination, lesion number, lesion site, site description, sum of shortest diameter of nodal lesions, and assessment method and results. The results of non-target lesions include the date of detection, lesion number, lesion site, site description, and assessment method. The results of new lesions include the date of detection, lesion number, lesion site, site description, and assessment method and results.

5.6 SAFETY ANALYSIS

All safety summaries and analyses will be based on the safety set (SS).

On April 26, 2020, the IWRS administrator discovered after receiving the unblinding reminder email due to a subject experiencing SAE that subjects who were randomized to the placebo arm were receiving HLX10 combination chemotherapy. Subsequently, it was discovered in further review that subjects who were 33.3 kg $<$ weight $\leq$ 66.6 kg in the control arm were erroneously set to be in the HLX10 arm in the IWRS system drug settings. As of April 26, 2020, this weight arm enrolled a total of 51 subjects, resulting in some of the subjects in the control arm being erroneously treated with

HLX10 in combination with chemotherapy. On the day the issue was discovered, the system setting error was immediately corrected. Ultimately, some subjects received control arm treatment in a blinded state after receiving HLX10 in combination with chemotherapy for some time. As these subjects who experienced the systematic drug dispensing error received both HLX10 in combination with chemotherapy and the control arm treatment, they will be analyzed separately. The tables for the safety analyses will present five arms:

a. received placebo in combination with chemotherapy throughout
b. received HLX10 in combination with chemotherapy throughout
c. investigational drug was dispensed by mistake (and medication was alternated)
d. received HLX10 (b and c combined)
e. total

5.6.1 Adverse Events

Adverse events (AEs)

Adverse event (AE) refers to any untoward medical occurrence of a patient or clinical study subject administered a pharmaceutical product . There is not necessarily a causal relationship between the adverse event and the treatment. Adverse events include adverse and abnormal signs (includes abnormal tests and test findings), symptoms or diseases temporally related to the use of the (investigational) drug, regardless of whether they are related to the (investigational) drug (according to the ICH definition). The investigator will assess whether the causal relationship between the study drug and an AE is “related”, “probably related”, “unlikely related”, “not related”, or “cannot be determined”. If the relationship between the AE and study drug is not provided, the AE will be considered “possibly related” to the study drug. The degree of severity of adverse events shall be determined to be Grade 1, Grade 2, Grade 3, Grade 4 or Grade 5 according to CTCAE v4.03.

Adverse Events of Special Interest (AESIs)

Adverse events of special interest (AESI) are events of scientific and medical concern of the study drug that may require close surveillance and prompt communication between the sponsor and the investigator. AESIs may be serious or non-serious. AESIs of HLX10 include, but are not limited to, events with potential inflammatory or immune-mediated mechanisms and that may require more frequent surveillance and/or intervention, such as events of steroid, immunosuppressant, and/or

hormone replacement therapy. The AESIs in this study include infusion reactions (infusion-related adverse reactions, IRR) and immune-related adverse events (irAEs).

Immune-related adverse events (irAEs) refer to AEs that are related to drug exposure, are consistent with the immune-mediated mechanism of action and have no other clear etiology.

Serious Adverse Events (SAEs)

Serious adverse events (SAEs) refer to adverse events that meet any one or more of the following criteria during the clinical trial: (1) leading to death; (2) life-threatening; (3) requiring hospitalization or prolongation of current hospitalization; (4) leading to persistent or significant disability/dysfunction; (5) causing congenital anomalies or birth defects of offspring; (6) other important medical events.

Treatment-emergent Adverse Events (TEAEs)

Treatment-emergent adverse events (TEAEs) are defined as adverse events that occur or aggravate during or after the first administration of the study drug (C1D1) until 90 days after the last drug administration or initiation of a new anti-tumor therapy (whichever occurs first) and only SAEs related to the study drug (HLX10/placebo) shall be recorded thereafter.

Adverse Drug Reactions (ADRs)

Adverse drug reactions (ADR) are defined as adverse events with a “related”, “probably related”, “cannot be determined” or missing relationship with HLX10.

General principles for analysis of adverse events:

- Code all adverse events with MedDRA coding system 23.0 or later versions.
- Summarize according to grouping and total.
- For adverse events that recur in the same subject, count the most severe event once when calculating the number of subjects without differentiating severity and take the most severe event of the same event that occurred on the same day when calculating the number of cases based on the actual number of occurrences and drug severity.
- Do not present the percentage for number of cases.
- When TEAEs are summarized according to SOC and PT based on the highest CTCAE grade, if the subject experiences the same AE multiple times, only take the AE with the highest CTCAE grade.

- If the CTCAE grade of a certain adverse event is missing and the subject did not experience CTCAE Grade ≥ 3 AEs under the same SOC and PT, "missing" should be added to the summary list.
- When the TEAE is summarized by SOC and PT based on the relationship with the study drug, if the subject experiences the same AE multiple times, take only the AE most closely related to the study drug for statistical summary.
- If adverse events with an incidence greater than a certain percentage need to be summarized, they will be searched by any treatment arm and any System Organ Class (SOC)/Preferred Term (PT).
- Sort SOC and PT in a descending order based on the total number of occurrences.

Summarize the number of subjects who experienced adverse events, incidence and number of cases by treatment arm and total, mainly including:

- treatment-emergent adverse events (TEAE)
- CTCAE Grade 3 and above TEAEs
- HLX10/placebo-related TEAEs
- CTCAE Grade 3 and above HLX10/placebo-related TEAEs
- treatment-related TEAEs
  - related to cisplatin
  - related to fluorouracil
- serious TEAEs (TESAEs)
- CTCAE Grade 3 and above TESAEs
- HLX10/placebo-related TESAEs
- CTCAE Grade 3 and above HLX10/placebo-related TESAEs
- treatment-related TESAEs
  - related to cisplatin
  - related to fluorouracil
- TEAEs leading to death
- treatment-related TEAEs leading to death
  - related to HLX10/placebo

- related to cisplatin
- related to fluorouracil

- TEAEs leading to temporary drug discontinuation
- CTCAE Grade 3 and above TEAEs leading to temporary drug discontinuation
- HLX10/placebo-related TEAEs leading to temporary drug discontinuation
- CTCAE Grade 3 and above HLX10/placebo-related TEAEs leading to temporary drug discontinuation
- related to HLX10/placebo
  - TEAEs leading to temporary discontinuation of HLX10/placebo
  - TEAEs leading to temporary discontinuation of cisplatin
  - TEAEs leading to temporary discontinuation of fluorouracil
- TEAEs leading to permanent drug discontinuation
- CTCAE Grade 3 and above TEAEs leading to permanent drug discontinuation
- HLX10/placebo-related TEAEs leading to permanent drug discontinuation
- CTCAE Grade 3 and above HLX10/placebo-related TEAEs leading to permanent drug discontinuation
- related to HLX10/placebo
  - TEAEs leading to permanent discontinuation of HLX10/placebo
  - TEAEs leading to permanent discontinuation of cisplatin
  - TEAEs leading to permanent discontinuation of fluorouracil
- adverse events of special interest (AESIs)
  - infusion-related adverse events (IRRs)
  - immune-related adverse events (irAEs)
- CTCAE Grade 3 and above AESIs
  - CTCAE Grade 3 and above IRRs
  - CTCAE Grade 3 and above irAEs
- serious AESIs (SAESIs)
  - serious IRRs
  - serious irAEs (irSAEs)

- CTCAE Grade 3 and above SAESIs
  - CTCAE Grade 3 and above serious IRRs
  - CTCAE Grade 3 and above irSAEs
- TEAEs with an incidence of ≥ 1%
- CTCAE Grade 3 and above TEAEs with an incidence of ≥ 1%
- TESAEs with an incidence of ≥ 1%
- CTCAE Grade 3 and above TEAEs with an incidence of ≥ 1%
- HLX10/placebo-related TEAEs with an incidence of ≥ 1%
- HLX10/placebo-related CTCAE Grade 3 and above TEAEs with an incidence of ≥ 1%
- HLX10/placebo-related TESAEs with an incidence of ≥ 1%
- HLX10/placebo-related CTCAE Grade 3 and above TESAEs with an incidence of ≥1%
- TEAEs with an incidence of ≥ 5%
- CTCAE Grade 3 and above TEAEs with an incidence of ≥ 5%
- TESAEs with an incidence of ≥ 5%
- CTCAE Grade 3 and above TESAEs with an incidence of ≥ 5%
- HLX10/placebo-related TEAEs with an incidence of ≥ 5%
- HLX10/placebo-related CTCAE Grade 3 and above TEAEs with an incidence of ≥ 5%
- HLX10/placebo-related TESAEs with an incidence of ≥ 5%
- HLX10/placebo-related CTCAE Grade 3 and above TESAEs with an incidence of ≥ 5%
- TEAEs with an incidence of ≥ 10%
- CTCAE Grade 3 and above TEAEs with an incidence of ≥ 10%
- TESAEs with an incidence of ≥ 10%
- CTCAE Grade 3 and above TESAEs with an incidence of ≥ 10%
- HLX10/placebo-related TEAEs with an incidence of ≥ 10%
- HLX10/placebo-related CTCAE Grade 3 and above TEAEs with an incidence of ≥ 10%
- HLX10/placebo-related TESAEs with an incidence of ≥ 10%
- HLX10/placebo-related CTCAE Grade 3 and above TESAEs with an incidence of ≥ 10%

Adverse events will be summarized by the number of subjects, incidence and number of events, respectively, according to the coded System Organ Class (SOC) and Preferred Term (PT). The

following adverse events will be summarized using the same method:

- treatment-emergent adverse events (TEAEs) summarized by SOC and PT
- TEAEs summarized by SOC, PT and severity
- CTCAE Grade 3 and above TEAEs summarized by SOC and PT
- TEAEs summarized by SOC, PT and causal relationship with HLX10/placebo
- HLX10/placebo-related TEAEs summarized by SOC and PT
- HLX10/placebo-related TEAEs summarized by SOC, PT and severity
- CTCAE Grade 3 and above HLX10/placebo-related TEAEs summarized by SOC and PT
- TEAEs summarized by SOC, PT and causal relationship with cisplatin
- cisplatin-related TEAEs summarized by SOC and PT
- cisplatin-related TEAEs summarized by SOC, PT and severity
- CTCAE Grade 3 and above cisplatin-related TEAEs summarized by SOC and PT
- TEAEs summarized by SOC, PT, and causal relationship with fluorouracil
- fluorouracil-related TEAEs summarized by SOC and PT
- fluorouracil-related TEAEs summarized by SOC, PT, and severity
- CTCAE Grade 3 and above fluorouracil-related TEAEs summarized by SOC and PT
- serious TEAEs (TESAEs) summarized by SOC and PT
- TESAEs summarized by SOC, PT and severity
- CTCAE Grade 3 and above TESAEs summarized by SOC and PT
- HLX10/placebo-related TESAEs summarized by SOC and PT
- HLX10/placebo-related TESAEs summarized by SOC, PT and severity
- CTCAE Grade 3 and above HLX10/placebo-related TESAEs summarized by SOC and PT
- TEAEs leading to death summarized by SOC and PT
- HLX10/placebo-related TEAEs leading to death summarized by SOC and PT
- cisplatin-related TEAEs leading to death summarized by SOC and PT
- fluorouracil-related TEAEs leading to death summarized by SOC and PT
- TEAEs leading to temporary discontinuation of HLX10/placebo summarized by SOC and PT
- TEAEs leading to temporary discontinuation of HLX10/placebo summarized by SOC, PT

and severity

- CTCAE Grade 3 and above TEAEs leading to temporary discontinuation of HLX10/placebo summarized by SOC and PT
- HLX10/placebo-related TEAEs leading to temporary discontinuation of HLX10/placebo summarized by SOC and PT
- HLX10/placebo-related TEAEs leading to temporary discontinuation of HLX10/placebo summarized by SOC, PT and severity
- Grade 3 and above HLX10/placebo-related TEAEs leading to temporary discontinuation of HLX10/placebo summarized by SOC and PT
- TEAEs leading to temporary discontinuation of cisplatin summarized by SOC and PT
- TEAEs leading to temporary discontinuation of cisplatin summarized by SOC, PT and severity
- CTCAE Grade 3 and above TEAEs leading to temporary discontinuation of cisplatin summarized by SOC and PT
- HLX10/placebo-related TEAEs leading to temporary discontinuation of cisplatin summarized by SOC and PT
- HLX10/placebo-related TEAEs leading to temporary discontinuation of cisplatin summarized by SOC, PT and severity
- Grade 3 and above HLX10/placebo-related TEAEs leading to temporary discontinuation of cisplatin summarized by SOC and PT
- TEAEs leading to temporary discontinuation of fluorouracil summarized by SOC and PT
- TEAEs leading to temporary discontinuation of fluorouracil summarized by SOC, PT and severity
- CTCAE Grade 3 and above TEAEs leading to temporary discontinuation of fluorouracil summarized by SOC and PT
- HLX10/placebo-related TEAEs leading to temporary discontinuation of fluorouracil summarized by SOC and PT
- HLX10/placebo-related TEAEs leading to temporary discontinuation of fluorouracil summarized by SOC, PT and severity

- Grade 3 and above HLX10/placebo-related TEAEs leading to temporary discontinuation of fluorouracil summarized by SOC and PT
- TEAEs leading to permanent discontinuation of HLX10/placebo summarized by SOC and PT
- TEAEs leading to permanent discontinuation of HLX10/placebo summarized by SOC, PT and severity
- CTCAE Grade 3 and above TEAEs leading to permanent discontinuation of HLX10/placebo summarized by SOC and PT
- HLX10/placebo-related TEAEs leading to permanent discontinuation of HLX10/placebo summarized by SOC and PT
- HLX10/placebo-related TEAEs leading to permanent discontinuation of HLX10/placebo summarized by SOC, PT and severity
- Grade 3 and above HLX10/placebo-related TEAEs leading to permanent discontinuation of HLX10/placebo summarized by SOC and PT
- TEAEs leading to permanent discontinuation of cisplatin summarized by SOC and PT
- TEAEs leading to permanent discontinuation of cisplatin summarized by SOC, PT and severity
- CTCAE Grade 3 and above TEAEs leading to permanent discontinuation of cisplatin summarized by SOC and PT
- HLX10/placebo-related TEAEs leading to permanent discontinuation of cisplatin summarized by SOC and PT
- HLX10/placebo-related TEAEs leading to permanent discontinuation of cisplatin summarized by SOC, PT and severity
- Grade 3 and above HLX10/placebo-related TEAEs leading to permanent discontinuation of cisplatin summarized by SOC and PT
- TEAEs leading to permanent discontinuation of fluorouracil summarized by SOC and PT
- TEAEs leading to permanent discontinuation of fluorouracil summarized by SOC, PT and severity
- CTCAE Grade 3 and above TEAEs leading to permanent discontinuation of fluorouracil

summarized by SOC and PT

- HLX10/placebo-related TEAEs leading to permanent discontinuation of fluorouracil summarized by SOC and PT
- HLX10/placebo-related TEAEs leading to permanent discontinuation of fluorouracil summarized by SOC, PT and severity
- HLX10/placebo-related CTCAE Grade 3 and above TEAEs leading to permanent discontinuation of fluorouracil summarized by SOC and PT
- TEAEs of special interest (AESIs) summarized by SOC and PT
- details of AESI summarized by SOC and PT
- AESIs summarized by SOC, PT and severity
- CTCAE Grade 3 and above AESIs summarized by SOC and PT
- details of CTCAE Grade 3 and above AESIs summarized by SOC and PT
- serious AESIs (SAESIs) summarized by SOC and PT
- details of SAESIs summarized by SOC and PT
- SAESIs summarized by SOC, PT and severity
- CTCAE Grade 3 and above SAESIs summarized by SOC and PT
- details of CTCAE Grade 3 and above SAESIs summarized by SOC and PT
- infusion-related TEAEs (IRRs) summarized by SOC and PT
- IRRs summarized by SOC, PT and severity
- CTCAE Grade 3 and above IRRs summarized by SOC and PT
- serious IRRs summarized by SOC and PT
- serious IRRs summarized by SOC, PT, and severity
- CTCAE Grade 3 and above serious IRRs summarized by SOC and PT
- immune-related TEAEs (irAEs) summarized by SOC and PT
- irAEs summarized by SOC, PT, and severity
- CTCAE Grade 3 and above irAEs summarized by SOC and PT
- serious irAEs (irSAEs) summarized by SOC and PT
- serious irSAEs summarized by SOC, PT, and severity
- CTCAE Grade 3 and above irSAEs summarized by SOC and PT

- TEAEs with an incidence of ≥ 1% summarized by SOC and PT
- TEAEs with an incidence of ≥1% summarized by SOC, PT and severity
- CTCAE Grade 3 and above TEAEs with an incidence of ≥ 1% summarized by SOC and PT
- serious TEAEs (TESAEs) with an incidence of ≥ 1% summarized by SOC and PT
- TESAEs with an incidence of ≥ 1% summarized by SOC, PT and severity
- CTCAE Grade 3 and above TESAEs with an incidence of ≥ 1% summarized by SOC and PT
- HLX10/placebo-related TEAEs with an incidence of ≥ 1% summarized by SOC and PT
- HLX10/placebo-related TEAEs with an incidence of ≥1% summarized by SOC, PT and severity
- HLX10/placebo-related CTCAE Grade 3 and above TEAEs with an incidence of ≥ 1% summarized by SOC and PT
- HLX10/placebo-related TESAEs with an incidence of ≥ 1% summarized by SOC and PT
- HLX10/placebo-related TESAEs with an incidence of ≥ 1% summarized by SOC, PT and severity
- HLX10/placebo-related CTCAE Grade 3 and above TESAEs with an incidence of ≥ 1% summarized by SOC and PT
- TEAEs with an incidence of ≥ 5% summarized by SOC and PT
- TEAEs with an incidence of ≥ 5% summarized by SOC, PT and severity
- CTCAE Grade 3 and above TEAEs with an incidence of ≥ 5% summarized by SOC and PT
- serious TEAEs (TESAEs) with an incidence of ≥ 5% summarized by SOC and PT
- TESAEs with an incidence of ≥ 5% summarized by SOC, PT and severity
- CTCAE Grade 3 and above TESAEs with an incidence of ≥ 5% summarized by SOC and PT
- HLX10/placebo-related TEAEs with an incidence of ≥ 5% summarized by SOC and PT
- HLX10/placebo-related TEAEs with an incidence of ≥ 5% summarized by SOC, PT and severity

- HLX10/placebo-related CTCAE Grade 3 and above TEAEs with an incidence of ≥ 5% summarized by SOC and PT
- HLX10/placebo-related TESAEs with an incidence of ≥ 5% summarized by SOC and PT
- HLX10/placebo-related TESAEs with an incidence of ≥ 5% summarized by SOC, PT and severity
- HLX10/placebo-related CTCAE Grade 3 and above TESAEs with an incidence of ≥ 5% summarized by SOC and PT
- TEAEs with an incidence of ≥ 10% summarized by SOC and PT
- TEAEs with an incidence of ≥ 10% summarized by SOC, PT and severity
- CTCAE Grade 3 and above TEAEs with an incidence of ≥ 10% summarized by SOC and PT
- serious TEAEs (TESAEs) with an incidence of ≥ 10% summarized by SOC and PT
- TESAEs with an incidence of ≥ 10% summarized by SOC, PT and severity
- CTCAE Grade 3 and above TESAEs with an incidence of ≥ 10% summarized by SOC and PT
- HLX10/placebo-related TEAEs with an incidence of ≥ 10% summarized by SOC and PT
- HLX10/placebo-related TEAEs with an incidence of ≥ 10% summarized by SOC, PT and severity
- HLX10/placebo-related CTCAE Grade 3 and above TEAEs with an incidence of ≥ 10% summarized by SOC and PT
- HLX10/placebo-related TESAEs with an incidence of ≥ 10% summarized by SOC and PT
- HLX10/placebo-related TESAEs with an incidence of ≥ 10% summarized by SOC, PT and severity
- HLX10/placebo-related CTCAE Grade 3 and above TESAEs with an incidence of ≥ 10% summarized by SOC and PT

For adverse events of special interest (AESIs), Kaplan-Meier method will be used to calculate

- median time to IRR and its 95% confidence interval (only for TEAEs analyzed as IRRs)
- median duration of IRR and its 95% confidence interval (only for TEAEs analyzed as IRRs)

- time to irAE classified by SOC (only for TEAEs analyzed as irAEs)
- duration of irAEs classified by SOC (only for TEAEs analyzed as irAEs)

95% CI will be estimated using Brookmeyer-Crowley method. The outcome of IRRs and that of irAEs classified by SOC will be summarized

The number of subjects, incidence, and number of cases of each outcome of TEAEs (not recovered/not resolved, recovered/resolved, recovered/resolved with sequelae, recovering/resolving, aggravation/worsening, death, and unknown) will be summarized separately by System Organ Class (SOC) and Preferred Term (PT) and overall.

The number and percentage of subjects who died throughout the study, treatment period and follow-up period will be summarized by treatment arm and total.

The MedDRA System Organ Class, Preferred Term, name of adverse event, start time of (investigational drug) drug administration, time of last investigational drug administration, start date/end date, outcome, severity (CTCAE grade), relationship with study drug, measure taken for study drug, measure taken on subject, concomitant medications and concomitant procedures, whether the event caused trial termination, whether it is a SAE, SAE category, whether it is an AESI, and whether it is an infusion-related adverse reaction (IRR) or immune-related adverse event (irAE) of each adverse event will be listed. Lists will include all treatment-emergent and non-treatment-emergent adverse events that occurred during the study.

Subjects who died will be listed separately. The number and percentage of subjects who died during the treatment period (from the first administration of the study treatment to within 90 days after the last administration) and during the follow-up period (outside of 90 days after the last administration of the study treatment) and the number and percentage of subjects under each primary cause of death will be described respectively by treatment arm. The date of death, primary cause of death, related AEs and other information of subjects who died will be listed by treatment arm.

5.6.2 Laboratory Tests

Each laboratory test (routine blood test, blood biochemistry test, routine urine test, coagulation function test, cardiac function marker test, pancreatic enzyme test, thyroid function test, virology test and pregnancy test, etc.) at baseline, at each post-baseline visit, and the difference between the tests at each post-baseline visit and at baseline will be summarized using descriptive statistics. Quantitative

results and qualitative routine urine test results will be listed and summarized separately.

The clinical judgment or normal/abnormal changes before and after drug administration (routine blood test, blood biochemistry test, routine urine test, coagulation function test, cardiac function marker test, pancreatic enzyme test and thyroid function test) at each visit will be summarized using a crosstab.

Of which, the virology test only includes the following subjects: if at screening period (baseline): 1. HBV DNA (< 500 IU/mL or 2,500 copies/mL), and 1. HBsAg (+) or 2 HBcAb (+), hepatitis B 5-item (1). HBsAg (hepatitis B surface antigen) 2). HBsAb (hepatitis B surface antibody) 3). HBeAg (hepatitis B e antigen) 4). HBeAb (hepatitis B e antigen) 5). HbcAb (hepatitis B core antibody)) and HBV DNA tests shall be performed every 2 cycles during the treatment period. If at baseline: 1. HCV antibody (+), and HCV RNA (-), HCV antibody and HCV RNA should be tested every 2 treatment cycles during the treatment period.

Each laboratory test item will be listed by subject number, visit, test date, range of normal values, test result, unit, and clinical significance, etc. Clinically significant abnormal results of each laboratory test will be listed separately or marked. Values outside the clinical reference range will be marked in the data lists as “Normal”, “High”, or “Low”.

Details of the serum pregnancy test at each visit will be listed, and only the subjects tested will be listed.

Details of the laboratory virology test at each visit will be listed, and only the subjects tested will be listed.

5.6.3 12-lead ECG

Continuous indicators will be used for descriptive statistical analysis of 12-lead ECG results at baseline, at each post-baseline visit, and the difference between each post-baseline visit and baseline, using the non-missing number of subjects, arithmetic mean, standard deviation, median, minimum and maximum, mainly including:

- Heart rate (HR, beats/min)
- PR interval (msec)
- QRS duration (msec)
- QT interval (msec)

- QTcF interval (msec)

The number and percentage of subjects with clinically significant changes in 12-lead ECG test results from baseline to each post-baseline visit will be summarized using a crosstab.

The ECG results will be listed according to subject screening number, visit, examination date and event, ECG indicator and clinical judgment by treatment arm and total.

5.6.4 Vital Signs

Continuous indicators will be used for statistical description of vital sign test results at baseline, at each post-baseline visit, and the difference between each post-baseline visit and baseline, using the non-missing number of subjects, arithmetic mean, standard deviation, median, minimum and maximum, mainly including:

- body temperature (°C)
- Pulse (beats/minute)
- Respiratory rate (breaths/minute)
- Systolic blood pressure/diastolic blood pressure (mmHg)
- Weight (kg)

Details of vital sign test results of subjects will be listed by treatment arm.

5.6.5 Physical Examination

The number and percentage of subjects with complete physical examination results at baseline and post-baseline symptom-directed physical examination results will be summarized and analyzed by treatment arm and total.

The physical examination results at each visit, including the date of examination, clinical assessment and description of abnormalities, will be listed.

5.6.6 ECOG Score

The baseline data of ECOG score and the number and percentage of subjects at each visit after using the study drug will be statistically described, the change from before drug administration will be summarized using a crosstab, and the score results at each scheduled visit will be listed.

5.7 PHARMACOKINETIC ANALYSIS

The pharmacokinetic analysis is based on the pharmacokinetics set (PKS).

The serum HLX10 concentration and PK parameters of subjects who received HLX10 treatment will be statistically summarized. The concentration data below the limit of quantification (BLQ) will be included in statistical summary, parameter calculation and plotting as 0.

The non-missing number of subjects, number of BLQ cases, arithmetic mean, standard deviation, coefficient of variation (%), geometric mean, geometric coefficient of variation (%), median, minimum and maximum will be used according to the scheduled sampling time points. If there are fewer than 3 valid data at a certain time point, only the non-missing number of subjects, number of BLQ cases, maximum and minimum will be shown, and the remaining statistics will be marked as NC (not calculated).

The PK parameters of Cycles 1 and 8 will be analyzed, and the PK parameters include:

- maximum concentration (C_{max}): i.e., the concentration within 0.5 hours after the end of the 1st (C1-C_{max}) and 8th (C8-C_{max}) drug administrations;
- trough concentration (C_{trough}): i.e., the concentration within 24 hours before the 2nd (C2-C_{trough}) and 8th (C8-C_{trough}) drug administrations;
- accumulation ratio (R_{ac}_C_{max}): i.e., the ratio of C_{max} after the 8th drug administration to that after the 1st drug administration (round to 3 decimal places);
- accumulation ratio (R_{ac}_C_{trough}): i.e., the ratio of C_{trough} before the 8th drug administration to that before the 2nd drug administration (round to 3 decimal places).

The non-missing number of subjects, arithmetic mean, standard deviation, coefficient of variation (%), geometric mean, geometric coefficient of variation (%), minimum, maximum and median will be used to statistically describe the HLX10 PK parameters of subjects. If a parameter has fewer than 3 valid data, only the non-missing number of subjects, maximum and minimum will be presented, and the remaining statistics will be marked as NC (not calculated).

If there are 3 or more subjects in the HLX10 arm with positive ADA or NAb results (if the subject has at least 1 positive ADA/NAb sample after receiving HLX10 treatment, the subject will be considered as an ADA- or NAb-positive subject), all serum HLX10 concentrations and PK parameters will be analyzed by descriptive statistics stratified by ADA/NAb status (ADA positive, ADA negative, NAb positive, and NAb negative).

The mean and standard deviation of the serum HLX10 concentration (linear) and log-serum HLX10 concentration (semi-log-linear) of the subjects at the scheduled sampling time points will be plotted.

The scheduled sampling time points, actual sampling time and serum HLX10 concentrations of the subjects at each visit and the PK parameters of the subjects will be listed.

5.8 IMMUNOGENICITY ANALYSIS

The immunogenicity analysis is based on the safety set (SS).

The ADA/NAb status (negative, positive) at each visit and overall post-administration status will be summarized.

Overall ADA/NAb-positive subjects are defined as subjects who have at least 1 positive ADA/NAb sample at any time point after receiving HLX10 administration.

The occurrence of TEAEs will be summarized by overall immunogenic status (ADA positive, ADA negative, NAb positive, and NAb negative). Refer to the summary of TEAEs in "5.6.1 Adverse Events" for the summary.

Immunogenicity data, including the date and time of sample collection at each visit, antibody type, and test results will be presented in a list.

5.9 BIOMARKER ANALYSIS

The biomarker test results during the screening period will be summarized and listed using the intention-to-treat (ITT) set and the modified intention-to-treat (mITT) set, and PD-L1 expression levels (negative and positive) and MSI/MMR (microsatellite stability or microsatellite instability-low (MSS/MSI-L) and microsatellite instability-high (MSI-H)) and TMB ($<$ 10 mutations/Mb and $\geq$ 10 mutations/Mb) will be summarized using the number and percentage of subjects. At the same time, the biomarker classification results will be used as subgroup grouping information to analyze the efficacy of different subgroups (refer to "5.5 Efficacy Analysis").

5.10 QUALITY OF LIFE SCORE

Quality of life analysis is based on the intention-to-treat set (ITT), and the analysis of change from baseline will be performed in the population with baseline and at least 1 post-baseline score.

The quality of life scales include EuroQoL 5-Dimension 5-Level (EQ-5D-5L) and European

Organization for Research and Treatment of Cancer Quality of Life Questionnaires (EORTC QLQ-C30 and EORTC QLQ-OES18). The changes in each item of EQ-5D-5L and VAS scores will be described by visit, and the quality of life questionnaire index scores of EORTC QLQ-C30 and EORTC QLQ-OES18 will be calculated by dimension (Fayers et. al., 2001). Descriptive statistics (number of subjects, mean, standard deviation, median, maximum, and minimum) will be used to describe the baseline and post-baseline scores of each EQ-5D-5L item (number and percentage of subjects), the baseline and post-baseline scores of each dimension in each scale, as well as change from baseline between the 2 control arms by visit.

5.10.1 EQ-5D-5L

The EQ-5D-5L scale consists of 5 dimensions (mobility, self-care, usual activities, pain or discomfort, anxiety or depression) and a 100-point EQ-5D visual scale (EQ VAS), with each dimension consisting of 5 rank options (no difficulty, slight difficulty, some difficulty, a lot of difficulty, and extreme difficulty).

MULT8 algorithm using the corrected multiplication model for the Chinese population (Luo et al. 2017) will be used to summarize the EQ-5D-5L score summary index of subjects, which is calculated as:

$$
\begin{aligned}
y = \alpha + \sum_l \left(\sum_d \beta_d c_{dl}\right) L_l + e \\
= \alpha + (\beta_{MO} c_{MO2} + \beta_{SC} c_{SC2} + \beta_{UA} c_{UA2} + \beta_{PD} c_{PD2} + \beta_{AD} c_{AD2}) L_2 \\
+ (\beta_{MO} c_{MO3} + \beta_{SC} c_{SC3} + \beta_{UA} c_{UA3} + \beta_{PD} c_{PD3} + \beta_{AD} c_{AD3}) L_3 \\
+ (\beta_{MO} c_{MO4} + \beta_{SC} c_{SC4} + \beta_{UA} c_{UA4} + \beta_{PD} c_{PD4} + \beta_{AD} c_{AD4}) L_4 \\
+ (\beta_{MO} c_{MO5} + \beta_{SC} c_{SC5} + \beta_{UA} c_{UA5} + \beta_{PD} c_{PD5} + \beta_{AD} c_{AD5}) L_5 + e
\end{aligned}
$$

Of which, MO represents mobility, SC represents self-care, UA represents usual activities, PD represents pain or discomfort, and AD represents anxiety or depression. d represents dimension, l represents the evaluation result of a certain dimension, and c_{dl} represents a dummy variable of dimension d at level l.

When calculating the summary index, the index value = 1 - sum of disutility of the problematic dimension, depending on the options for each dimension. If the EQ-5D-5L score of a certain subject at a certain visit is “1, 3, 2, 5, 4”, the subject’s utility index value for this visit is 1 - (0.116 + 0.045 + 0.302 + 0.215) = 0.322. Refer to Table 3 for the coefficients of each dimension.

Table 3 Disutility Coefficient of EQ-5D-5L Scale

Dimension 1	Coefficient	Dimension 2	Coefficient	Dimension 3	Coefficient	Dimension 4	Coefficient	Dimension 5	Coefficient
MO2	0.066	SC2	0.048	UA2	0.045	PD2	0.058	AD2	0.049
MO3	0.158	SC3	0.116	UA3	0.107	PD3	0.138	AD3	0.118
MO4	0.287	SC4	0.210	UA4	0.194	PD4	0.252	AD4	0.215
MO5	0.345	SC5	0.253	UA5	0.233	PD5	0.302	AD5	0.258

5.10.2 EORTC QLQ-C30

The EORTC QLQ-C30 has 30 items in total, including 5 functioning dimensions, 3 symptom dimensions, 1 global health/QoL dimension, and 6 single items.

The 5 **functioning dimensions** are physical (items 1 to 5), role (items 6 and 7), cognitive (items 20 and 25), emotional (items 21 to 24), and social functioning (items 26 and 27), the 3 **symptom dimensions** are fatigue (items 10, 12 and 18), nausea and vomiting (items 14 and 15) and pain (items 9 and 19), there is 1 **global health/QoL dimension** (items 29 and 30), and the 6 single symptom items are dyspnea (item 8), insomnia (item 11), loss of appetite (item 13), constipation (item 16), diarrhea (item 17) and financial difficulties (item 28). Of which, there are 4 rank options for items 1 through 28 (not at all, a little bit, quite a bit and very much) and 7 rank options for items 29 and 30 (“very poor” to “excellent”).

All dimensions and items are converted to scores of 0 – 100 so that they are comparable. Higher scores in the functioning and global health/QoL dimensions represent high levels of health functioning or quality of life, and higher scores in the symptom dimensions represent more severe symptom problems. Dimension/item score is calculated as follows:

（1） calculate the total RS (raw score) of the dimension

$$RS = (I_1 + I_2 + \cdots + I_n)/n$$

（2） carry out linear transformation to obtain a standardized score S

functioning dimension:

$$S = \left\{1 - \frac{RS - 1}{range}\right\} \times 100$$

symptom dimension/single item:

$$S = \{(RS - 1)/range\} \times 100$$

global health status/QOL:

$$S = \{(RS - 1)/range\} \times 100$$

Of which, range represents the difference between the maximum possible score and minimum possible score of the dimension/item. The range of items 1 to 28 is 3, and the range of items 29 to 30 is 6.

5.10.3 EORTC QLQ-OES18

The EORTC QLQ-OES18 consists of 18 items with 4 symptom dimensions and 6 single symptom items.

The 4 symptom dimensions are dysphagia (item 31 to item 33), trouble with eating (item 37 to item 39), reflux (items 44 and 45), and pain (items 46 to 48), and the 6 single symptom items are dry mouth (item 40), trouble with taste (item 41) trouble in talking (item 43), trouble with coughing (item 42), asphyxia (item 35), and trouble swallowing saliva (item 34).

The calculation method of dimension/single item score is the same as that of EORTC QLQ-C30 symptom dimension/item score.

MMRM (mixed-effect model for repeated measures) will be used to compare the corrected mean LS Mean and 95% CI of the change in overall quality of life score between the two arms throughout the study. The fixed effect is the treatment arm, and the covariates are visits and the interaction between the treatment arm and visit. Visits are used as grade covariates. If there are too many visits, resulting in model instability, consider only including visits with more than 25% of subjects in the model.

Reference program (adjusted according to actual situation):

```
proc mixed data = adqs;
    class treat month pat;
```

```
        model score = treat month treat*month;
        repeated month/ type=un subject=pat(treat) rcorr;
    run;
```

The results of each subject's quality of life scale at each visit will be listed, including the summary of results of each item on the scale and the scores for each dimension.

6. CHANGES TO THE PROTOCOL/STATISTICAL ANALYSIS PLAN

6.1 CHANGES INCONSISTENT WITH THE PROTOCOL

6.1.1 Definition of "Modified Intention-to-treat Set (mITT)"

In April 2020, a subject in this study experienced an SAE and the pharmacovigilance department needed to perform unblinding and decide whether to report the SAE to the regulatory department. On April 26, 2020, the IWRS administrator discovered after receiving the unblinding reminder email that subjects who were randomized to the placebo arm were receiving HLX10 combination chemotherapy. Subsequently, it was discovered in further review that subjects who were 33.3 kg $<$ weight $\leq$ 66.6 kg in the control arm were erroneously set to be in the HLX10 arm in the IWRS system drug settings. As of April 26, 2020, this weight arm enrolled a total of 51 subjects, resulting in some of the subjects in the control arm being erroneously treated with HLX10 in combination with chemotherapy.

On the day the issue was discovered, the system setting error was corrected. On April 30, 2020, ethics submission of 21 affected study sites and informed notification to the affected subjects were initiated. On October 27, 2020, the event was reported to the National Medical Products Administration (NMPA). On November 09, 2020, an IDMC meeting was convened to review the safety data of this study and this event. IDMC recommends to continue the study according to the current plan. The sponsor was requested to actively take measures and further communicate with the ethics committee and CDE, so as to seek guiding opinions. Hence, on November 12, 2020 and December 10, 2020, the leading site's ethics committee convened a 3rd and 4th meeting for this event, respectively, and granted approval to continue the study. It also requested for informed notification and unblinding to be performed on 7 of the affected subjects. As of April 23, 2021, 6 out of the 7 subjects that the leading site's ethics committee requested to unblind were unblinded. One subject

died on November 07, 2020 and the sub-site's ethics committee filed the approval to not perform unblinding. In addition, 2 subjects should be unblinded as per the request of the sub-site's ethics committee. Upon discussion, clinical medical experts believe that the systemic drug dispensing error cannot represent the actual clinical practice and cannot reflect the true therapeutic effects of the HLX10 arm or the control arm in actual clinical practice. Therefore, it is recommended to exclude the efficacy data of subjects who experienced systemic drug dispensing errors from the ITT set and use it as the modified intention-to-treat set (mITT), using ITT as the primary analysis population and mITT as the sensitivity analysis population.

The modified intention-to-treat set (mITT) is defined as all randomized subjects who entered the trial and did not experience systemic drug dispensing errors. The mITT population shall be used as the sensitivity analysis population for efficacy analysis in this study and the mITT population analysis shall be analyzed based on the randomized treatment arms. Subjects who experienced systemic drug dispensing errors are defined as the 51 subjects who were affected by erroneous IWRS settings and assigned to the placebo arm but received HLX10 therapy.

6.1.2 Definition of Estimand

This Statistical Analysis Plan defines the target population, variables, treatment, intercurrent events and treatment strategies, and population-level summaries using estimands as required by ICH-E9(R1), and therefore

（1） The primary efficacy co-indicators in the original protocol, namely "IRRC-assessed PFS according to RECIST 1.1" and "OS" are defined as primary co-estimands, respectively. The secondary efficacy indicators in the original protocol, namely "Progression-free survival (PFS (assessed by IRRC according to iRECIST and assessed by the investigator according to RECIST v1.1 and iRECIST)" are defined as primary estimands for sensitivity analysis.

（2） The secondary efficacy indicators in the original protocol, namely IRRC-assessed objective response rate (ORR) and duration of response (DOR) according to RECIST v1.1 are defined as secondary estimands, and secondary efficacy indicators in the original protocol, namely ORR and DOR assessed by the IRRC according to iRECIST and by the investigator according to RECIST v1.1 and iRECIST, will be used as secondary estimands for sensitivity analysis.

（3） The PPS population shall be used for the supplementary analysis of all analyses.

6.2 CHANGES TO STATISTICAL ANALYSIS PLAN

Not applicable.

7. REFERENCES

[1] ICH. Statistical Principles for Clinical Trials, Guideline E9, 1998. Available at https://www.ema.europa.eu/en/documents/scientific-guideline/ich-e-9-statistical-principles-clinical-trials-step-5_en.pdf

[2] ICH. ICH E3 Guideline: Structure and Content of Clinical Study Reports Questions & Answers, 2012. Available at https://www.ema.europa.eu/en/documents/scientific-guideline/international-conference-harmonisation-technical-requirements-registration-pharmaceuticals-human-use_en-5.pdf

[3] Sun, Steven, et al. "Estimands in hematologic oncology trials.", 2021. Pharmaceutical Statistics

[4] EMA. ICH E9 (R1) addendum on estimands and sensitivity analysis in clinical trials to the guideline on statistical principles for clinical trials Step 5 https://www.ema.europa.eu/en/documents/scientific-guideline/ich-e9-r1-addendum-estimands-sensitivity-analysis-clinical-trials-guideline-statistical-principles_en.pdf

[5] CDE. E9(R1): Estimands and Sensitivity Analysis in Clinical Trials, 2020. https://www.cde.org.cn/main/news/viewInfoCommon/b62a86e3b88b7ec7f3114798a0b5bc4f

[6] DIA China. ICH E9(R1) Blue Book for Estimands and Sensitivity Analysis, 2021.

[7] Combination weighted log-rank tests for survival analysis with non-proportional hazards,2020. https://www.sas.com/content/dam/SAS/support/en/sas-global-forum-proceedings/2020/5062-2020.pdf
