# Supplementary material for: First-line serplulimab or placebo plus chemotherapy in PD-L1-positive esophageal squamous cell carcinoma: a randomized, double-blind phase 3 trial
Source: Nat Med. 2023 Feb 2;29(2):473–82. doi: 10.1038/s41591-022-02179-2 (PMC9941045; doi:10.1038/s41591-022-02179-2)
Supplement: Supplementary file 2 — Reporting Summary [file 41591_2022_2179_MOESM2_ESM.pdf]

Corresponding author(s): Jing Huang

Last updated by author(s): Dec 7, 2022

Reporting Summary

Nature Portfolio wishes to improve the reproducibility of the work that we publish. This form provides structure for consistency and transparency in reporting. For further information on Nature Portfolio policies, see our Editorial Policies and the Editorial Policy Checklist.

Statistics

For all statistical analyses, confirm that the following items are present in the figure legend, table legend, main text, or Methods section.

n/a	Confirmed	
☐	☒	The exact sample size (*n*) for each experimental group/condition, given as a discrete number and unit of measurement
☒	☐	A statement on whether measurements were taken from distinct samples or whether the same sample was measured repeatedly
☐	☒	The statistical test(s) used AND whether they are one- or two-sided<br>*Only common tests should be described solely by name; describe more complex techniques in the Methods section.*
☐	☒	A description of all covariates tested
☐	☒	A description of any assumptions or corrections, such as tests of normality and adjustment for multiple comparisons
☐	☒	A full description of the statistical parameters including central tendency (e.g. means) or other basic estimates (e.g. regression coefficient) AND variation (e.g. standard deviation) or associated estimates of uncertainty (e.g. confidence intervals)
☐	☒	For null hypothesis testing, the test statistic (e.g. F, t, r) with confidence intervals, effect sizes, degrees of freedom and P value noted<br>*Give P values as exact values whenever suitable.*
☒	☐	For Bayesian analysis, information on the choice of priors and Markov chain Monte Carlo settings
☐	☒	For hierarchical and complex designs, identification of the appropriate level for tests and full reporting of outcomes
☐	☒	Estimates of effect sizes (e.g. Cohen's d, Pearson's r), indicating how they were calculated

Our web collection on statistics for biologists contains articles on many of the points above.

Software and code

Policy information about availability of computer code

Data collection	The data in this study were collected using the RAVE Electronic Data Capture (EDC) System version 2018.2.4 by Medidata Solutions, Inc.
Data analysis	The data in this study were analyzed using SAS version 9.4 by SAS Institute Inc.

For manuscripts utilizing custom algorithms or software that are central to the research but not yet described in published literature, software must be made available to editors and reviewers. We strongly encourage code deposition in a community repository (e.g. GitHub). See the Nature Portfolio guidelines for submitting code & software for further information.

Data

Policy information about availability of data

All manuscripts must include a data availability statement. This statement should provide the following information, where applicable:

- Accession codes, unique identifiers, or web links for publicly available datasets
- A description of any restrictions on data availability
- For clinical datasets or third party data, please ensure that the statement adheres to our policy

All requests for data will be reviewed by the leading clinical site (National Cancer Center/National Clinical Research Center for Cancer/Cancer Hospital, Chinese Academy of Medical Sciences and Peking Union Medical College) and the sponsor (Shanghai Henlius Biotech, Inc.) to verify whether the request is subject to any intellectual property or confidentiality obligations. Requests for access to the patient-level data from this study can be submitted via email to

huangjingwg@163.com with detailed proposals for use of information; responses to such requests can be expected within one month. A signed data access agreement with the sponsor is required before accessing shared data.

Human research participants

Policy information about studies involving human research participants and Sex and Gender in Research.

Reporting on sex and gender	Sex was recorded by the investigators according to the identity information provided by the patients. The study enrolled 470 (85%) male and 81 (15%) female patients. Subgroup analyses of the primary endpoints indicated that both male and female patients might benefit from the addition of serplulimab to chemotherapy. No individual-level data were shared in this manuscript.
Population characteristics	Patients with previously untreated inoperable locally advanced or metastatic, PD-L1–positive (CPS ≥1) esophageal squamous cell carcinoma were enrolled. 85% of the enrolled patients were male and 15% were female. 44% of the patients had PD-L1 CPS ≥10. Median age was 64 (interquartile range 57–68) years in both groups.
Recruitment	A total of 976 patients were screened at 70 hospitals in China and 551 were randomly assigned to serplulimab plus chemotherapy group (n=368) or placebo plus chemotherapy group (n=183). With the double-blind, placebo-controlled, randomized trial design, self-selection bias was avoided.
Ethics oversight	The study protocol was approved by the institutional review boards or ethics committees of all participating centers (ethics committee of the leading clinical center: Ethics Committee of National Cancer Center/Cancer Hospital, Chinese Academy of Medical Sciences and Peking Union Medical College). All patients provided written informed consent before entering the study. Patients received compensation as described in detail in the informed consent form.

Note that full information on the approval of the study protocol must also be provided in the manuscript.

Field-specific reporting

Please select the one below that is the best fit for your research. If you are not sure, read the appropriate sections before making your selection.

☒ Life sciences ☐ Behavioural & social sciences ☐ Ecological, evolutionary & environmental sciences

For a reference copy of the document with all sections, see nature.com/documents/nr-reporting-summary-flat.pdf

Life sciences study design

All studies must disclose on these points even when the disclosure is negative.

Sample size	The planned sample size was 540 patients, with 339 PFS events and 388 OS events needed respectively to achieve a power of 80% to show a HR of 0.68 for PFS at a one-sided α level of 0.005 and 0.73 for OS at a one-sided α level of 0.02 for comparison between the serplulimab plus chemotherapy group and the placebo plus chemotherapy group.
Data exclusions	None.
Replication	As a double-blind, placebo-controlled, randomized phase 3 study, no replication is required.
Randomization	Eligible patients were randomly assigned (2:1) using an integrated web response system to receive either serplulimab plus chemotherapy or placebo plus chemotherapy. Randomization was stratified by PD-L1 expression level (CPS ≥10 vs CPS <10), age (≥65 years vs <65 years), and disease status (locally advanced vs distantly metastatic).
Blinding	Patients, investigators, and the sponsor’s study team were masked to group assignment.

Reporting for specific materials, systems and methods

We require information from authors about some types of materials, experimental systems and methods used in many studies. Here, indicate whether each material, system or method listed is relevant to your study. If you are not sure if a list item applies to your research, read the appropriate section before selecting a response.

Materials & experimental systems

n/a	Involved in the study
☐	☒ Antibodies
☒	☐ Eukaryotic cell lines
☒	☐ Palaeontology and archaeology
☒	☐ Animals and other organisms
☐	☒ Clinical data
☒	☐ Dual use research of concern

Methods

n/a	Involved in the study
☒	☐ ChIP-seq
☒	☐ Flow cytometry
☒	☐ MRI-based neuroimaging

Antibodies

Antibodies used	Serplulimab (the investigational product in this study), a fully humanized, selective immunoglobulin G4 monoclonal antibody against PD-1 receptor, Shanghai Henlius Biotech, Inc.; Clone 22C3 (supplied as ready to use), monoclonal mouse anti-PD-L1, PD-L1 IHC 22C3 pharmDx kit, Dako, Agilent.
Validation	PD-L1 IHC 22C3 pharmDx immunohistochemical assay was validated by Dako, Agilent.

Clinical data

Policy information about clinical studies

All manuscripts should comply with the ICMJE guidelines for publication of clinical research and a completed CONSORT checklist must be included with all submissions.

Clinical trial registration	ClinicalTrials.gov, NCT03958890.
Study protocol	The full study protocol is provided in the supplementary information.
Data collection	Between June 19, 2019, and December 17, 2021, 976 patients were screened and 551 were randomized. The data cutoff date for data reported in this manuscript was April 15, 2022. All efficacy and safety data were collected at all participating centers.
Outcomes	The dual primary endpoints were progression-free survival (PFS) assessed by the blinded independent radiological review committee (IRRC) per RECIST v1.1, and overall survival. Secondary endpoints included PFS per IRRC using immune-RECIST (iRECIST), investigator-assessed PFS using RECIST v1.1 and iRECIST, objective response rate, duration of response, safety and tolerability, quality of life, and investigation of the relationship between biomarkers and clinical outcomes.
